# Supplementary material for: A rapid access to aliphatic sulfonyl fluorides
Source: Nat Commun. 2019 Aug 21;10:3752. doi: 10.1038/s41467-019-11805-6 (PMC6704106; doi:10.1038/s41467-019-11805-6)
Supplement: Supplementary file 1 — Supplementary Information [file 41467_2019_11805_MOESM1_ESM.pdf]

Supplementary Information

A rapid access to aliphatic sulfonyl fluorides

Xu *et al.*

Supplementary Methods

General information

Unless stated otherwise, all reactions were carried out under an atmosphere of argon using standard Schlenk techniques. All solvents and reagents were obtained from commercial sources and were purified according to standard procedures before use. Organic solutions were concentrated under reduced pressure on a Büchi rotary evaporator. Chromatographic purification of products was accomplished by flash chromatography on silica gel. The product spots on the thin layer chromatography (TLC) was identified/visualized by fluorescence quenching or potassium permanganate stains. ^{1}H and ^{13}C NMR spectra were recorded on a Bruker Avance 400 (400 MHz and 100 MHz) or a Bruker Avance 500 (500 MHz and 126 MHz) instrument, and are internally referenced to solvent residual signals (note: $CDCl_3$ referenced at 7.26 and 77.00 ppm respectively). Data for ^{1}H NMR were reported as follows: chemical shift (δ, ppm), multiplicity (s = singlet, d = doublet, t = triplet, m = multiplet or unresolved, coupling constant(s) in Hz, integration). Data for ^{13}C NMR are reported in ppm with the internal chloroform signal at 77.00 ppm as a standard. High resolution mass spectra were recorded on Waters Micromass GCT Premier (EI) and Exactive Plus LC-MS (ESI) mass spectrometers. GC measurements were performed on a GCMS-QP2010SE from SHIMADZU. Vinyl sulfonyl fluoride (VSF) was prepared according to the literature procedure.[1]

General procedure for synthesis of NHPI redox-active esters

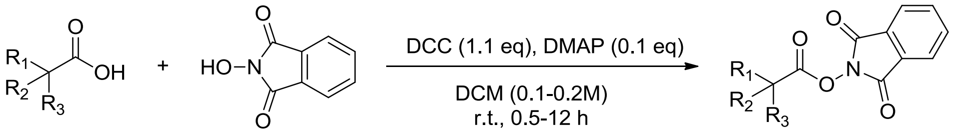

NHPI esters were prepared according to the known procedures.[2] In a round-bottom flask was charged with carboxylic acid (1.0 equiv., if liquid, added via syringe before DCC), *N*-hydroxyphthalimide (1.0 – 1.1 equiv.), and DMAP (0.1 equiv.). Dichloromethane was added (0.1 – 0.2 M), and the mixture was stirred vigorously. DCC (1.1 equiv) was then added and the mixture allowed to stir until the acid was completely consumed (checked by TLC, 0.5 – 12 hours). The mixture was filtered through thin pads of Celite or silica gel, and then rinsed with DCM. The filtrate was collected, and the solvent was removed under reduced pressure. Purification of the resulting residue by column chromatography afforded the desired NHPI redox-active ester products. For the amino acid substrates in Table 2, after passing through a short pad of silica gel (2-3 cm) and concentrated, crude redox-active ester products were directly used for the subsequent reaction without further purification.

General procedure for the decarboxylative fluorosulfonylethylation

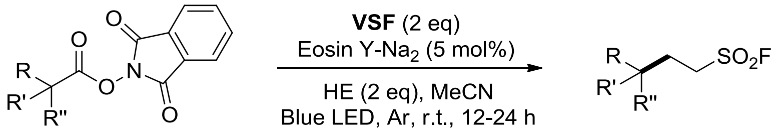

Under argon, to an oven-dried Schlenk tube (10 mL) equipped with a stir bar, was added NHPI redox-active ester (0.2 mmol, 1 equiv.), Eosin Y-Na_2 (6.8 mg, 0.01 mmol, 0.05 equiv.), and HE (101.2 mg, 0.4 mmol, 2 equiv.), followed by the addition of dry MeCN (2 mL) and VSF (32 μL, 0.4 mmol, 2 equiv.). The reaction mixture was then degassed by three freeze-pump-thaw cycles. The Schlenk tube was then backfilled with argon. The reaction mixture was stirred at room temperature for 12 to 24 hours under the irradiation of blue LED bulb (18W x 2, at approximately 2 cm away from the light sources, ca. 25 ℃). The product was purified by flash chromatography (SiO_2, PE/EA = 20:1 to 4:1) to give the corresponding pure product.

The scaled-up, one-opt procedure for the synthesis of 32

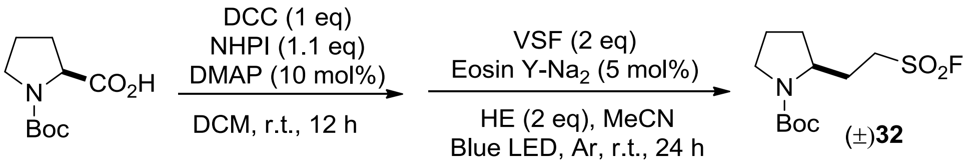

(±)**32**

To a 25-mL round-bottom flask was charged with *N*-Boc-*L*-Proline (430 mg, 2 mmol, 1.0 equiv.), *N*-hydroxyphthalimide (360 mg, 2.2 mmol, 1.1 equiv.) and DMAP (24 mg, 0.2 mmol, 0.1 equiv.). Dichloromethane was added (10 mL, 0.2 M), and the mixture was stirred vigorously. DCC (412 mg, 2 mmol, 1 equiv) was then added and the mixture allowed to stir until the acid was completely consumed (determined by TLC). The crude reaction mixture was concentrated under reduced pressure. The residue was dissolved in acetonitrile (20 mL), and transferred into a 50 mL Schlenk flask. Eosin Y-Na_2 (68 mg, 0.1 mmol, 0.05 equiv.), HE (1.0 g, 4.0 mmol, 2 equiv.), and VSF (320 μL, 4.0 mmol, 2 equiv.) were added. The reaction mixture was then degassed by three freeze-pump-thaw cycles, and backfilled with argon. The reaction mixture was stirred at room temperature for 24 h under the irradiation of blue LED bulb (18W x 2, at approximately 2 cm away from the light sources, ca. 25 ℃). Purification by flash chromatography (SiO_2, PE/EA = 6:1, R_f = 0.38) gave the desired product as a colorless oil in 85% yield.

One-opt procedure for the synthesis of four-peptide 47

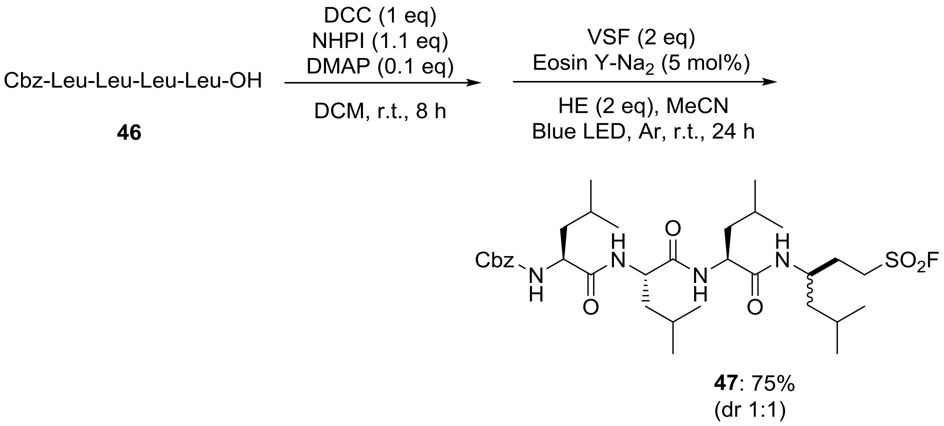

To a Schlenk tube was charged with Cbz-Leu-Leu-Leu-Leu-OH (100.0 mg, 0.165 mmol, 1.0 equiv.), N-hydroxyphthalimide (29.6 mg, 0.182 mmol, 1.1 equiv.) and DMAP (2.0 mg, 0.017 mmol, 0.1 equiv.). Dichloromethane was added (2 mL), and the mixture was stirred vigorously. DCC (34.0 mg, 0.165 mmol, 1 equiv) was then added and the mixture allowed to stir until the acid was consumed (determined by TLC). The crude reaction mixture was concentrate in vacuo. The residue was dissolved in acetonitrile (1.65 mL), and transferred to a 5 mL Schlenk tube. Eosin Y -Na_2 (5.6 mg, 0.008 mmol, 0.05 equiv.), HE (83.5 mg, 0.33 mmol, 2 equiv.), and VSF (27 μL, 0.33 mmol, 2 equiv.) were added. The reaction mixture was degassed by three freeze-pump-thaw cycles. The reaction mixture was stirred and irradiated with 18W×2 blue LED bulbs for 24 hours. The product was purified by flash chromatography (SiO_2, DCM/MeOH = 80:1, R_f = 0.15) to give the desired product as white solids. A second flash chromatography was conducted to obtain one pure diastereoisomer.

Synthesis of sultam 48 & 49

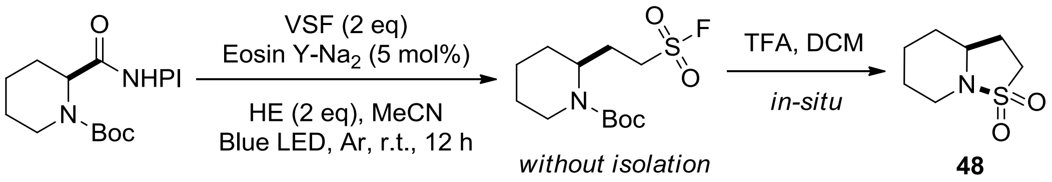

The reaction was performed on a 0.2 mmol scale, according to the general procedure, followed by deprotection with TFA. After irradiation for 12 hours, the reaction mixture was

concentrate in vacuo and diluted with dichloromethane (0.4 mL). The solution was transferred to a 5 mL glass vial and 0.2 ml of TFA was added slowly via syringe. The reaction mixture was stirred overnight at room temperature to achieve full conversion. The product was purified by flash chromatography (SiO_2, PE/EA = 2:1, R_f = 0.48) to obtain the product as a colorless oil in 84% yield.

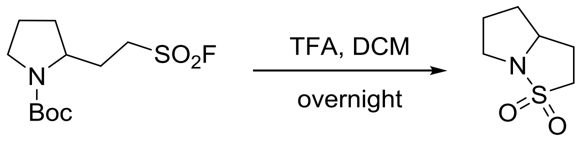

49

To a solution of the sulfonyl fluoride (28.1 mg, 0.10 mmol) in dichloromethane (0.2 mL) was added TFA (0.1 mL) slowly via syringe. The reaction mixture was stirred overnight at room temperature to achieve full conversion. The product was purified by flash chromatography (SiO_2, PE/EA = 2:1, R_f = 0.28) to obtain the product as a colorless oil in 80% yield.

Synthesis of sulfonate 50

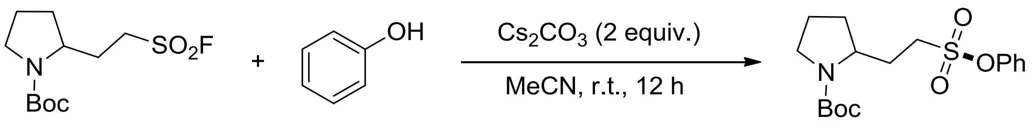

50

To a 5 mL glass vial were added sulfonyl fluoride (28.1 mg, 0.10 mmol, 1.0 equiv.), phenol (10.3 mg, 0.11 mmol, 1.1 equiv.), Cs_2CO_3 (65.2 mg, 0.20 mmol, 2.0 equiv.), followed by the addition of dry MeCN (0.5 mL). The reaction mixture was stirred at room temperature for 12 hours to achieve full conversion. The crude reaction mixture was concentrated in vacuo and purified by flash chromatography (SiO_2, PE/EA = 6:1, R_f = 0.33), giving the desired product as a colorless oil in 85% yield.

Synthesis of sulfonamide 51

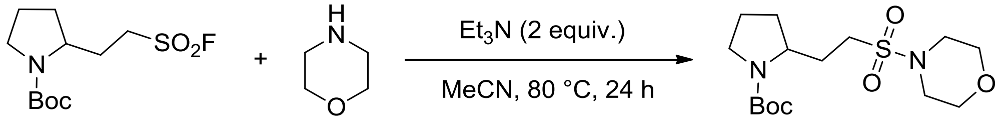

51

Sulfonyl fluoride (28.1 mg, 0.10 mmol, 1.0 equiv.) was added to a solution of morpholine (17.4 mg, 0.20 mmol, 2.0 equiv.) and triethylamine (28 μL, 0.2 mmol, 2.0 equiv.) in acetonitrile (0.1 mL). The reaction mixture was stirred at 80 ℃ for 24 hours to achieve full conversion. The crude reaction mixture was concentrated in vacuo and purified by flash chromatography (SiO_2, DCM/EA = 8:1, R_f = 0.62) to give the product as a yellow oil in 98% yield.

Synthesis of sulfonyl azide 52

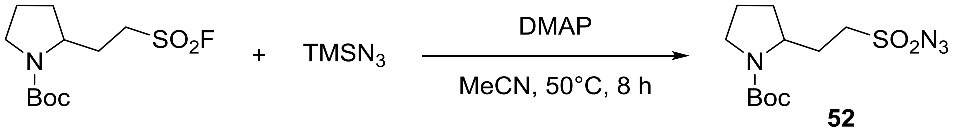

52

To a solution of sulfonyl fluoride (28.1 mg, 0.10 mmol, 1.0 equiv.) in MeCN (0.2 mL) at 50 ℃ was added DMAP (18.3 mg, 0.15 mmol, 1.5 equiv.) followed by $TMSN_3$ (10 μL, 0.075 mmol, 0.75 equiv.). The solution was stirred at 50 ℃ for 15 min, then further two portions of $TMSN_3$ (10 μL, 0.075 mmol, 0.75 equiv.) were added at intervals of 15 min. The solution was stirred for 6 hours to achieve full conversion. The crude reaction mixture was concentrated in vacuo and purified by flash chromatography (SiO_2, PE/EA = 6:1, R_f = 0.33) to give the product as a colorless oil in 82% yield.

Synthesis of triazole 53

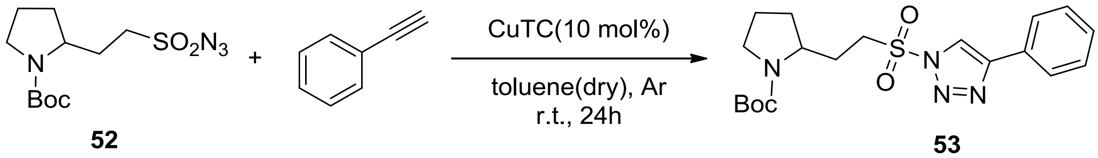

To a dry toluene (0.4 mL) suspension of CuTC (1.9 mg, 0.01 mmol, 0.10 equiv.), was added alkyne (11 μL, 0.10 mmol, 1.0 equiv.) with vigorous stirring. After 10 min, a toluene (0.1 mL) solution of **52** (30.9 mg, 0.11 mmol, 1.1 equiv.) was added dropwise over 15 min. The reaction was stirred at room temperature until complete consumption of the alkyne by TLC (24 hours). The crude reaction mixture was concentrate in vacuo and purified by flash chromatography (SiO_2, DCM, $R_f = 0.44$) to afford the pure triazole product as white solids in 85% yield.

Characterizations of redox-active esters and products

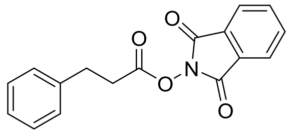

1[3]

^{1}H NMR (400 MHz, $CDCl_3$): δ 7.92 – 7.87 (m, 2H), 7.82 – 7.77 (m, 2H), 7.34 (t, *J* = 7.5 Hz, 2H), 7.27 – 7.24 (m, 3H), 3.11 (t, *J* = 7.7, 2H), 2.99 (t, *J* = 7.8, 2H);

^{13}C NMR (100 MHz, $CDCl_3$): δ 168.8, 161.8, 139.1, 134.7, 128.8, 128.7, 128.2, 126.7, 123.9, 32.7, 30.5.

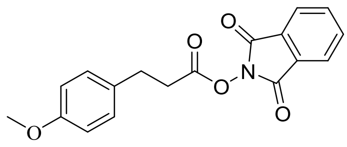

4s

^{1}H NMR (400 MHz, $CDCl_3$): δ 7.91 – 7.87 (m, 2H), 7.81 – 7.77 (m, 2H), 7.18 (d, *J* = 7.3 Hz, 2H), 6.87 (d, *J* = 7.2 Hz, 2H), 3.80 (s, 3H), 3.05 (t, *J* = 7.6 Hz, 2H), 2.94 (t, *J* = 7.6 Hz, 2H);

^{13}C NMR (100 MHz, $CDCl_3$): δ 168.8, 161.8, 158.3, 134.7, 131.2, 129.2, 128.8, 123.9, 114.0, 55.2, 32.9, 29.7;

HRMS (ESI+): $[M+Na]^+$ Calc. for $C_{18}H_{15}NNaO_5$: 348.0842; found: 348.0842.

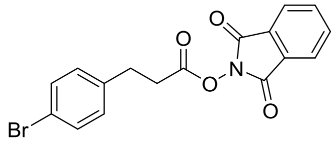

5s[4]

^{1}H NMR (400 MHz, $CDCl_3$): δ 7.90 – 7.85 (m, 2H), 7.81 – 7.76 (m, 2H), 7.44 (d, *J* = 8.0 Hz, 2H), 7.14 (d, *J* = 8.0 Hz, 2H), 3.05 (t, *J* = 7.5 Hz, 2H), 2.96 (t, *J* = 7.7 Hz, 2H);

^{13}C NMR (100 MHz, $CDCl_3$): δ 168.6, 161.8, 138.0, 134.8, 131.7, 130.0, 128.8, 123.9, 120.5, 32.4, 29.9.

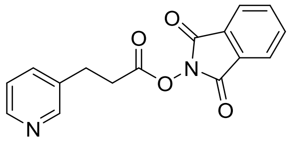

6s[4]

^{1}H NMR (400 MHz, $CDCl_3$): δ 8.54 – 8.50 (m, 2H), 7.90 – 7.86 (m, 2H), 7.81 – 7.78 (m, 2H), 7.61 (d, *J* = 7.7 Hz, 1H), 7.28 (s, 1H), 3.11 (t, *J* = 7.3 Hz, 2H), 3.00 (t, *J* = 7.4 Hz, 2H);
^{13}C NMR (100 MHz, $CDCl_3$): δ 168.3, 161.6, 148.7, 147.1, 137.0, 134.8, 133.8, 129.4, 123.9, 122.9, 32.0, 27.6.

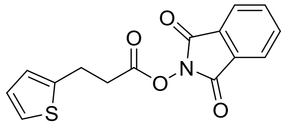

7s[5]

^{1}H NMR (400 MHz, $CDCl_3$): δ 7.90 – 7.86 (m, 2H), 7.81 – 7.77 (m, 2H), 7.19 – 7.16 (m, 1H), 6.99 – 6.88 (m, 2H), 3.30 (t, *J* = 7.4 Hz, 2H), 3.06 (t, *J* = 7.6 Hz, 2H);
^{13}C NMR (100 MHz, $CDCl_3$): δ 168.4, 161.7, 141.3, 134.7, 128.7, 127.0, 125.1, 123.9, 123.9, 32.9, 24.7.

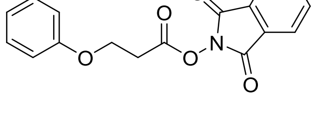

8s

^{1}H NMR (400 MHz, $CDCl_3$): δ 7.91 – 7.89 (m, 2H), 7.81 – 7.78 (m, 2H), 7.30 (t, *J* = 7.2 Hz, 2H), 7.00 – 6.95 (m, 3H), 4.38 (t, *J* = 6.2 Hz, 2H), 3.17 (t, *J* = 6.2 Hz, 2H);
^{13}C NMR (100 MHz, $CDCl_3$): δ 167.2, 161.7, 158.1, 134.8, 129.5, 128.8, 124.0, 121.4, 114.8, 62.5, 31.7;

HRMS (ESI+): $[M+Na]^+$ Calc. for $C_{17}H_{13}NNaO_5$: 334.0686; found: 334.0686.

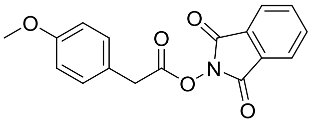

9s

^{1}H NMR (400 MHz, $CDCl_3$): δ 7.89 – 7.85 (m, 2H), 7.81 – 7.77 (m, 2H), 7.30 (d, *J* = 7.6 Hz, 2H), 6.91 (d, *J* = 7.5 Hz, 2H), 3.93 (s, 2H), 3.81 (s, 3H);

^{13}C NMR (100 MHz, $CDCl_3$): δ 167.9, 161.8, 159.1, 134.7, 130.3, 128.8, 123.9, 123.5, 114.2, 55.2, 36.8;

HRMS (ESI+): $[M+Na]^+$ Calc. for $C_{17}H_{13}NNaO_5$: 334.0686; found: 334.0686.

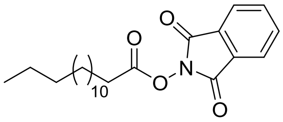

10s

^{1}H NMR (400 MHz, $CDCl_3$): δ 7.89 – 7.86 (m, 2H), 7.80 – 7.77 (m, 2H), 2.66 (t, *J* = 7.5 Hz, 2H), 1.82 – 1.74 (m, 2H), 1.47 – 1.26 (m, 22H), 0.87 (t, *J* = 6.5 Hz, 3H);

^{13}C NMR (100 MHz, $CDCl_3$): δ 169.6, 162.0, 134.7, 128.9, 123.9, 31.9, 31.0, 29.66, 29.63, 29.62, 29.60, 29.53, 29.34, 29.33, 29.1, 28.8, 24.7, 22.7, 14.1;

HRMS (ESI+): $[M+Na]^+$ Calc. for $C_{23}H_{33}NNaO_4$: 410.2302; found: 410.2306.

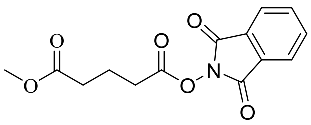

11s[6]

^{1}H NMR (400 MHz, $CDCl_3$): δ 7.88 – 7.84 (m, 2H), 7.79 – 7.75 (m, 2H), 3.68 (s, 3H), 2.75 (t, *J* = 6.8 Hz, 2H), 2.49 (t, *J* = 6.9 Hz, 2H), 2.12 – 2.05 (m, 2H);

^{13}C NMR (100 MHz, $CDCl_3$): δ 172.9, 169.0, 161.8, 134.7, 128.8, 123.9, 51.7, 32.4, 30.0, 19.8.

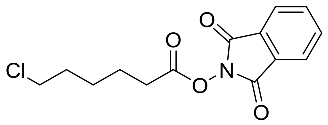

12s

^{1}H NMR (400 MHz, $CDCl_3$): δ 7.90 – 7.87 (m, 2H), 7.82 – 7.77 (m, 2H), 3.57 (t, *J* = 6.4 Hz, 2H), 2.69 (t, *J* = 7.1 Hz, 2H), 1.88 – 1.79 (m, 4H), 1.65 – 1.59 (m, 2H);

^{13}C NMR (100 MHz, $CDCl_3$): δ 169.3, 161.9, 134.7, 128.9, 123.9, 44.5, 32.0, 30.8, 26.1, 24.0;

HRMS (ESI+): $[M+Na]^+$ Calc. for $C_{14}H_{14}ClNNaO_4$: 318.0504; found: 318.0499.

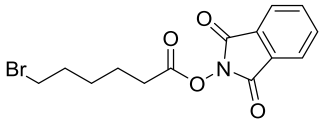

13s[5]

^{1}H NMR (400 MHz, $CDCl_3$): δ 7.90 – 7.87 (m, 2H), 7.81 – 7.77 (m, 2H), 3.43 (t, *J* = 7.3, 2H), 2.69 (t, *J* = 7.7, 2H), 1.97 – 1.89 (m, 2H), 1.87 – 1.79 (m, 2H), 1.65 – 1.60 (m, 2H);

^{13}C NMR (100 MHz, $CDCl_3$): δ 169.2, 161.8, 134.7, 128.8, 123.9, 33.1, 32.1, 30.7, 27.2, 23.8.

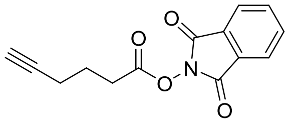

14s[4]

^{1}H NMR (400 MHz, $CDCl_3$): δ 7.91 – 7.86 (m, 2H), 7.82 – 7.77 (m, 2H), 2.84 (t, *J* = 7.4 Hz, 2H), 2.40 – 2.37 (m, 2H), 2.03 – 1.98 (m, 3H);

^{13}C NMR (100 MHz, $CDCl_3$): δ 169.0, 161.8, 134.7, 128.7, 123.9, 82.4, 69.8, 29.6, 23.3, 17.5.

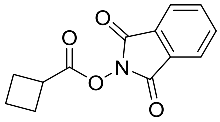

15s[2]

^{1}H NMR (400 MHz, $CDCl_3$): δ 7.90 – 7.85 (m, 2H), 7.80 – 7.76 (m, 2H), 3.55 – 3.46 (m, 1H), 2.55 – 2.36 (m, 4H), 2.16 – 2.00 (m, 2H);

^{13}C NMR (100 MHz, $CDCl_3$): δ 171.4, 162.0, 134.7, 128.9, 123.9, 35.0, 25.3, 18.7.

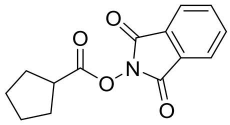

16s[2]

^{1}H NMR (400 MHz, $CDCl_3$): δ 7.86 – 7.83 (m, 2H), 7.77 – 7.74 (m, 2H), 3.13 – 3.05 (m, 1H), 2.10 – 1.96 (m, 4H), 1.80 – 1.71 (m, 2H), 1.68 – 1.60 (m, 2H);

^{13}C NMR (100 MHz, $CDCl_3$): δ 172.8, 162.0, 134.6, 128.9, 123.8, 40.6, 30.2, 25.9.

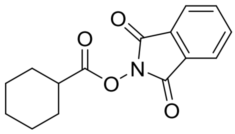

17s[7]

^{1}H NMR (400 MHz, $CDCl_3$): δ 7.90 – 7.85 (m, 2H), 7.80 – 7.75 (m, 2H), 2.77 – 2.70 (m, 1H), 2.13 – 2.06 (m, 2H), 1.87 – 1.80 (m, 2H), 1.70 – 1.61 (m, 3H), 1.43 – 1.29 (m, 3H);

^{13}C NMR (100 MHz, $CDCl_3$): δ 171.7, 161.9, 134.6, 128.9, 123.7, 40.3, 28.7, 25.4, 24.9.

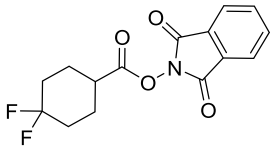

18s[4]

^{1}H NMR (400 MHz, $CDCl_3$): δ 7.91 – 7.87 (m, 2H), 7.82 – 7.77 (m, 2H), 2.92 – 2.84 (m, 1H), 2.25 – 2.03 (m, 6H), 1.97 – 1.84 (m, 2H);

^{13}C NMR (100 MHz, $CDCl_3$): δ 170.4, 161.8, 134.8, 128.7, 123.9, 122.1 (t, *J* = 240.0 Hz), 37.7, 31.9 (t, *J* = 24.6 Hz), 24.8 (t, *J* = 5.2 Hz);

^{19}F NMR (376 MHz, $CDCl_3$): δ -95.7 (d, *J* = 246.7 Hz), -98.5 (d, *J* = 235.8 Hz).

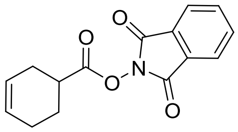

19s[7]

^{1}H NMR (400 MHz, $CDCl_3$): δ 7.91 – 7.87 (m, 2H), 7.81 – 7.76 (m, 2H), 5.79 – 5.68 (m, 2H), 3.02 – 2.96 (m, 1H), 2.47 – 2.43 (m, 2H), 2.26 – 2.13 (m, 3H), 1.95 – 1.85 (m, 1H);

^{13}C NMR (100 MHz, $CDCl_3$): δ 171.7, 162.0, 134.7, 128.9, 126.8, 124.3, 123.9, 36.9, 27.1, 24.9, 23.9.

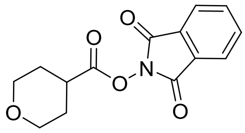

20s[7]

^{1}H NMR (400 MHz, $CDCl_3$): δ 7.91 – 7.87 (m, 2H), 7.82 – 7.78 (m, 2H), 4.04 – 4.00 (m, 2H), 3.56 – 3.50 (m, 2H), 3.03 – 2.97 (m, 1H), 2.07 – 1.93 (m, 4H);

^{13}C NMR (100 MHz, $CDCl_3$): δ 170.5, 161.9, 134.8, 128.8, 123.9, 66.5, 37.6, 28.3.

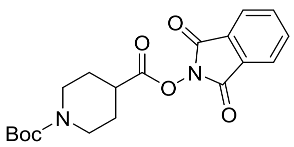

21s[5]

^{1}H NMR (400 MHz, $CDCl_3$): δ 7.90 – 7.86 (m, 2H), 7.81 – 7.77 (m, 2H), 4.08 – 3.97 (m, 2H), 3.03 – 2.97 (m, 2H), 2.93 – 2.87 (m, 1H), 2.09 – 2.02 (m, 2H), 1.89 – 1.80 (m, 2H), 1.46 (s, 9H);

^{13}C NMR (100 MHz, $CDCl_3$): δ 170.5, 161.8, 154.4, 134.7, 128.8, 123.9, 79.7, 42.5, 38.4, 28.3, 27.6.

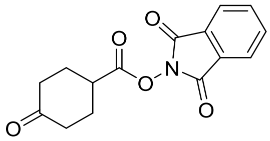

22s[8]

^{1}H NMR (400 MHz, $CDCl_3$): δ 7.90 – 7.85 (m, 2H), 7.81 – 7.77 (m, 2H), 3.22 – 3.14 (m, 1H), 2.64 – 2.55 (m, 2H), 2.47 – 2.21 (m, 6H);

^{13}C NMR (100 MHz, $CDCl_3$): δ 208.8, 170.4, 161.9, 134.9, 128.9, 124.0, 39.1, 38.0, 28.2.

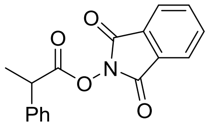

23s[4]

^{1}H NMR (400 MHz, $CDCl_3$): δ 7.88 – 7.84 (m, 2H), 7.79 – 7.75 (m, 2H), 7.43 – 7.32 (m, 5H), 4.13 (q, *J* = 7.1 Hz, 1H), 1.68 (d, *J* = 7.1 Hz, 3H);

^{13}C NMR (100 MHz, $CDCl_3$): δ 170.7, 161.7, 138.2, 134.6, 128.8, 128.7, 127.7, 127.5, 123.8, 42.8, 18.9.

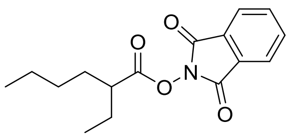

24s[2]

^{1}H NMR (400 MHz, $CDCl_3$): δ 7.91 – 7.86 (m, 2H), 7.81 – 7.76 (m, 2H), 2.69 – 2.62 (m, 1H), 1.86 – 1.59 (m, 4H), 1.50 – 1.31 (m, 4H), 1.08 (t, *J* = 7.2 Hz, 3H), 0.94 (t, *J* = 7.0 Hz, 3H);
^{13}C NMR (100 MHz, $CDCl_3$): δ 172.4, 162.0, 134.6, 129.0, 123.8, 44.8, 31.7, 29.2, 25.6, 22.5, 13.8, 11.5.

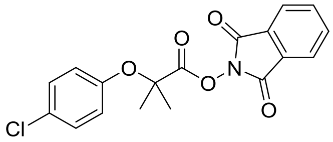

25s[9]

^{1}H NMR (400 MHz, $CDCl_3$): δ 7.92 – 7.88 (m, 2H), 7.83 – 7.79 (m, 2H), 7.29 (d, *J* = 7.6 Hz, 2H), 7.04 (d, *J* = 7.3 Hz, 2H), 1.77 (s, 6H);
^{13}C NMR (100 MHz, $CDCl_3$): δ 170.5, 161.7, 153.1, 134.9, 129.3, 128.8, 128.4, 124.0, 121.6, 78.9, 25.5.

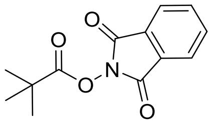

26s[10]

^{1}H NMR (400 MHz, $CDCl_3$): δ 7.91 – 7.86 (m, 2H), 7.80 – 7.76 (m, 2H), 1.44 (s, 9H);
^{13}C NMR (100 MHz, $CDCl_3$): δ 174.3, 162.0, 134.6, 129.0, 123.8, 38.4, 27.0.

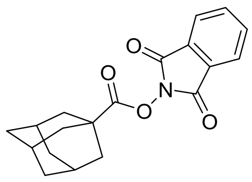

27s[5]

^{1}H NMR (400 MHz, $CDCl_3$): δ 7.89 – 7.87 (m, 2H), 7.79 – 7.71 (m, 2H), 2.14 (s, 6H), 2.11 (s, 3H), 1.81 (s, 6H).

^{13}C NMR (100 MHz, $CDCl_3$): δ 173.2, 162.1, 134.6, 129.0, 123.8, 40.5, 38.4, 36.2, 27.6.

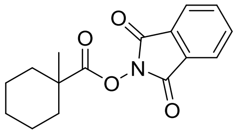

28s[11]

^{1}H NMR (400 MHz, $CDCl_3$): δ 7.90 – 7.86 (m, 2H), 7.80 – 7.76 (m, 2H), 2.26 – 2.22 (m, 2H), 1.70 – 1.52 (m, 6H), 1.43 (s, 3H), 1.41 – 1.33 (m, 2H);

^{13}C NMR (100 MHz, $CDCl_3$): δ 173.6, 162.2, 134.6, 129.0, 123.8, 43.1, 35.7, 26.7, 25.4, 23.0.

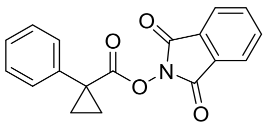

29s[4]

^{1}H NMR (400 MHz, $CDCl_3$): δ 7.87 – 7.83 (m, 2H), 7.78 – 7.74 (m, 2H), 7.52 (d, *J* = 7.2 Hz, 2H), 7.38 – 7.29 (m, 3H), 1.93 – 1.89 (m, 2H), 1.51 – 1.47 (m, 2H);

^{13}C NMR (100 MHz, $CDCl_3$): δ 171.0, 161.8, 136.9, 134.6, 130.5, 128.9, 128.4, 127.9, 123.8, 27.3, 18.6.

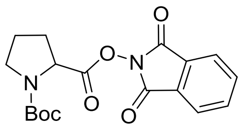

32s[10]

^{1}H NMR (400 MHz, $CDCl_3$): δ 7.90 – 7.87 (m, 2H), 7.81 – 7.77 (m, 2H), 4.72 – 4.59 (m, 1H), 3.66 – 3.41 (m, 2H), 2.47 – 2.32 (m, 2H), 2.13 – 1.94 (m, 2H), 1.54 – 1.46 (m, 9H);

^{13}C NMR (100 MHz, $CDCl_3$): δ 169.6, 161.6, 153.4, 134.7, 128.8, 123.9, 81.0, 57.1, 46.2, 31.3, 28.0, 23.5.

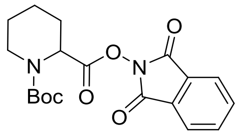

33s[5]

^{1}H NMR (400 MHz, $CDCl_3$): δ 7.90 – 7.87 (m, 2H), 7.81 – 7.77 (m, 2H), 5.37 – 5.13 (m, 1H), 4.08 – 3.94 (m, 1H), 3.07 – 3.00 (m, 1H), 2.36 (d, J = 13.7 Hz, 1H), 1.86 – 1.74 (m, 3H), 1.55 – 1.42 (m, 11H);

^{13}C NMR (100 MHz, $CDCl_3$): δ 168.7, 161.6, 155.0, 134.7, 128.8, 123.9, 80.9, 53.5, 41.1, 28.0, 27.1, 24.3, 20.2.

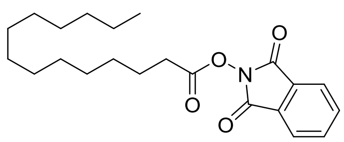

38s[10]

^{1}H NMR (400 MHz, $CDCl_3$): δ 7.91 – 7.86 (m, 2H), 7.81 – 7.77 (m, 2H), 2.66 (t, J = 6.9 Hz, 2H), 1.80 – 1.72 (m, 2H), 1.48 – 1.20 (m, 20H), 0.87 (t, J = 7.0 Hz, 3H);

^{13}C NMR (100 MHz, $CDCl_3$): δ 169.6, 161.9, 134.7, 128.9, 123.9, 31.9, 31.0, 29.63, 29.61, 29.59, 29.53, 29.34, 29.32, 29.1, 28.8, 24.6, 22.7, 14.1.

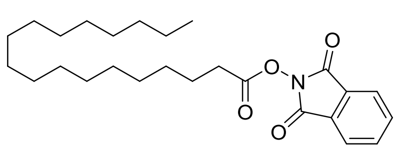

39s[4]

^{1}H NMR (400 MHz, $CDCl_3$): δ 7.91 – 7.86 (m, 2H), 7.81 – 7.77 (m, 2H), 2.66 (t, *J* = 7.3 Hz, 2H), 1.82 – 1.74 (m, 2H), 1.47 – 1.23 (m, 28H), 0.87 (t, *J* = 6.3 Hz, 3H);

^{13}C NMR (100 MHz, $CDCl_3$): δ 169.6, 162.0, 134.7, 129.0, 123.9, 31.9, 31.0, 29.68(5C), 29.65(2C), 29.62, 29.55, 29.4, 29.1, 28.8, 24.7, 22.7, 14.1.

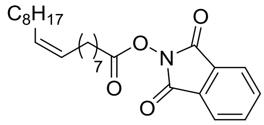

40s[4]

^{1}H NMR (400 MHz, $CDCl_3$): δ 7.90 – 7.86 (m, 2H), 7.80 – 7.76 (m, 2H), 5.39 – 5.31 (m, 2H), 2.66 (t, *J* = 7.5 Hz, 2H), 2.06 – 1.97 (m, 4H), 1.78 (p, *J* = 7.5 Hz, 2H), 1.47 – 1.23 (m, 20H), 0.87 (t, *J* = 7.0 Hz, 3H).

^{13}C NMR (100 MHz, $CDCl_3$): δ 169.6, 162.0, 134.7, 130.0, 129.7, 128.9, 123.9, 31.9, 31.0, 29.8, 29.6, 29.5, 29.3 (2C), 29.0 (2C), 28.8, 27.2, 27.1, 24.6, 22.7, 14.1.

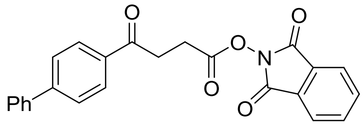

41s[4]

^{1}H NMR (400 MHz, $CDCl_3$): δ 8.08 (d, *J* = 7.2 Hz, 2H), 7.91 – 7.87 (m, 2H), 7.81 – 7.77 (m, 2H), 7.70 (d, *J* = 7.2 Hz, 2H), 7.63 (d, *J* = 7.1 Hz, 2H), 7.49 – 7.38 (m, 3H), 3.50 (t, *J* = 6.2 Hz, 2H), 3.18 (t, *J* = 6.2 Hz, 2H);

^{13}C NMR (100 MHz, $CDCl_3$): δ 196.0, 169.3, 161.8, 146.1, 139.7, 134.72, 134.67, 128.92, 128.85, 128.7, 128.3, 127.3, 127.2, 123.9, 33.2, 25.4.

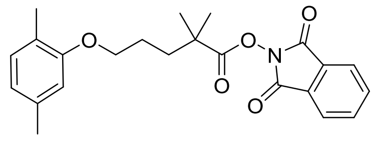

42s[12]

¹H NMR (400 MHz, $CDCl_3$): δ 7.91 – 7.86 (m, 2H), 7.81 – 7.77 (m, 2H), 7.00 (d, *J* = 6.7 Hz, 1H), 6.67 – 6.62 (m, 2H), 4.02 – 4.00 (m, 2H), 2.31 (s, 3H), 2.19 (s, 3H), 1.99 – 1.92 (m, 4H), 1.45 (s, 6H);

¹³C NMR (100 MHz, $CDCl_3$): δ 173.7, 162.0, 156.9, 136.4, 134.6, 130.2, 129.0, 123.8, 123.5, 120.6, 111.9, 67.6, 41.9, 37.3, 25.1, 24.9, 21.3, 15.7.

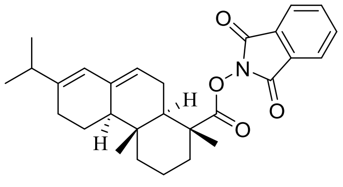

43s[4]

¹H NMR (400 MHz, $CDCl_3$): δ 7.89 – 7.84 (m, 2H), 7.80 – 7.75 (m, 2H), 5.80 (s, 1H), 5.46 (s, 1H), 2.28 – 1.81 (m, 10H), 1.84 – 1.81 (m, 1H), 1.70 – 1.64 (m, 2H), 1.45 (s, 3H), 1.25 – 1.19 (m, 2H), 1.03 – 0.98 (m, 6H), 0.88 (s, 3H);

¹³C NMR (100 MHz, $CDCl_3$): δ 174.5, 162.1, 145.1, 135.2, 134.6, 129.0, 123.8, 122.4, 120.5, 50.8, 46.6, 45.1, 38.0, 37.2, 34.8, 34.6, 27.4, 25.5, 22.4, 21.4, 20.8, 17.8, 16.9, 14.1.

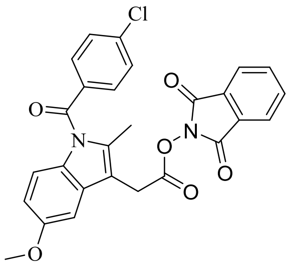

44s[4]

¹H NMR (400 MHz, $CDCl_3$): δ 7.90 – 7.86 (m, 2H), 7.81 – 7.76 (m, 2H), 7.69 (d, *J* = 7.5 Hz, 2H), 7.48 (d, *J* = 7.4 Hz, 2H), 7.03 (s, 1H), 6.93 (d, *J* = 8.9 Hz, 1H), 6.70 (d, *J* = 8.9 Hz, 1H), 4.04 (s, 2H), 3.89 (s, 3H), 2.42 (s, 3H);

¹³C NMR (100 MHz, $CDCl_3$): δ 168.2, 167.0, 161.7, 156.2, 139.4, 136.4, 134.8, 133.6, 131.2, 130.7, 129.9, 129.1, 128.8, 123.9, 115.0, 112.4, 110.1, 100.6, 55.7, 27.1, 13.4.

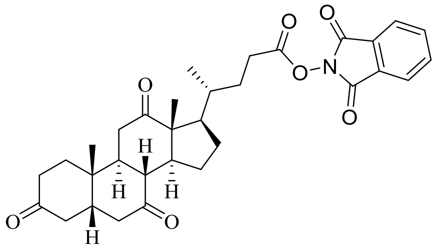

45s[9]

^{1}H NMR (400 MHz, $CDCl_3$): δ 7.91 – 7.85 (m, 2H), 7.82 – 7.77 (m, 2H), 2.93 – 2.82 (m, 3H), 2.79 – 2.71 (m, 1H), 2.67 – 2.60 (m, 1H), 2.37 – 2.20 (m, 6H), 2.16 – 2.13 (m, 2H), 2.08 – 1.95 (m, 5H), 1.90 – 1.83 (m, 1H), 1.59 – 1.50 (m, 4H), 1.40 (s, 3H), 1.31 – 1.26 (m, 1H), 1.11 (s, 3H), 0.92 (d, *J* = 6.1 Hz, 3H);

^{13}C NMR (100 MHz, $CDCl_3$): **^{13}C NMR** (100 MHz, $CDCl_3$): δ 211.8, 209.0, 208.6, 169.8, 161.9, 134.7, 128.8, 123.8, 56.8, 51.7, 48.9, 46.7, 45.5, 45.4, 44.9, 42.7, 38.5, 36.4, 35.9, 35.2 (2C), 30.2, 28.4, 27.5, 25.0, 21.8, 18.4, 11.8.

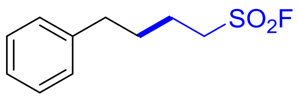

3

Compound **3**: 85% yield, colorless oil.

^{1}H NMR (400 MHz, $CDCl_3$): δ 7.32 (t, *J* = 7.4 Hz, 2H), 7.25 - 7.18 (m, 3H), 3.39 - 3.34 (m, 2H), 2.70 (t, *J* = 7.4 Hz, 2H), 2.03 - 1.95 (m, 2H), 1.87 - 1.79 (m, 2H);

^{13}C NMR (100 MHz, $CDCl_3$): δ 140.7, 128.5, 128.3, 126.2, 50.7 (d, *J* = 16.1 Hz), 35.0, 29.4, 22.9;

^{19}F NMR (376 MHz, $CDCl_3$): δ 53.6;

HRMS (ESI+): $[M+Na]^+$ Calc. for $C_{10}H_{13}FNaO_2S$: 239.0512; found: 239.0512.

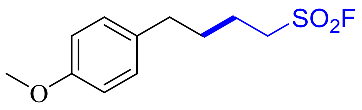

4

Compound **4**: 89% yield, colorless oil.

^{1}H NMR (400 MHz, $CDCl_3$): δ 7.08 (d, *J* = 7.5 Hz, 2H), 6.85 (d, *J* = 7.1 Hz, 2H), 3.79 (s, 3H), 3.38 - 3.33 (m, 2H), 2.63 (t, *J* = 7.4 Hz, 2H), 2.00 - 1.93 (m, 2H), 1.82 - 1.75 (m, 2H);

^{13}C NMR (100 MHz, $CDCl_3$): δ 158.1, 132.7, 129.2, 114.0, 55.3, 50.8 (d, *J* = 15.9 Hz), 34.1, 29.7, 22.9;

^{19}F NMR (376 MHz, $CDCl_3$): δ 53.6;

HRMS (ESI+): $[M+Na]^+$ Calc. for $C_{11}H_{15}FNaO_3S$: 269.0618; found: 269.0623.

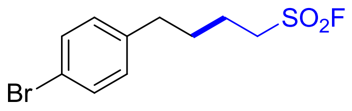

5

Compound **5**: 82% yield, colorless oil.

^{1}H NMR (400 MHz, $CDCl_3$): δ 7.42 (d, *J* = 7.9 Hz, 2H), 7.04 (d, *J* = 7.7 Hz, 2H), 3.39 – 3.34 (m, 2H), 2.64 (t, *J* = 7.4 Hz, 2H), 2.00 – 1.93 (m, 2H), 1.83 – 1.76 (m, 2H);

^{13}C NMR (100 MHz, $CDCl_3$): δ 139.6, 131.6, 130.0, 120.0, 50.7 (d, J = 16.4 Hz), 34.5, 29.3, 22.9;

^{19}F NMR (376 MHz, $CDCl_3$): δ 53.8;

HRMS (ESI+): $[M+Na]^+$ Calc. for $C_{10}H_{12}BrFNaO_2S$: 316.9618; found: 316.9628.

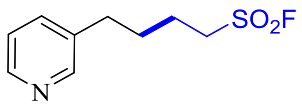

6

Compound **6**: 81% yield, colorless oil.

^{1}H NMR (400 MHz, $CDCl_3$): δ 8.48 – 8.45 (m, 2H), 7.49 (d, J = 7.6 Hz, 1H), 7.24 (t, J = 6.9 Hz, 1H), 3.41 – 3.37 (m, 2H), 2.69 (t, J = 7.6 Hz, 2H), 2.04 – 1.96 (m, 2H), 1.87 – 1.80 (m, 2H);

^{13}C NMR (100 MHz, $CDCl_3$): δ 149.7, 147.9, 135.9, 135.7, 123.5, 50.6 (d, J = 16.7 Hz), 32.2, 29.2, 23.0;

^{19}F NMR (376 MHz, $CDCl_3$): δ 54.0;

HRMS (ESI+): $[M+Na]^+$ Calc. for $C_9H_{12}FNNaO_2S$: 240.0465; found: 240.0469.

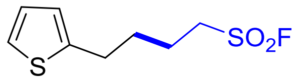

7

Compound **7**: 27% yield, colorless oil.

^{1}H NMR (400 MHz, $CDCl_3$): δ 7.15 (d, J = 4.8 Hz, 1H), 6.93 (s, 1H), 6.81 (s, 1H), 3.39 – 3.35 (m, 2H), 2.91 (t, J = 7.2 Hz, 2H), 2.06 – 1.98 (m, 2H), 1.91 – 1.84 (m, 2H);

^{13}C NMR (100 MHz, $CDCl_3$): δ 143.2, 126.9, 124.7, 123.5, 50.6 (d, J = 16.5 Hz), 29.8, 29.1, 22.8;

^{19}F NMR (376 MHz, $CDCl_3$): δ 53.2;

HRMS (ESI+): $[M+Na]^+$ Calc. for $C_8H_{11}FNaO_2S_2$: 245.0077; found: 245.0081.

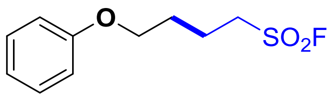

8

Compound **8**: 60% yield, colorless oil.

^{1}H NMR (400 MHz, $CDCl_3$): δ 7.30 (t, *J* = 7.2 Hz, 2H), 6.97 (t, *J* = 7.3 Hz, 1H), 6.89 (d, *J* = 7.8 Hz, 2H), 4.03 (t, *J* = 5.3 Hz, 2H), 3.52 – 3.48 (m, 2H), 2.23 – 2.16 (m, 2H), 2.03 – 1.96 (m, 2H);

^{13}C NMR (100 MHz, $CDCl_3$): δ 158.5, 129.6, 121.1, 114.4, 66.5, 50.7 (d, *J* = 16.5 Hz), 27.4, 20.9;

^{19}F NMR (376 MHz, $CDCl_3$): δ 53.4;

HRMS (ESI+): $[M+Na]^+$ Calc. for $C_{10}H_{13}FNaO_3S$: 255.0462; found: 255.0466.

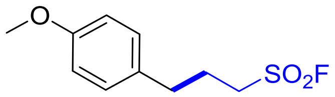

9

Compound **9**: 83% yield, colorless oil.

^{1}H NMR (400 MHz, $CDCl_3$): δ 7.10 (d, *J* = 7.6 Hz, 2H), 6.86 (d, *J* = 7.2 Hz, 2H), 3.80 (s, 3H), 3.33 – 3.29 (m, 2H), 2.76 (t, *J* = 7.2 Hz, 2H), 2.28 – 2.21 (m, 2H);

^{13}C NMR (100 MHz, $CDCl_3$): δ 158.5, 130.8, 129.4, 114.2, 55.3, 49.9 (d, *J* = 16.5 Hz), 32.6, 25.1;

^{19}F NMR (376 MHz, $CDCl_3$): δ 53.8;

HRMS (EI+): m/z Calc. for $C_{10}H_{13}FO_3S$: 232.0569; found: 232.0572.

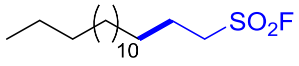

10

Compound **10**: 86% yield, white solid.

^{1}**H NMR** (400 MHz, $CDCl_3$): δ 3.37 – 3.33 (m, 2H), δ 1.98 – 1.91 (m, 2H), δ 1.51 – 1.44 (m, 2H), δ 1.35 – 1.26 (m, 24H), 0.88 (t, *J* = 6.2 Hz, 3H);

^{13}**C NMR** (100 MHz, $CDCl_3$): δ 50.9 (d, *J* = 16.0 Hz), 31.9, 29.68, 29.66, 29.64 (2C), 29.60, 29.5, 29.42, 29.35, 29.1, 28.8, 27.9, 23.4, 22.7, 14.1;

^{19}**F NMR** (376 MHz, $CDCl_3$): δ 53.3;

HRMS (EI+): m/z Calc. for $C_{16}H_{33}FO_2S$: 308.2185; found: 308.2188.

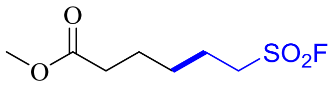

11

Compound **11**: 85% yield, colorless oil.

^{1}**H NMR** (400 MHz, $CDCl_3$): δ 3.66 (s, 3H), 3.39 - 3.34 (m, 2H), 2.34 (t, *J* = 7.2 Hz, 2H), 2.00 – 1.92 (m, 2H), 1.72 - 1.64 (m, 2H), 1.56 – 1.48 (m, 2H);

^{13}**C NMR** (100 MHz, $CDCl_3$): δ 173.5, 51.6, 50.6 (d, *J* = 16.3 Hz), 33.3, 27.2, 24.0, 23.1;

^{19}**F NMR** (376 MHz, $CDCl_3$): δ 53.5;

HRMS (ESI+): $[M+Na]^+$ Calc. for $C_7H_{13}FNaO_4S$: 235.0411; found: 235.0414.

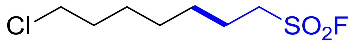

12

Compound **12**: 98% yield, colorless oil.

^{1}**H NMR** (400 MHz, $CDCl_3$): δ 3.53 (t, *J* = 6.4 Hz, 2H), 3.38 – 3.34 (m, 2H), 2.00 - 1.92 (m, 2H), 1.81 - 1.74 (m, 2H), 1.55 - 1.44 (m, 4H), 1.42 – 1.35 (m, 2H);

^{13}**C NMR** (100 MHz, $CDCl_3$): δ 50.8 (d, *J* = 16.1 Hz), 44.8, 32.3, 28.1, 27.7, 26.4, 23.3;

^{19}**F NMR** (376 MHz, $CDCl_3$): δ 53.6;

HRMS (ESI+): $[M+Na]^+$ Calc. for $C_7H_{14}ClFNaO_2S$: 239.0279; found: 239.0282.

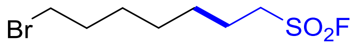

13

Compound **13**: 98% yield, colorless oil.

^{1}H NMR (400 MHz, $CDCl_3$): δ 3.40 (t, *J* = 7.1 Hz, 2H), 3.38 – 3.34 (m, 2H), 2.00 - 1.92 (m, 2H), 1.90 - 1.83 (m, 2H), 1.55 - 1.44 (m, 4H), 1.42 – 1.35 (m, 2H);

^{13}C NMR (100 MHz, $CDCl_3$): δ 50.8 (d, *J* = 16.1 Hz), 33.6, 32.4, 28.0, 27.6 (2C), 23.3;

^{19}F NMR (376 MHz, $CDCl_3$): δ 53.5;

HRMS (ESI+): $[M+Na]^+$ Calc. for $C_7H_{14}BrFNaO_2S$: 282.9774; found: 282.9781.

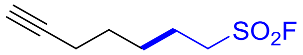

14

Compound **14**: 53% yield, colorless oil.

^{1}H NMR (400 MHz, $CDCl_3$): δ 3.40 – 3.35 (m, 2H), 2.23 (t, *J* = 6.0 Hz, 2H), 2.01 – 1.94 (m, 3H), 1.66 – 1.58 (m, 4H);

^{13}C NMR (100 MHz, $CDCl_3$): δ 83.4, 69.0, 50.7 (d, *J* = 16.5 Hz), 27.5, 26.8, 23.0, 18.0;

^{19}F NMR (376 MHz, $CDCl_3$): δ 53.0;

HRMS (EI+): $[M-C_3H_3]^+$ Calc. for $C_4H_8FO_2S$: 139.0229; found: 139.0228.

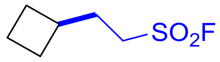

15

Compound **15**: 74% yield, colorless oil.

^{1}H NMR (400 MHz, $CDCl_3$): δ 3.27 – 3.23 (m, 2H), 2.46 – 2.34 (m, 1H), 2.17 – 2.07 (m, 2H), 2.06 – 2.00 (m, 2H), 1.96 – 1.83 (m, 2H), 1.71 – 1.62 (m, 2H);

^{13}C NMR (100 MHz, $CDCl_3$): δ 49.0 (d, *J* = 16.2 Hz), 33.9, 30.0, 27.5, 18.1;

^{19}F NMR (376 MHz, $CDCl_3$): δ 53.1;

HRMS (EI+): m/z Calc. for $C_6H_{11}FO_2S$: 166.0464; found: 166.0470.

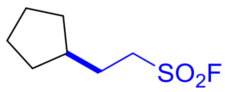

16

Compound **16**: 75% yield, colorless oil.

^{1}H NMR (400 MHz, $CDCl_3$): δ 3.39 – 3.33 (m, 2H), 1.97 – 1.78 (m, 5H), 1.70 – 1.52 (m, 4H), 1.18 – 1.10 (m, 2H);

^{13}C NMR (100 MHz, $CDCl_3$): δ 50.3 (d, *J* = 16.0 Hz), 38.4, 32.2, 29.3, 25.0;

^{19}F NMR (376 MHz, $CDCl_3$): δ 52.8;

HRMS (EI+): $[M\text{-}C_5H_9]^+$ Calc. for $C_2H_4FO_2S$: 110.9916; found: 110.9921.

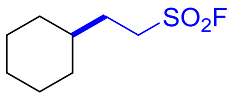

17

Compound **17**: 98% yield, colorless oil.

^{1}H NMR (400 MHz, $CDCl_3$): δ 3.39 – 3.34 (m, 2H), 1.86 – 1.80 (m, 2H), 1.77 – 1.72 (m, 5H), 1.46 – 1.35 (m, 1H), 1.29 – 1.14 (m, 3H), 1.00 - 0.91 (m, 2H);

^{13}C NMR (100 MHz, $CDCl_3$): δ 49.0 (d, *J* = 16.1 Hz), 36.2, 32.6, 30.3, 26.1, 25.9;

^{19}F NMR (376 MHz, $CDCl_3$): δ 52.0;

HRMS (EI+): m/z Calc. for $C_8H_{15}FO_2S$: 194.0777; found: 194.0782.

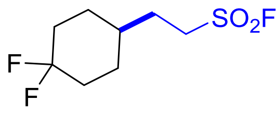

18

Compound **18**: 75% yield, colorless oil.

^{1}H NMR (400 MHz, $CDCl_3$): δ 3.42 – 3.36 (m, 2H), 2.15 – 2.08 (m, 2H), 1.94 – 1.89 (m, 2H), 1.82 – 1.65 (m, 4H), 1.59 – 1.56 (m, 1H), 1.38 – 1.29 (m, 2H);

^{13}C NMR (100 MHz, $CDCl_3$): δ 122.9 (dd, *J* = 241.2, 237.8 Hz), 48.8 (d, *J* = 16.7 Hz), 34.2, 33.1 (dd, *J* = 25.4, 23.1 Hz), 29.1 (d, *J* = 2.7 Hz), 28.3 (d, *J* = 9.7 Hz);

^{19}**F NMR** (376 MHz, $CDCl_3$): δ 53.5, -92.4 (d, J = 237.0 Hz), -102.4 (d, J = 236.5 Hz);

HRMS (EI+): $[M-HF]^+$ Calc. for $C_8H_{12}F_2O_2S$: 210.0526; found: 210.0531.

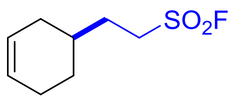

19

Compound **19**: 46% yield, colorless oil.

^{1}**H NMR** (400 MHz, $CDCl_3$): δ 5.71 – 5.62 (m, 2H), 3.43 – 3.38 (m, 2H), 2.16 – 2.09 (m, 3H), 1.96 – 1.90 (m, 2H), 1.79 – 1.69 (m, 3H), 1.36 – 1.29 (m, 1H);

^{13}**C NMR** (100 MHz, $CDCl_3$): δ 127.1, 125.2, 49.0 (d, J = 16.4 Hz), 32.2, 31.0, 29.5, 28.1, 24.6;

^{19}**F NMR** (376 MHz, $CDCl_3$): δ 53.0;

HRMS (EI+): m/z Calc. for $C_8H_{13}FO_2S$: 192.0620; found: 192.0629.

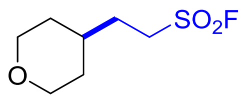

20

Compound **20**: 85% yield, colorless oil.

^{1}**H NMR** (400 MHz, $CDCl_3$): δ 3.98 (d, J = 10.6 Hz, 2H), 3.37 (t, J = 12.2 Hz, 4H), 1.93 – 1.87 (m, 2H), 1.71 – 1.60 (m, 3H), 1.38 – 1.29 (m, 2H);

^{13}**C NMR** (100 MHz, $CDCl_3$): δ 67.5, 48.3 (d, J = 16.8 Hz), 33.5, 32.3, 30.0;

^{19}**F NMR** (376 MHz, $CDCl_3$): δ 53.41;

HRMS (ESI+): $[M+Na]^+$ Calc. for $C_7H_{13}FNaO_3S$: 219.0462; found: 219.0467.

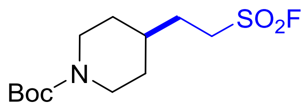

21

Compound **21**: 98% yield, colorless oil.

^{1}H NMR (400 MHz, $CDCl_3$): δ 4.16 – 4.04 (m, 2H), 3.40 – 3.35 (m, 2H), 2.66 (t, *J* = 11.6 Hz, 2H), 1.90 – 1.84 (m, 2H), 1.67 – 1.58 (m, 3H), 1.43 (s, 9H), 1.18 – 1.09 (m, 2H);

^{13}C NMR (100 MHz, $CDCl_3$): δ 154.6, 79.5, 48.4 (d, *J* = 16.6 Hz), 43.5, 34.5, 31.4, 29.6, 28.3;

^{19}F NMR (376 MHz, $CDCl_3$) δ 53.4;

HRMS (ESI+): $[M+Na]^+$ Calc. for $C_{12}H_{22}FNNaO_4S$: 318.1146; found: 318.1150.

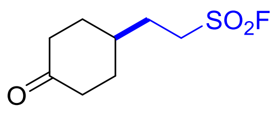

22

Compound **22**: 50% yield, colorless oil.

^{1}H NMR (400 MHz, $CDCl_3$): δ 3.46 – 3.42 (m, 2H), 2.45 – 2.32 (m, 4H), 2.12 – 2.08 (m, 2H), 2.01 – 1.97 (m, 3H), 1.53 – 1.43 (m, 2H);

^{13}C NMR (100 MHz, $CDCl_3$): δ 210.1, 49.0 (d, *J* = 16.8 Hz), 40.2, 34.5, 31.9, 29.0;

^{19}F NMR (376 MHz, $CDCl_3$): δ 53.7;

HRMS (ESI+): $[M+Na]^+$ Calc. for $C_8H_{13}FNaO_3S$: 231.0642; found: 231.0647.

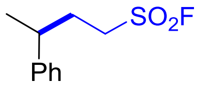

23

Compound **23**: 60% yield, colorless oil.

^{1}H NMR (400 MHz, $CDCl_3$): δ 7.35 (t, *J* = 7.3 Hz, 2H), 7.26 (t, *J* = 7.4 Hz, 1H), 7.18 (d, *J* = 7.4 Hz, 2H), 3.27 – 3.10 (m, 2H), 2.92 – 2.84 (m, 1H), 2.31 – 2.13 (m, 2H), 1.35 (d, *J* = 6.8 Hz, 3H);

^{13}C NMR (100 MHz, $CDCl_3$): δ 143.8, 129.0, 127.1, 126.8, 49.3 (d, *J* = 16.4 Hz), 38.5, 31.3, 22.2;

^{19}F NMR (376 MHz, $CDCl_3$): δ 53.3;

HRMS (EI+): m/z Calc. for $C_{10}H_{13}FO_2S$: 216.0620; found: 216.0619.

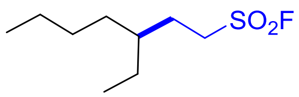

24

Compound **24**: 98% yield, colorless oil.

^{1}H NMR (400 MHz, $CDCl_3$): δ 3.35 – 3.30 (m, 2H), 1.93 – 1.88 (m, 2H), 1.47 – 1.26 (m, 9H), 0.92 – 0.87 (m, 6H);

^{13}C NMR (100 MHz, $CDCl_3$): δ 48.9 (d, *J* = 16.1 Hz), 37.6, 32.2, 28.6, 26.4, 25.3, 22.9, 14.0, 10.5;

^{19}F NMR (376 MHz, $CDCl_3$): δ 52.8;

HRMS (EI+): $[M\text{-}C_4H_9]^+$ Calc. for $C_5H_{10}FO_2S$: 153.0386; found: 153.0388.

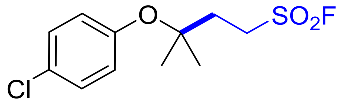

25

Compound **25**: 51% yield, colorless oil.

^{1}H NMR (400 MHz, $CDCl_3$): δ 7.25 (d, *J* = 6.8 Hz, 2H), 6.87 (d, *J* = 7.4 Hz, 2H), 3.70 – 3.64 (m, 2H), 2.29 – 2.24 (m 2H), 1.31 (s, 6H);

^{13}C NMR (100 MHz, $CDCl_3$): δ 152.7, 129.5, 129.3, 125.0, 78.4, 46.8 (d, *J* = 17.5 Hz), 35.8, 26.2;

^{19}F NMR (376 MHz, $CDCl_3$): δ 52.9;

HRMS (ESI+): $[M+Na]^+$ Calc. for $C_{11}H_{14}ClFNaO_3S$: 303.0228; found: 303.0233.

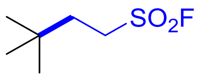

26

Compound **26**: 60% yield, colorless oil.

^{1}H NMR (400 MHz, $CDCl_3$): δ 3.36 – 3.30 (m, 2H), 1.83 (t, *J* = 7.8, 2H), 0.97 (s, 9H);

^{13}C NMR (100 MHz, $CDCl_3$): δ 47.7 (d, *J* = 16.5 Hz), 36.4, 30.1, 28.7;

^{19}F NMR (376 MHz, $CDCl_3$): δ 52.2;

HRMS (EI+): $[M-CH_3]^+$ Calc. for $C_5H_{10}FO_2S$: 153.0386; found: 153.0385.

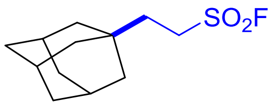

27

Compound **27**: 81% yield, colorless oil.

^{1}H NMR (400 MHz, $CDCl_3$): δ 3.35 – 3.30 (m, 2H), 2.01 (s, 3H), 1.75 – 1.68 (m, 5H), 1.63 (d, *J* = 12.2 Hz, 3H), 1.50 (s, 6H);

^{13}C NMR (100 MHz, $CDCl_3$): δ 46.2 (d, *J* = 16.3 Hz), 41.7, 36.7, 36.6, 31.9, 28.3;

^{19}F NMR (376 MHz, $CDCl_3$): δ 52.2;

HRMS (EI+): m/z Calc. for $C_{12}H_{19}FO_2S$: 246.1090; found: 246.1097.

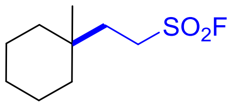

28

Compound **28**: 85% yield, colorless oil.

^{1}H NMR (400 MHz, $CDCl_3$): δ 3.44 – 3.28 (m, 2H), 1.86 (t, *J* = 8.2, 2H), 1.49 – 1.44 (m, 5H), 1.39 – 1.28 (m, 5H), 0.92 (s, 3H);

^{13}C NMR (100 MHz, $CDCl_3$): δ 46.9 (d, *J* = 16.2 Hz), 37.2, 34.5, 32.5, 26.0, 24.3, 21.7;

^{19}F NMR (376 MHz, $CDCl_3$): δ 52.2;

HRMS (EI+): $[M-CH_3]^+$ Calc. for $C_8H_{14}FO_2S$: 193.0699; found: 193.0701.

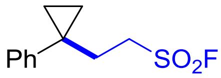

29

Compound **29**: 72% yield, colorless oil.

^{1}H NMR (400 MHz, $CDCl_3$): δ 7.34 – 7.22 (m, 5H), 3.33 – 3.28 (m, 2H), 2.17 (d, *J* = 8.2 Hz, 2H), 0.96 – 0.93 (m, 2H), 0.83 – 0.80 (m, 2H);

^{13}C NMR (100 MHz, $CDCl_3$): δ 141.9, 128.8, 128.8, 127.1, 49.1 (d, *J* = 16.0 Hz), 34.2, 24.3, 13.1;

^{19}F NMR (376 MHz, $CDCl_3$): δ 53.3;

HRMS (ESI+): $[M+Na]^+$ Calc. for $C_{11}H_{13}FNaO_2S$: 251.0512; found: 251.0512.

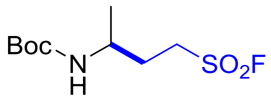

30

Compound **30**: 84% yield, white solid.

^{1}H NMR (400 MHz, $CDCl_3$): δ 4.40 (d, *J* = 5.8 Hz, 1H), 3.79 (s, 1H), 3.48 – 3.43 (m, 2H), 2.16 – 2.07 (m, 1H), 1.99 – 1.89 (m, 1H), 1.44 (s, 9H), 1.22 (d, *J* = 6.5 Hz, 3H);

^{13}C NMR (100 MHz, $CDCl_3$): δ 155.4, 80.0, 48.3 (d, *J* = 17.7 Hz), 45.2, 31.3, 28.3, 21.3;

^{19}F NMR (376 MHz, $CDCl_3$): δ 52.6;

HRMS (ESI+): $[M+Na]^+$ Calc. for $C_9H_{18}FNNaO_4S$: 278.0833; found: 278.0830.

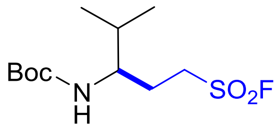

31

Compound **31**: 80% yield, white solid.

^{1}H NMR (400 MHz, $CDCl_3$): δ 4.38 (d, *J* = 8.4 Hz, 1H), 3.51 – 3.43 (m, 3H), 2.18 – 2.11 (m, 1H), 1.89 – 1.71 (m, 2H), 1.44 (s, 9H), 0.94 (t, *J* = 6.8 Hz, 6H);

^{13}C NMR (100 MHz, $CDCl_3$): δ 156.0, 80.0, 54.4, 48.8 (d, *J* = 17.2 Hz), 32.6, 28.3, 27.2, 19.0, 17.8;

^{19}F NMR (376 MHz, $CDCl_3$): δ 52.8;

HRMS (ESI+): $[M+Na]^+$ Calc. for $C_{11}H_{22}FNNaO_4S$: 306.1146; found: 306.1143.

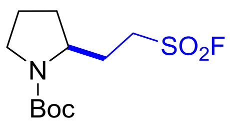

32

Compound **32**: 87% yield, colorless oil.

^{1}H NMR (400 MHz, $CDCl_3$): δ 3.94 (s, 1H), 3.48 – 3.31 (m, 4H), 2.16 (s, 1H), 2.02 (s, 2H), 1.87 (s, 2H), 1.66 – 1.60 (m, 1H), 1.45 (s, 9H);

^{13}C NMR (126 MHz, $CDCl_3$): δ 155.3, 154.4, 80.2, 79.8, 65.5, 55.4, 48.9 (d, J = 14.2 Hz), 48.2 (d, J = 14.2 Hz), 46.7, 46.4, 30.8, 29.4, 28.6, 28.4, 23.7, 23.0;

^{19}F NMR (376 MHz, $CDCl_3$): δ 52.95, 52.3;

HRMS (ESI+): $[M+Na]^+$ Calc. for $C_{11}H_{20}FNNaO_4S$:304.0989; found: 304.0986.

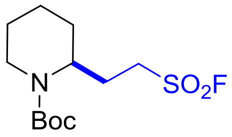

33

Compound **33**: 33% yield, colorless oil. (close to the byproduct of oxidized HE on column)

^{1}H NMR (400 MHz, $CDCl_3$): δ 4.38 (s, 1H), 4.02 (d, J = 13.0 Hz, 1H), 3.44 – 3.38 (m, 1H), 3.29 – 3.22 (m, 1H), 2.73 (t, J = 13.3 Hz, 1H), 2.49 – 2.39 (m, 1H), 1.97 – 1.89 (m, 1H), 1.75 – 1.53 (m, 6H), 1.46 (s, 9H);

^{13}C NMR (100 MHz, $CDCl_3$): δ 154.9, 80.2, 48.8, 48.3 (d, J = 17.1 Hz), 38.8, 28.8, 28.4, 25.2, 24.1, 18.9;

^{19}F NMR (376 MHz, $CDCl_3$): δ 53.0;

HRMS (ESI+): $[M+Na]^+$ Calc. for $C_{12}H_{22}FNNaO_4S$:318.1146; found: 318.1143.

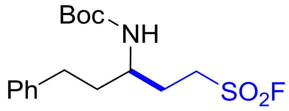

34

Compound **34**: 65% yield, colorless oil.

^{1}H NMR (400 MHz, $CDCl_3$): 7.31 – 7.15 (m, 5H), 4.38 (d, *J* = 9.0 Hz, 1H), 3.69 (s, 1H), 3.47 – 3.43 (m, 2H), 2.76 – 2.61 (m, 2H), 2.20 – 2.12 (m, 1H), 1.99 – 1.72 (m, 3H), 1.49 (s, 9H);

^{13}C NMR (100 MHz, $CDCl_3$): δ 155.7, 140.7, 128.6, 128.3, 126.2, 80.1, 49.1, 48.3 (d, *J* = 17.1 Hz), 37.3, 32.2, 30.0, 28.3;

^{19}F NMR (376 MHz, $CDCl_3$): δ 52.9;

HRMS (ESI+): $[M+Na]^+$ Calc. for $C_{16}H_{24}FNNaO_4S$:368.1302; found: 368.1300.

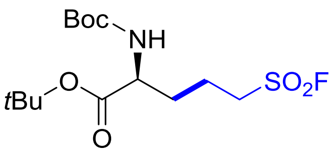

35

Compound **35**: 58% yield, white solid.

^{1}H NMR (400 MHz, $CDCl_3$): δ 5.14 (d, *J* = 5.6 Hz, 1H), 4.21 (s, 1H), 3.54 – 3.38 (m, 2H), 2.08 – 1.99 (m, 3H), 1.85 – 1.79 (m, 1H), 1.48 (s, 9H), 1.45 (s, 9H);

^{13}C NMR (100 MHz, $CDCl_3$): δ 170.9, 155.4, 82.8, 80.2, 52.8, 50.2 (d, *J* = 16.7 Hz), 31.3, 29.7, 28.3, 28.0, 19.6;

^{19}F NMR (376 MHz, $CDCl_3$): δ 53.8;

HRMS (ESI+): $[M+Na]^+$ Calc. for $C_{14}H_{26}FNNaO_6S$:378.1357; found: 378.1356.

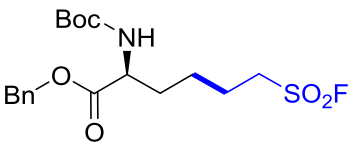

36

Compound **36**: 64% yield, white solid.

^{1}H NMR (400 MHz, $CDCl_3$): δ 7.36 (s, 5H), 5.23 (d, *J* = 12.1 Hz, 1H), 5.13 (d, *J* = 12.4 Hz, 1H), 5.08 (s, br, 1H), 4.38 – 4.33 (m, 1H), 3.27 – 3.23 (m, 2H), 1.96 – 1.82 (m, 3H), 1.74 – 1.49 (m, 3H), 1.43 (s, 9H);

^{13}C NMR (100 MHz, $CDCl_3$): δ 172.1, 155.3, 135.2, 128.7, 128.6, 128.5, 80.1, 67.2, 52.9, 50.5 (d, *J* = 16.5 Hz), 32.1, 28.3, 23.5, 22.9;

^{19}F NMR (376 MHz, $CDCl_3$): δ 53.6;

HRMS (ESI+): $[M+Na]^+$ Calc. for $C_{18}H_{26}FNNaO_6S$:426.1357; found: 426.1355.

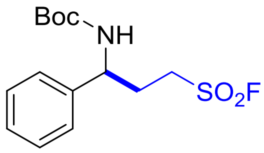

37

Compound **37**: 40% yield, white solid.

^{1}H NMR (400 MHz, $CDCl_3$): δ 7.41 – 7.31 (m, 3H), 7.27 (d, *J* = 8.6 Hz, 2H)，4.85 (d, *J* = 7.5 Hz, 1H), 4.78 – 4.74 (m, 1H), 3.47 – 3.33 (m, 2H), 2.47 – 2.37 (m, 2H), 1.43 (s, 9H);

^{13}C NMR (126 MHz, $CDCl_3$): δ 155.2, 139.9, 129.3, 128.4, 126.2, 80.4, 53.4, 48.3 (d, *J* = 17.5 Hz), 30.4, 28.3;

^{19}F NMR (376 MHz, $CDCl_3$): δ 53.2;

HRMS (ESI+): $[M+Na]^+$ Calc. for $C_{14}H_{20}FNNaO_4S$: 340.0989; found: 340.0988.

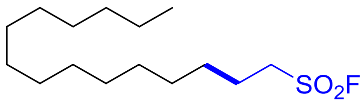

38

Compound **38**: 75% yield, white solid.

^{1}H NMR (400 MHz, $CDCl_3$): δ 3.37 – 3.33 (m, 2H), δ 1.98 – 1.91 (m, 2H), δ 1.51 – 1.44 (m, 2H), δ 1.36 – 1.32 (m, 22H), 0.88 (t, *J* = 6.2 Hz, 3H);

^{13}C NMR (100 MHz, $CDCl_3$): δ 50.9 (d, *J* = 16.0 Hz), 31.9, 29.7, 29.64 (2C), 29.61, 29.5, 29.43, 29.35, 29.1, 28.8, 27.9, 23.4, 22.7, 14.1;

^{19}F NMR (376 MHz, $CDCl_3$): δ 53.3;

HRMS (EI+): m/z Calc. for $C_{15}H_{31}FO_2S$: 294.2029; found: 294.2030.

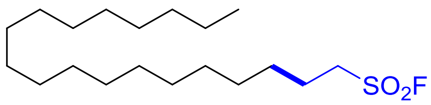

39

Compound **39**: 86% yield, white solid.

^{1}H NMR (400 MHz, $CDCl_3$): δ 3.37 – 3.33 (m, 2H), δ 1.98 – 1.91 (m, 2H), δ 1.51 – 1.44 (m, 2H), δ 1.33 – 1.22 (m, 30H), 0.88 (t, *J* = 6.6 Hz, 3H);

^{13}C NMR (100 MHz, $CDCl_3$): δ 50.9 (d, *J* = 16.0 Hz), 31.9, 29.69 (5C), 29.65 (2C), 29.61, 29.5, 29.43, 29.36, 29.1, 28.8, 27.9, 23.4, 22.7, 14.1;

^{19}F NMR (376 MHz, $CDCl_3$): δ 53.3;

HRMS (EI+): m/z Calc. for $C_{19}H_{39}FO_2S$: 350.2655; found: 350.2659.

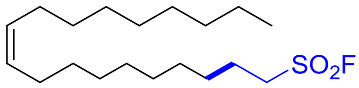

40

Compound **40**: 78% yield, white solid.

^{1}H NMR (400 MHz, $CDCl_3$): δ 5.39 – 5.31 (m, 2H), δ 3.38 – 3.32 (m, 2H), δ 2.05 – 1.90 (m, 6H), δ 1.49 – 1.44 (m, 2H), δ 1.38 – 1.23 (m, 22H), 0.88 (t, *J* = 6.3 Hz, 3H);

^{13}C NMR (100 MHz, $CDCl_3$): δ 130.0, 129.7, 50.9 (d, *J* = 16.0 Hz), 31.9, 29.8, 29.7, 29.5, 29.3 (3C), 29.2, 29.1, 28.8, 27.9, 27.21, 27.15, 23.4, 22.7, 14.1;

^{19}F NMR (376 MHz, $CDCl_3$): δ 53.3;

HRMS (ESI+): $[M+Na]^+$ Calc. for $C_{19}H_{37}FNaO_2S$: 371.2391; found: 371.2391.

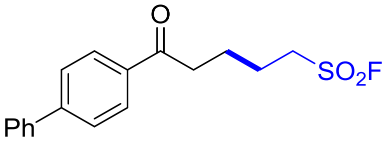

41

Compound **41**: 52% yield, white solid.

^{1}H NMR (400 MHz, $CDCl_3$): δ 8.03 (d, *J* = 8.0 Hz, 2H), 7.70 (d, *J* = 8.0 Hz, 2H), 7.63 (d, *J* = 7.6 Hz, 2H), 7.48 (t, *J* = 7.4 Hz, 2H), 7.41 (t, *J* = 7.3 Hz, 1H), 3.48 – 3.43 (m , 2H), 3.10 (t, *J* = 6.7 Hz, 2H), 2.12 – 2.04 (m, 2H), 2.01-1.94 (m, 2H);

^{13}C NMR (100 MHz, $CDCl_3$): δ 198.2, 146.1, 139.7, 135.2, 129.0, 128.6, 128.3, 127.34, 127.25, 50.8 (d, *J* = 16.4 Hz), 37.4, 23.2, 22.2;

^{19}F NMR (376 MHz, $CDCl_3$): δ 53.6;

HRMS (ESI+): $[M+Na]^+$ Calc. for $C_{17}H_{17}FNaO_3S$:343.0775; found: 343.0780.

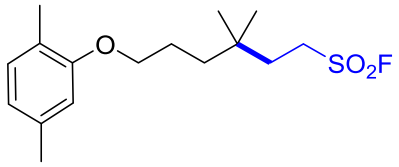

42

Compound **42**: 82% yield, white solid.

^{1}H NMR (400 MHz, $CDCl_3$): δ 7.03 (d, *J* = 7.4 Hz, 1H), 6.69 (d, *J* = 7.4 Hz, 1H), 6.64 (s, 1H), 3.95 (t, *J* = 5.9 Hz, 2H), 3.38 – 3.22 (m, 2H), 2.33 (s, 3H), 2.20 (s, 3H), 1.92 – 1.88 (m, 2H), 1.82 – 1.75 (m, 2H), 1.47 – 1.43 (m, 2H), 1.01 (s, 6H);

^{13}C NMR (100 MHz, $CDCl_3$): δ 156.8, 136.5, 130.3, 123.4, 120.8, 112.0, 67.8, 47.2 (d, *J* = 16.9 Hz), 37.6, 34.3, 32.4, 26.5, 24.0, 21.3, 15.8;

^{19}F NMR (376 MHz, $CDCl_3$): δ 52.2;

HRMS (ESI+): $[M+Na]^+$ Calc. for $C_{16}H_{25}FNaO_3S$:339.1401; found: 339.1400.

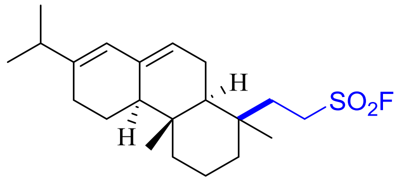

43

Compound **43**: 50% yield, white solid.

^{1}H NMR (400 MHz, $CDCl_3$): δ 5.70 (s, 1H), 5.21 (s, 1H), 3.46 – 3.32 (m, 2H), 2.80 (d, *J* = 7.9 Hz, 1H), 2.29 (t, *J* = 11.8 Hz, 1H), 2.21 – 2.15 (m, 1H), 2.06 – 1.90 (m, 3H), 1.85 – 1.75

(m, 3H), 1.61 – 1.44 (m, 4H), 1.32 – 1.19 (m, 2H), 1.16 (s, 3H), 1.14 – 1.02 (m, 2H), 0.99 (s, 3H), 0.97 (s, 3H), 0.85 (s, 3H);

^{13}C NMR (100 MHz, $CDCl_3$): δ 145.2, 137.1, 122.3, 121.9, 51.9, 49.4, 47.2 (d, *J* = 16.1 Hz), 40.4, 40.3, 39.1, 37.8, 37.2, 34.8, 27.0, 22.3, 21.3, 20.9, 20.3, 18.1, 16.5;

^{19}F NMR (376 MHz, $CDCl_3$): δ 52.5;

HRMS (ESI+): $[M+H]^+$ Calc. for $C_{21}H_{34}FO_2S$: 369.2258; found: 369.2256.

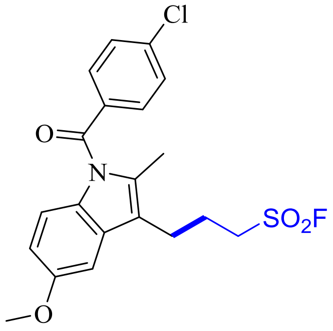

44

Compound **44**: 85% yield, white solid.

^{1}H NMR (400 MHz, $CDCl_3$): δ 7.66 (d, *J* = 7.0 Hz, 2H), 7.48 (d, *J* = 7.0 Hz, 2H), 6.90 (s, 1H), 6.84 (d, *J* = 9.0 Hz, 1H), 6.68 (d, *J* = 9.0 Hz, 1H), 3.84 (s, 3H), 3.43 – 3.39 (m, 2H), 2.90 (t, *J* = 7.2 Hz, 2H), 2.38 (s, 3H), 2.33 – 2.26 (m, 2H);

^{13}C NMR (100 MHz, $CDCl_3$): δ 168.2, 156.0, 139.3, 135.0, 133.8, 131.1, 130.9, 130.3, 129.1, 116.5, 115.1, 111.4, 100.9, 55.7, 50.1 (d, *J* = 16.4 Hz), 23.4, 21.9, 13.2;

^{19}F NMR (376 MHz, $CDCl_3$): δ 54.2;

HRMS (ESI+): $[M+Na]^+$ Calc. for $C_{20}H_{19}ClFNNaO_4S$: 446.0600; found: 446.0601.

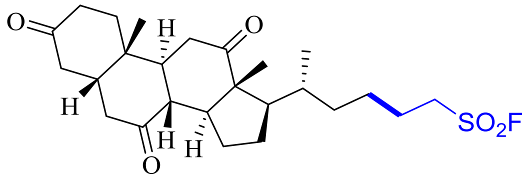

45

Compound **45**: 96% yield, white solid.

^{1}H NMR (400 MHz, $CDCl_3$): δ 3.39 – 3.33 (m, 2H), 2.94 – 2.81 (m, 3H), 2.36 – 1.80 (m, 15H), 1.62 – 1.43 (m, 4H), 1.40 (s, 3H), 1.31 – 1.18 (m, 4H), 1.07 (s, 3H), 0.84 (d, *J* = 5.7 Hz, 3H);

^{13}C NMR (100 MHz, $CDCl_3$): δ 211.9, 209.0 208.7, 56.9, 51.8, 50.9 (d, *J* = 15.8 Hz), 49.0, 46.8, 45.63, 45.55, 44.9, 42.8, 38.6, 36.4, 36.0, 35.7, 35.2, 34.6, 27.8, 25.1, 24.9, 23.7, 21.9, 18.9, 11.8;

^{19}F NMR (376 MHz, $CDCl_3$): δ 53.7;

HRMS (ESI+): $[M+Na]^+$ Calc. for $C_{25}H_{37}FNaO_5S$: 491.2238; found: 491.2240.

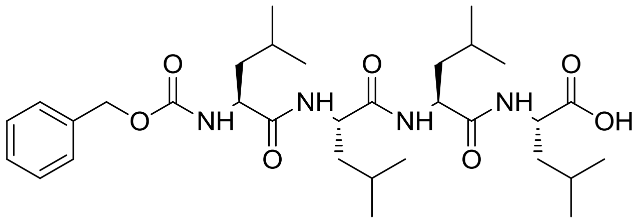

46

Compound **46**: white solid. Ordered from GL Biochem (Shanghai) Ltd.

^{1}H NMR (400 MHz, $CDCl_3$): δ 7.40 – 7.26 (m, 5H), 7.20 – 7.06 (m, 1H), 6.91 – 6.77 (m, 1H), 5.78 – 5.52 (m, 1H), 5.20 – 4.92 (m, 2H), 4.69 – 4.17 (m, 4H), 3.73 – 3.55 (m, 2H), 3.07 (q, *J* = 7.3 Hz, 2H), 1.74 – 1.46 (m, 8H), 1.41 – 1.34 (m, 9H), 0.95 – 0.81 (m, 15H);

^{13}C NMR (100 MHz, $CDCl_3$): δ 176.4, 172.3, 171.6, 171.2, 156.3, 136.5, 128.4, 127.9, 127.8, 66.7, 53.5, 53.1, 52.5, 51.7, 42.1, 41.9, 41.5, 41.2, 41.1, 24.9, 24.7, 24.6, 23.1, 22.9, 22.8, 22.6, 22.4, 22.2, 18.0, 11.6;

HRMS (ESI+): $[M+Na]^+$ Calc. for $C_{32}H_{52}N_4NaO_7$: 627.3728; found: 627.3738.

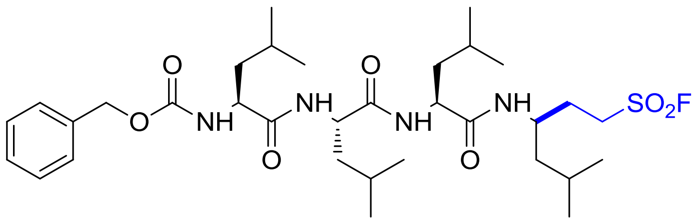

47

Compound **47**: 75% yield, white solid.

^{1}H NMR (400 MHz, $CDCl_3$): δ 7.39 – 7.32 (m, 5H), 7.02 (d , *J* = 8.1 Hz, 1H), 6.68 (d , *J* = 8.8 Hz, 1H), 6.25 (s, 1H), 5.15 – 5.07 (m, 3H), 4.57 (t , *J* = 9.7 Hz, 1H), 4.23 – 4.02 (m, 3H), 3.66 – 3.59 (m, 1H), 3.43 – 3.36 (m, 1H), 2.15 – 2.09 (m, 1H), 2.03 - 1.98 (m, 2H), 1.84 – 1.78 (m, 1H), 1.72 – 1.29 (m, 10H), 1.00 – 0.90 (m, 24H);

^{13}C NMR (126 MHz, $CDCl_3$): δ 173.8, 172.0, 171.4, 157.8, 134.9, 128.9, 128.8, 128.2, 68.1, 56.1, 53.6, 51.2, 48.1 (d, *J* = 16.2 Hz), 45.8, 44.2, 40.8, 40.1, 40.0, 29.5, 25.3, 25.2, 25.0, 24.8, 23.5, 23.06, 23.05, 22.8, 22.1, 21.6, 21.3, 21.0;

^{19}F NMR (376 MHz, $CDCl_3$): δ 52.6;

HRMS (ESI+): $[M+Na]^+$ Calc. for $C_{33}H_{55}FN_4NaO_7S$: 693.3668; found: 693.3669.

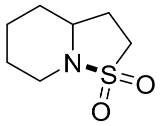

48

Compound **48**: 84% yield, colorless oil.

^{1}H NMR (400 MHz, $CDCl_3$): δ 3.47 (d, *J* = 11.1Hz, 1H), 3.23 – 3.16 (m, 1H), 3.11 – 3.03 (m, 1H), 2.99 – 2.94 (m, 1H), 2.63 (t, *J* = 12.1 Hz, 1H), 2.38 – 2.33 (m, 1H), 2.00 – 1.90 (m, 2H), 1.85 – 1.77 (m, 2H), 1.56 – 1.47 (m, 1H), 1.37 – 1.24 (m, 2H);

^{13}C NMR (100 MHz, $CDCl_3$): δ 56.5, 45.9, 41.3, 32.1, 26.7, 23.7, 23.1;

HRMS (ESI+): $[M+H]^+$ Calc. for $C_7H_{14}NO_2S$: 176.0740; found: 176.0739.

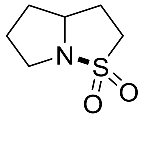

49

Compound **49**: 80% yield, colorless oil.

^{1}H NMR (400 MHz, $CDCl_3$): δ3.98 – 3.91 (m, 1H), 3.56 – 3.50 (m, 1H), 3.22 – 3.16 (m, 2H), 2.94 – 2.87 (m, 1H), 2.56 – 2.47 (m, 1H), 2.12 – 2.02 (m, 2H), 1.97 – 1.82 (m, 2H), 1.58 – 1.50 (m, 1H);

^{13}**C NMR** (100 MHz, $CDCl_3$): δ 61.9, 48.2, 46.1, 32.3, 26.9, 25.7;

HRMS (ESI+): $[M+Na]^+$ Calc. for $C_6H_{11}NNaO_2S$: 184.0403; found: 184.0403.

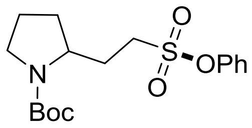

50

Compound **50**: 85% yield, colorless oil.

^{1}**H NMR** (400 MHz, $CDCl_3$): δ 7.46 – 7.35 (m, 2H), 7.34 – 7.23 (m, 3H), 3.95 (s, 1H), 3.61 – 3.13 (m, 4H), 2.22 (s, 1H), 2.14 – 1.95 (m, 2H), 1.93 – 1.78 (m, 2H), 1.69 – 1.58 (m, 1H), 1.45 (s, 9H);

^{13}**C NMR** (100 MHz, $CDCl_3$): δ 155.1, 154.5, 149.2, 129.9, 127.2, 122.1, 80.0, 79.5, 55.6, 48.2, 47.8, 46.6, 46.3, 31.1, 30.7, 29.2, 28.8, 28.4, 23.7, 23.0;

HRMS (ESI+): $[M+Na]^+$ Calc. for $C_{17}H_{25}NNaO_5S$: 378.1346; found: 378.1333.

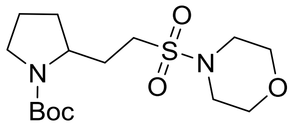

51

Compound **51**: 98% yield, yellow oil.

^{1}**H NMR** (400 MHz, $CDCl_3$) δ 3.90 (s, 1H), 3.81 – 3.64 (m, 4H), 3.52 – 3.16 (m, 6H), 3.07 – 2.79 (m, 2H), 2.14 – 1.78 (m, 5H), 1.68 – 1.58 (m, 1H), 1.44 (s, 9H);

^{13}**C NMR** (126 MHz, $CDCl_3$): δ 155.1, 154.5, 79.9, 79.4, 66.6, 55.9, 46.6, 46.5, 46.4, 46.1, 45.8, 31.1, 30.7, 28.5, 23.7, 23.0;

HRMS (ESI+): $[M+Na]^+$ Calc. for $C_{15}H_{28}N_2NaO_5S$: 371.1611; found: 371.1611.

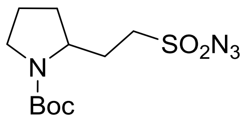

52

Compound **52**: 82% yield, colorless oil.

^{1}H NMR (400 MHz, $CDCl_3$): δ 4.03 – 3.78 (m, 1H), 3.58 – 3.14 (m, 4H), 2.23 – 2.07 (m, 1H), 2.06 – 1.93 (m, 2H), 1.91 – 1.74 (m, 2H), 1.68 – 1.57 (m, 1H), 1.45 (s, 9H);

^{13}C NMR (126 MHz, $CDCl_3$): δ 155.3, 154.4, 80.2, 79.7, 55.5, 53.8, 53.3, 46.7, 46.5, 31.2, 30.8, 29.7, 29.2, 28.4, 23.7, 23.0;

HRMS (ESI+): $[M+Na]^+$ Calc. for $C_{11}H_{20}N_4NaO_4S$: 327.1097; found: 327.1087.

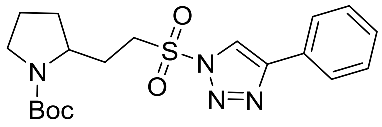

53

Compound **53**: 85% yield, white solid.

^{1}H NMR (400 MHz, $CDCl_3$): δ 8.28 (s, 1H), 7.84 (d, *J* = 7.5 Hz, 2H), 7.50 – 7.32 (m, 3H), 4.08 – 3.56 (m, 3H), 3.49 – 3.10 (m, 2H), 2.12 – 1.72 (m, 5H), 1.63 – 1.49 (m, 1H), 1.39 (s, 9H);

^{13}C NMR (126 MHz, $CDCl_3$): δ 155.3, 154.3, 147.3, 129.2, 129.0, 128.7, 128.6, 126.1, 119.7, 119.4, 80.4, 79.8, 55.3, 53.3, 52.8, 46.7, 46.4, 31.1, 30.7, 28.8, 28.4, 28.1, 23.7, 23.0;

HRMS (ESI+): $[M+Na]^+$ Calc. for $C_{19}H_{26}N_4NaO_4S$: 429.1567; found: 429.1573.

Supplementary Tables

Supplementary Table 1: Screening of photocatalyst.[a]

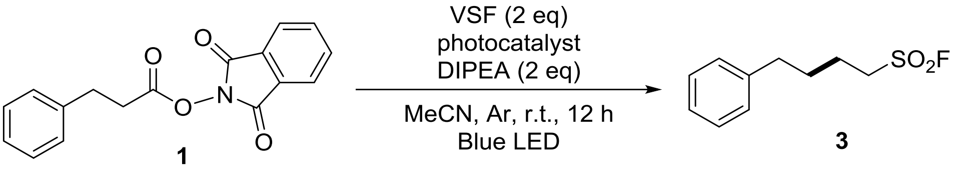

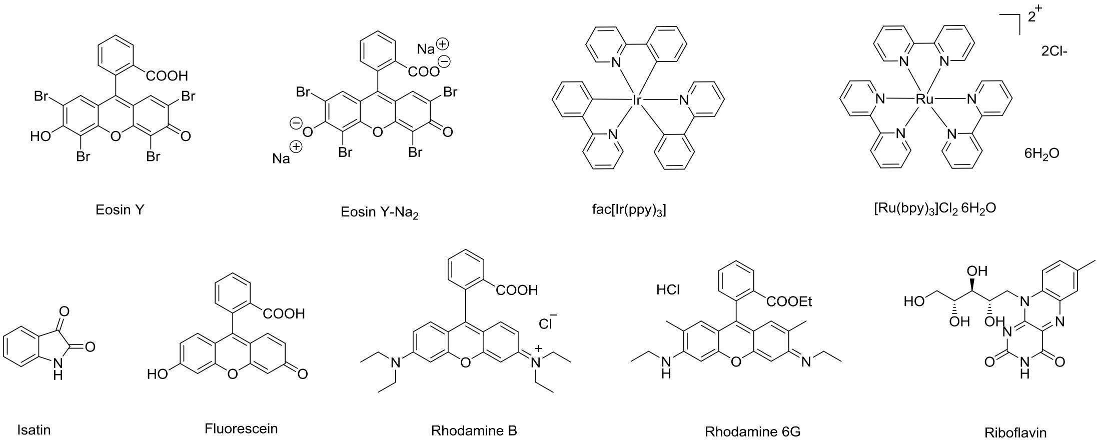

Entry	Photocatalyst	Yield (%)
1	$Ir(ppy)_3$ (2 mol%)	29%
2	$[Ru(bpy)_3]Cl_26H_2O$ (5 mol%)	24%
3	Eosin Y (5 mol%)	35%
4[b]	Eosin Y (10 mol%)	38%
5[c]	Eosin Y (10 mol%)	6%
6	Eosin Y-Na_2 (5 mol%)	37%
7	Isatin (5 mol%)	32%
8	Fluorescein (5 mol%)	26%
9	Rhodamine B (5 mol%)	17%
10	Rhodamine 6G (5 mol%)	26%
11	Riboflavin (5 mol%)	16%
12	No catalyst	14%

[a] Reaction condition: on 0.05 mmol scale, VSF (2 eq), photocatalyst, in MeCN (0.5 mL) at room temperature, under the irradiation of 18W x 2 blue LED bulbs, yields were determined by NMR. [b] In DCM, VSF (5 eq), Green LED. [c] In DCM, VSF (5 eq), Green LED, without degasing.

Supplementary Table 2: Screening of reductant amines.[a]

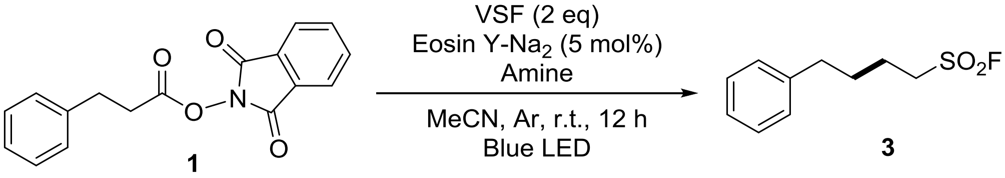

Entry	Amine	Yield (%)
1	DIPEA (2 eq)	37%
2	Et_3N (2 eq)	< 2
3	N,N-Dimethylaniline (2 eq)	N.P.
4	DBU (2 eq)	N.P.
5	TMEDA (2 eq)	< 2
6	PMDETA (2 eq)	N.P.
7	HE (2 eq)	89
8	HE (1.5 eq)	78
9	HE (1 eq)	65
10	HE/DIPEA (2 eq:1 eq)	73
11	HE/TEA (2 eq:1 eq)	29

[a] Reaction condition: on 0.05 mmol scale, VSF (2 eq), Eosin Y-Na_2 (5 mol%), in MeCN (0.5 mL) at room temperature, under the irradiation of 18W x 2 blue LED bulbs, yields were determined by ^{19}F NMR with $PhCF_3$ as an internal standard. N.P. = no product was observed.

Supplementary Table 3: Screening of solvent, component ratio, and light source.[a]

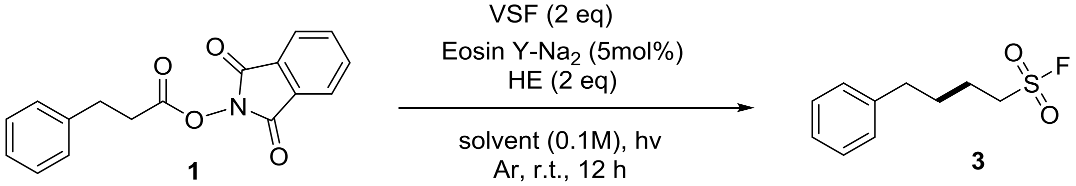

Entry	Light Source	Solvents	Yield (%)
1	Blue LED	MeCN	89
2	Blue LED	DMF	82
3	Blue LED	DMA	74
4	Blue LED	DMSO	83
5	Blue LED	DCM	44
6	Blue LED	EtOH	74
7	Blue LED	$PhCF_3$	37
8	Blue LED	Benzene	47
9[b]	Blue LED	MeCN	69
10[c]	Blue LED	MeCN	87
11[d]	Blue LED	MeCN	79
12[e]	Blue LED	MeCN	65
13	Green LED	MeCN	88
14[e]	Green LED	MeCN	12
15	In Dark	MeCN	N.P.

[a] Reaction condition: on 0.05 mmol scale, VSF (2 eq), Eosin Y-Na_2 (5 mol%), in MeCN (0.5 mL) at room temperature, under the irradiation of 18W x 2 blue LED bulbs. [b] With 1 equivalent of VSF. [c] With 3 equivalents of VSF. [d] With 1 mol% catalyst. [e] Without Eosin Y-Na_2.

Supplementary Discussion

*VSF stability in the presence of different amines (control experiments without ester **1**):*

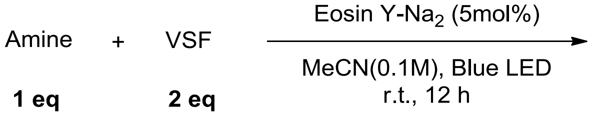

^{19}F NMR with $PhCF_3$ as an internal standard (VSF: 57.11 ppm; $PhCF_3$: -62.31 ppm):

a Amine = HE (HE:VSF = 1:2)

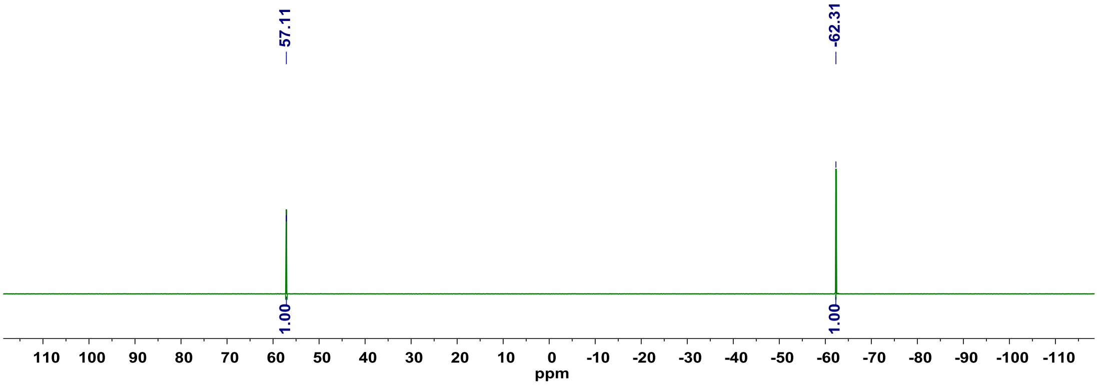

b Amine = DIPEA (DIPEA:VSF = 1:2)

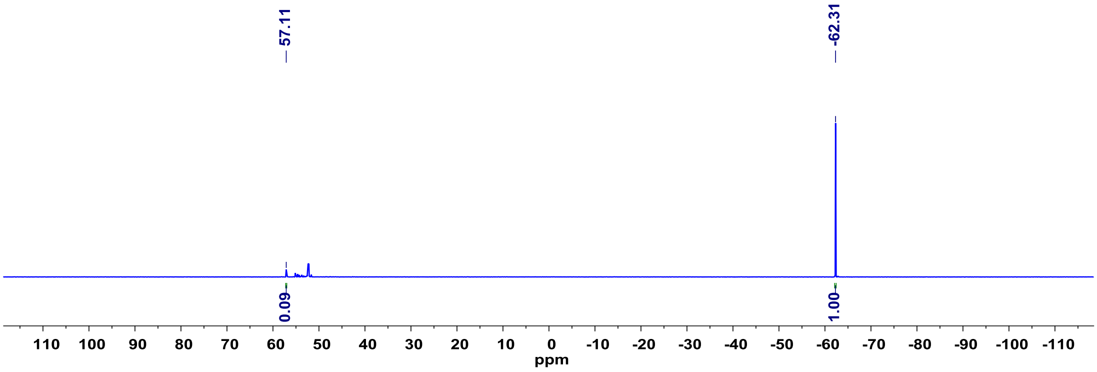

c Amine = TEA (TEA:VSF = 1:4)

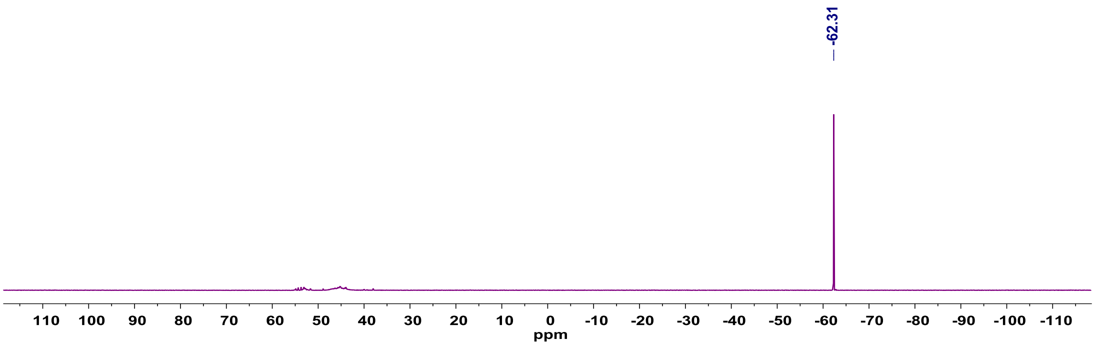

Supplementary Figure 1. ^{19}F NMR spectra for VSF stability experiment with different amines. **a**, Amine = HE. **b**, Amine = DIPEA. **c**, Amine = TEA.

VSF (^{19}F 57.11 ppm) in the presence of different amines after reaction:

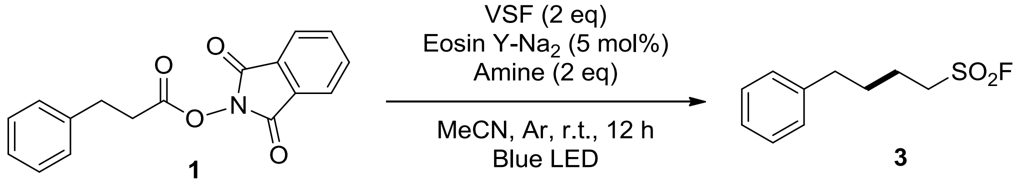

^{19}F NMR with $PhCF_3$ as an internal standard (VSF: 57.11 ppm, **3**: 52.59 ppm, $PhCF_3$: -62.31 ppm)

a Amine = HE

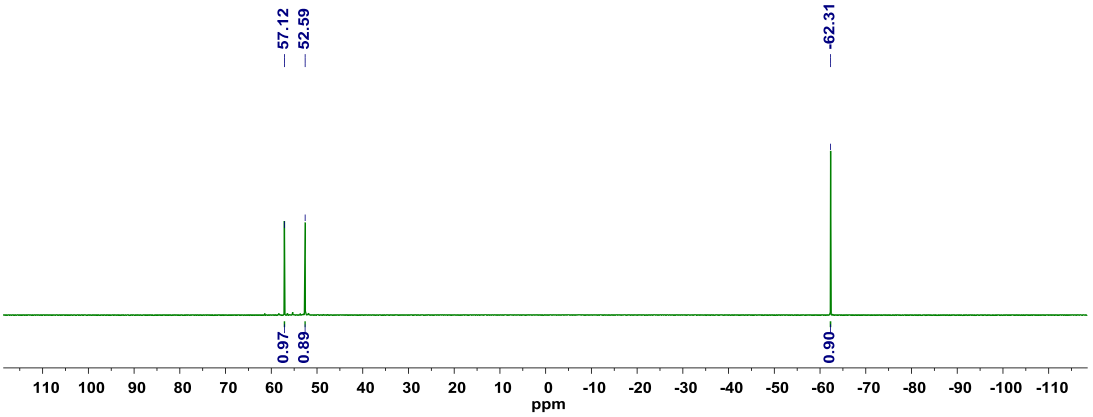

b Amine = DIPEA

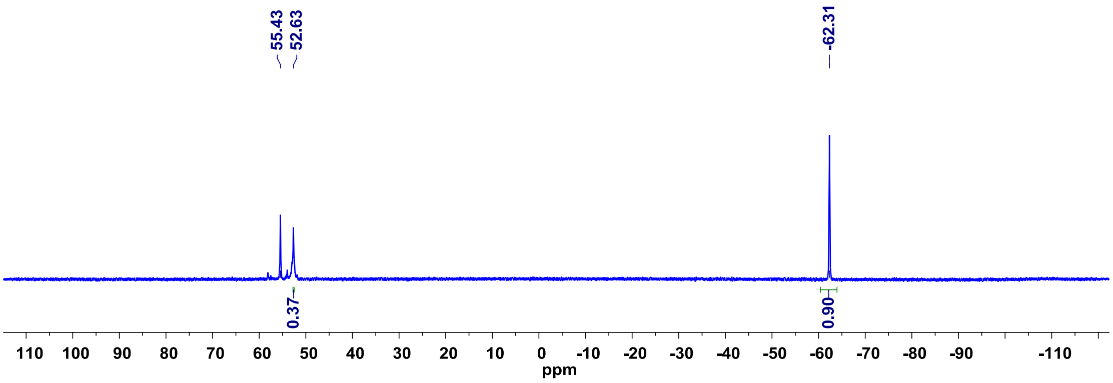

c Amine = TEA

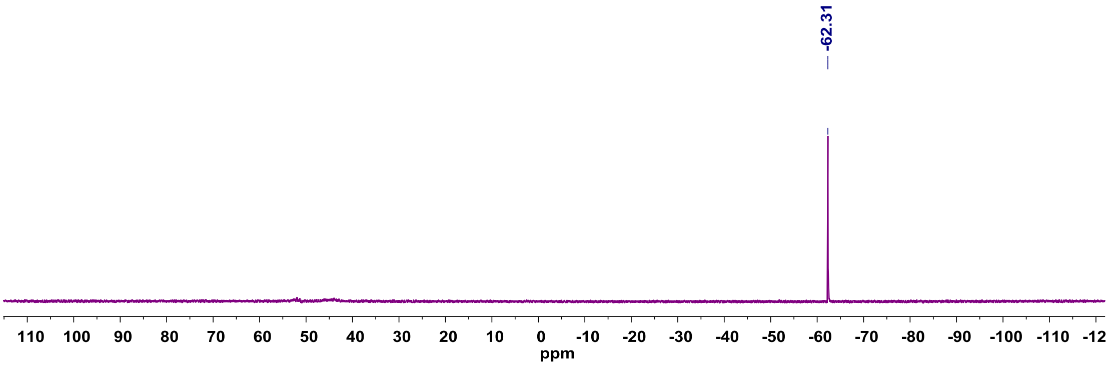

Supplementary Figure 2. ^{19}F NMR spectra for reactions with different amines. **a**, Amine = HE. **b**, Amine = DIPEA. **c**, Amine = TEA.

TEMPO trapping experiment

To a Schlenk tube (10 mL) equipped with stir bar were added NHPI redox-active ester **1** (29.5 mg, 0.1 mmol, 1.0 equiv.), Eosin Y-Na_2 (3.4 mg, 0.005 mmol, 0.05 equiv.), HE (50.6 mg, 0.20 mmol, 2 equiv.), TEMPO (31.3 mg, 0.20 mmol, 2 equiv.). After addition of dry MeCN (1 mL), VSF (16 μL, 0.20 mmol, 2 equiv.) was added via syringe. The reaction mixture was degassed with three freeze-pump-thaw cycles. The reaction mixture was stirred and irradiated with 18W×2 blue LED bulbs at room temperature for 12 hours. A sample of the reaction mixture was then submitted to HRMS analysis, which indicated the phenylethyl radical formed after decarboxylation and was trapped by TEMPO.

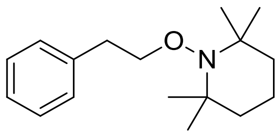

Chemical Formula: $C_{17}H_{27}NO$
m/z: 261.21 (100.0%), 262.21 (18.8%), 263.22 (1.7%)

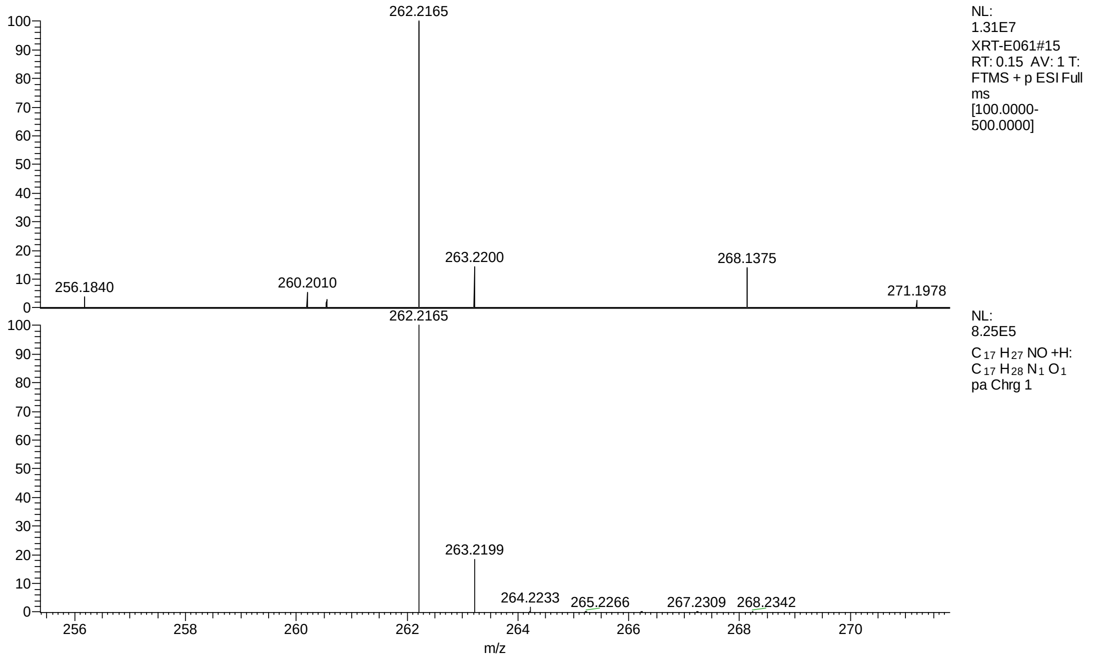

HRMS (ESI+): $[M+H]^+$ Calc. for $C_{17}H_{28}NO$: 262.2165; found: 262.2165.

Supplementary Figure 3. HRMS spectra for the TEMPO trapping reaction

Isotope-labeling experiment

The deuterated Hantzsch ester was synthesized according to the reported procedure.[13] An oven dried round bottom flask was charged with ethyl acetoacetate (1.6 ml, 12.48 mmol, 4 equiv.), D_2-paraformaldehyde (0.1 g, 3.12 mmol, 1 equiv.), ammonium acetate (0.48 g, 6.24 mmol, 2 equiv.) and water (6.5 ml), then the mixture was heated at 86 ℃. After 3 hours, the reaction mixture was allowed to cool down to room temperature and filtered. The precipitate was dried in vacuo to afford the desired compound as yellow solid (0.6 g, 76%). ^{1}H NMR (400 MHz, $CDCl_3$): δ 5.24 (s, 1H), 4.16 (q, J = 7.1 Hz, 4H), 2.18 (s, 6H), 1.27 (t, J = 6.8Hz, 6H). The reaction was performed under the standard conditions, and the deuterated product was isolated as a colorless oil.

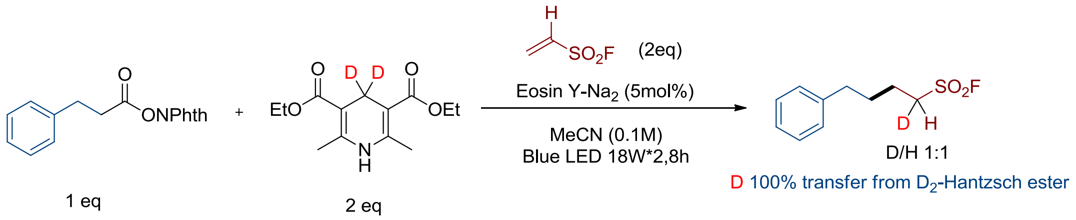

From the ^{1}H NMR (400 MHz, $CDCl_3$): δ 7.31 (t, J = 7.6 Hz, 2H), 7.24 - 7.16 (m, 3H), 3.37 - 3.32 (m, 1H), 2.69 (t, J = 7.3 Hz, 2H), 2.01 - 1.95 (m, 2H), 1.86 - 1.79 (m, 2H); we could see the interatration of the H_2C-SO_2F (δ 3.32-3.37 ppm) is only 1 proton, which meaned that the other proton was deuterium, coming from the deuterated Hantzsch ester.

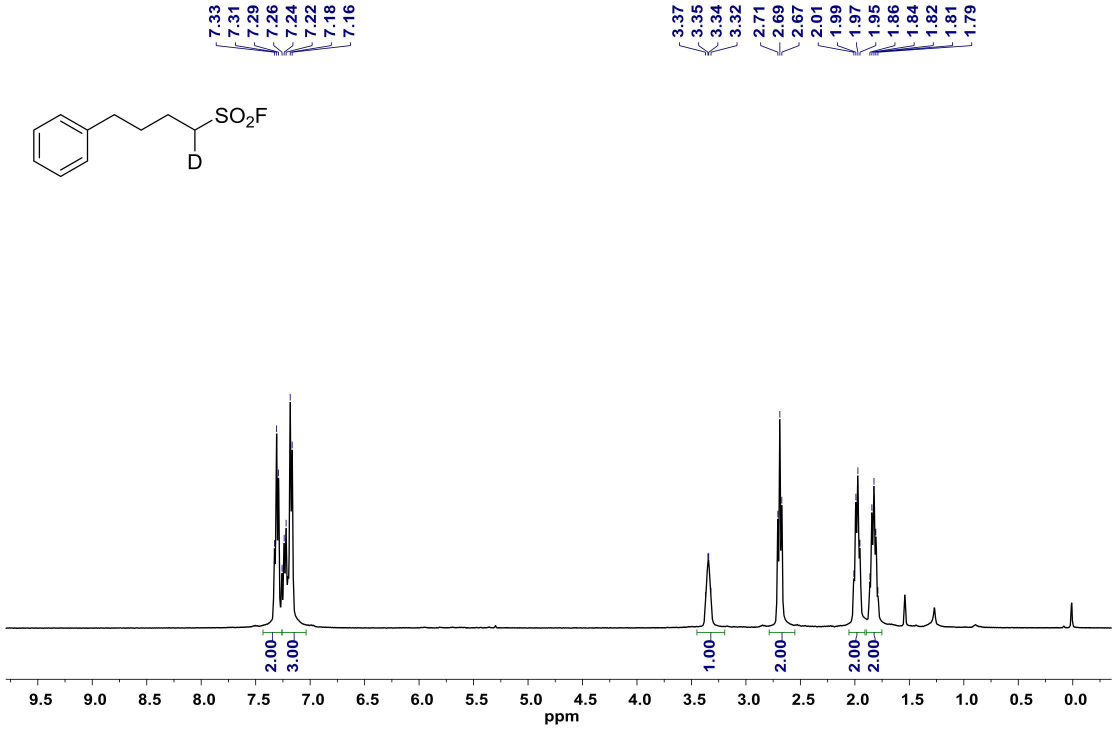

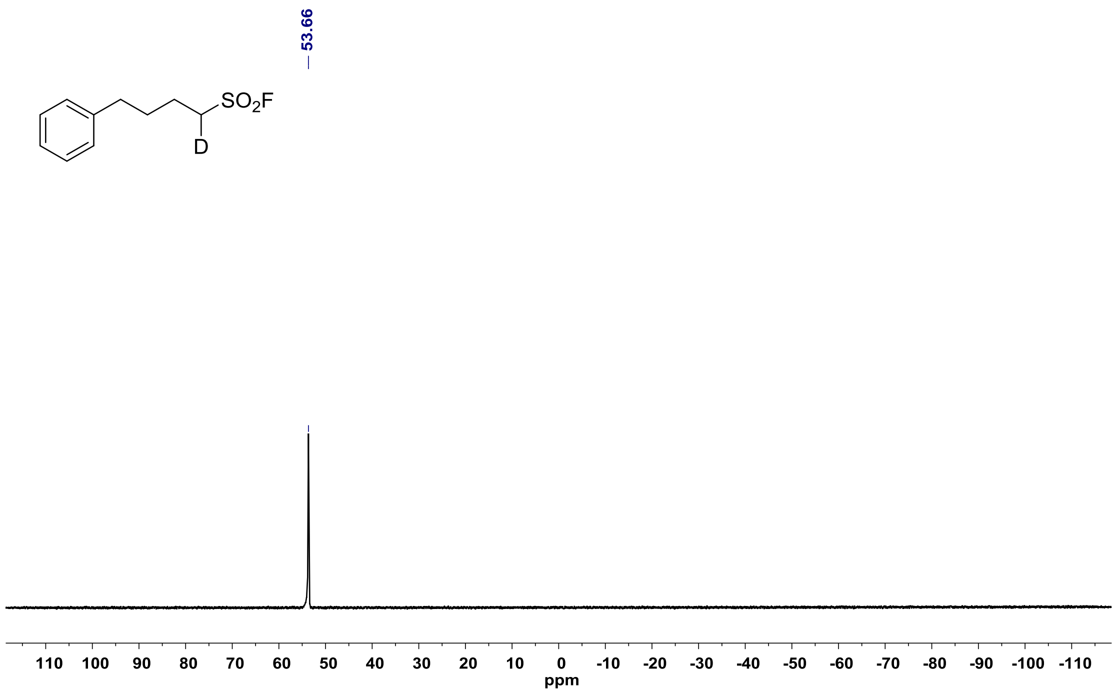

Supplementary Figure 4. ^{1}H and ^{19}F NMR spectra of the deuterium-labeled product

Proposed mechanism

Based on the above experiments and our results, as well as the reported eosin Y mediated photocatalysis,[11] a plausible mechanism was proposed as shown below.

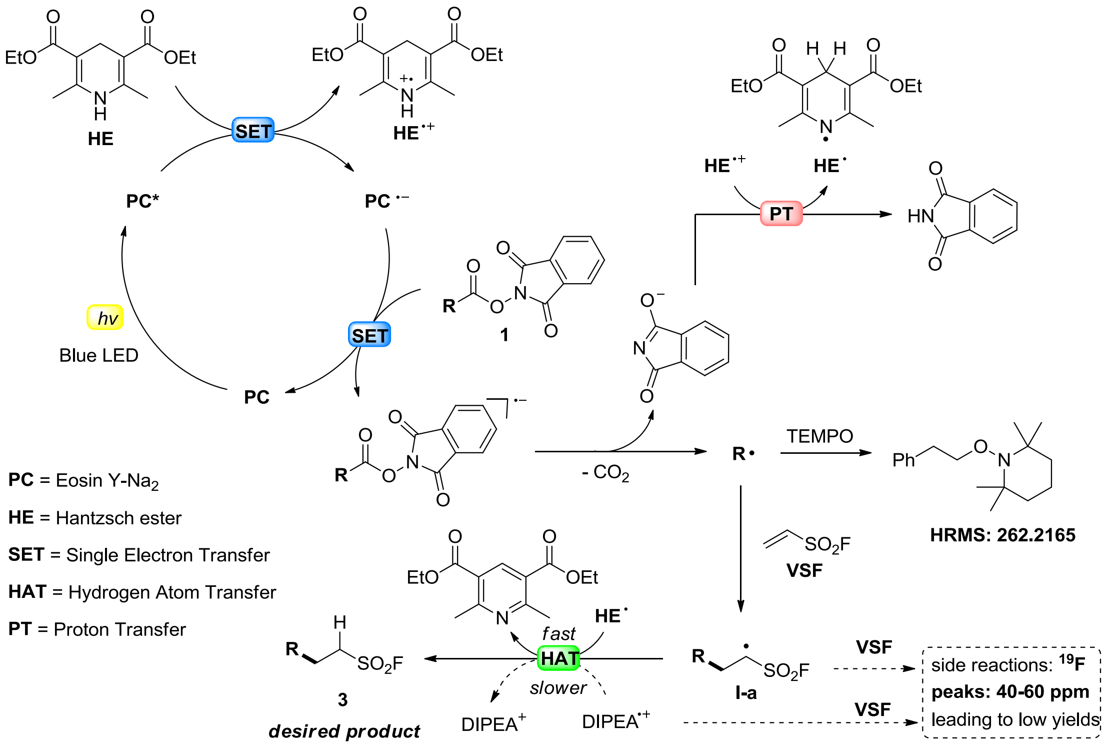

Supplementary Figure 5. Proposed reaction mechanism

The low yields with commonly used DIPEA and TEA may be ascribed to the radical species from DIPEA and TEA, which would cause side reactions to consume VSF. And, when the last HAT step with DIPEA is slow, the radical **I-a** formed through the R radical addition to VSF can also cause side reactions like polymerization to consume VSF. While, a fast hydrogen atom transfer to the radical intermediate **I-a** from HE would efficiently suppress the side reactions, as confirmed by the isotope-labeling experiments in part 3.4.

Supplementary Note 1

Stability test of aliphatic sulfonyl fluorides in physiological buffer:

The stability of sulfonyl fluoride products in physiological buffers was tested in phosphate-buffered saline (PBS buffer, pH = 7.2) at room temperature. ^{19}F NMR analysis was employed to monitor the change of the sulfonyl fluoride content, and 2,2,2-trifluroethanol (F_3CCH_2OH) was used as an internal standard and isopropanol or DMSO was used to dissolve the sulfonyl fluorides, which are not soluble in the aqueous PBS buffer.

A typical procedure: product **3** (4.4 mg, 0.02 mmol) was dissolved in 0.48 mL of isopropanol, followed by the addition of PBS buffer (0.50 mL) and 20 μL of F_3CCH_2OH isopropanol solution (1 M). The mixture reached a homogeneous, clear solution (PBS/isopropanol, 1:1, v/v), which was stirred at room temperature. The ratios of sulfonyl fluoride/F_3CCH_2OH were checked by ^{19}F NMR analysis at the beginning, and after 2 h, 4 h, 8 h, 24 h. The overall volume ratios PBS/isopropanol 7:3 and PBS/DMSO 1:3 were used for the proline-derived sulfonyl fluoride product **32** and tetrapeptide product **47**. The ^{19}F NMR analysis showed that the ratios of sulfonyl fluoride/F_3CCH_2OH were almost constant for all the three product tested, suggesting the three aliphatic sulfonyl fluorides are quite stable (no detectable loss or decomposition by ^{19}F NMR) in this physiological aqueous condition.

0 h

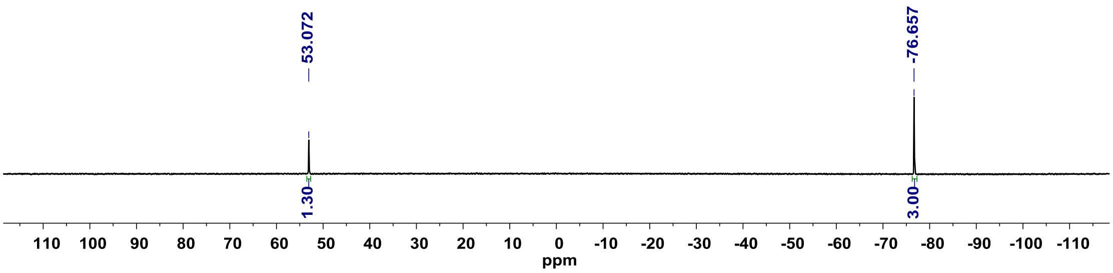

24 h

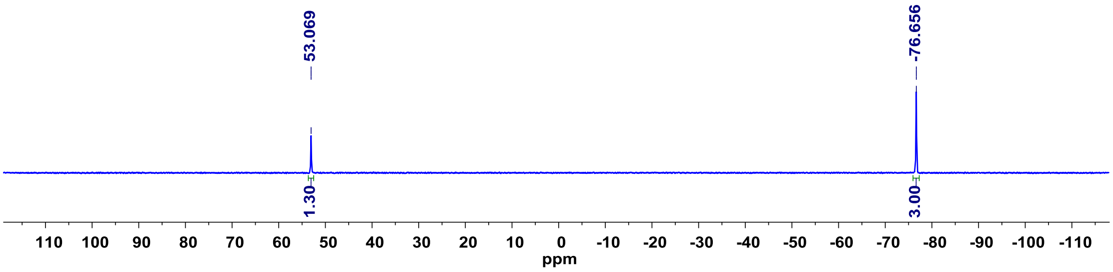

Supplementary Figure 6. ^{19}F NMR spectra for the stability test of **3**

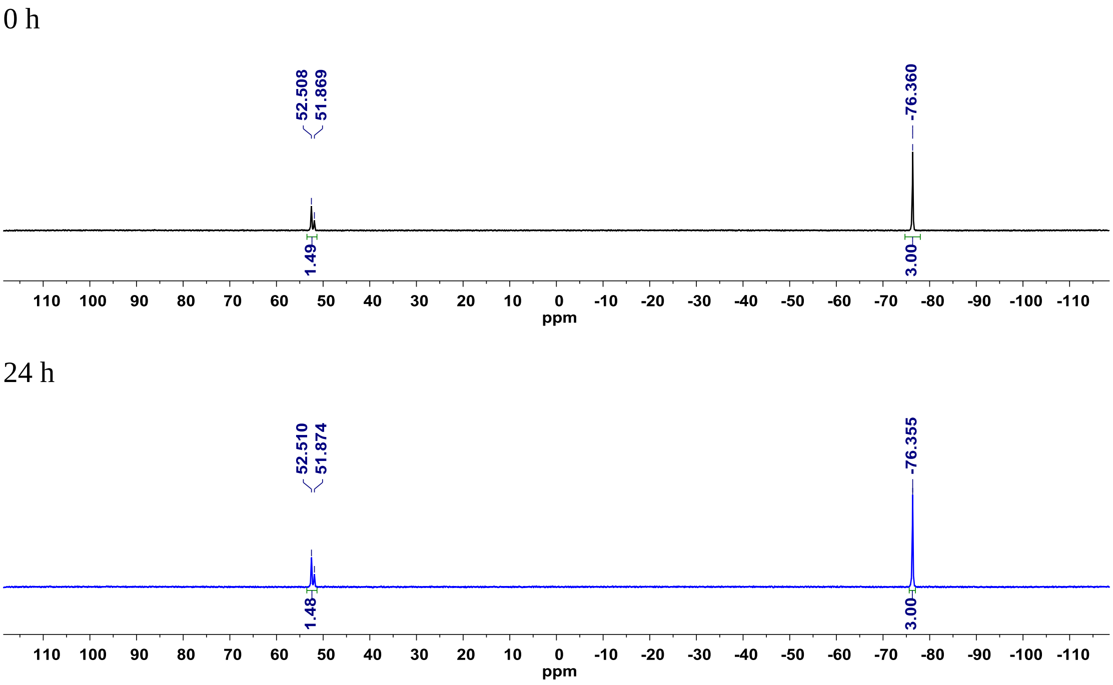

Supplementary Figure 7. ^{19}F NMR spectra for the stability test of **32**

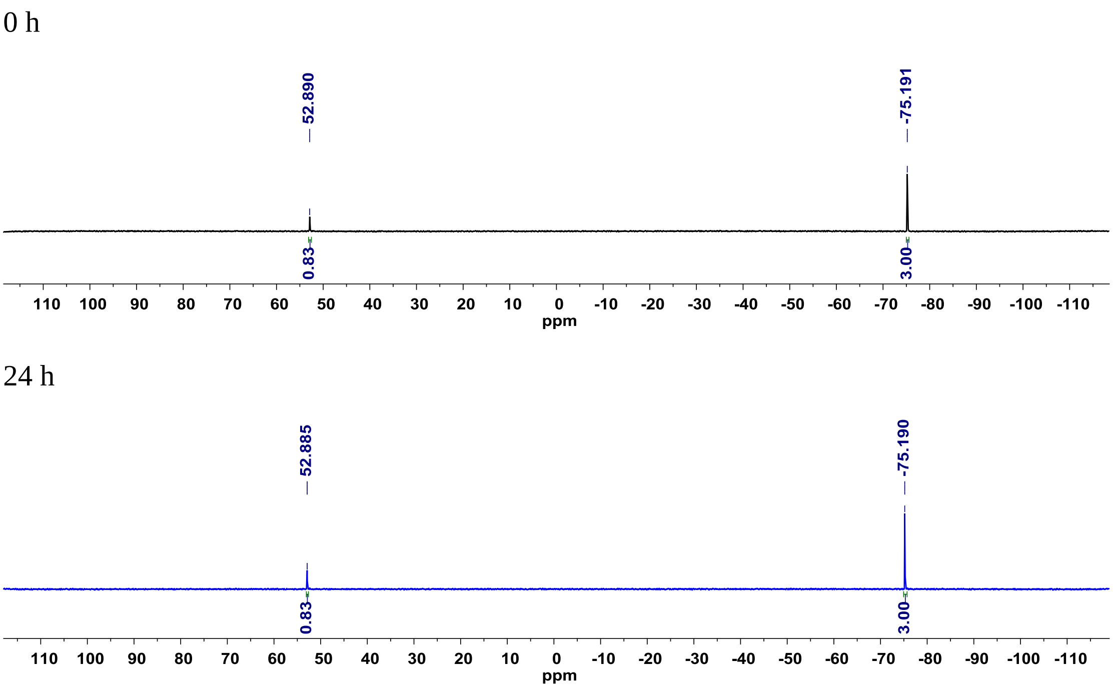

Supplementary Figure 8. ^{19}F NMR spectra for the stability test of **47**

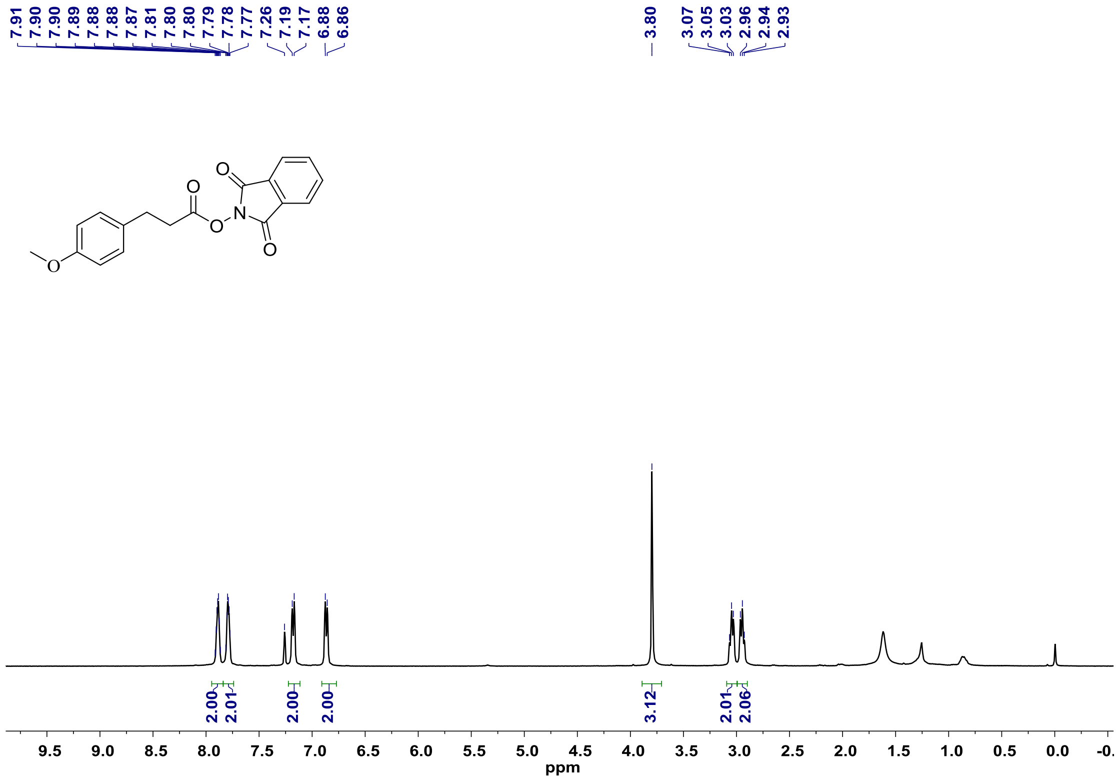

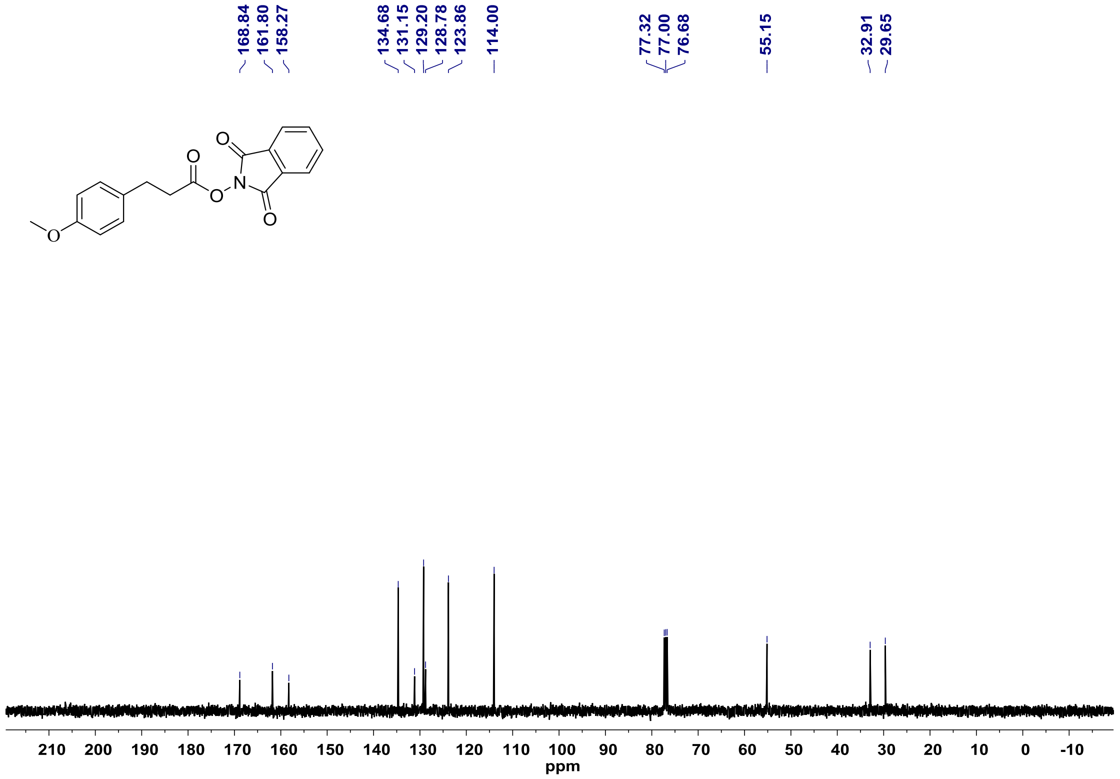

Supplementary Figure 9. ^{1}H and ^{13}C NMR spectra for compound **4s**

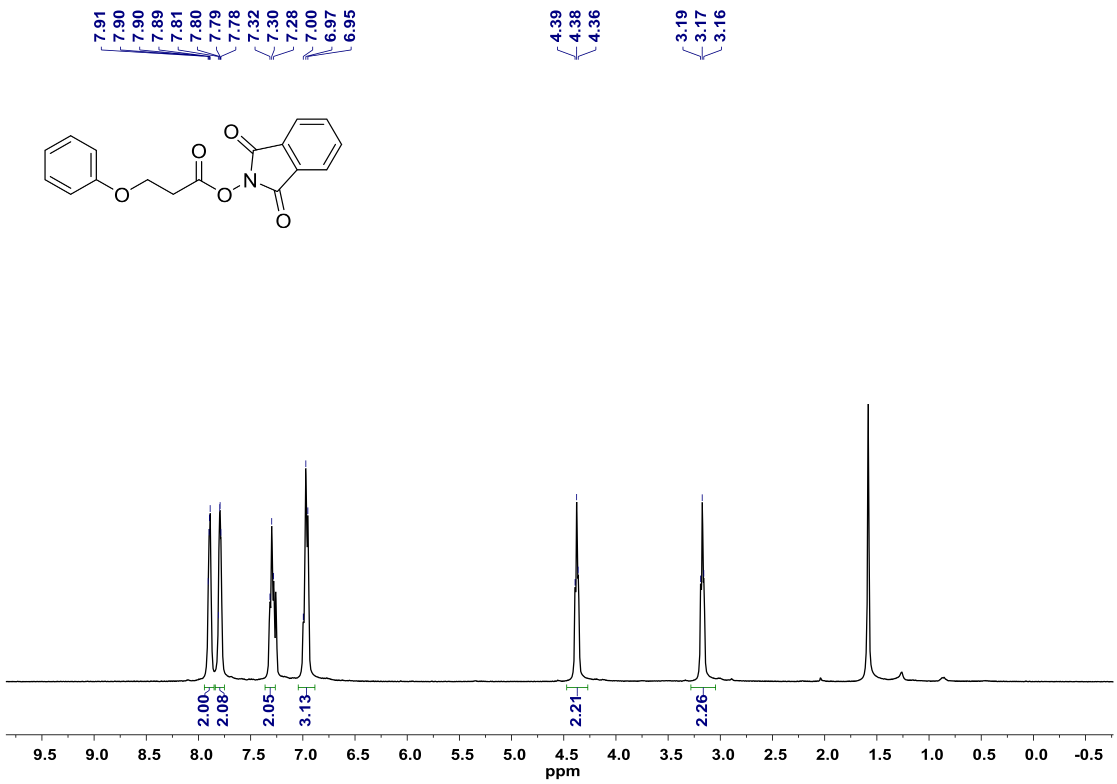

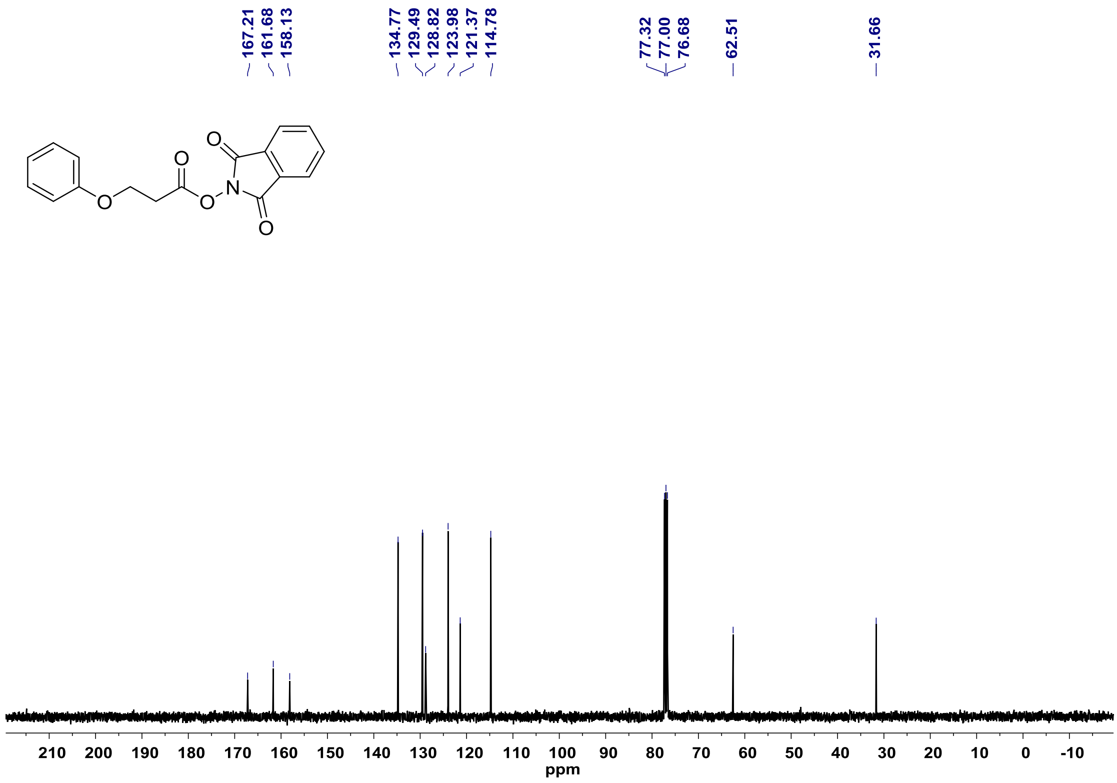

Supplementary Figure 10. ^1H and ^{13}C NMR spectra for compound **8s**

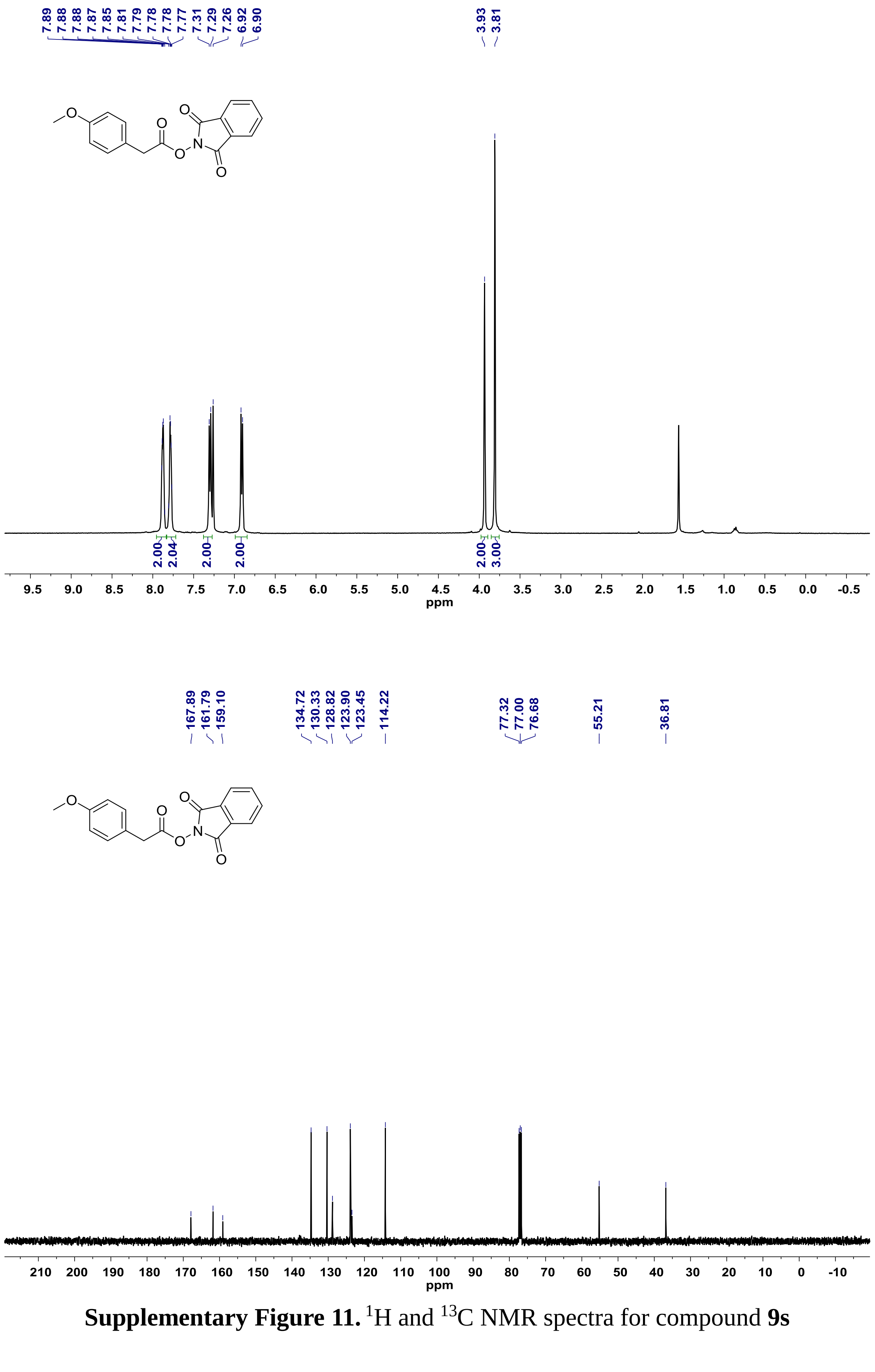

Supplementary Figure 11. ^{1}H and ^{13}C NMR spectra for compound **9s**

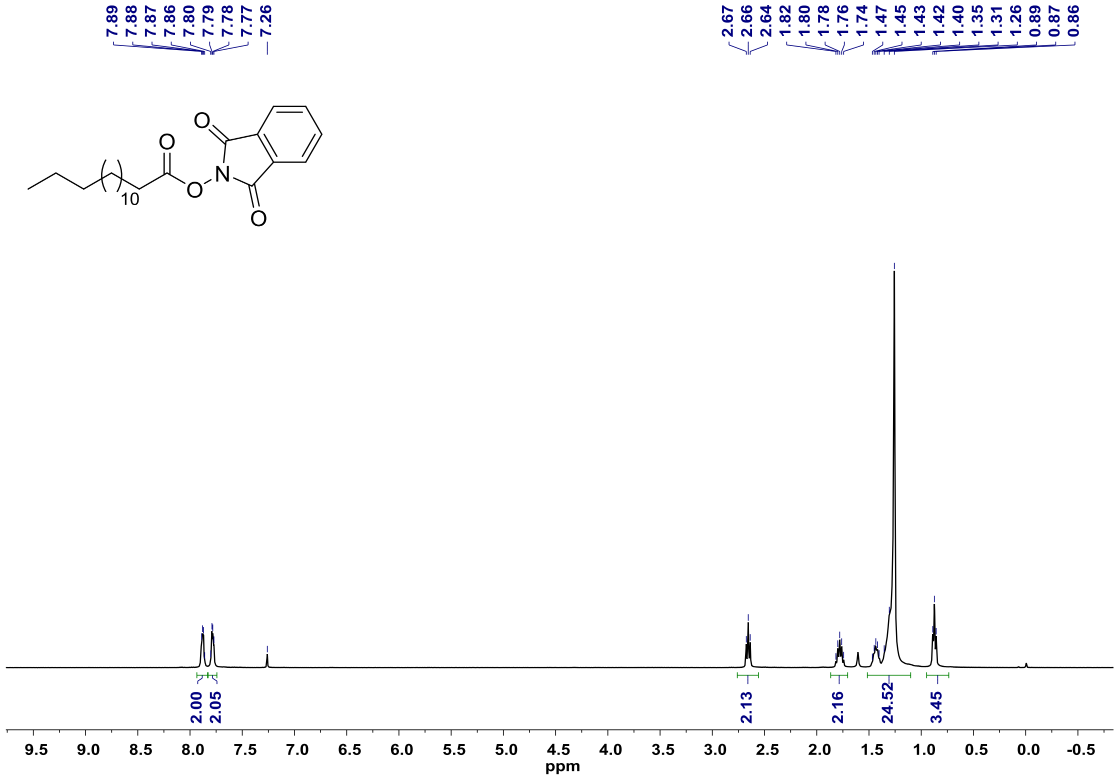

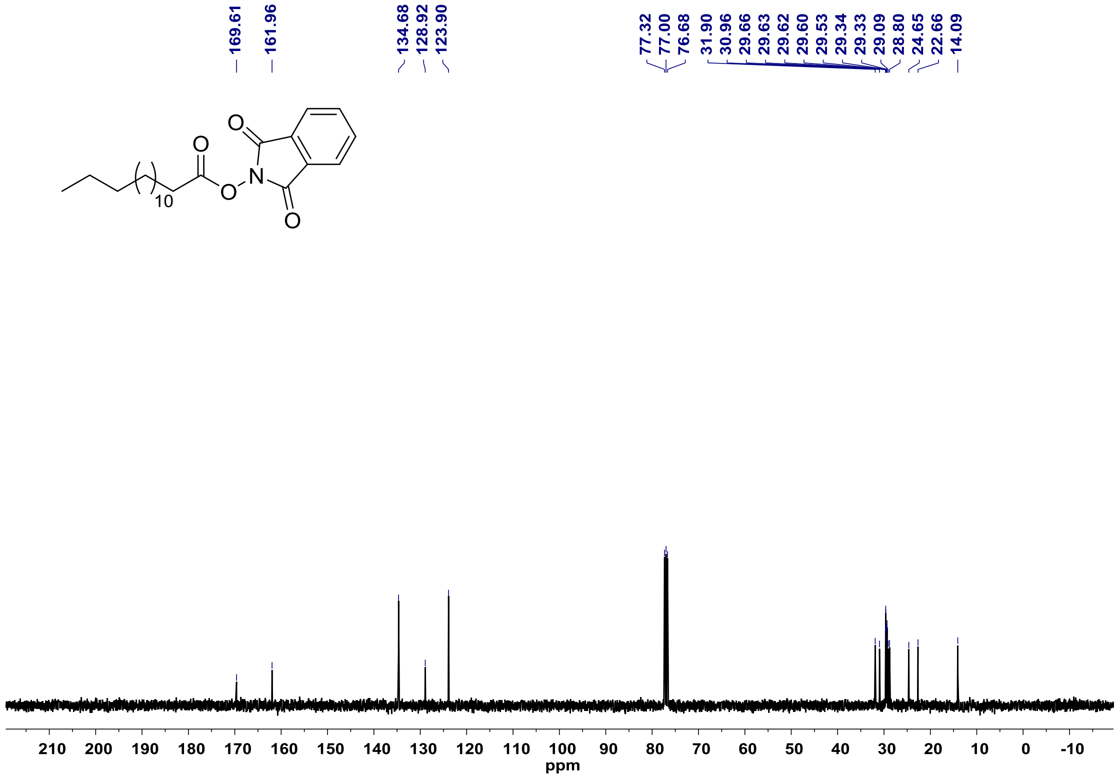

Supplementary Figure 12. ^{1}H and ^{13}C NMR spectra for compound **10s**

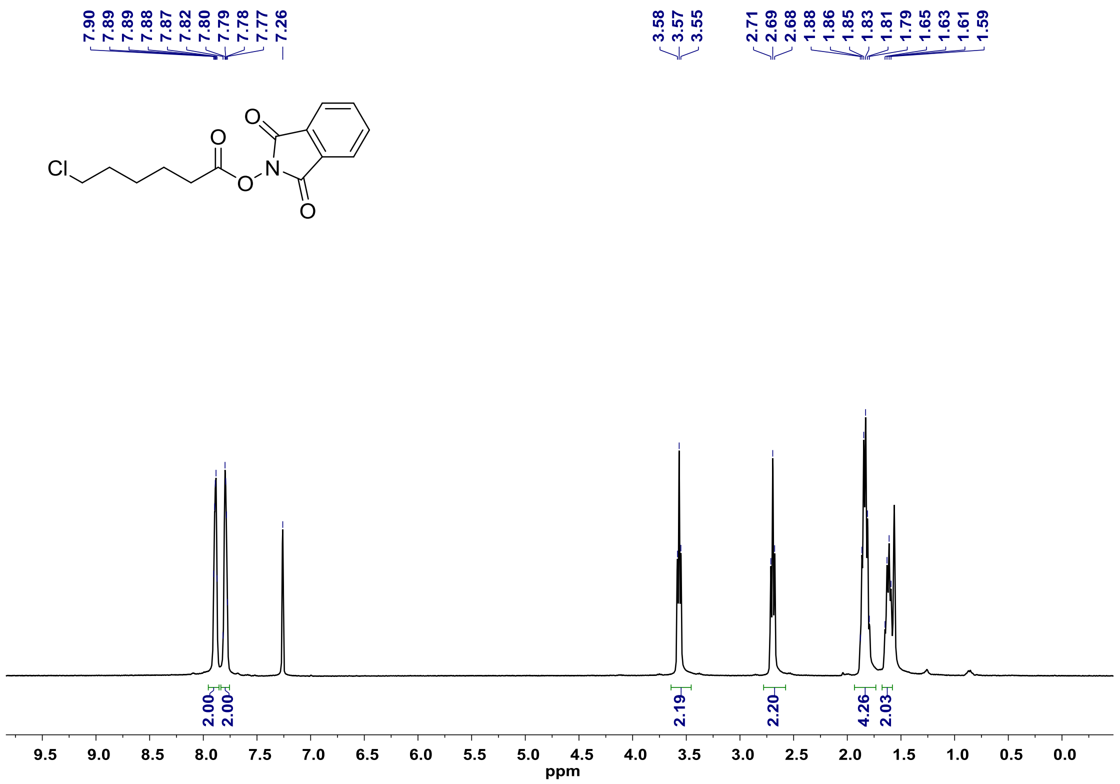

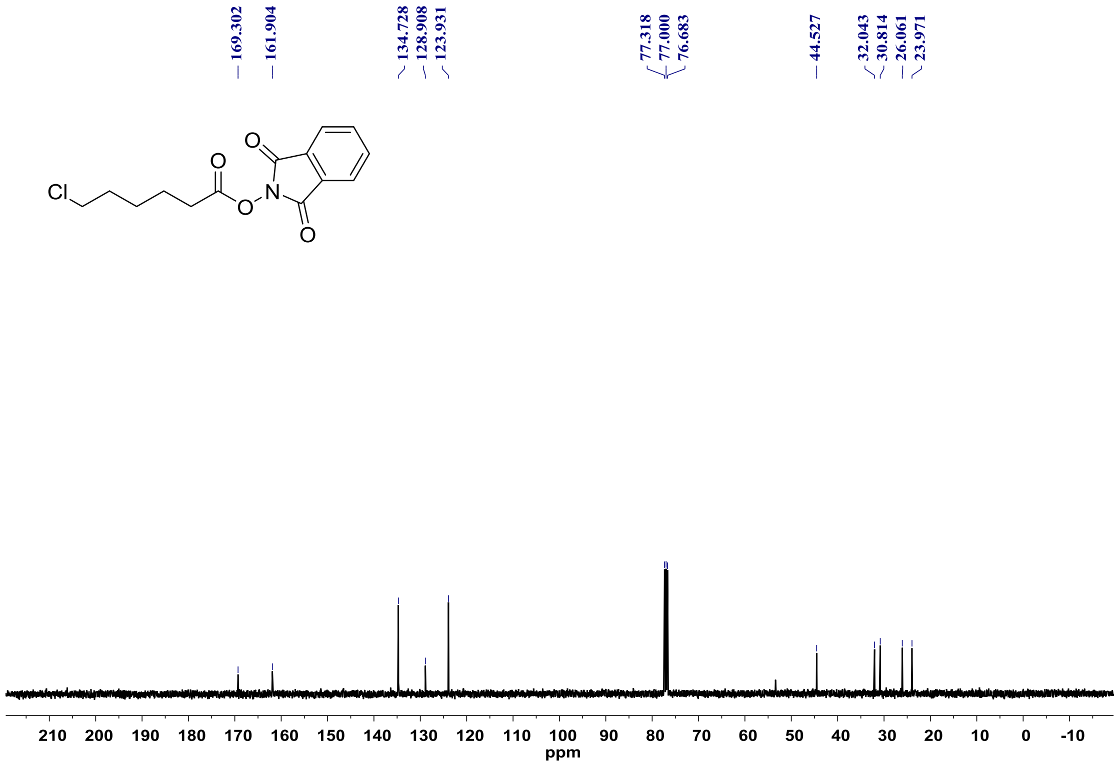

Supplementary Figure 13. ^{1}H and ^{13}C NMR spectra for compound **12s**

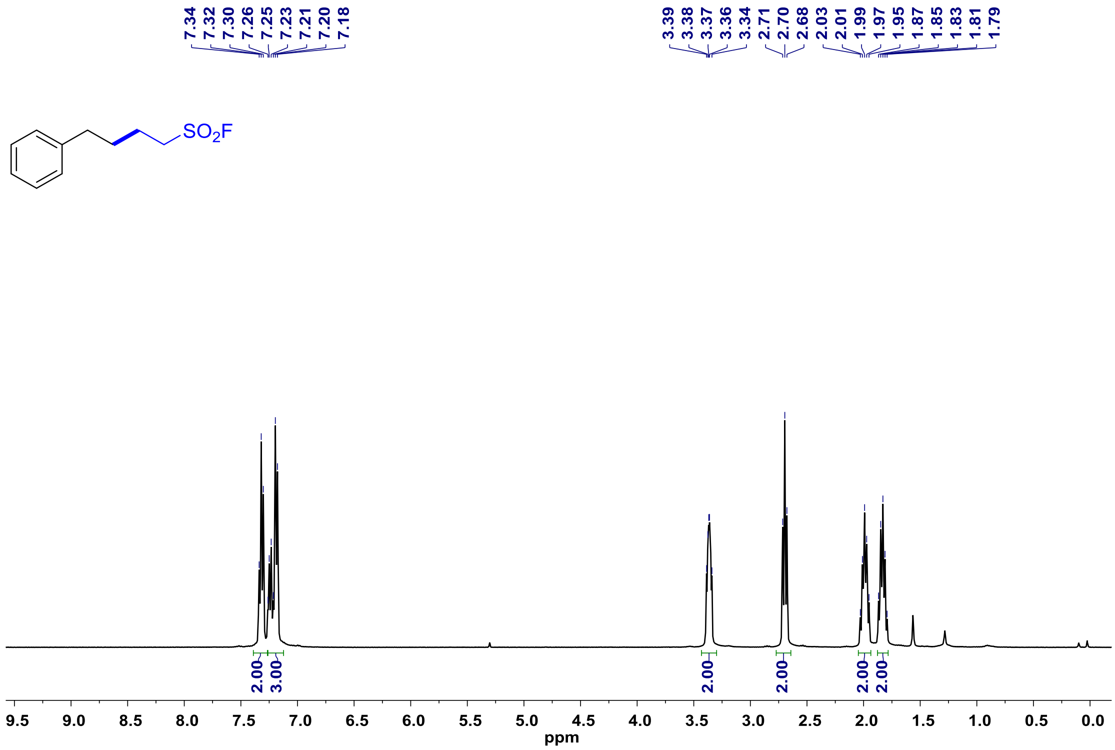

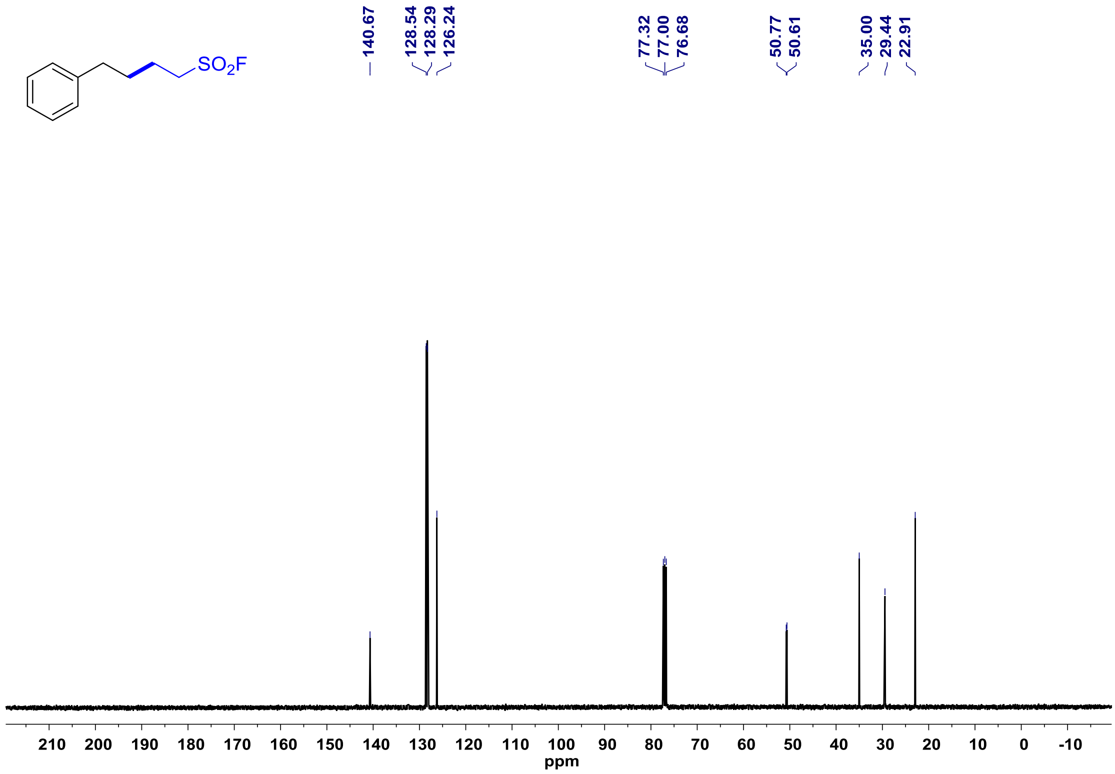

Supplementary Figure 14. ^{1}H and ^{13}C NMR spectra for compound **3**

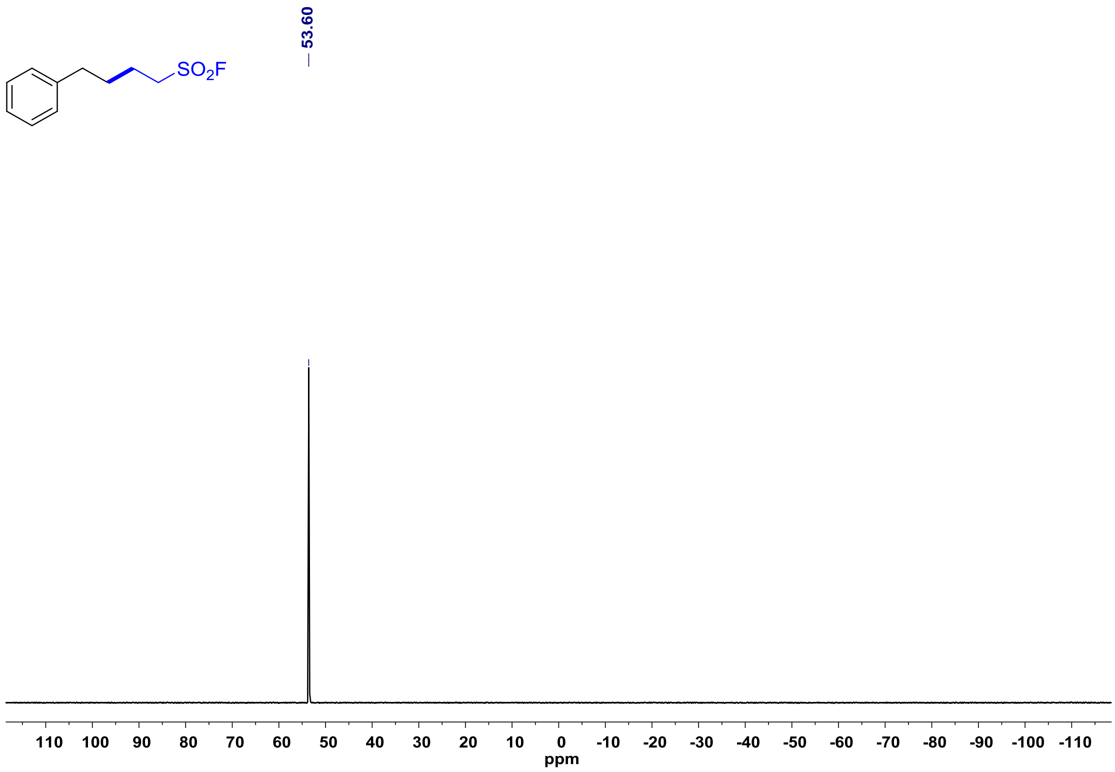

Supplementary Figure 15. ^{19}F NMR spectrum for compound **3**

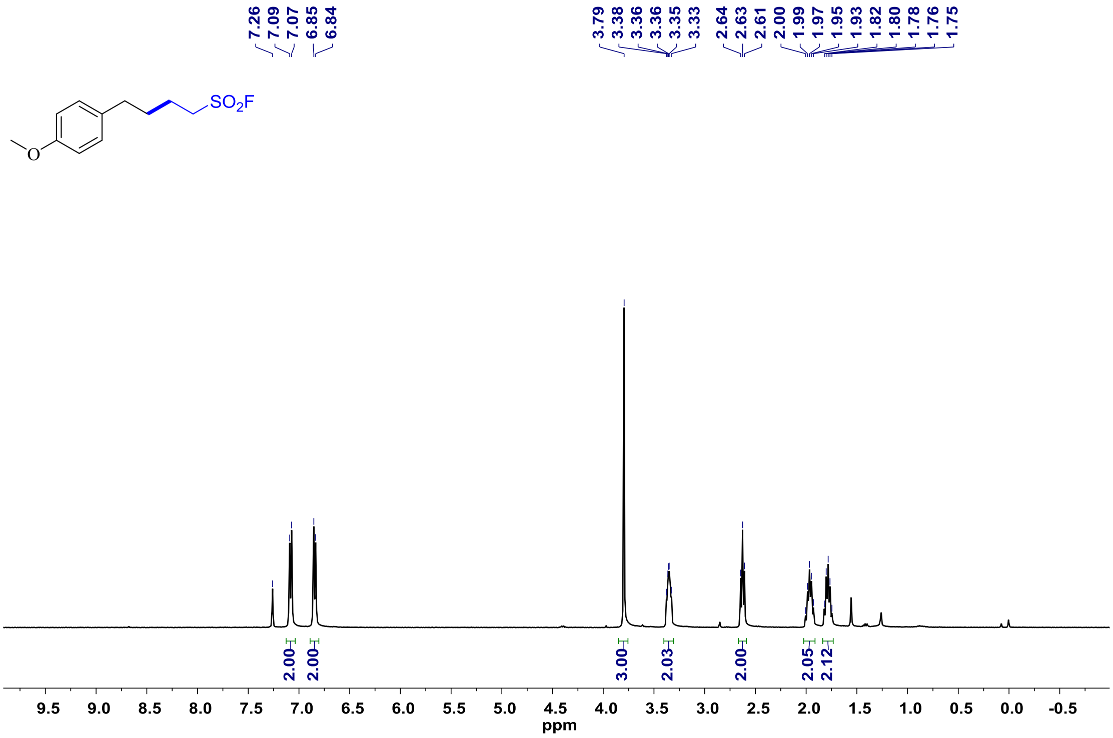

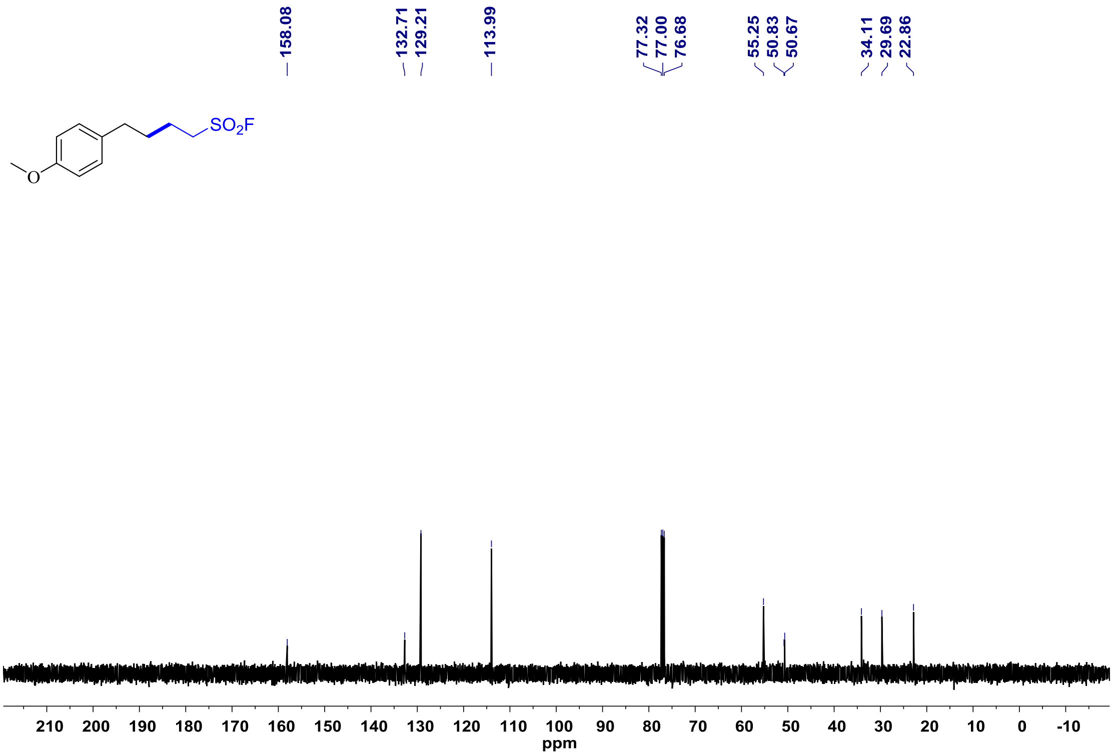

Supplementary Figure 16. ^{1}H and ^{13}C NMR spectra for compound **4**

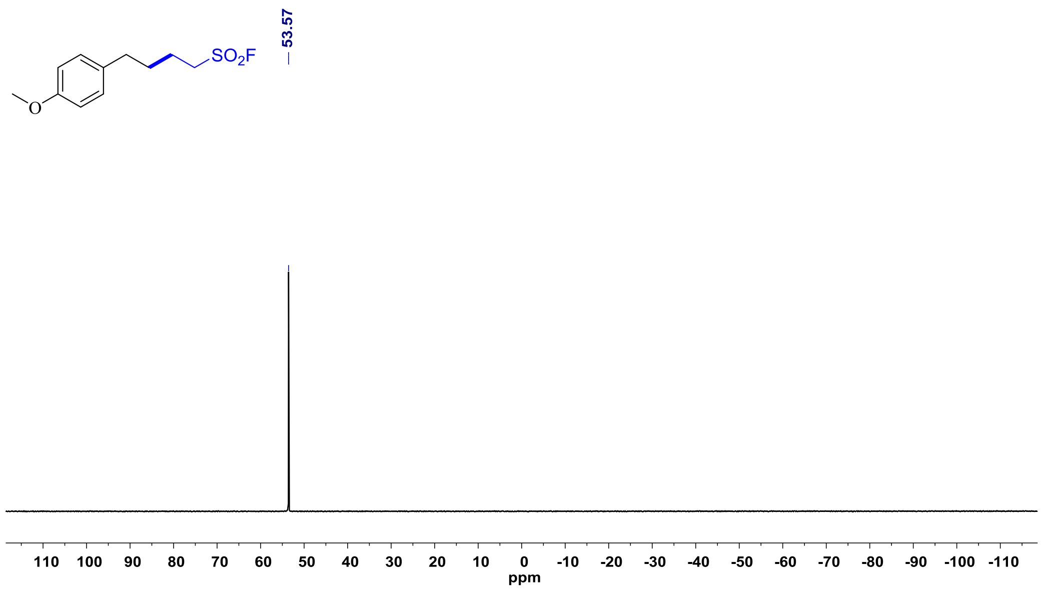

Supplementary Figure 17. ^{19}F NMR spectrum for compound **4**

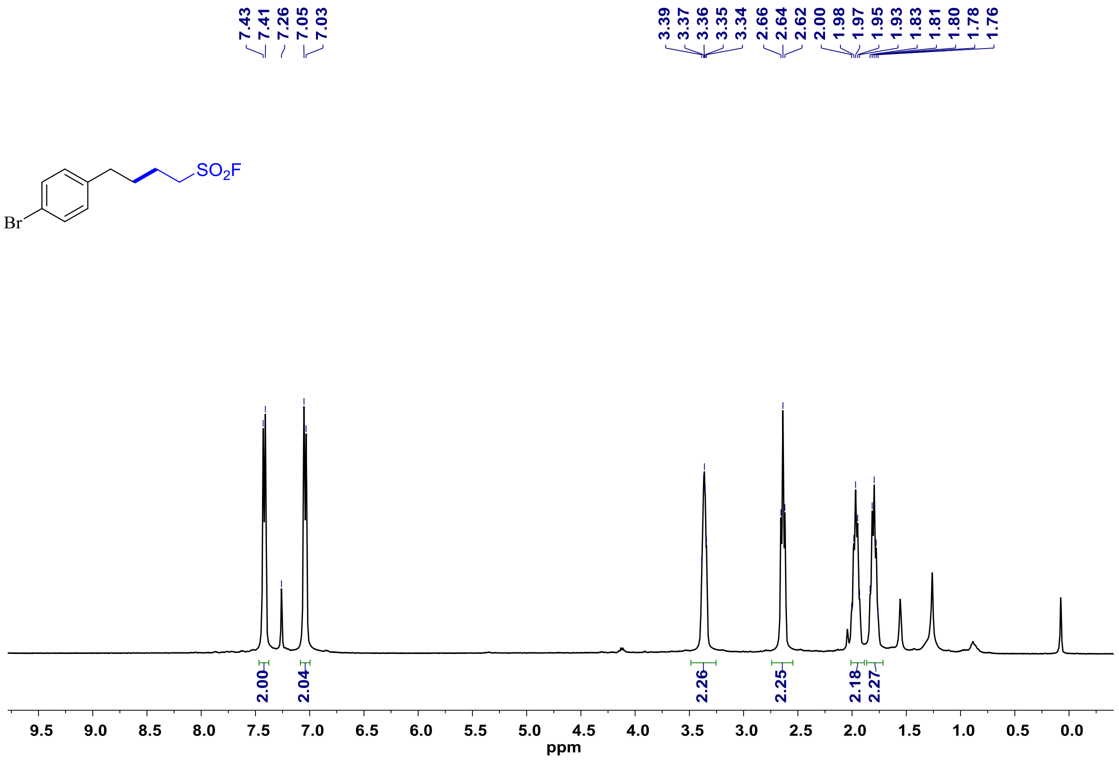

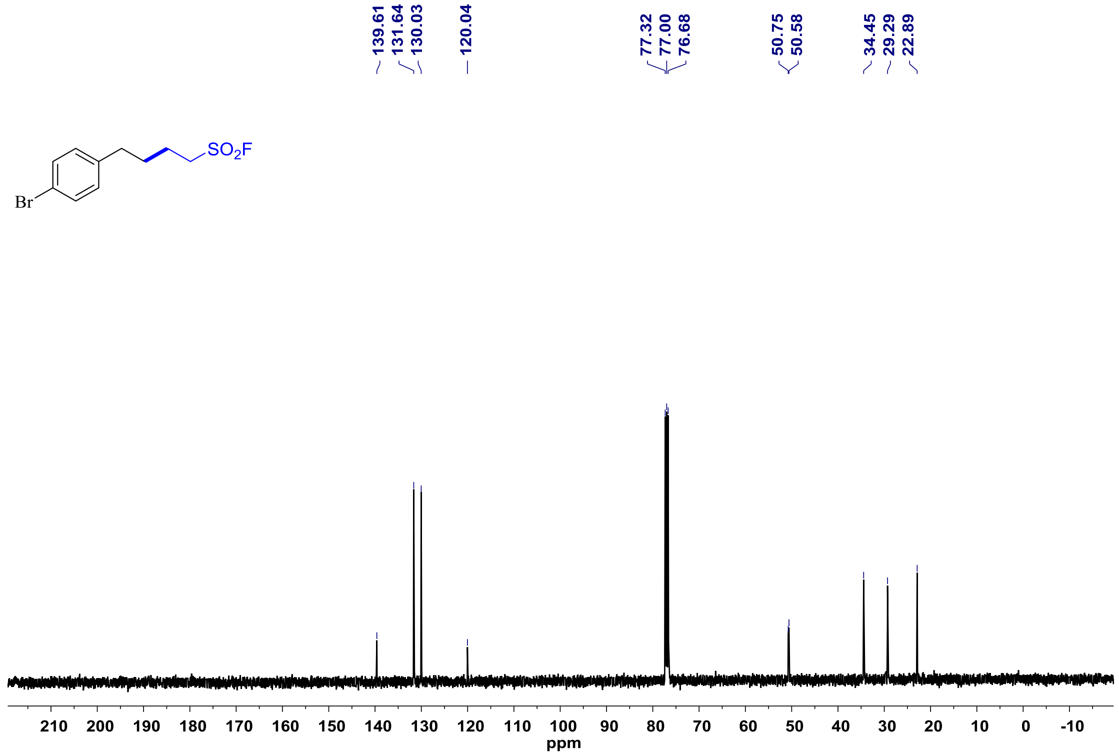

Supplementary Figure 18. ^{1}H and ^{13}C NMR spectra for compound **5**

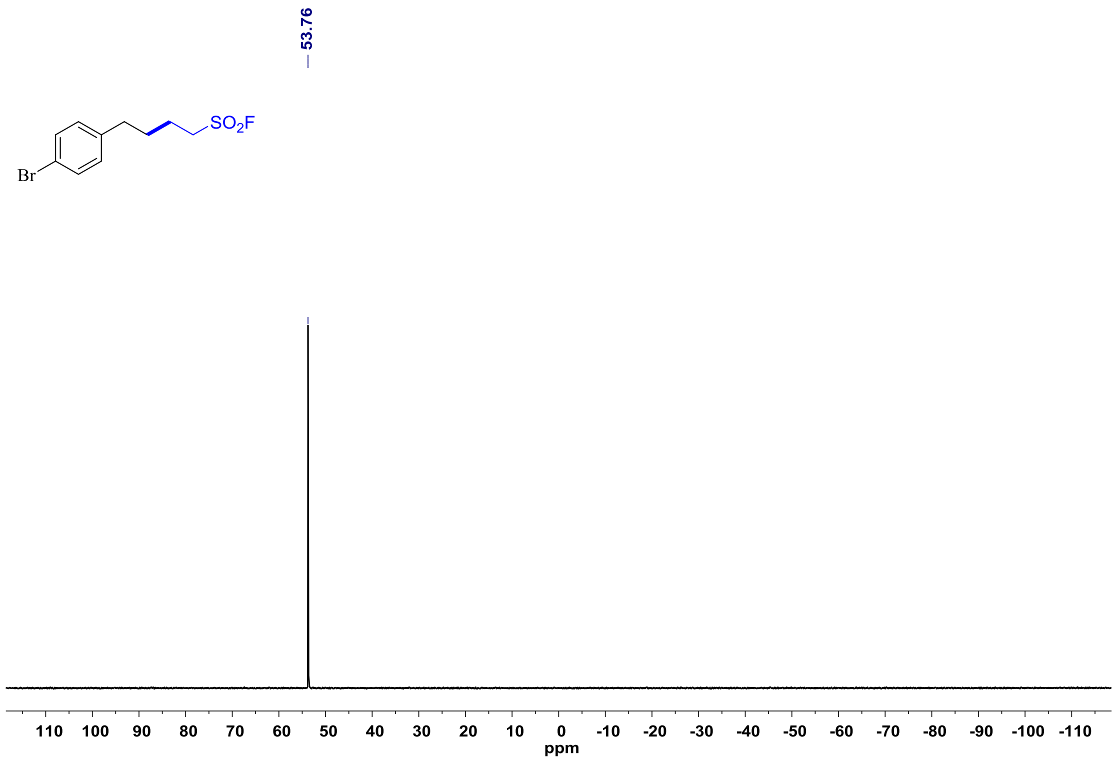

Supplementary Figure 19. ^{19}F NMR spectrum for compound **5**

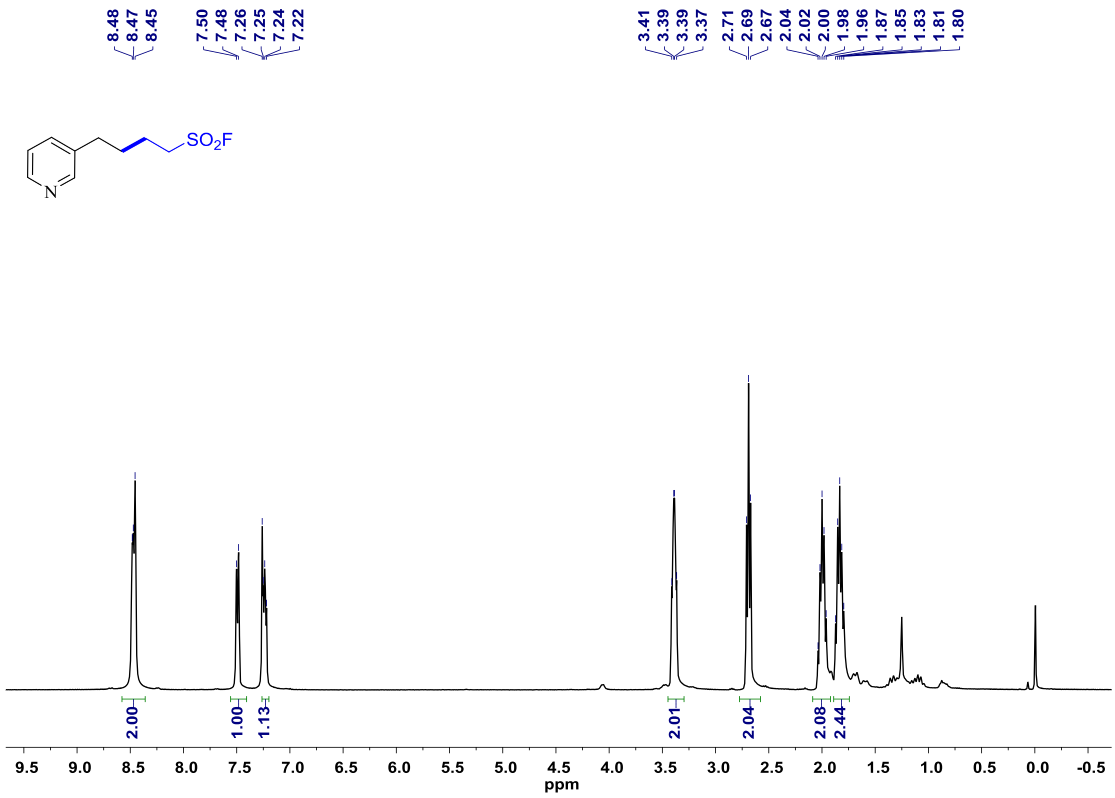

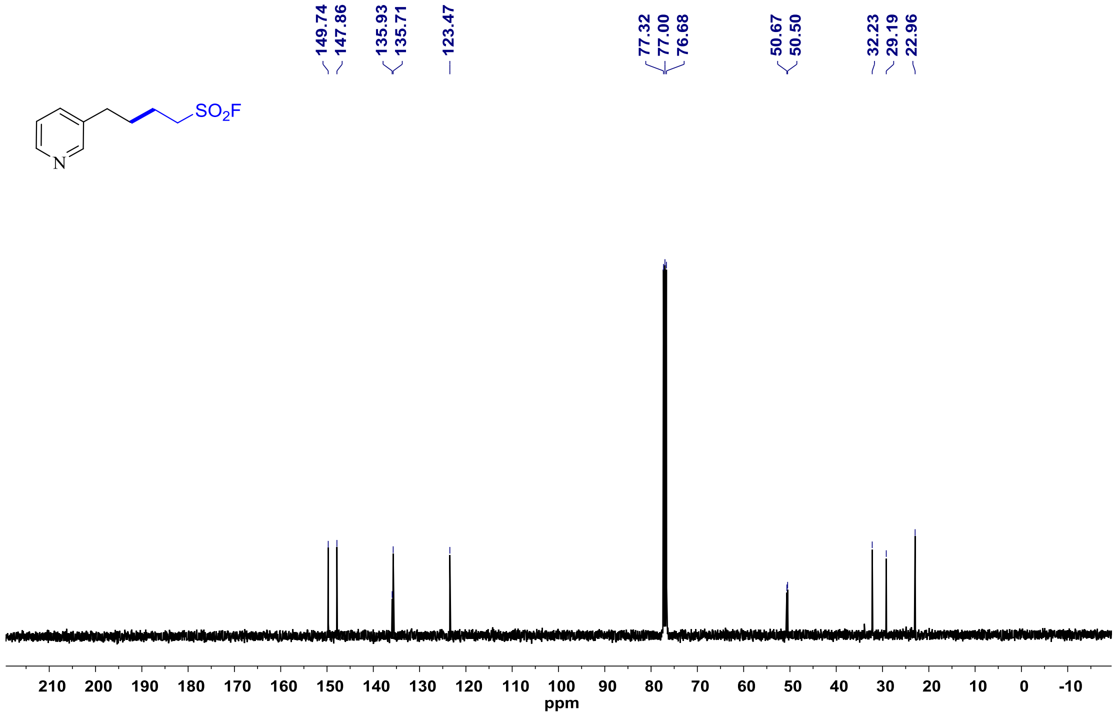

Supplementary Figure 20. ^{1}H and ^{13}C NMR spectra for compound **6**

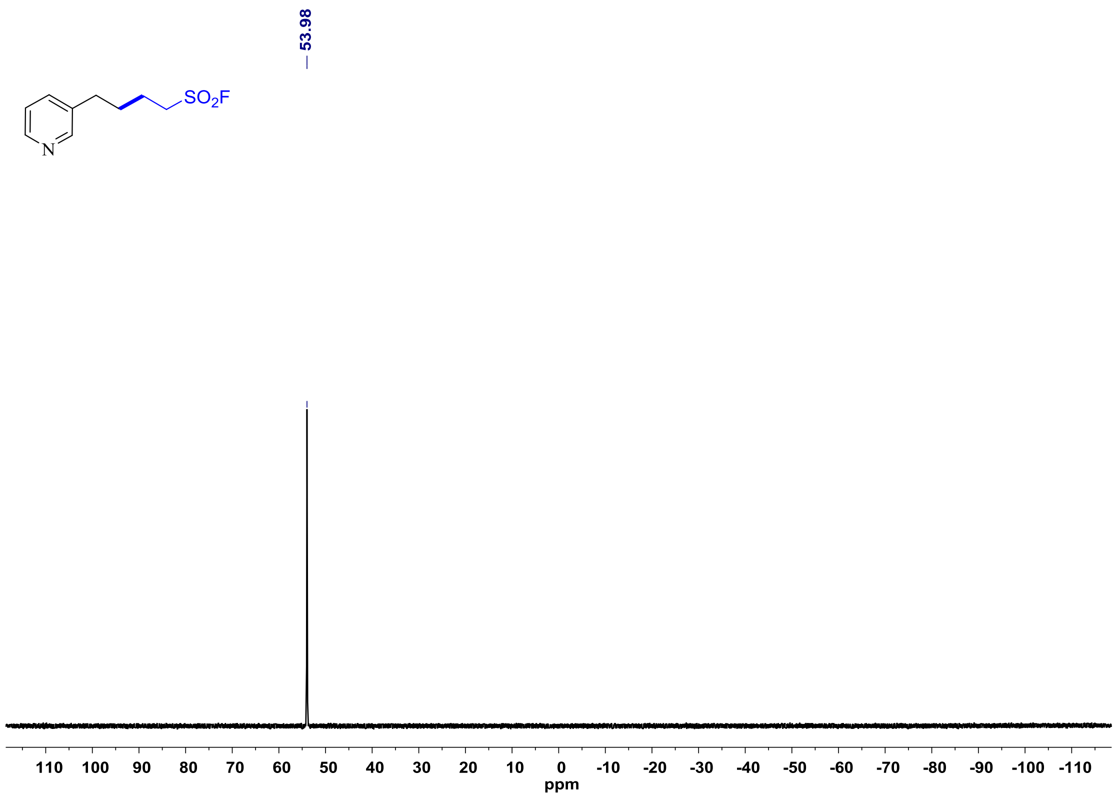

Supplementary Figure 21. ^{19}F NMR spectrum for compound **6**

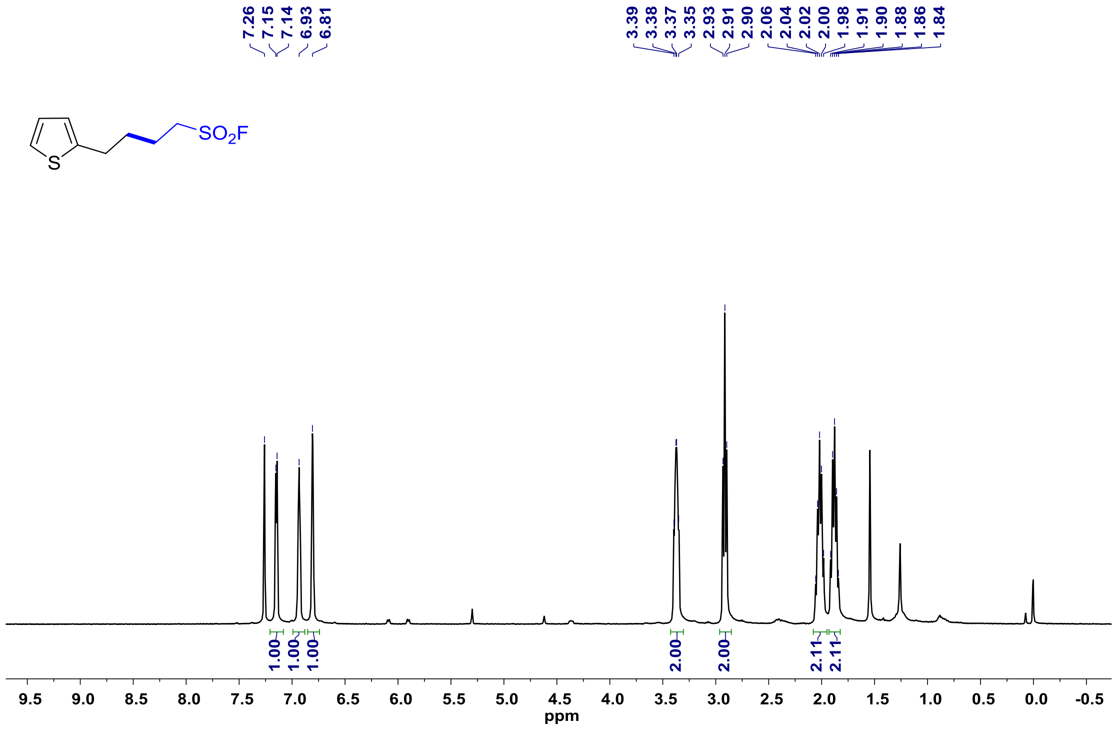

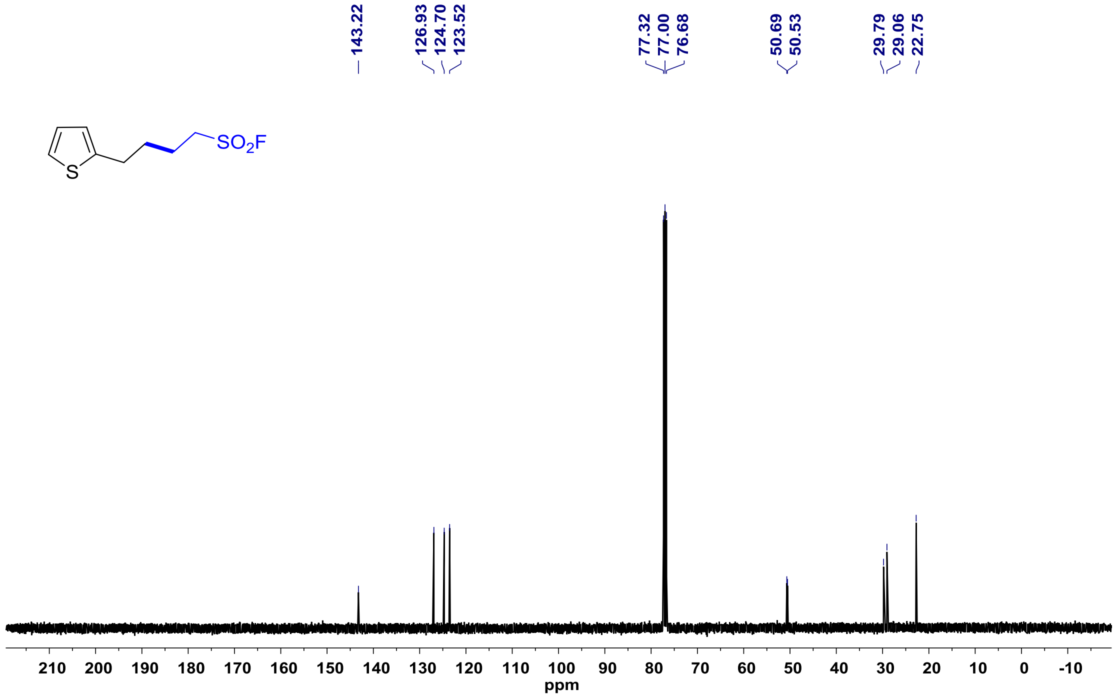

Supplementary Figure 22. ^{1}H and ^{13}C NMR spectra for compound **7**

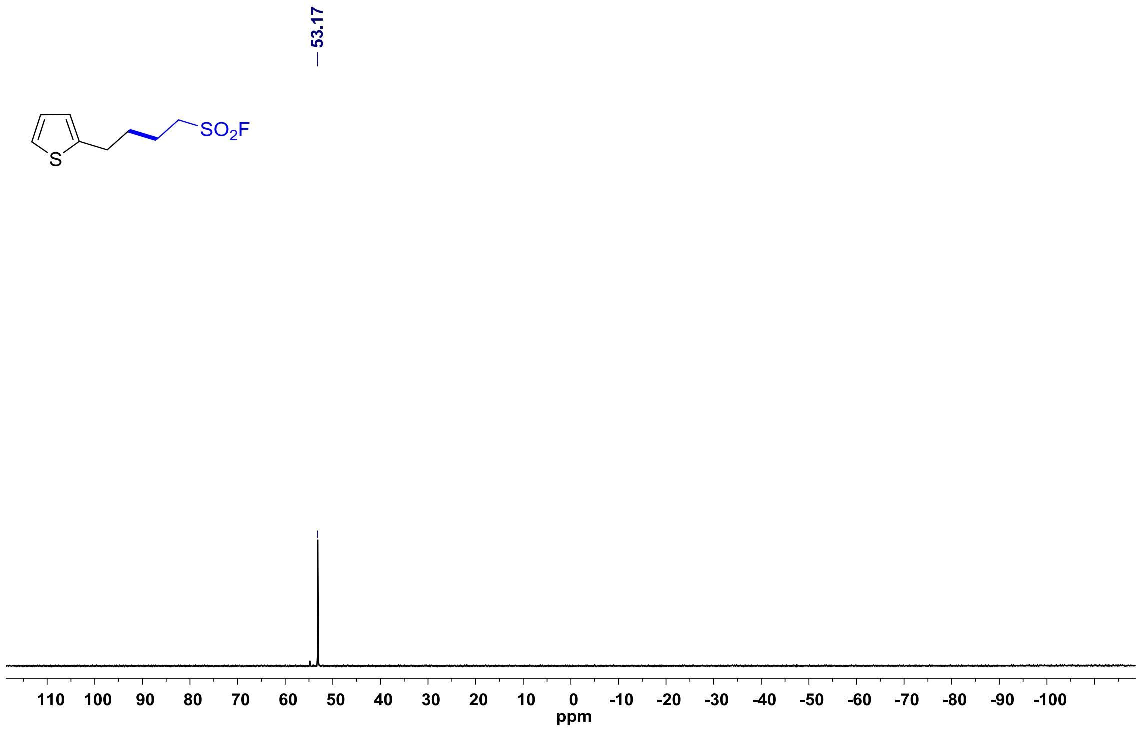

Supplementary Figure 23. ^{19}F NMR spectrum for compound **7**

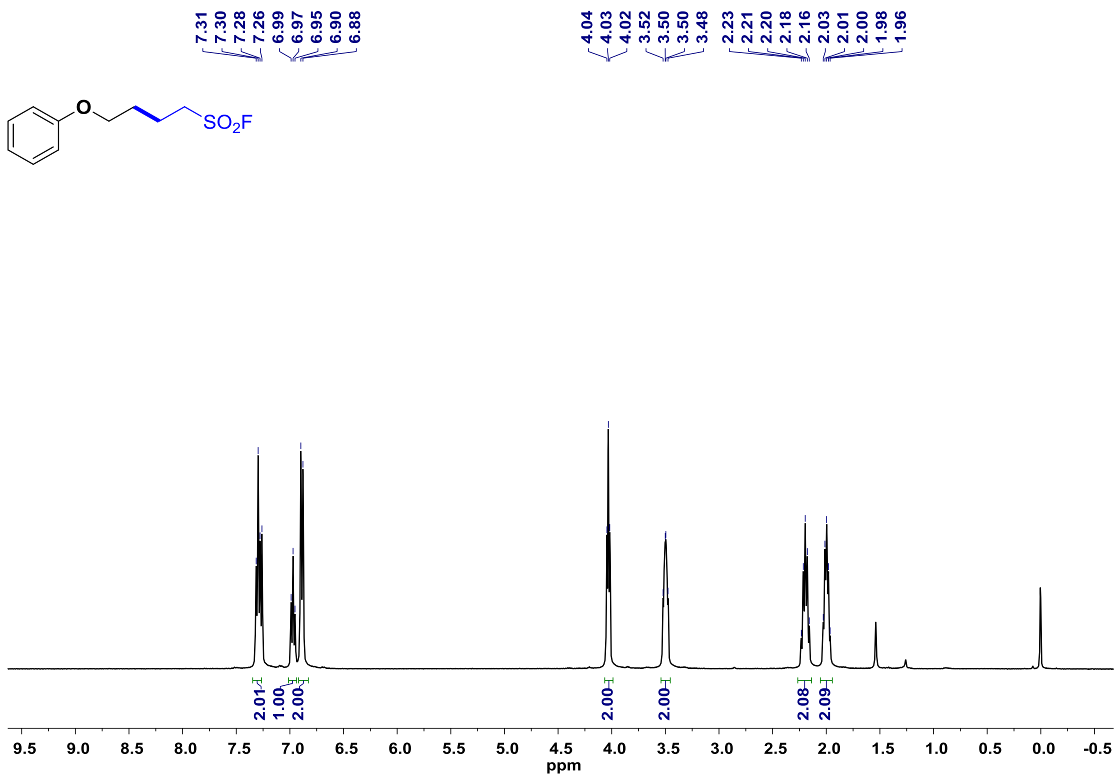

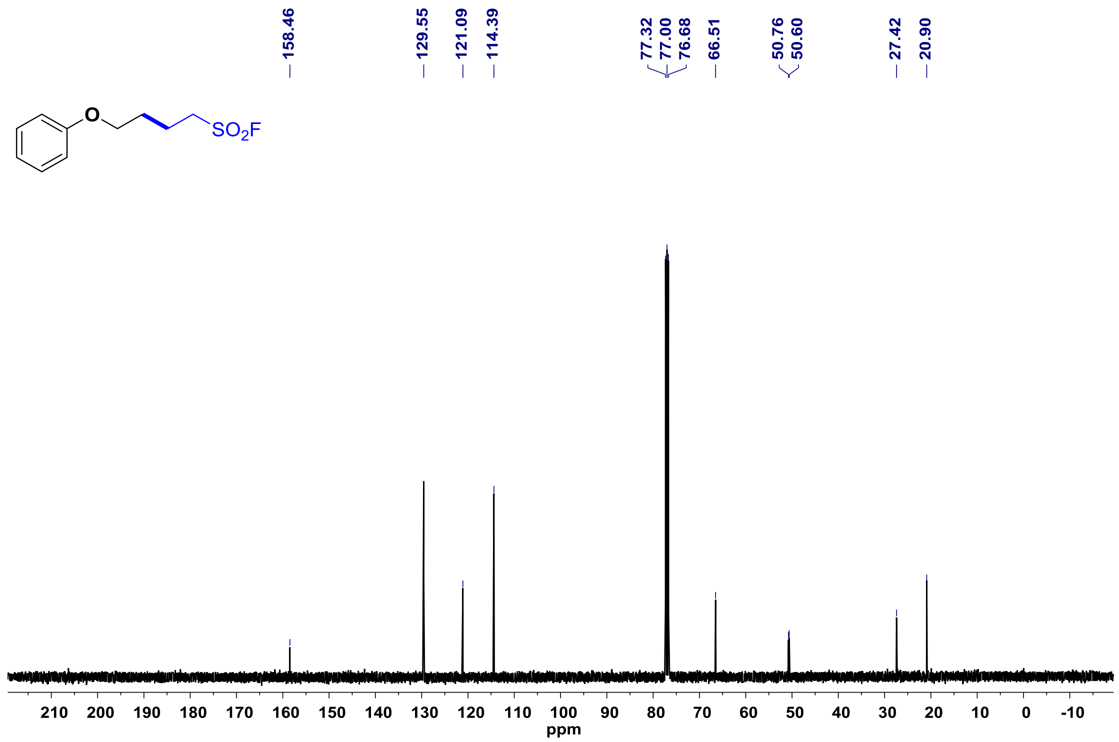

Supplementary Figure 24. ^{1}H and ^{13}C NMR spectra for compound **8**

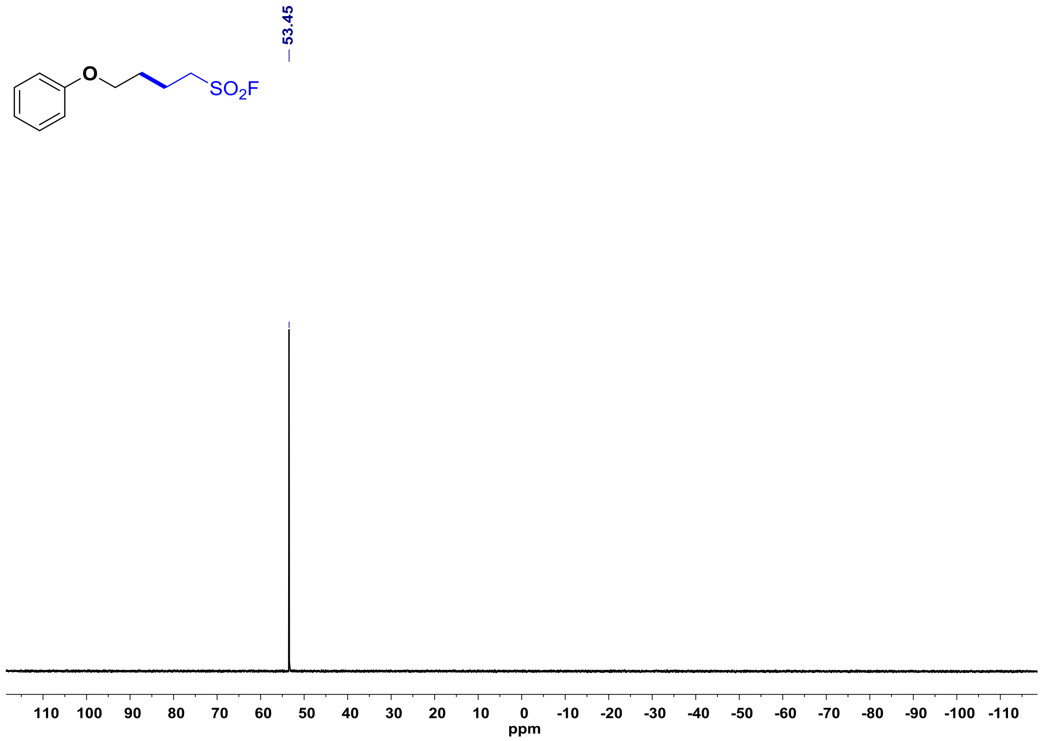

Supplementary Figure 25. ^{19}F NMR spectrum for compound **8**

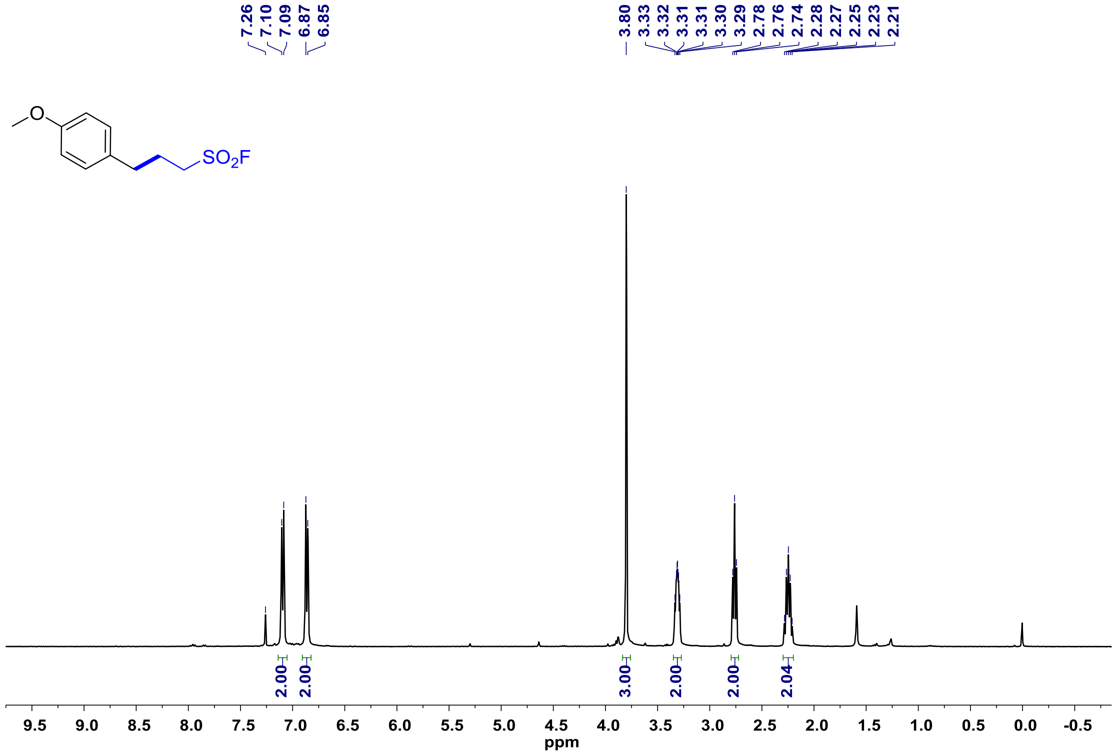

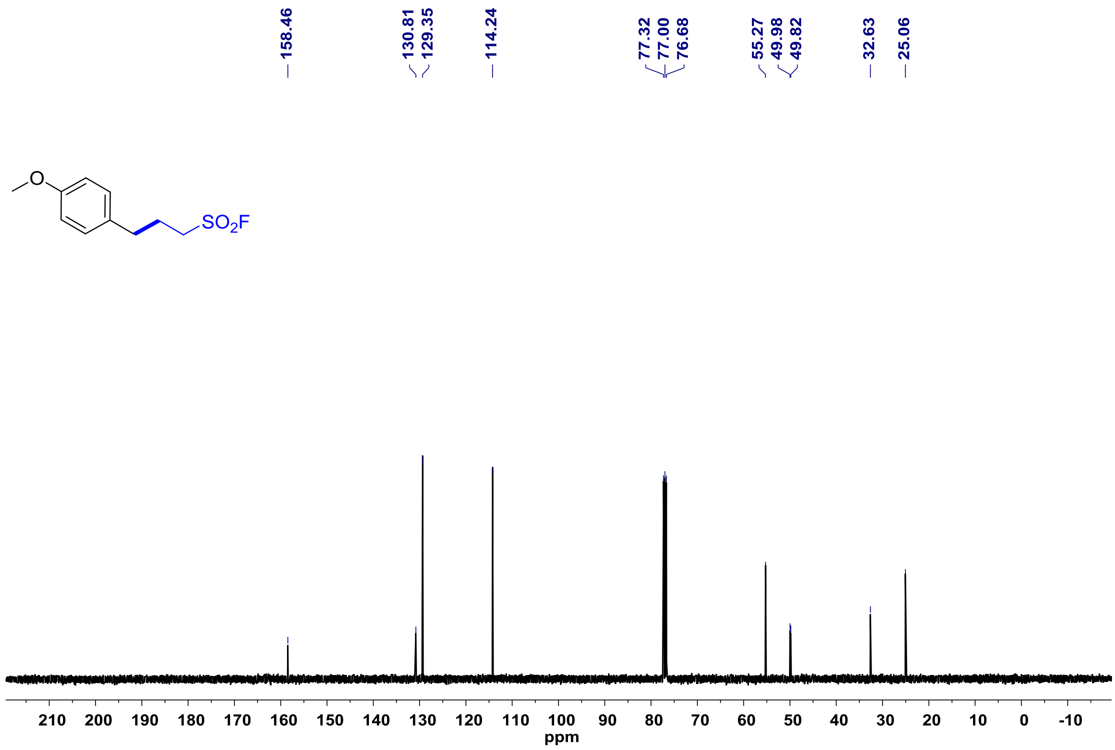

Supplementary Figure 26. ^{1}H and ^{13}C NMR spectra for compound **9**

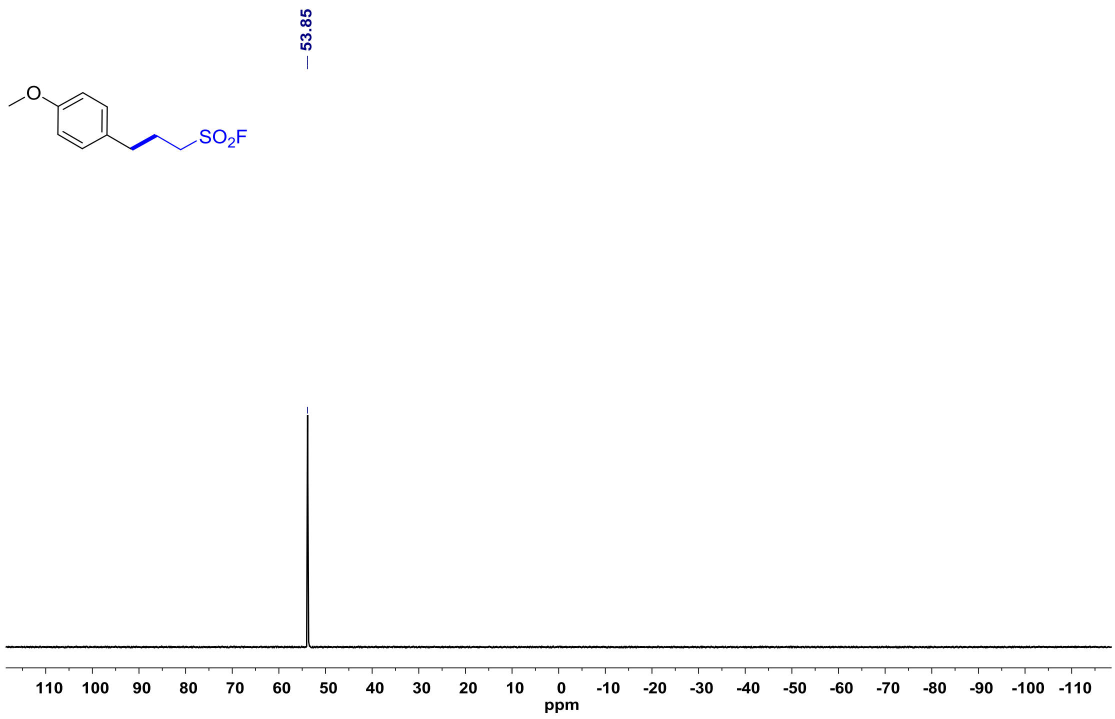

Supplementary Figure 27. ^{19}F NMR spectrum for compound **9**

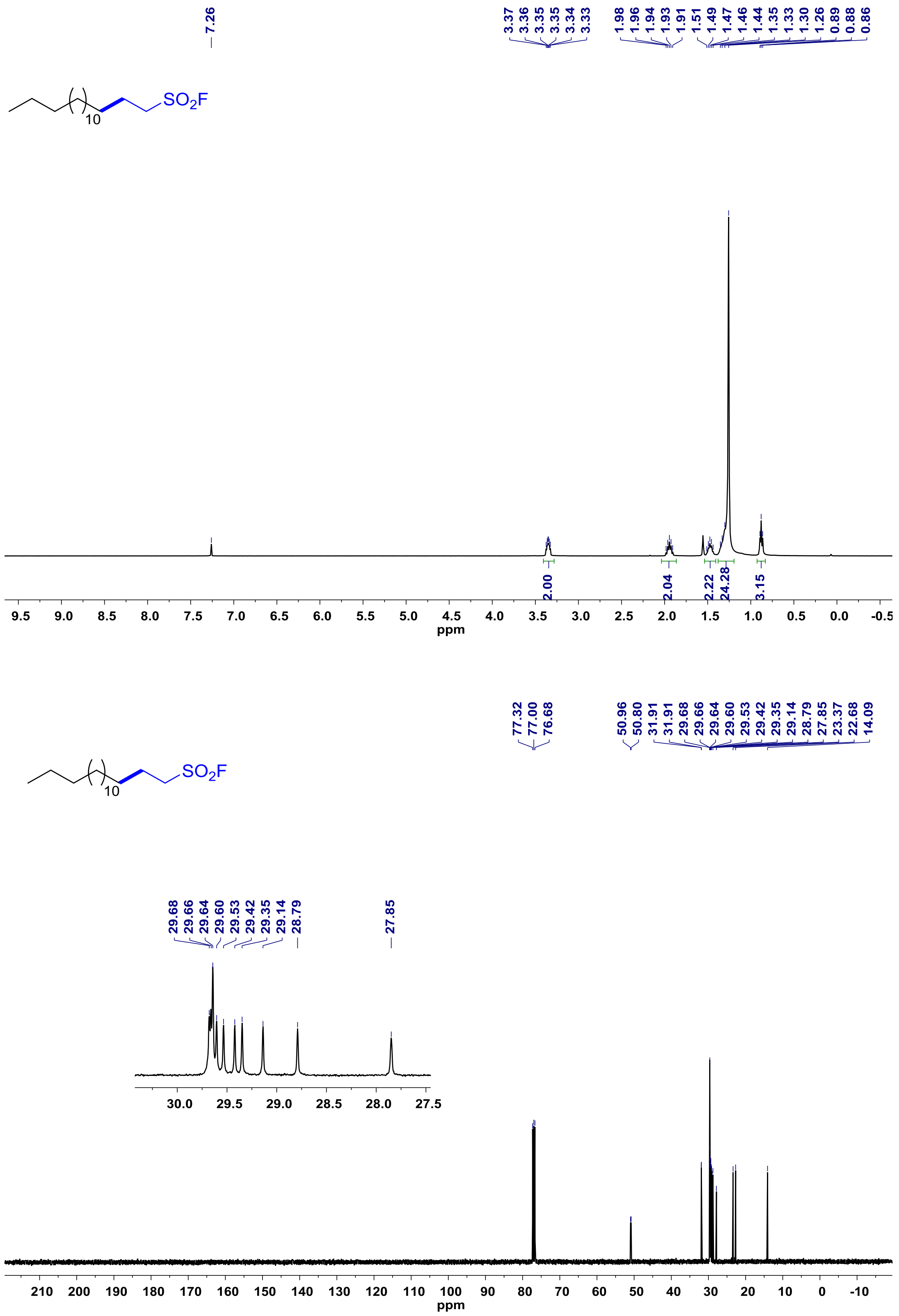

Supplementary Figure 28. ^{1}H and ^{13}C NMR spectra for compound **10**

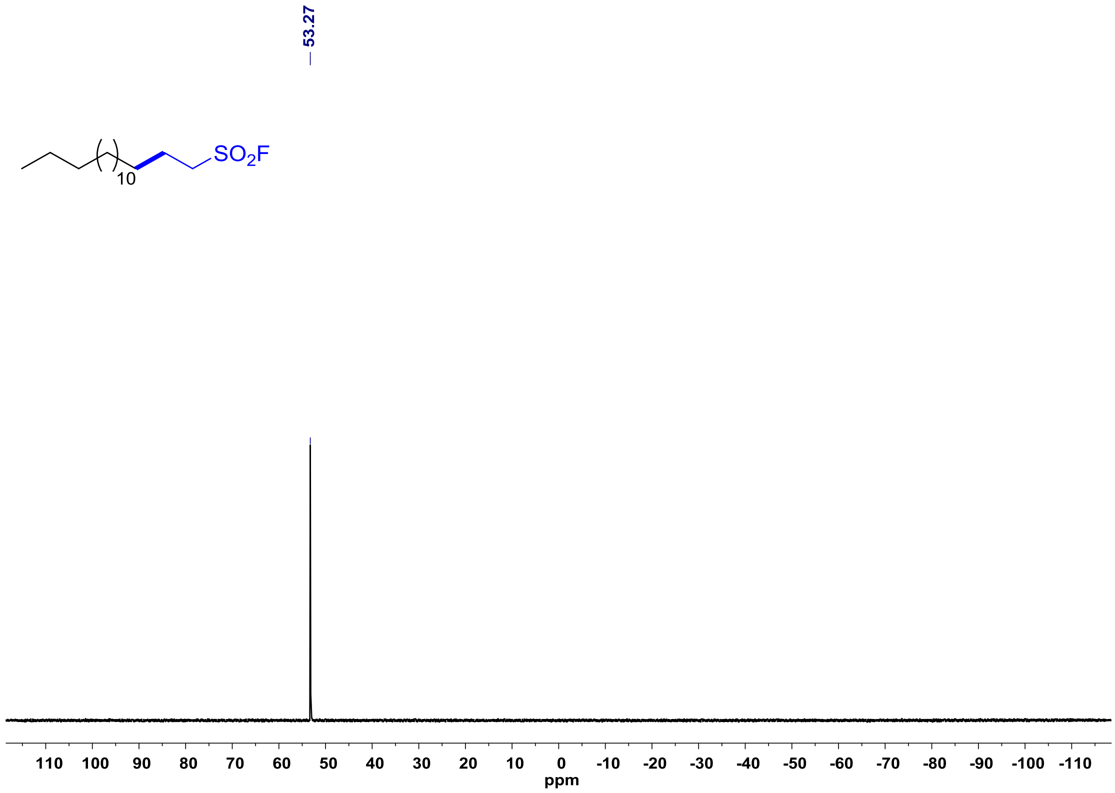

Supplementary Figure 29. ^{19}F NMR spectrum for compound **10**

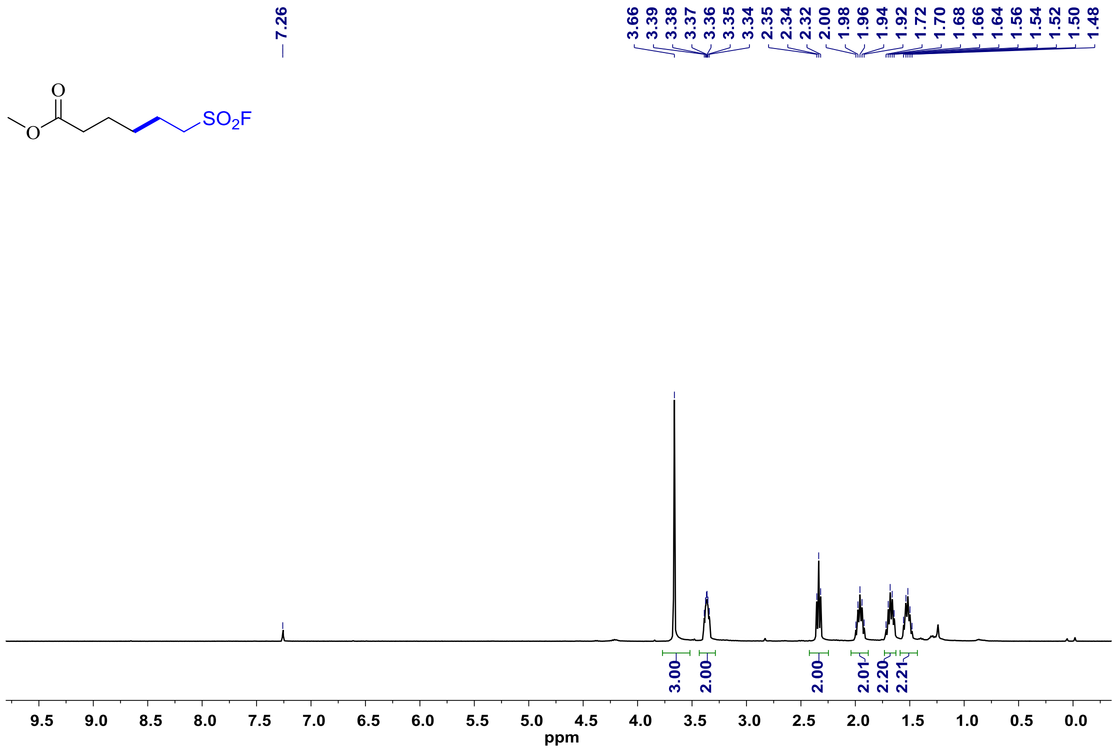

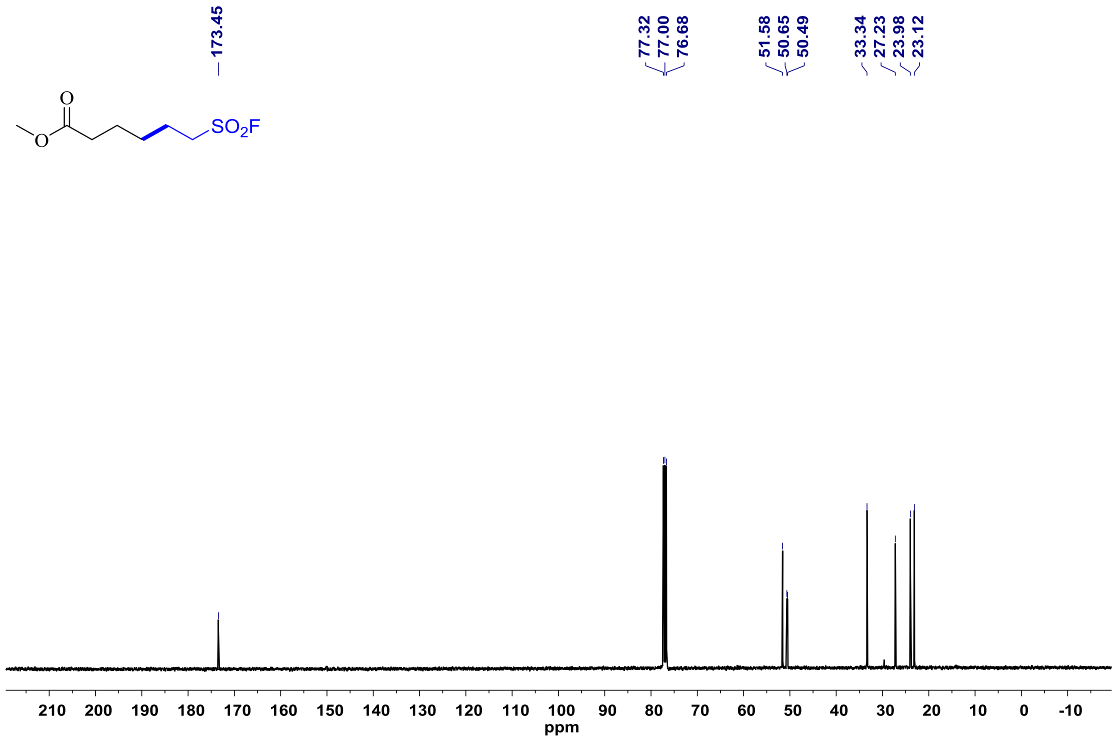

Supplementary Figure 30. ^{1}H and ^{13}C NMR spectra for compound **11**

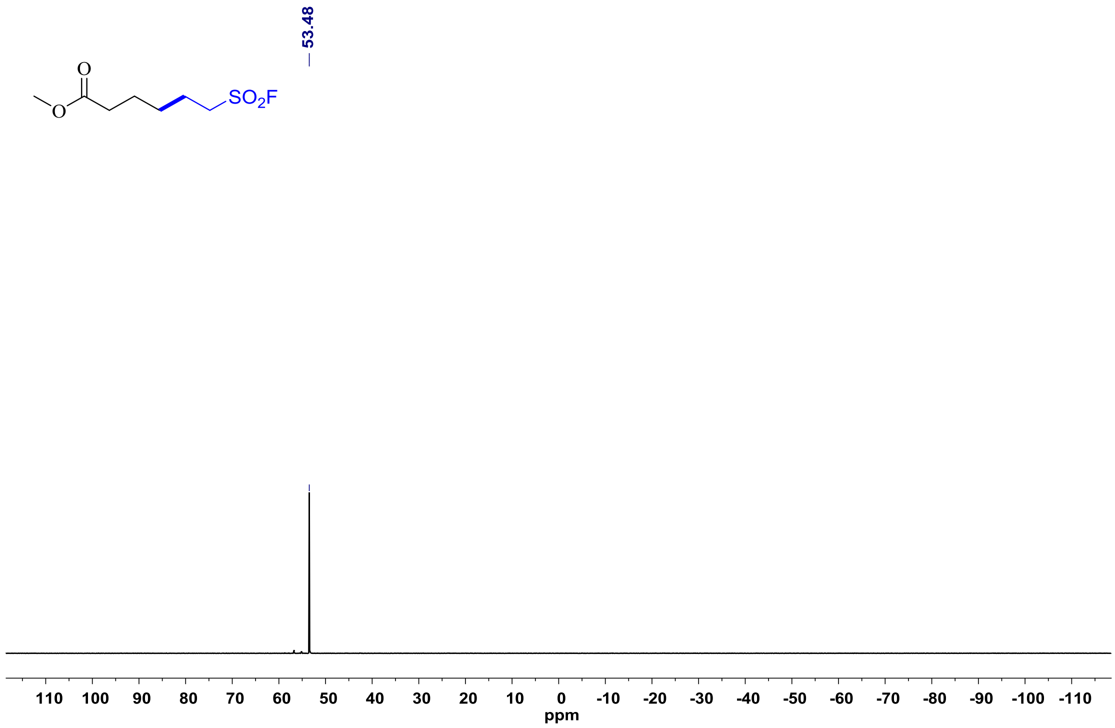

Supplementary Figure 31. ^{19}F NMR spectrum for compound **11**

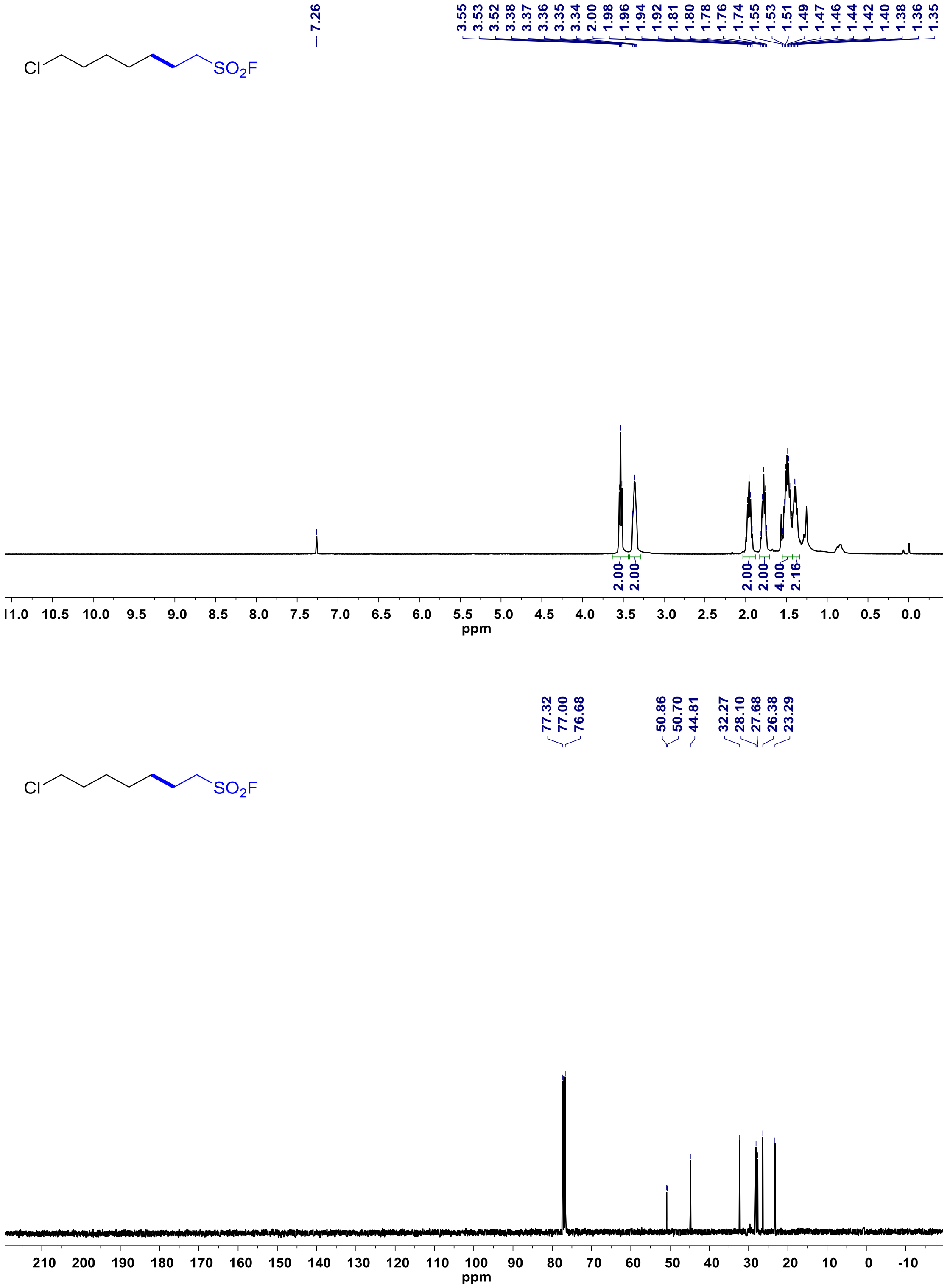

Supplementary Figure 32. ^{1}H and ^{13}C NMR spectra for compound **12**

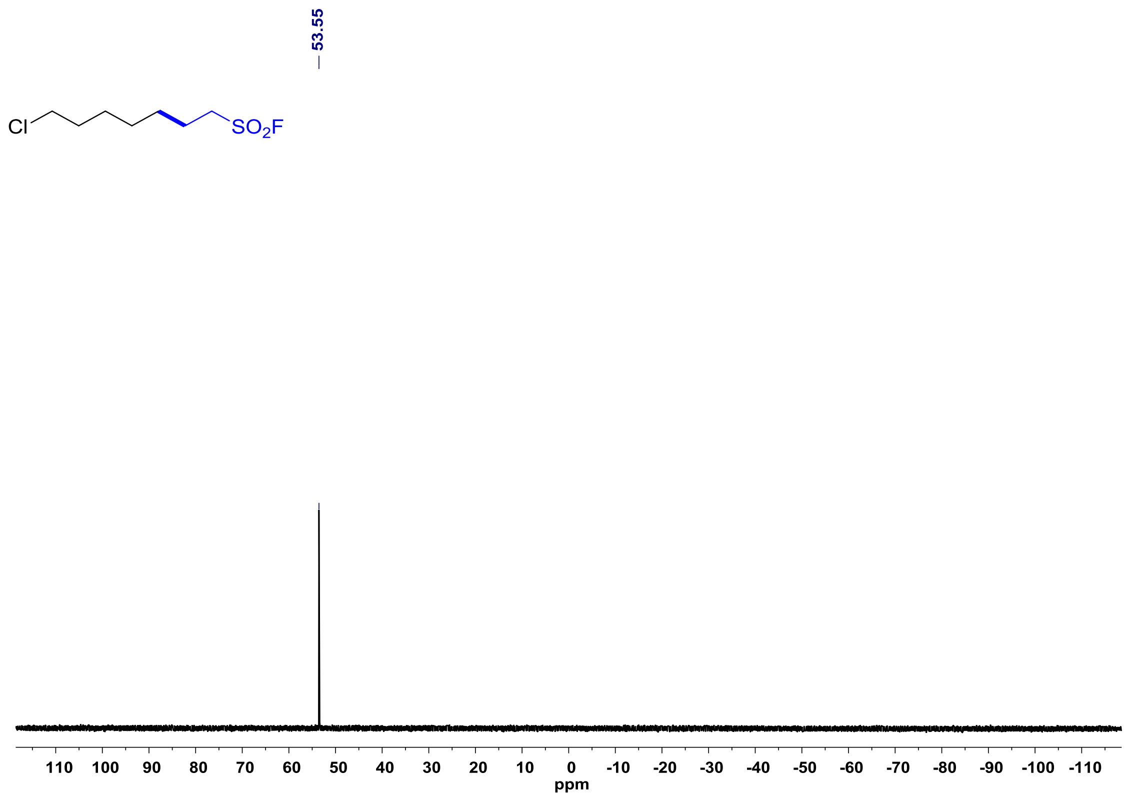

Supplementary Figure 33. ^{19}F NMR spectrum for compound **12**

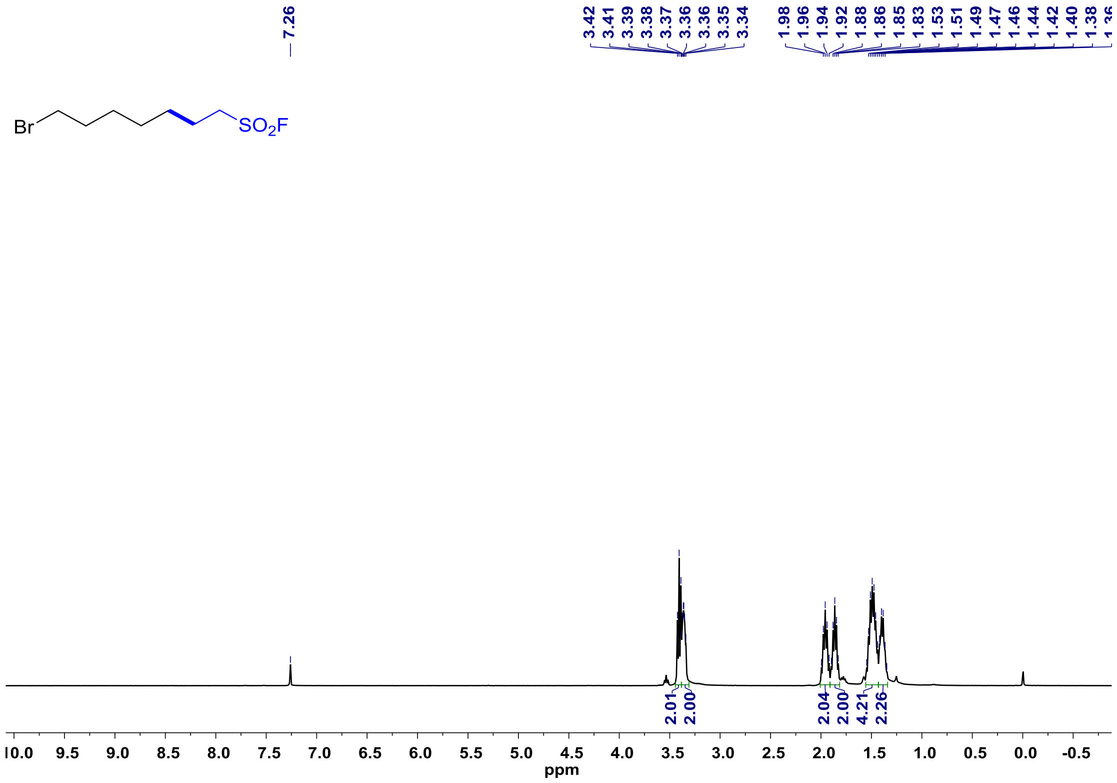

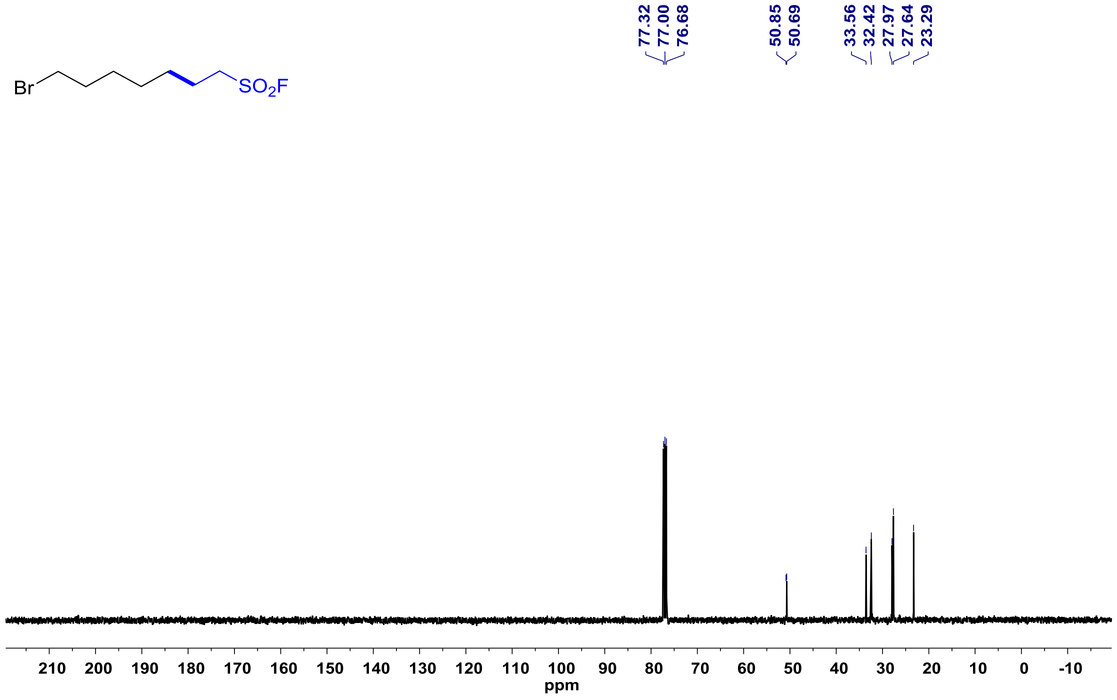

Supplementary Figure 34. ^{1}H and ^{13}C NMR spectra for compound **13**

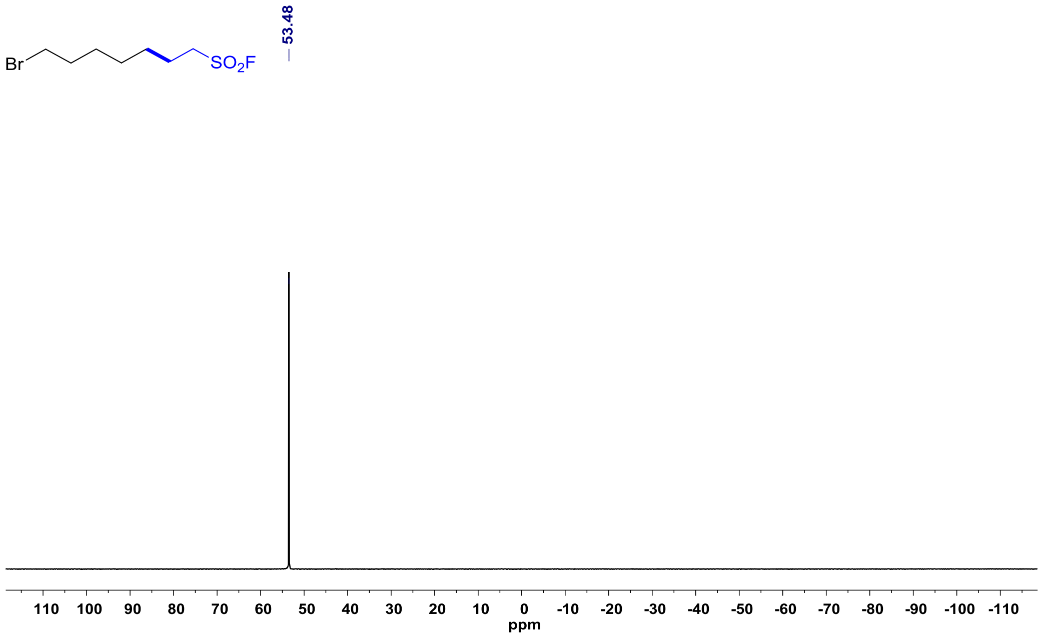

Supplementary Figure 35. ^{19}F NMR spectrum for compound **13**

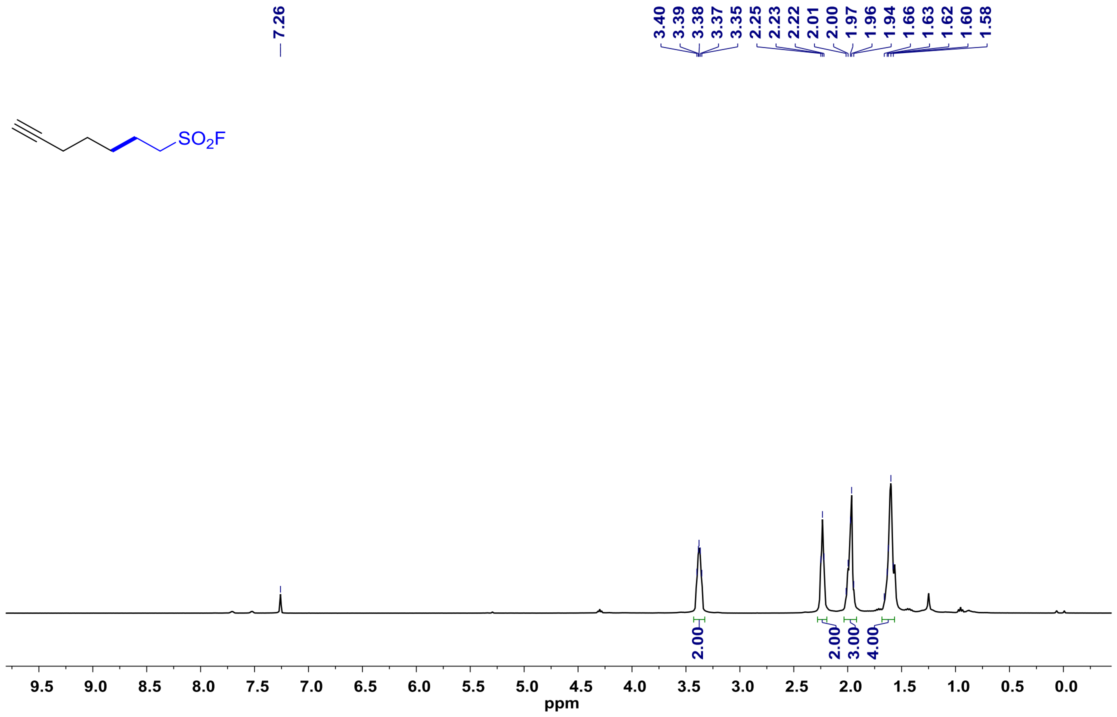

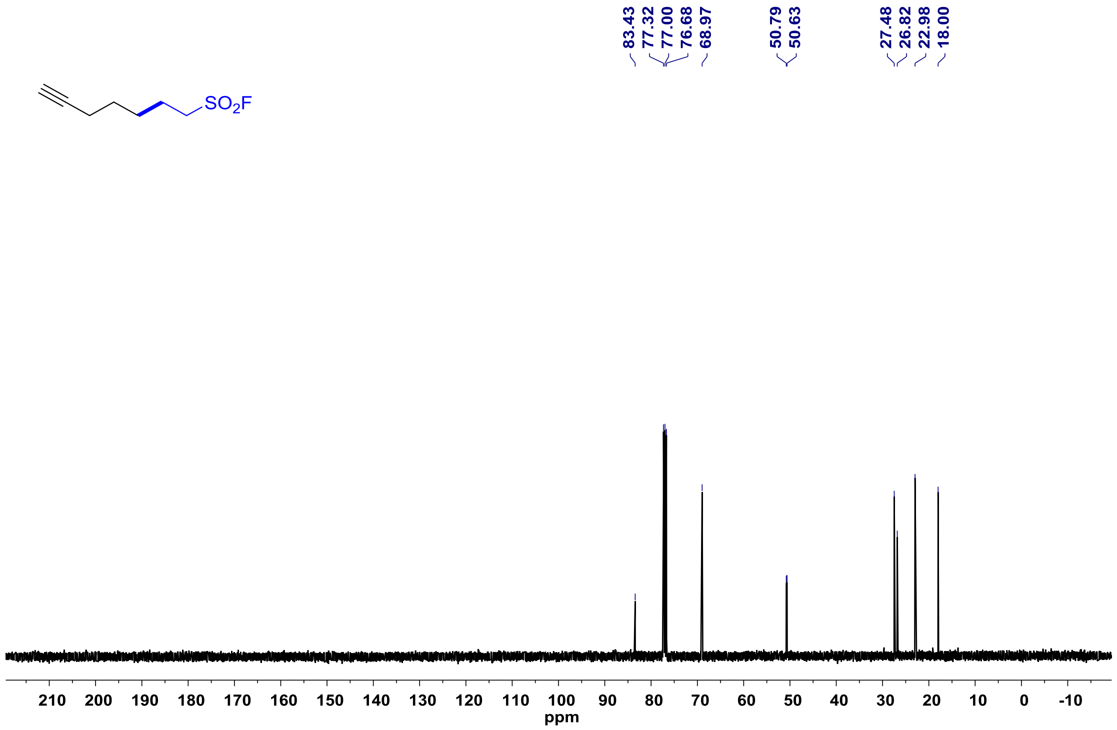

Supplementary Figure 36. ^{1}H and ^{13}C NMR spectra for compound **14**

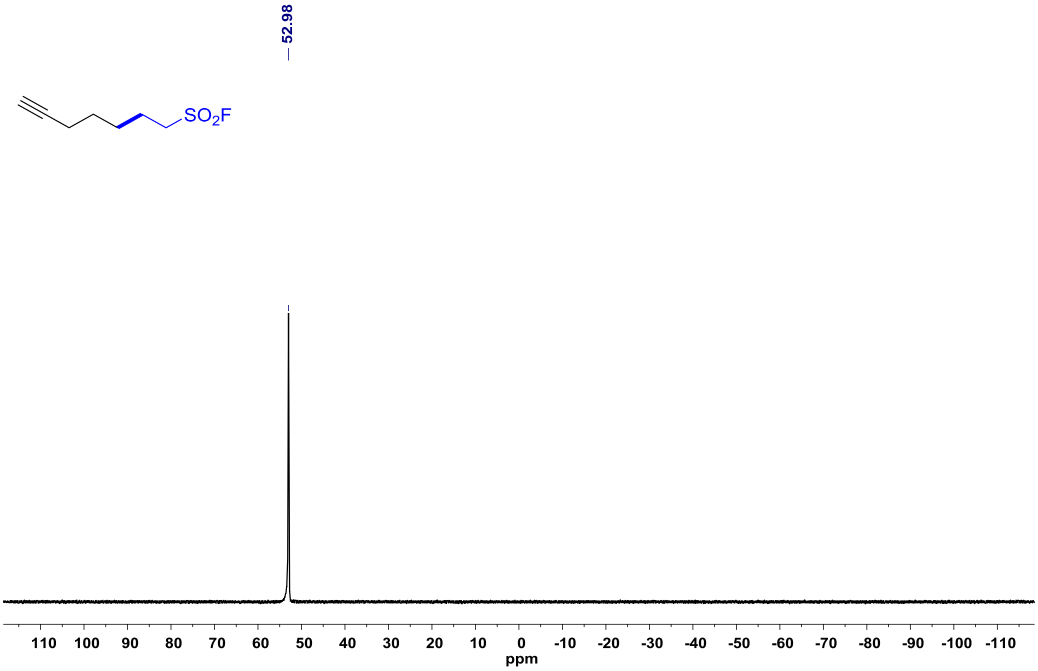

Supplementary Figure 37. ^{19}F NMR spectrum for compound **14**

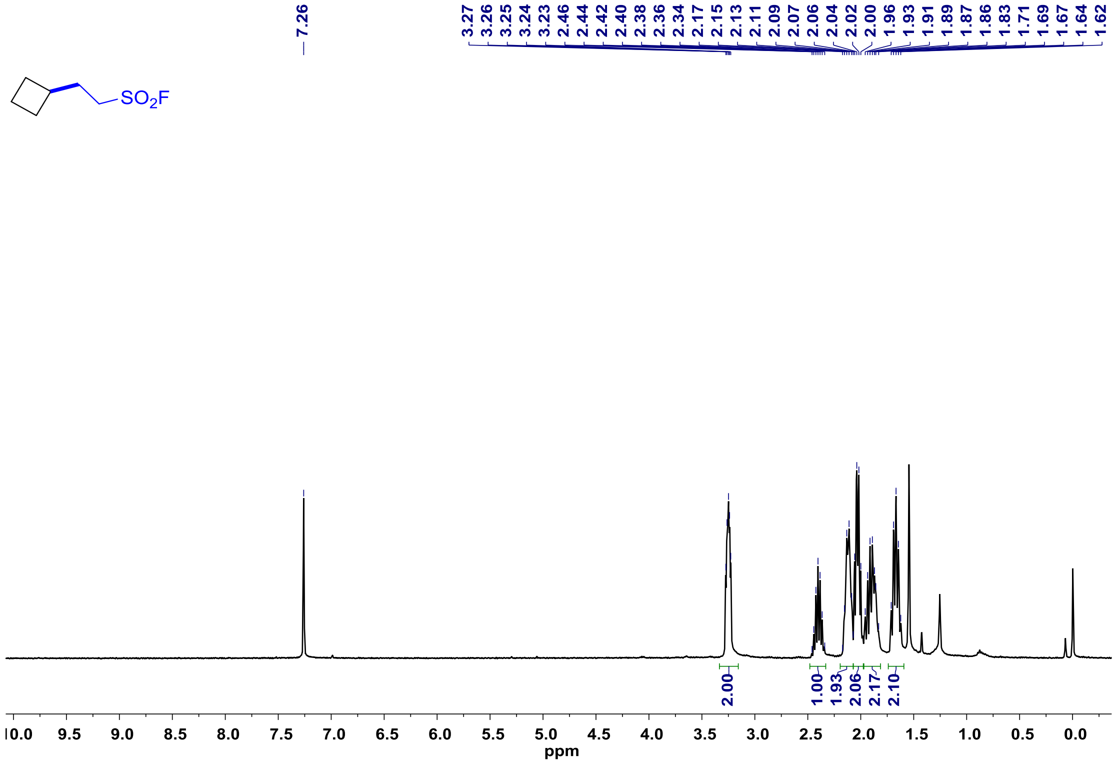

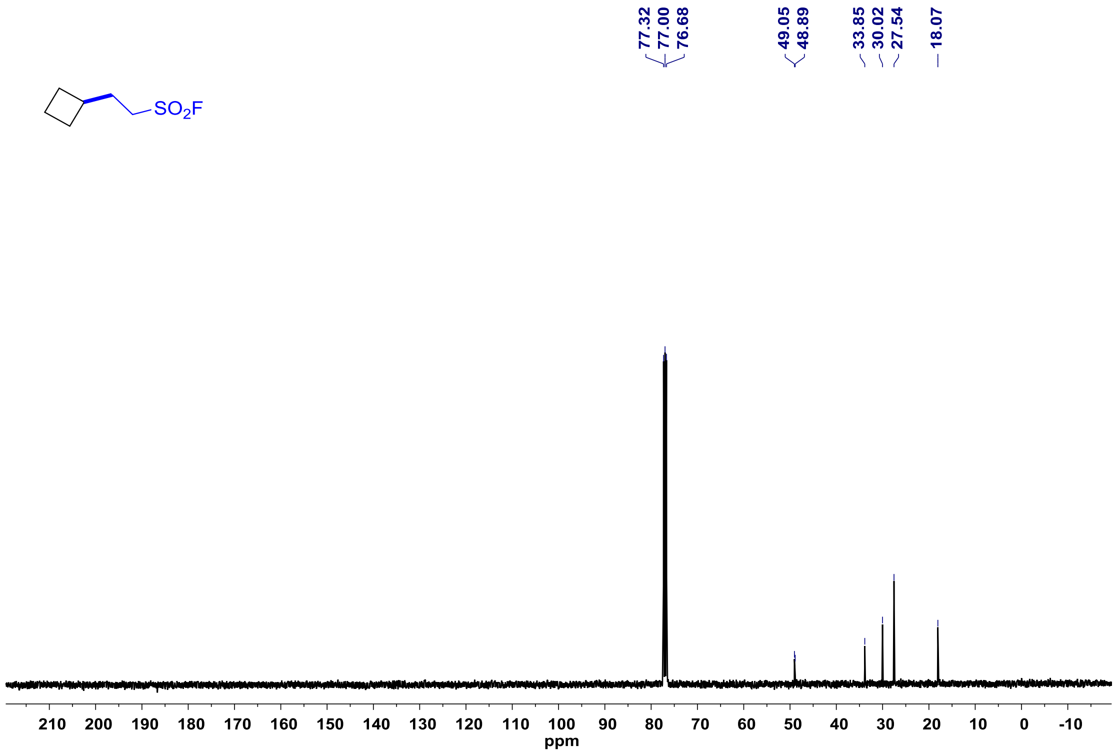

Supplementary Figure 38. ^{1}H and ^{13}C NMR spectra for compound **15**

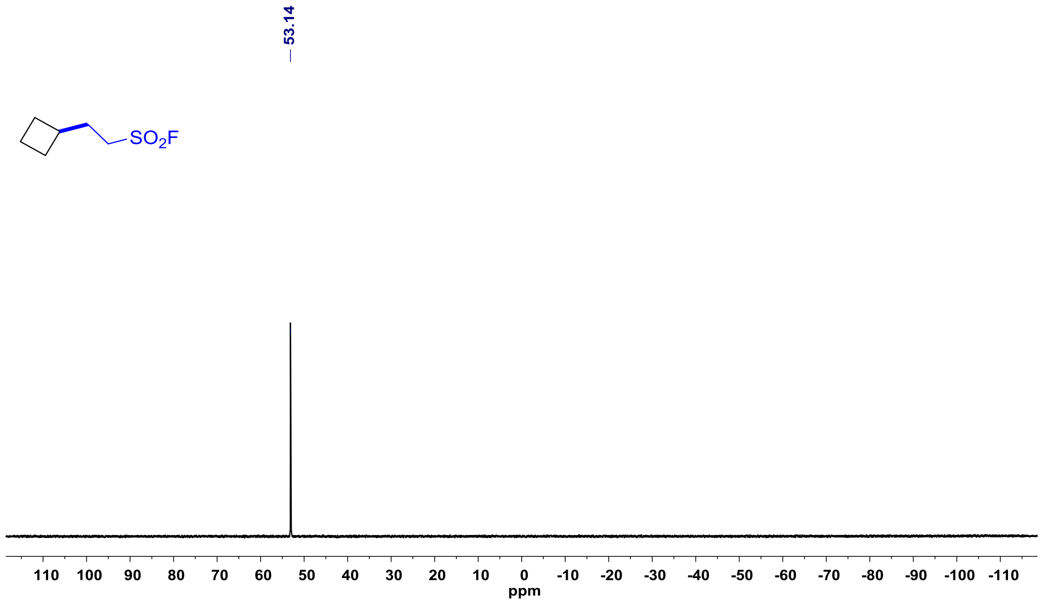

Supplementary Figure 39. ^{19}F NMR spectrum for compound **15**

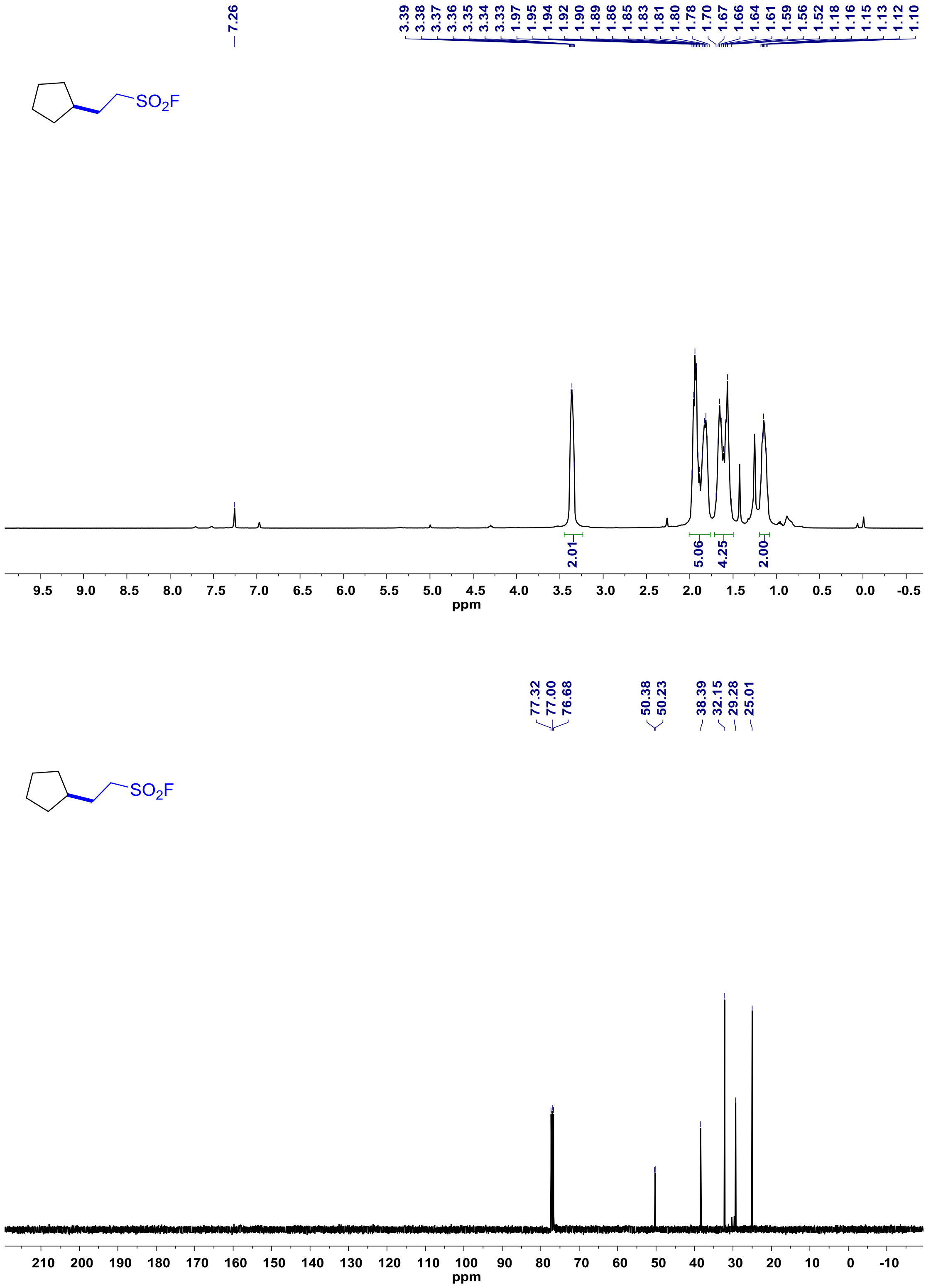

Supplementary Figure 40. ^{1}H and ^{13}C NMR spectra for compound **16**

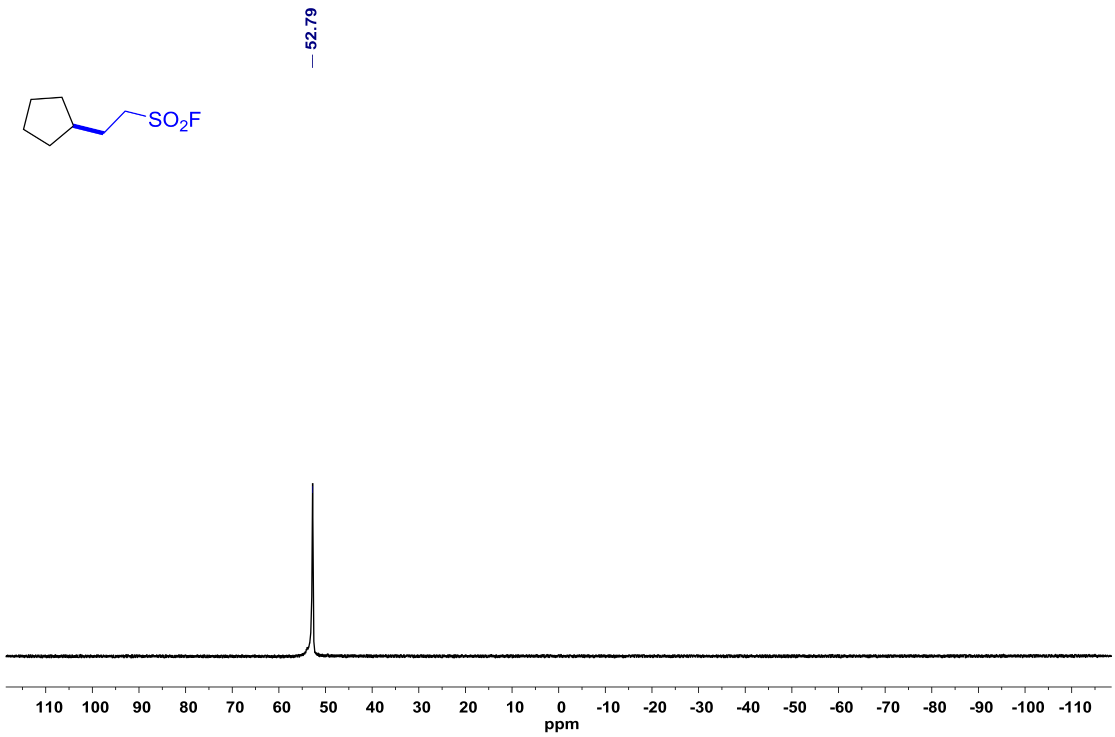

Supplementary Figure 41. ^{19}F NMR spectrum for compound **16**

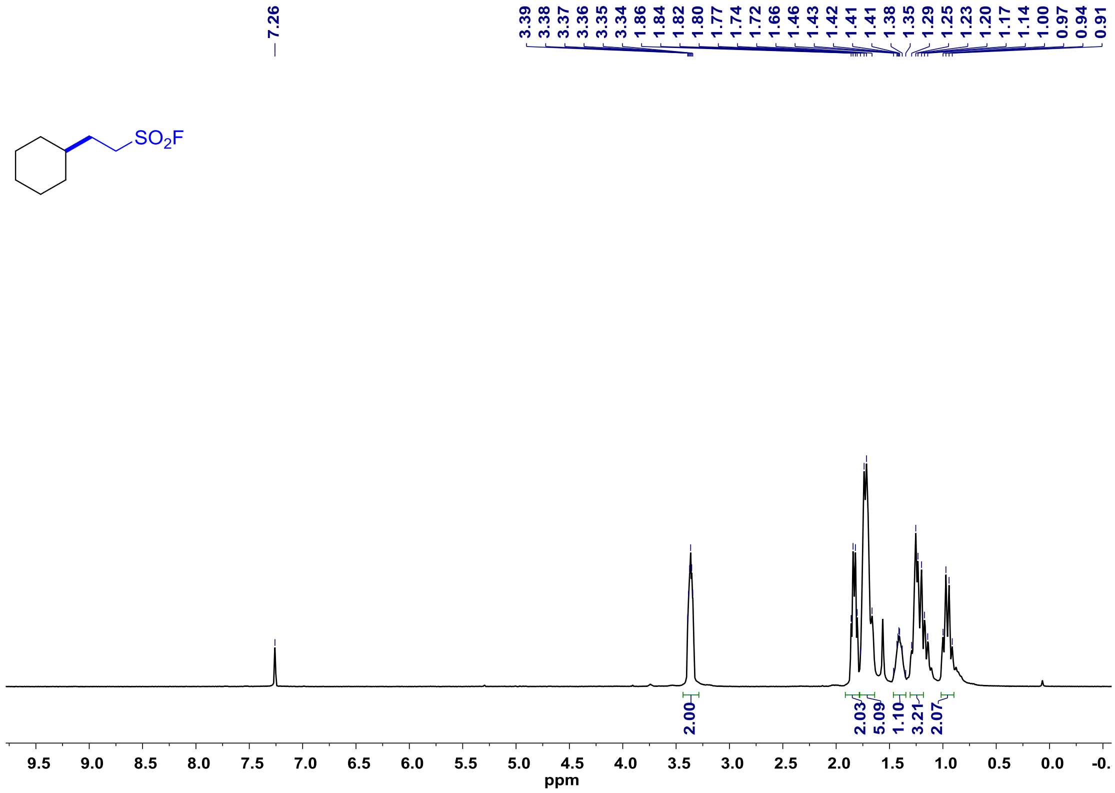

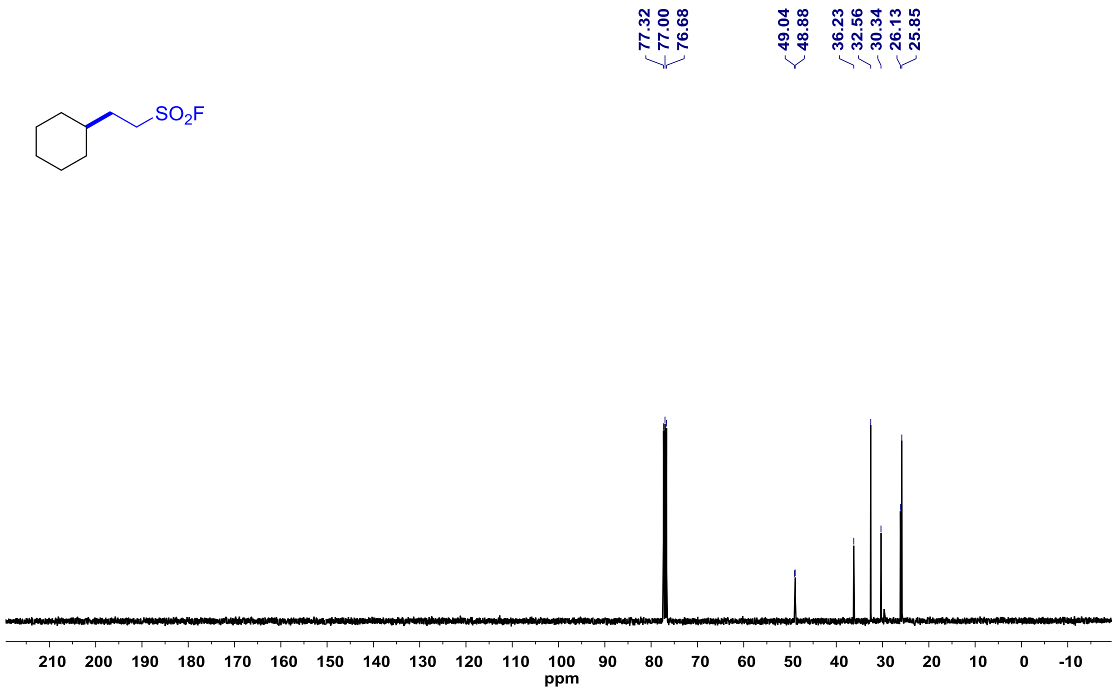

Supplementary Figure 42. ^{1}H and ^{13}C NMR spectra for compound **17**

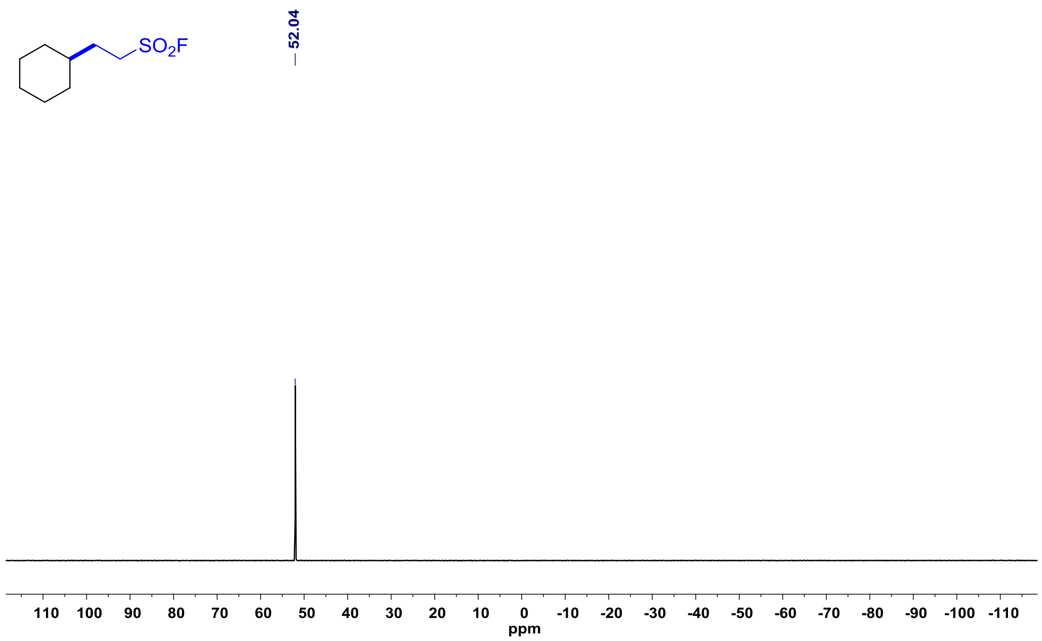

Supplementary Figure 43. ^{19}F NMR spectrum for compound **17**

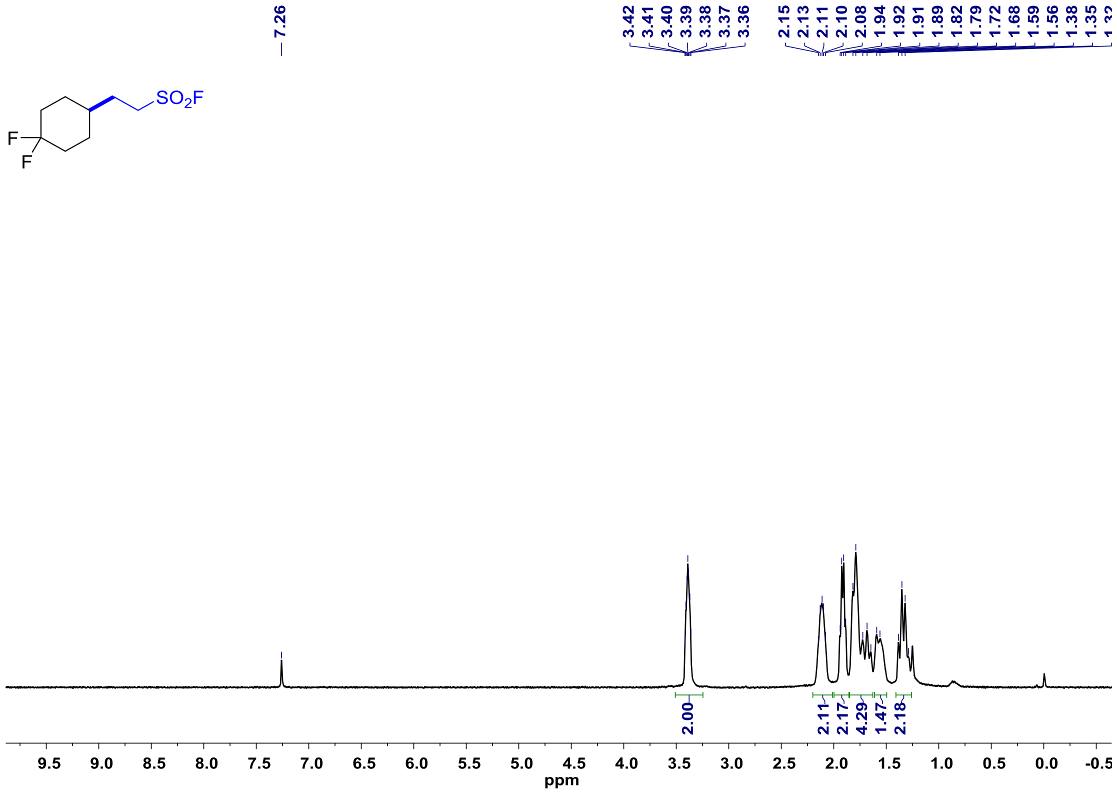

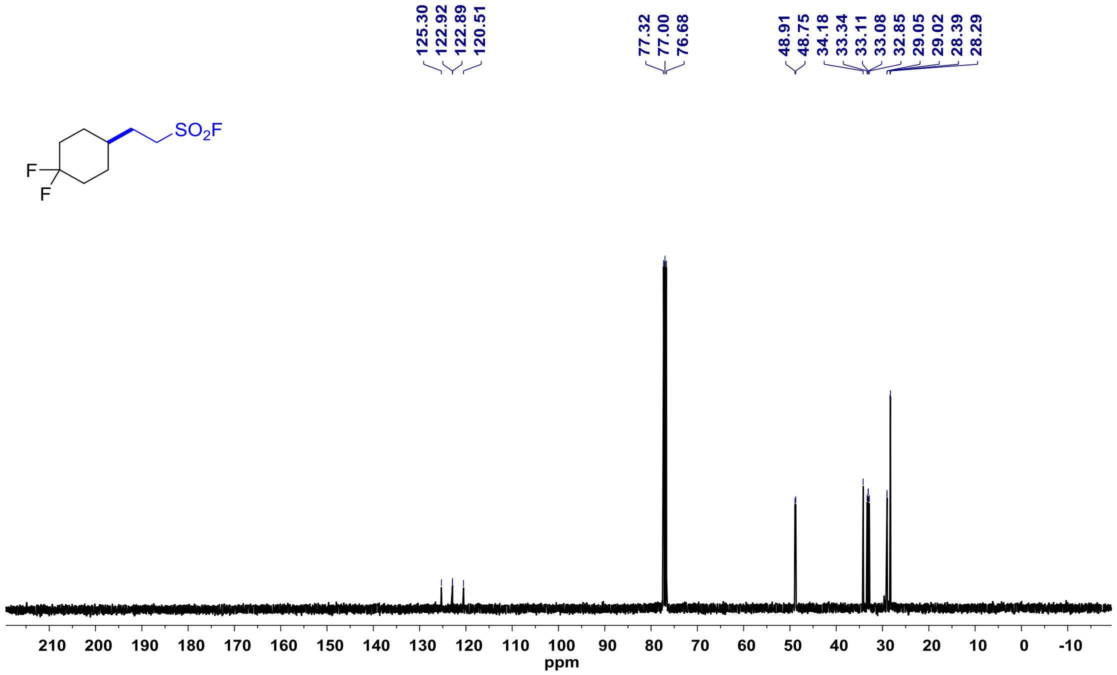

Supplementary Figure 44. ^{1}H and ^{13}C NMR spectra for compound **18**

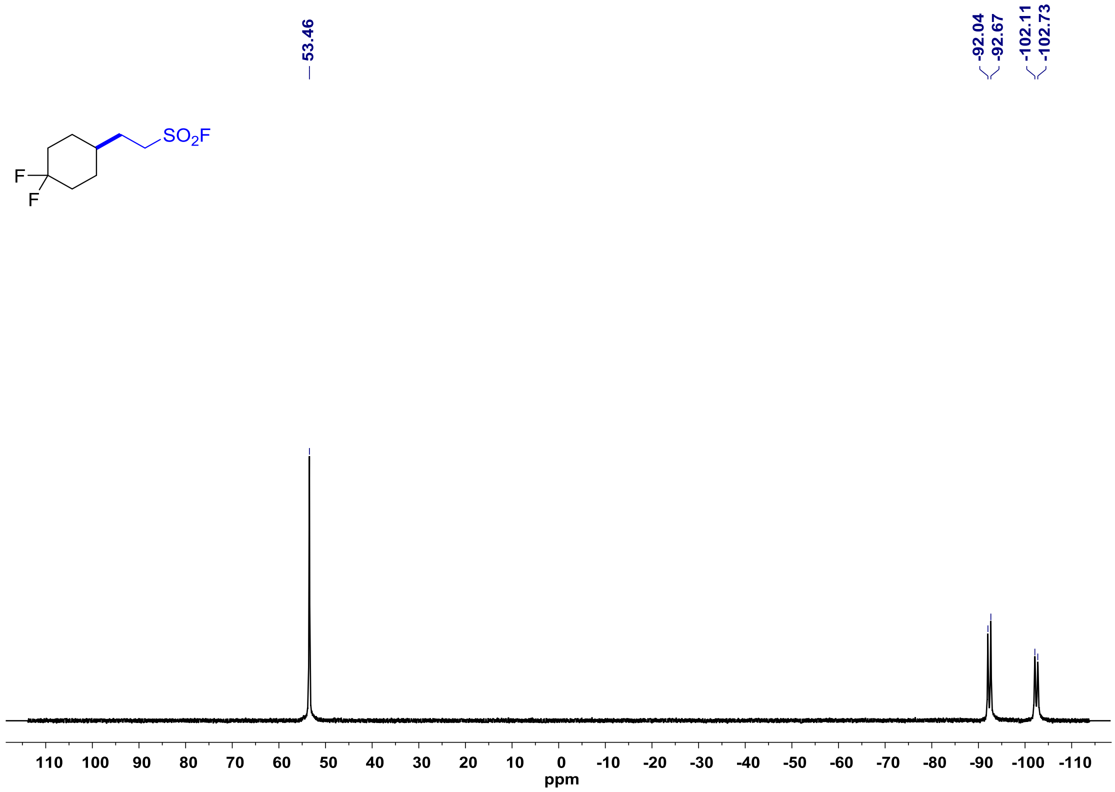

Supplementary Figure 45. ^{19}F NMR spectrum for compound **18**

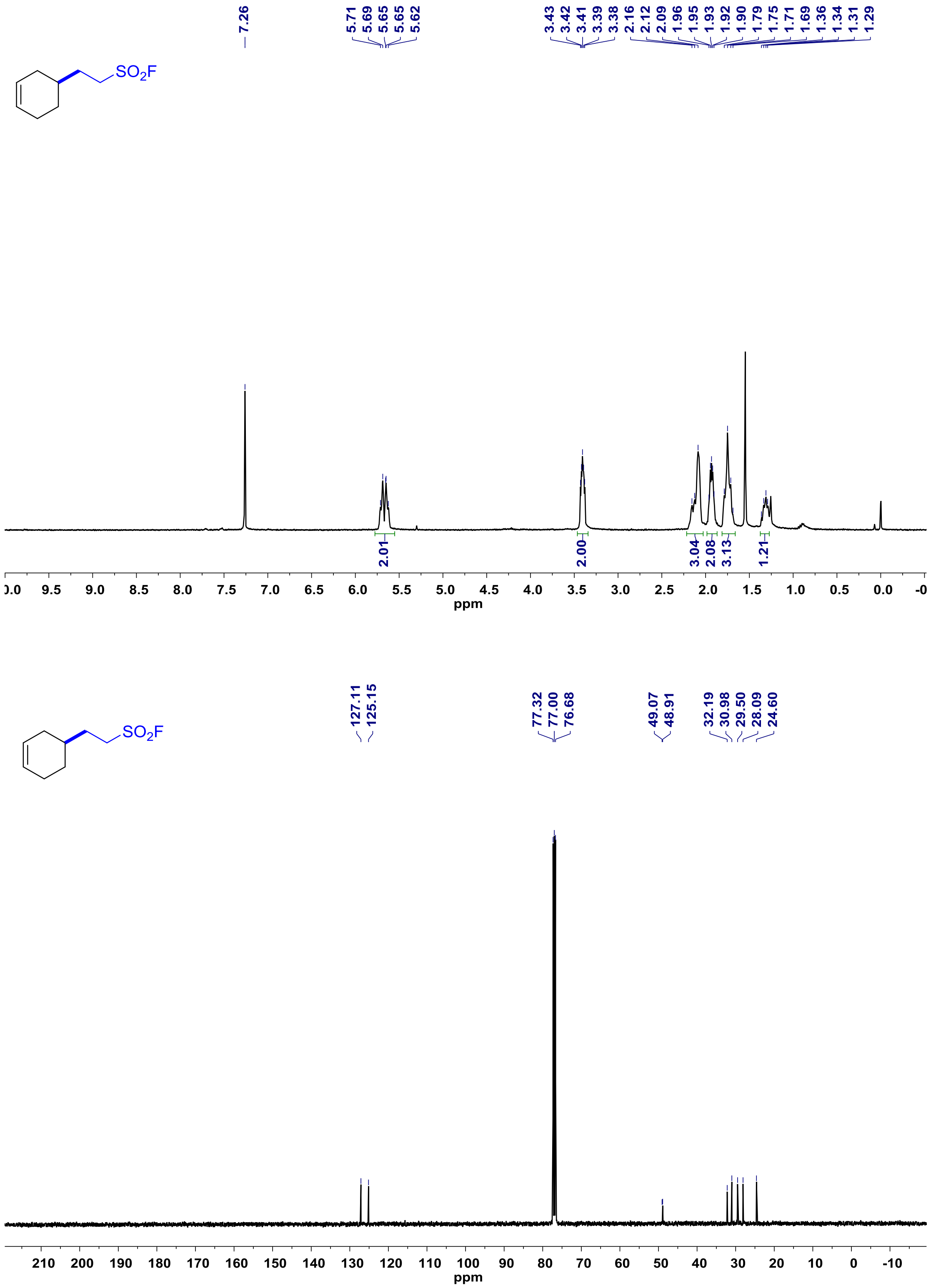

Supplementary Figure 46. ^{1}H and ^{13}C NMR spectra for compound **19**

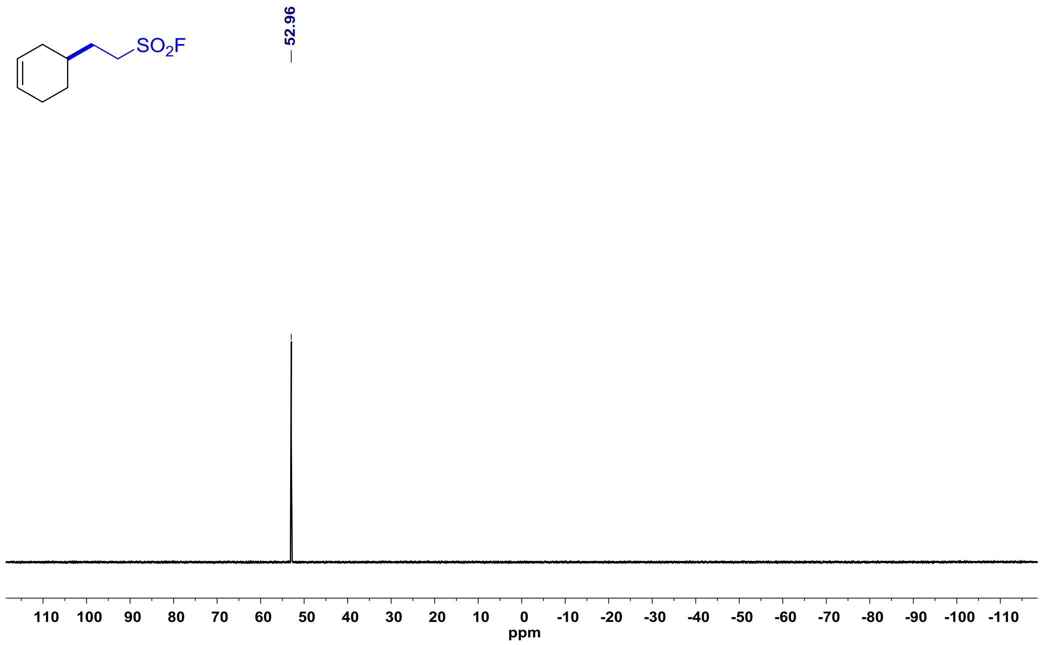

Supplementary Figure 47. ^{19}F NMR spectrum for compound **19**

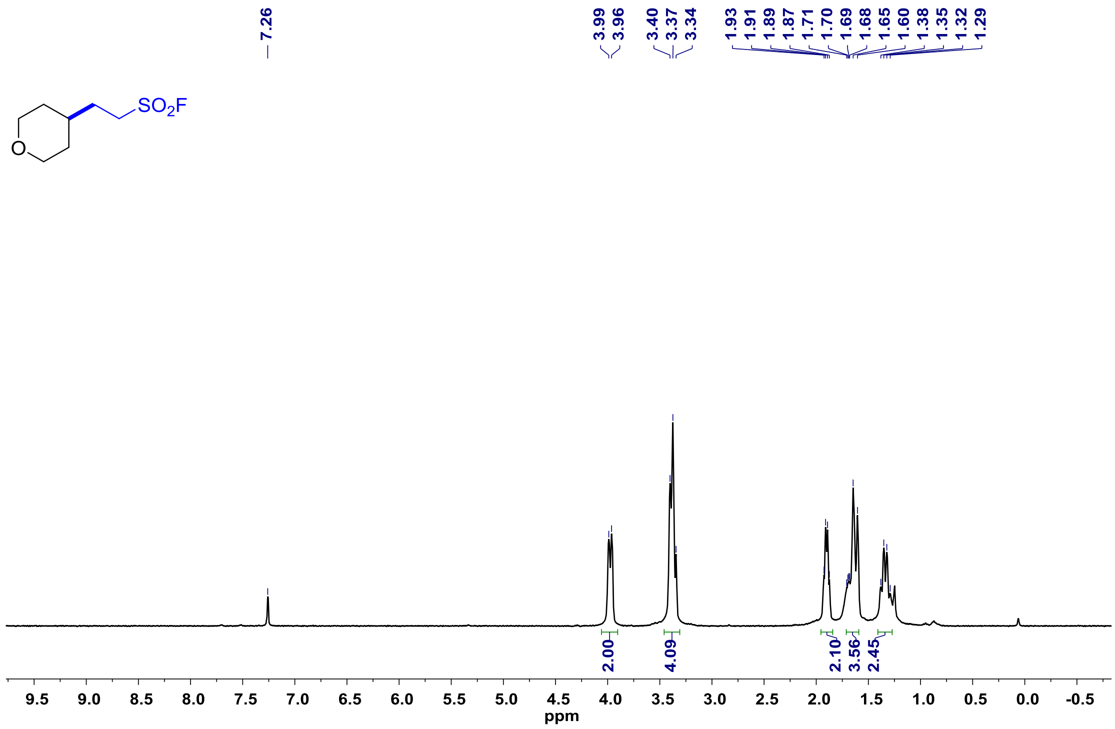

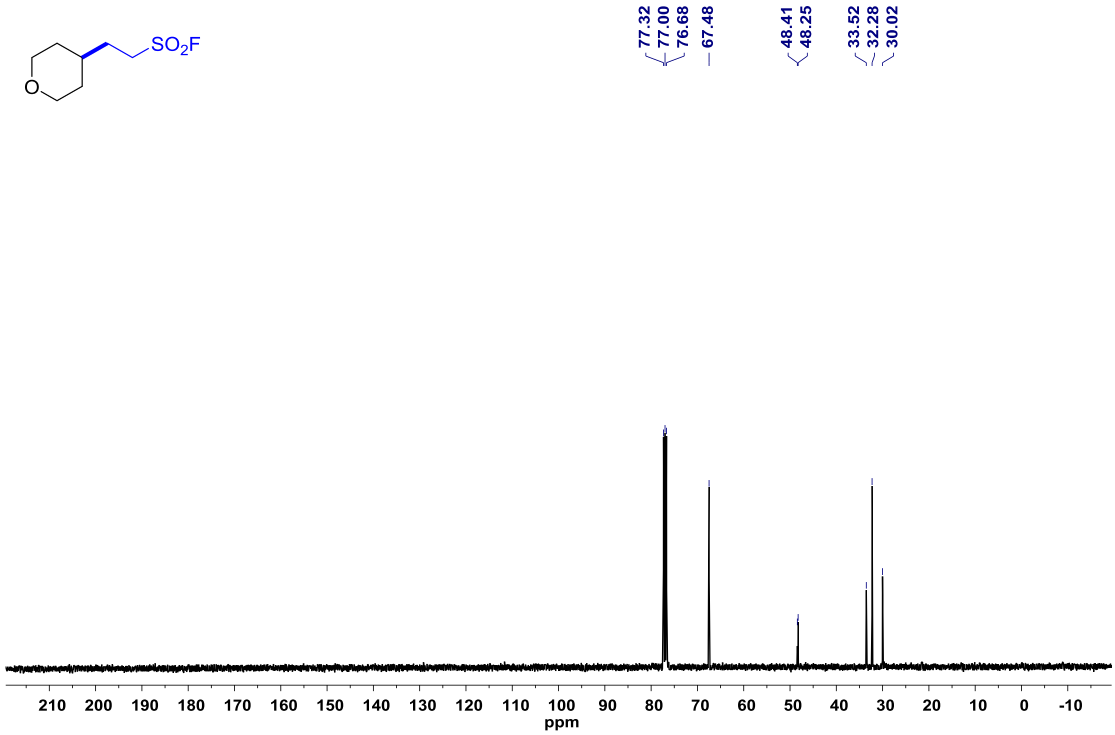

Supplementary Figure 48. ^{1}H and ^{13}C NMR spectra for compound **20**

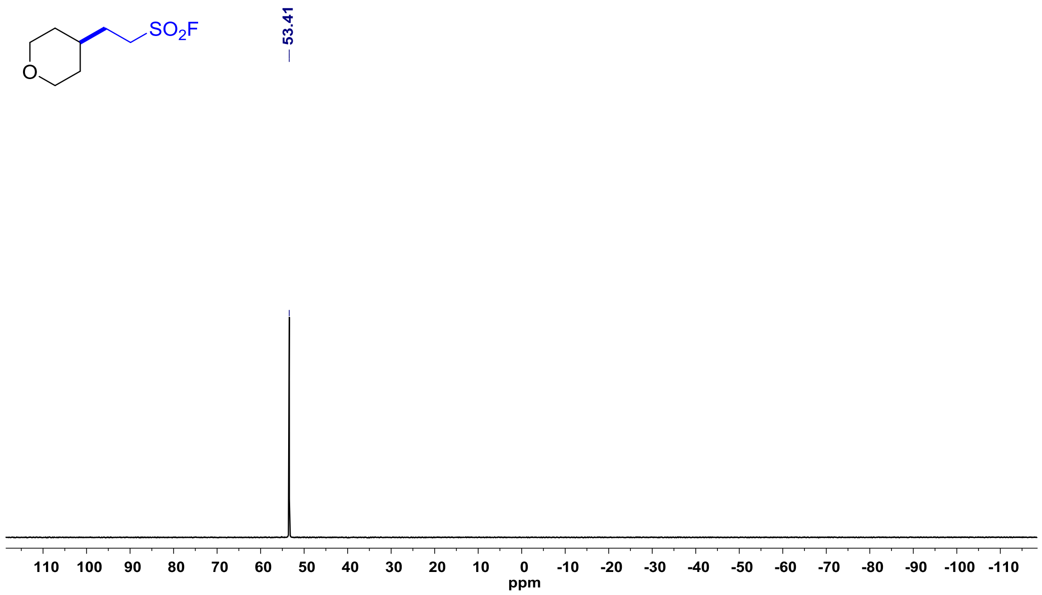

Supplementary Figure 49. ^{19}F NMR spectrum for compound **20**

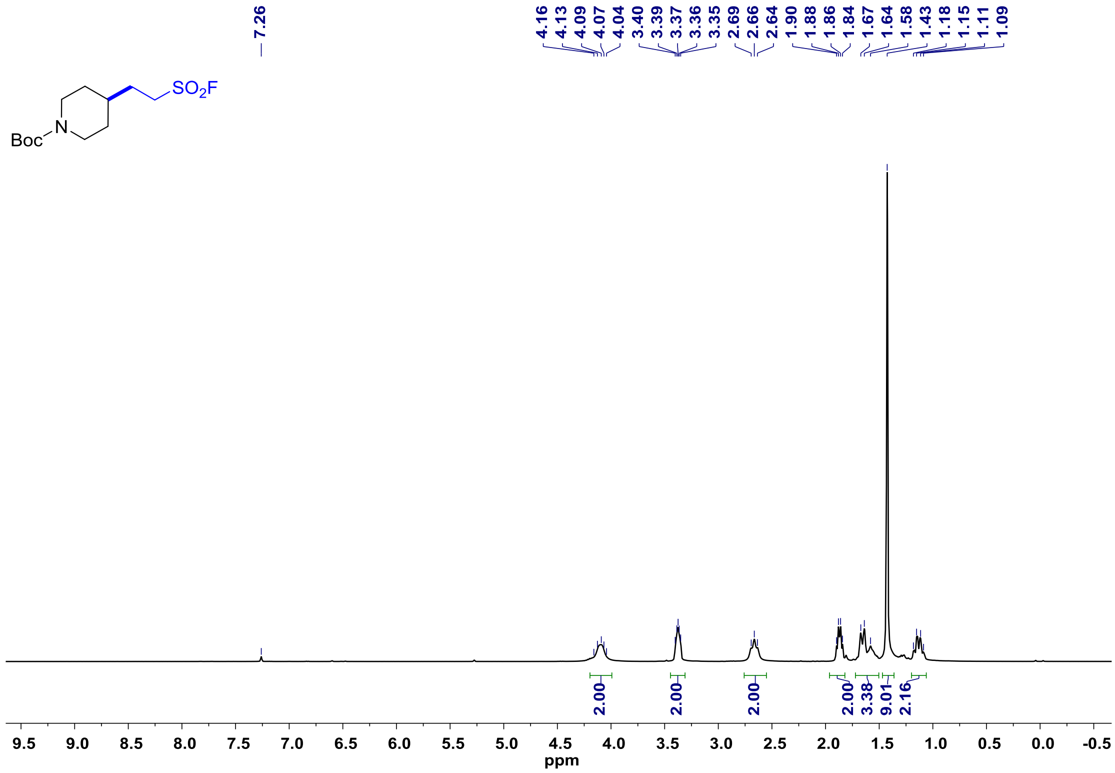

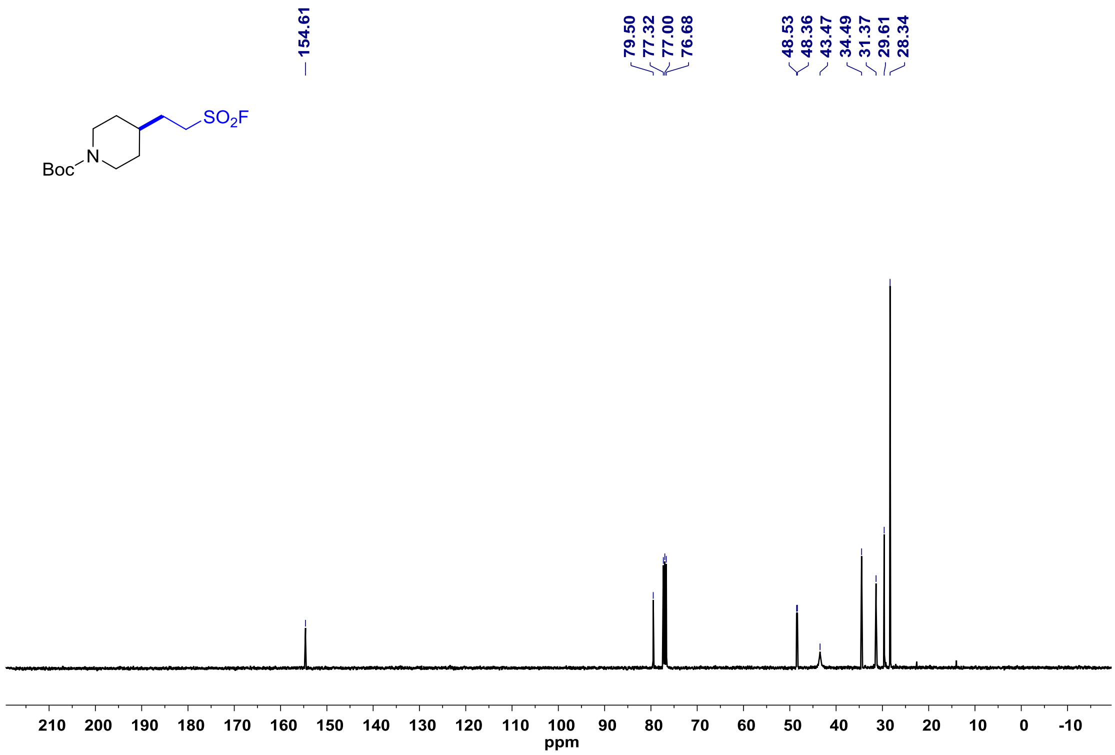

Supplementary Figure 50. ^{1}H and ^{13}C NMR spectra for compound **21**

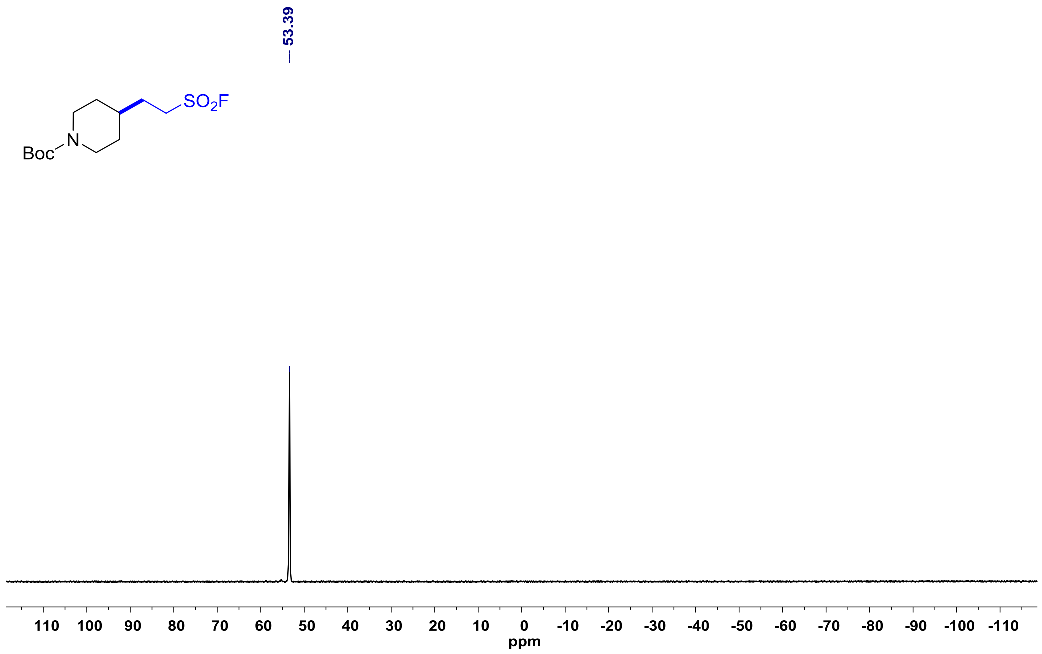

Supplementary Figure 51. ^{19}F NMR spectrum for compound **21**

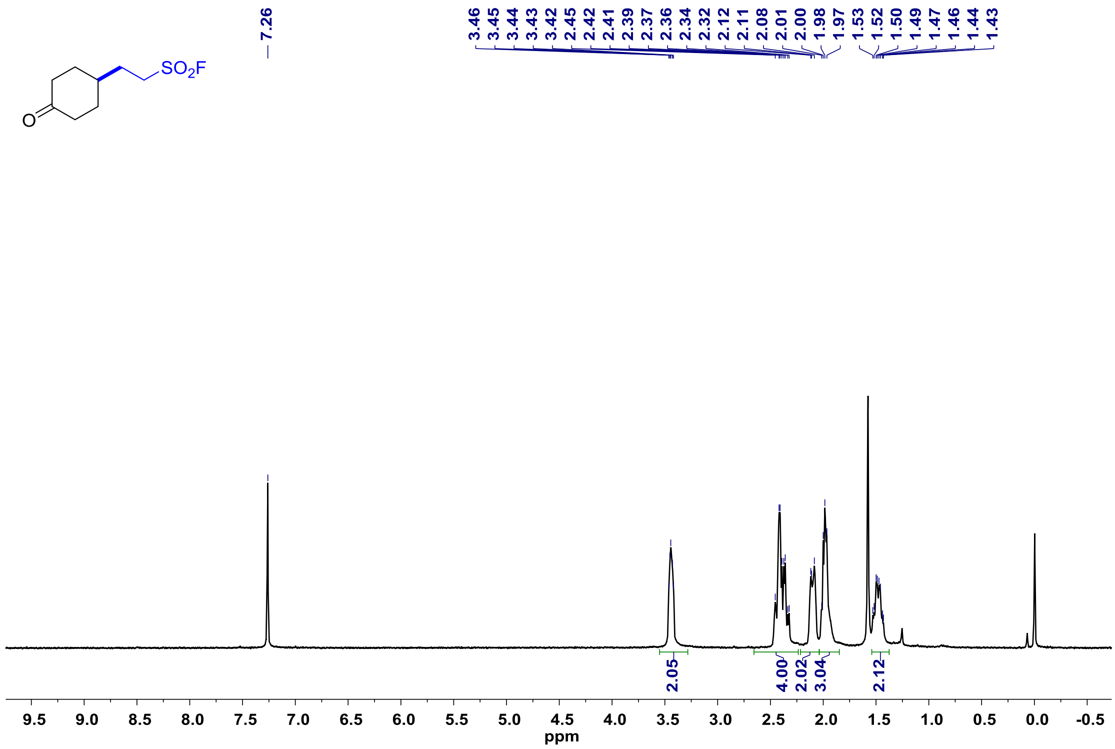

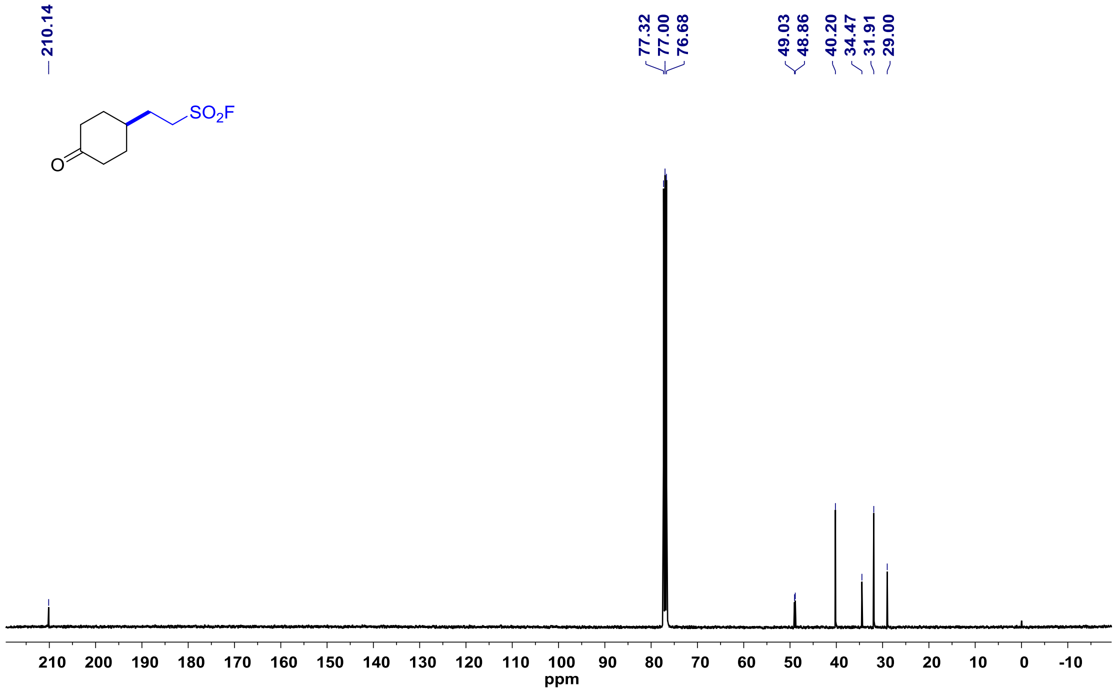

Supplementary Figure 52. ^{1}H and ^{13}C NMR spectra for compound **22**

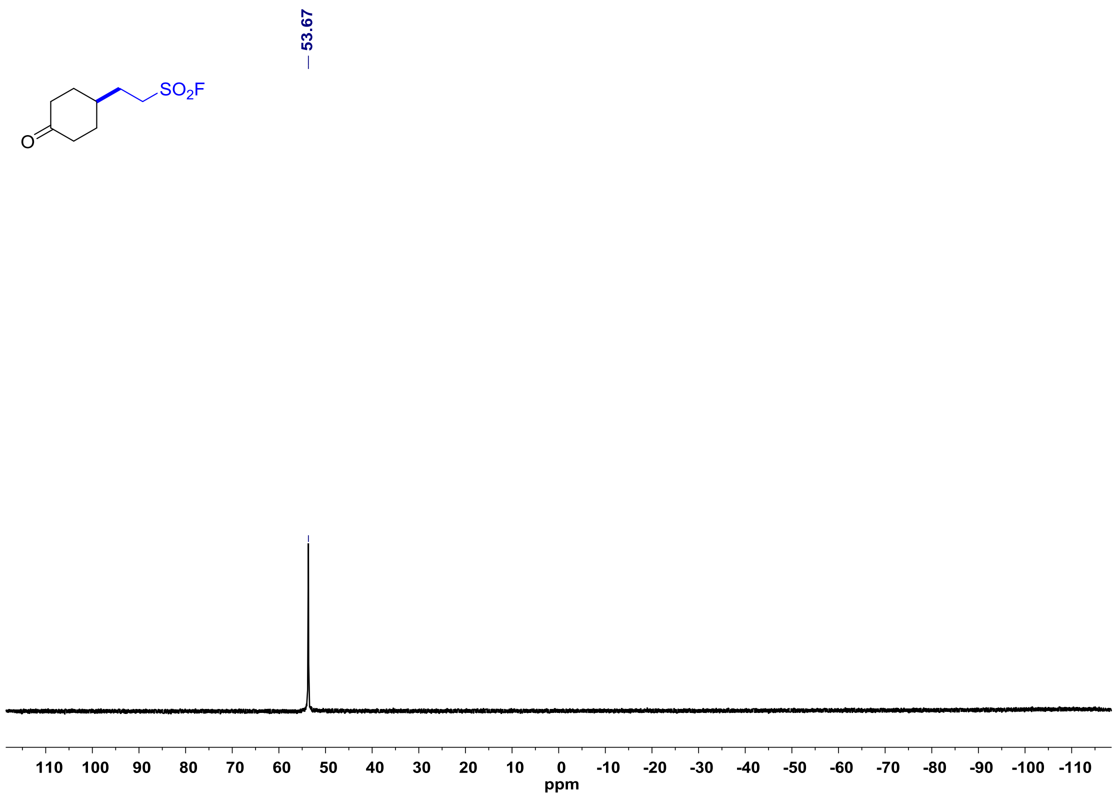

Supplementary Figure 53. ^{19}F NMR spectrum for compound **22**

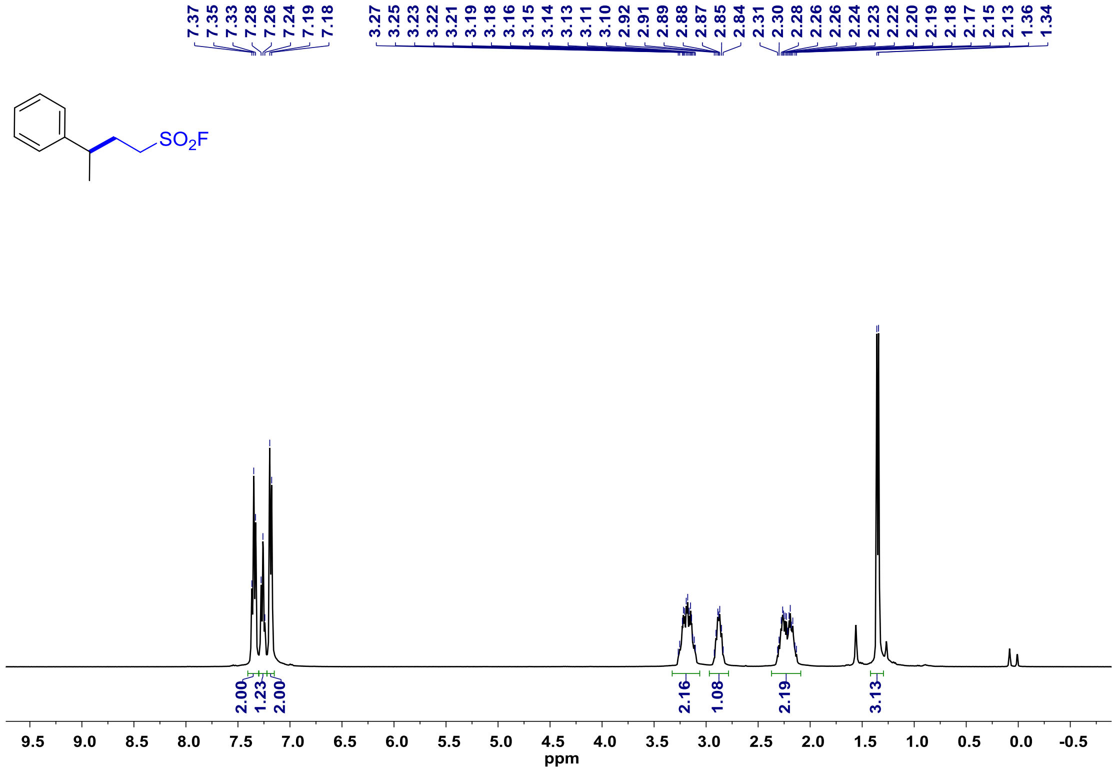

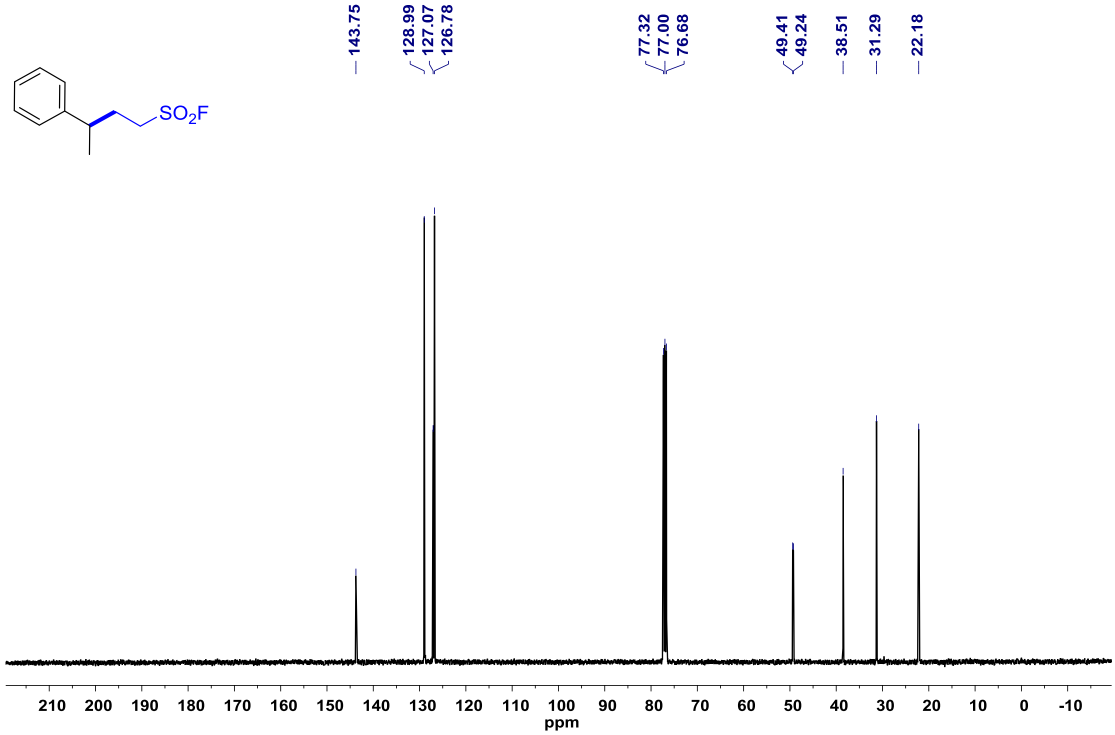

Supplementary Figure 54. ^{1}H and ^{13}C NMR spectra for compound **23**

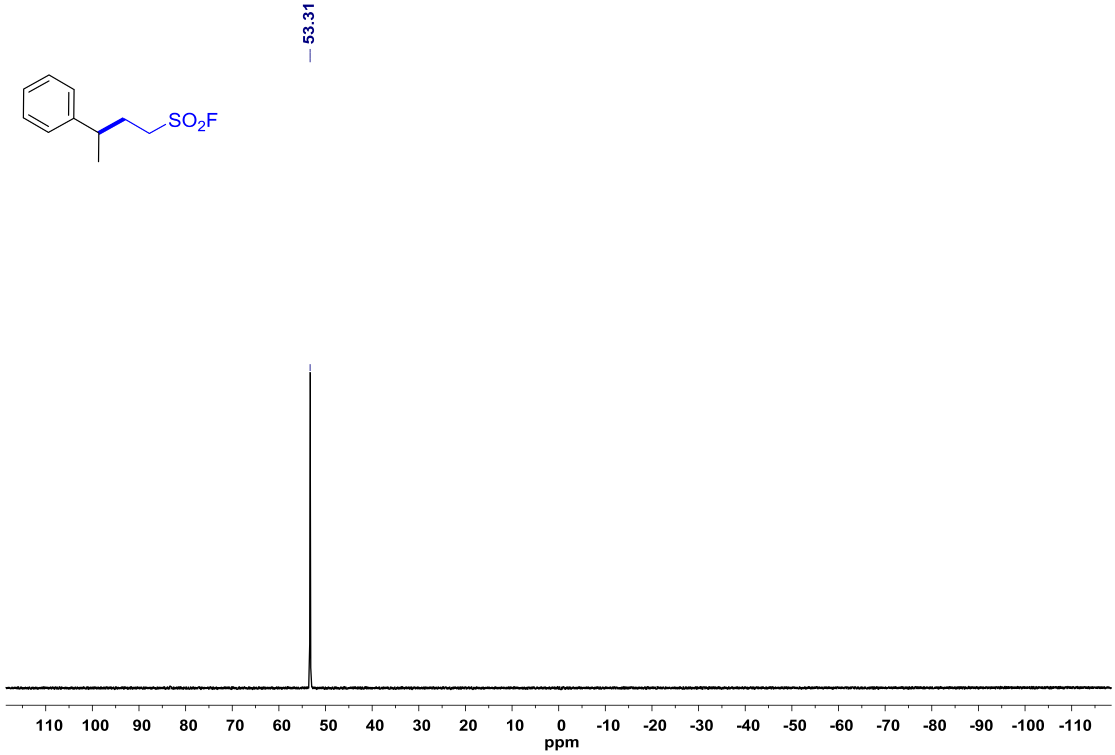

Supplementary Figure 55. ^{19}F NMR spectrum for compound **23**

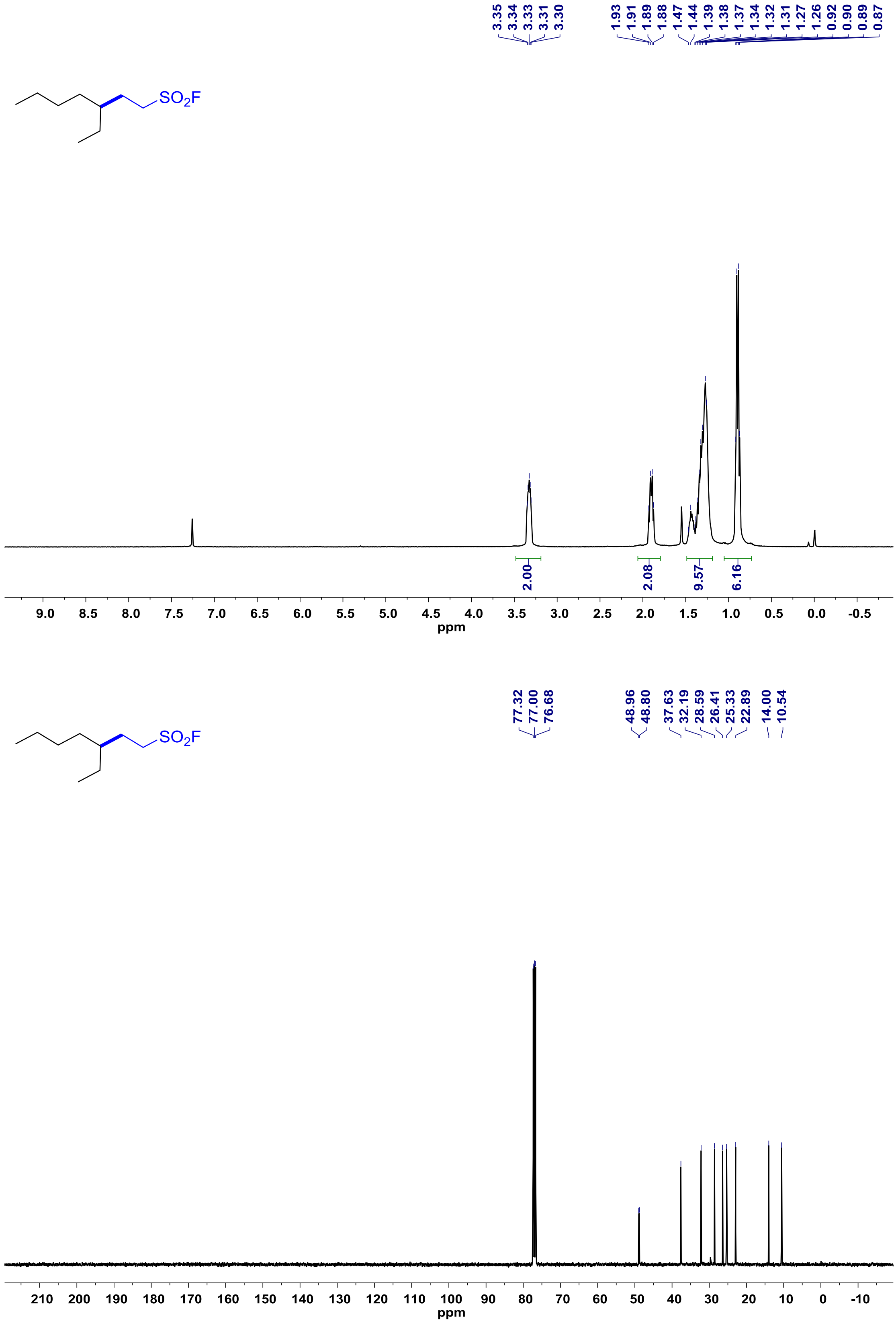

Supplementary Figure 56. ^{1}H and ^{13}C NMR spectra for compound **24**

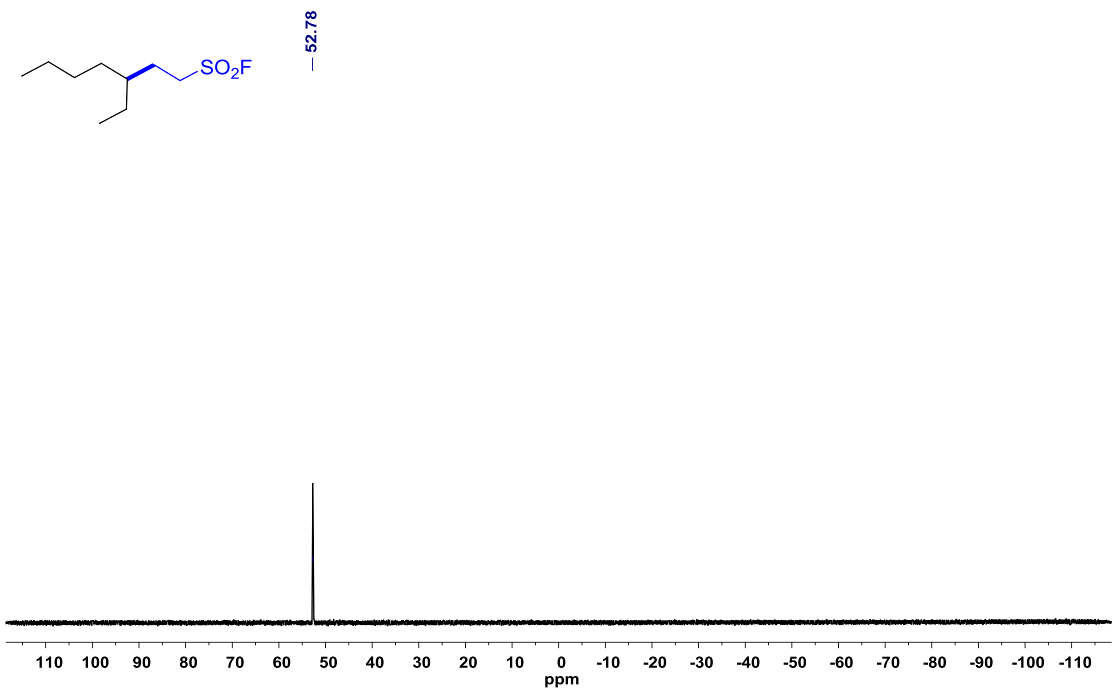

Supplementary Figure 57. ^{19}F NMR spectrum for compound **24**

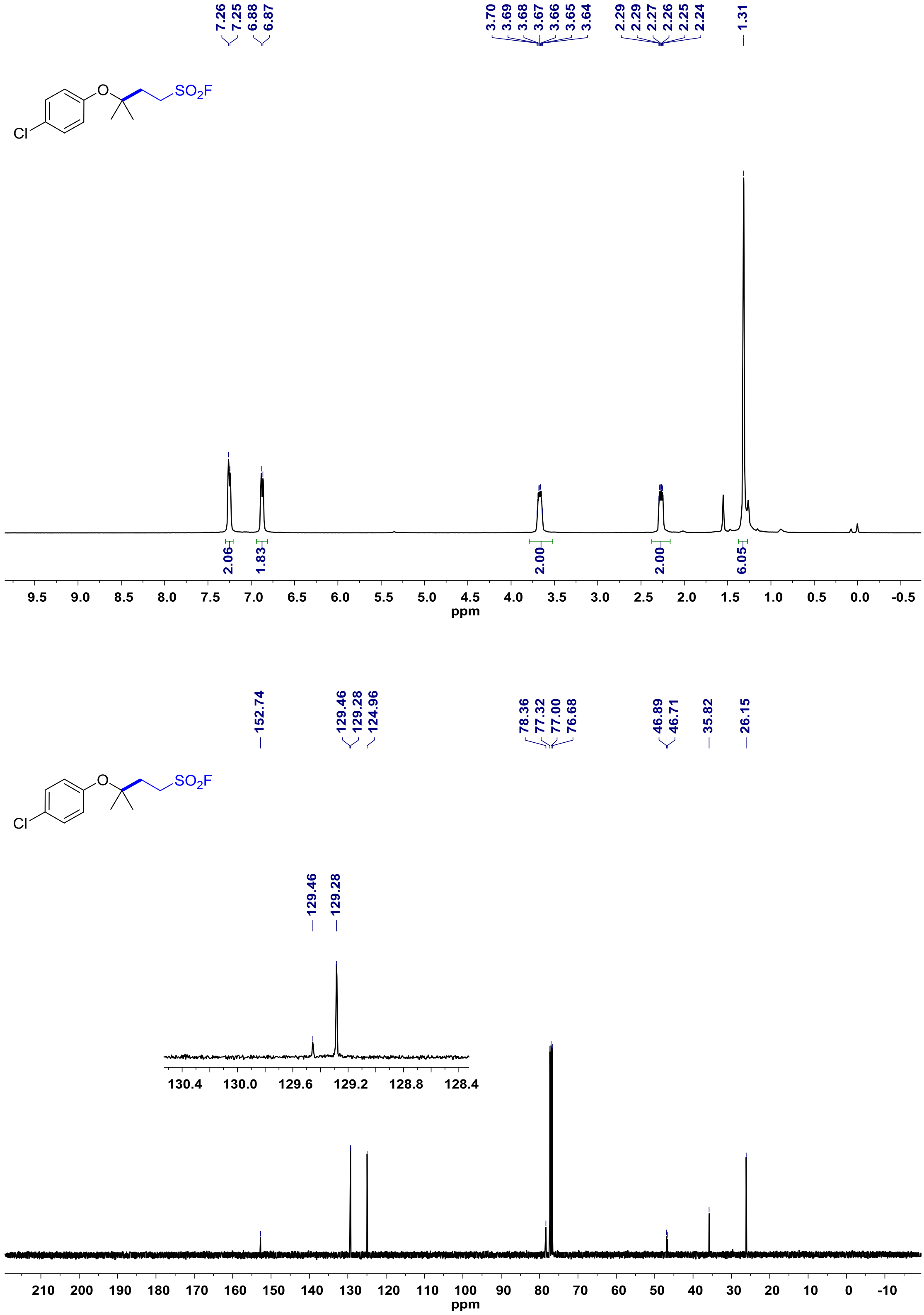

Supplementary Figure 58. ^{1}H and ^{13}C NMR spectra for compound **25**

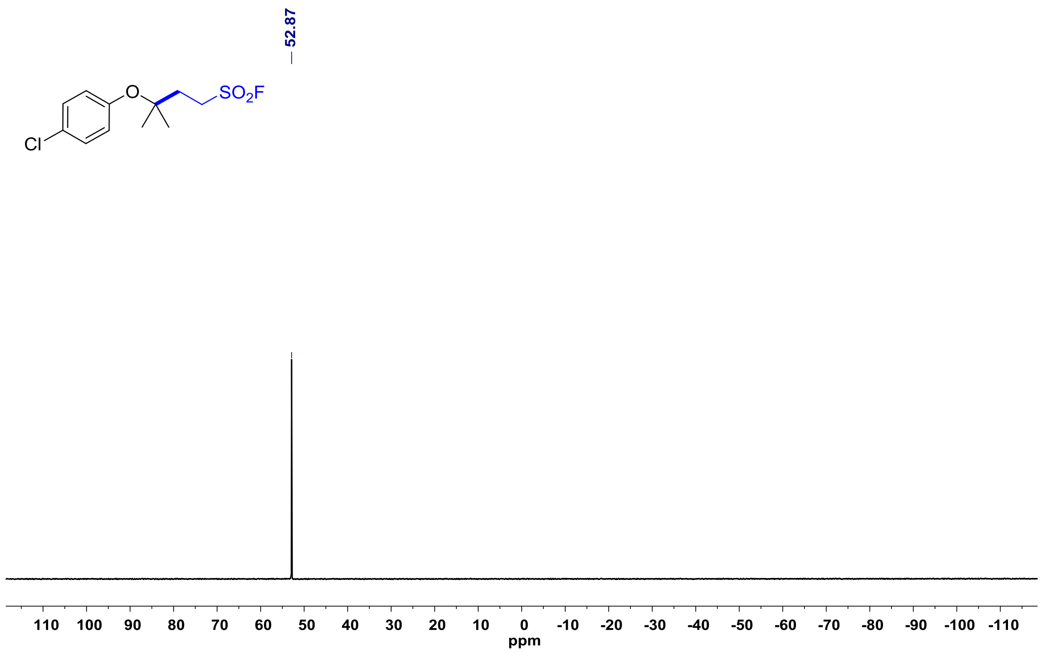

Supplementary Figure 59. ^{19}F NMR spectrum for compound **25**

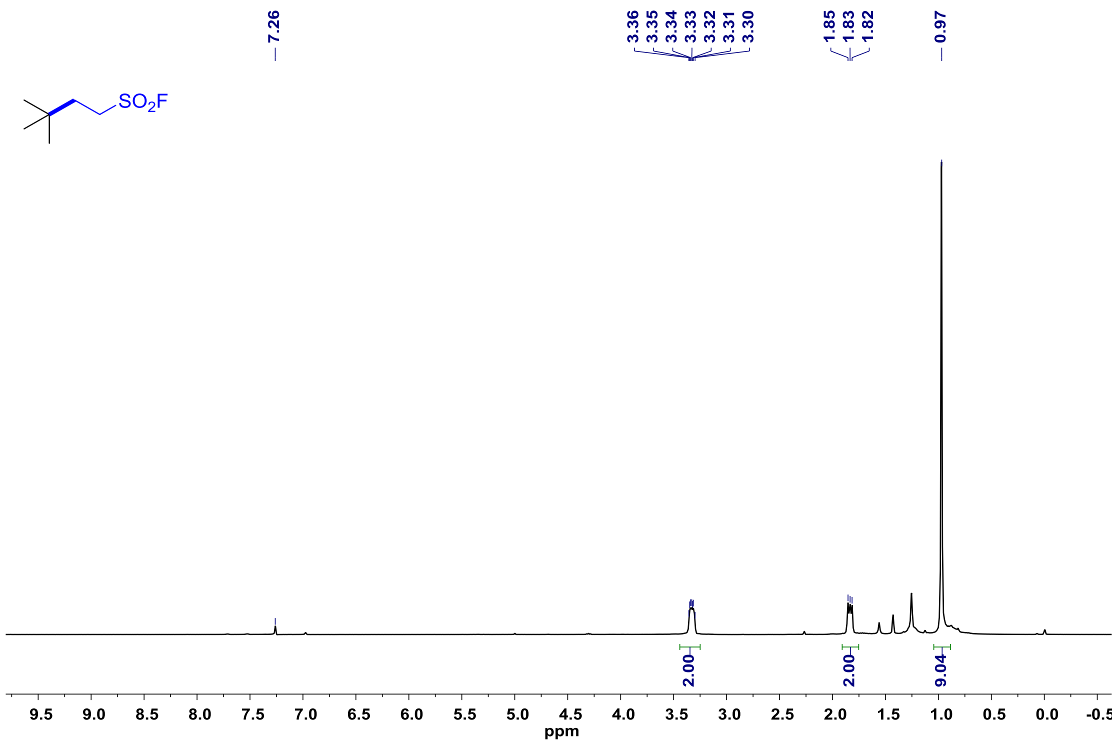

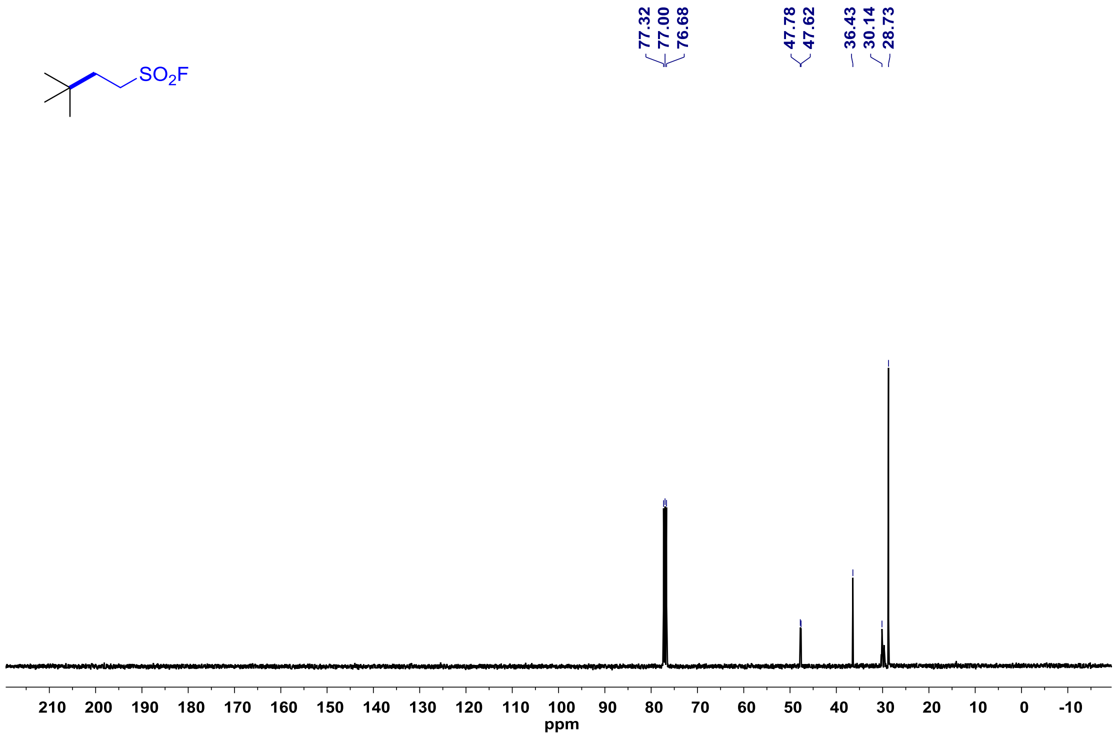

Supplementary Figure 60. ^{1}H and ^{13}C NMR spectra for compound **26**

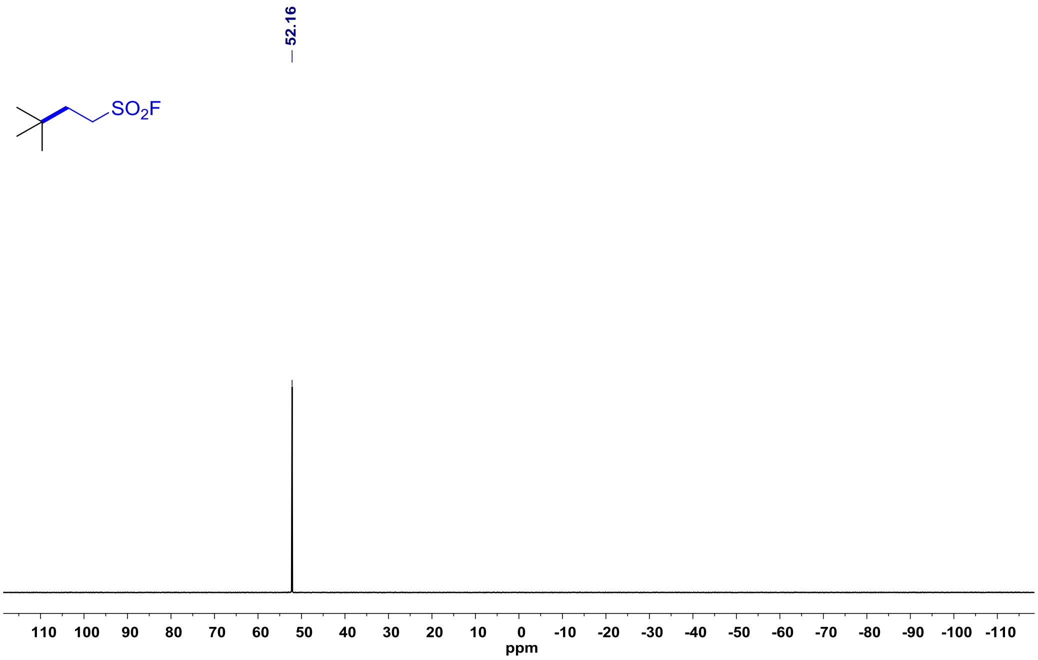

Supplementary Figure 61. ^{19}F NMR spectrum for compound **26**

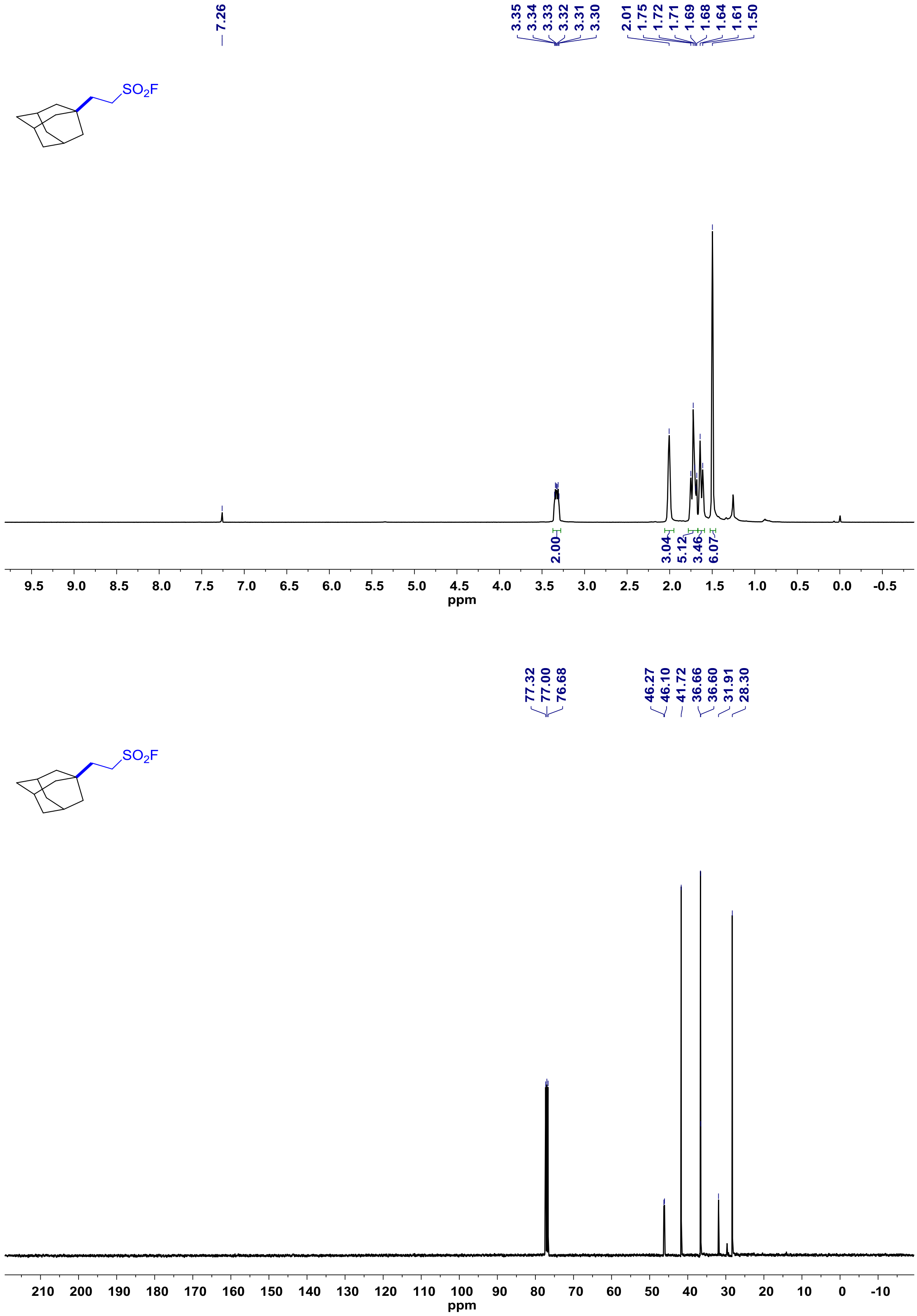

Supplementary Figure 62. ^{1}H and ^{13}C NMR spectra for compound **27**

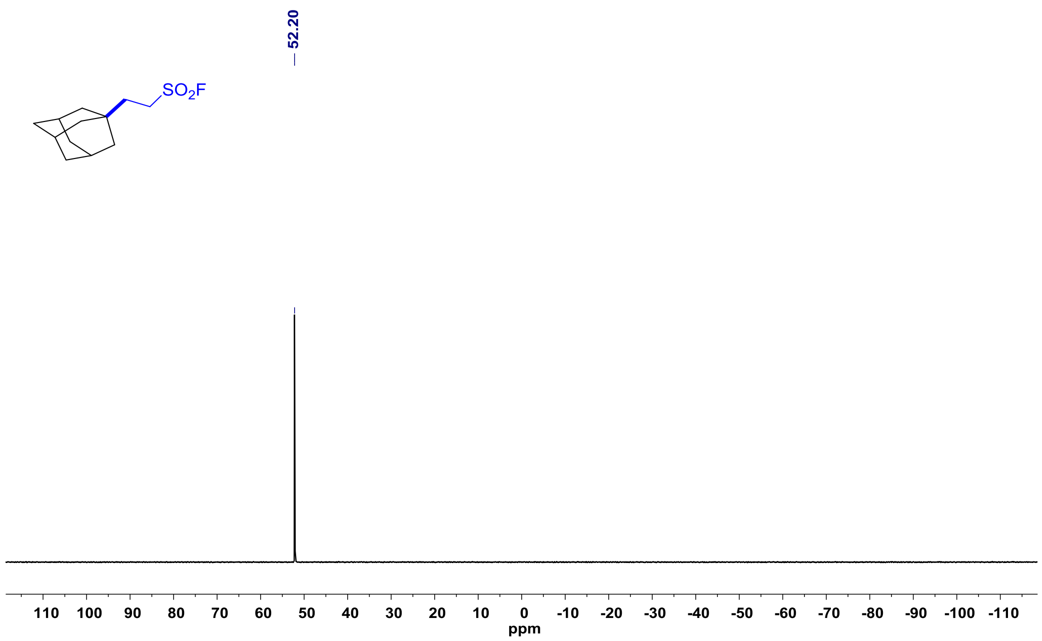

Supplementary Figure 63. ^{19}F NMR spectrum for compound **27**

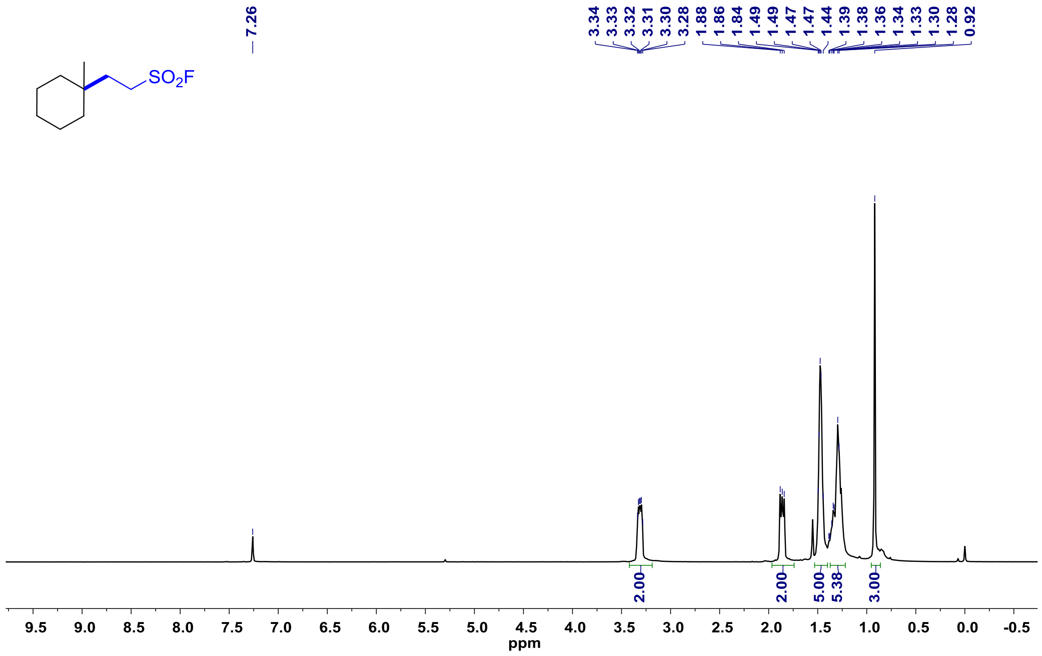

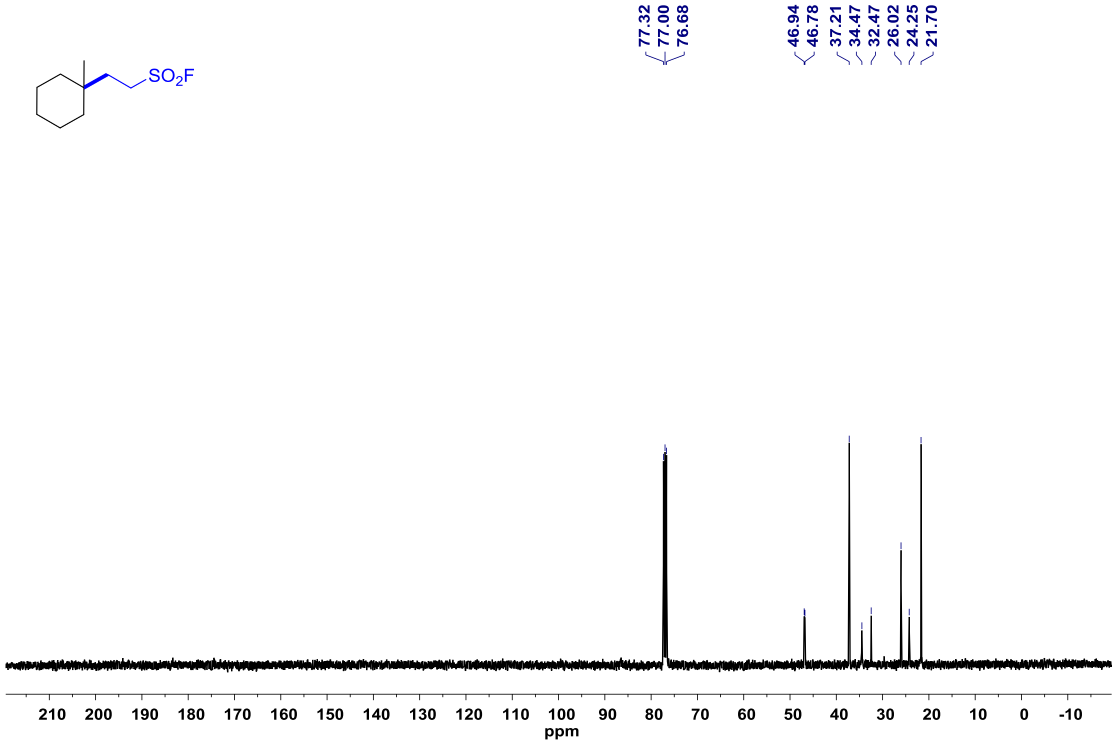

Supplementary Figure 64. ^{1}H and ^{13}C NMR spectra for compound **28**

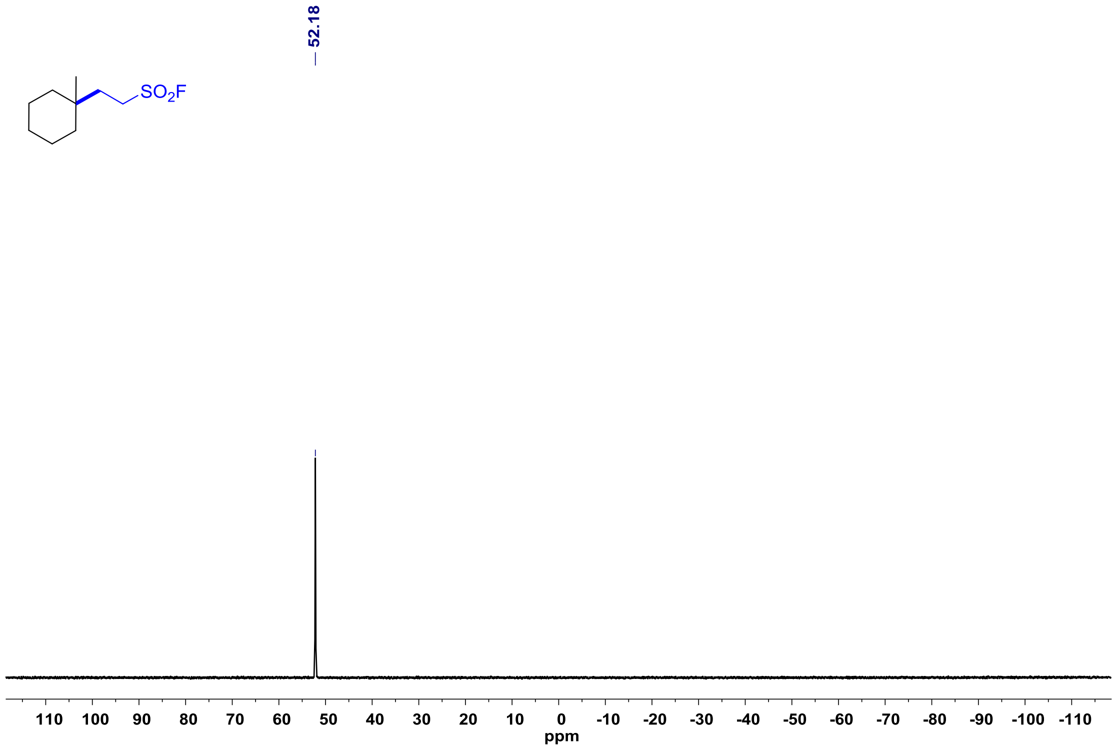

Supplementary Figure 65. ^{19}F NMR spectrum for compound **28**

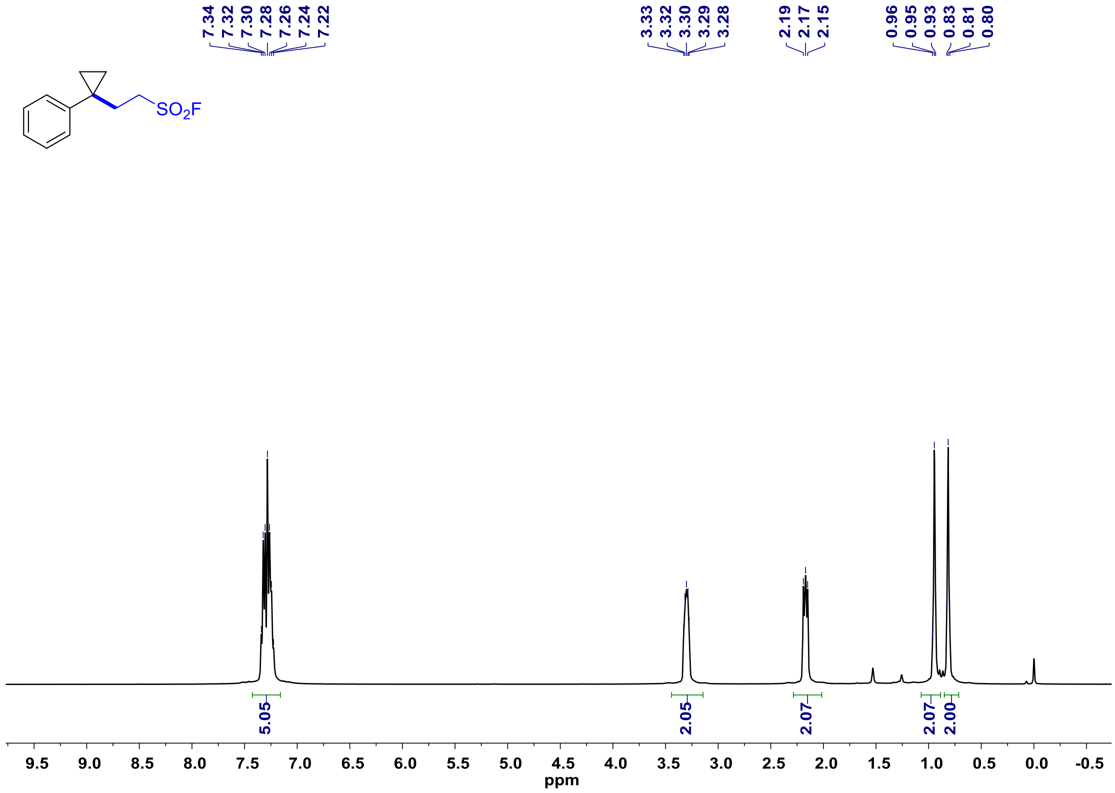

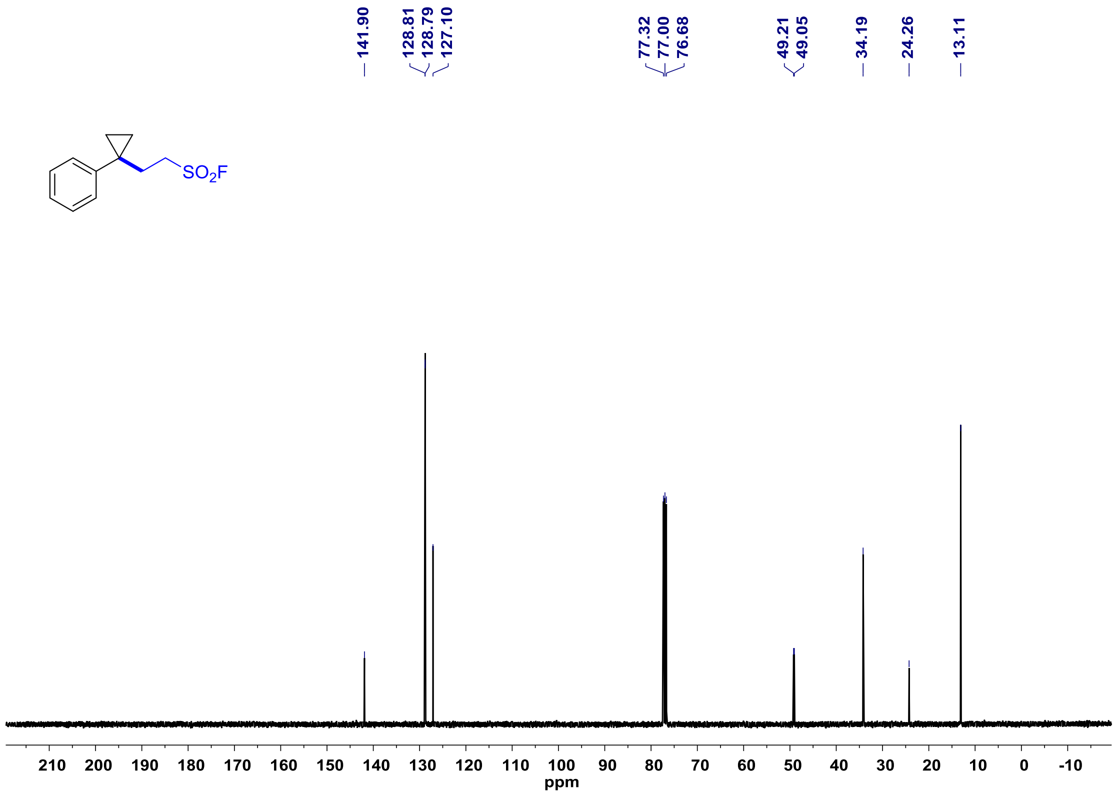

Supplementary Figure 66. ^{1}H and ^{13}C NMR spectra for compound **29**

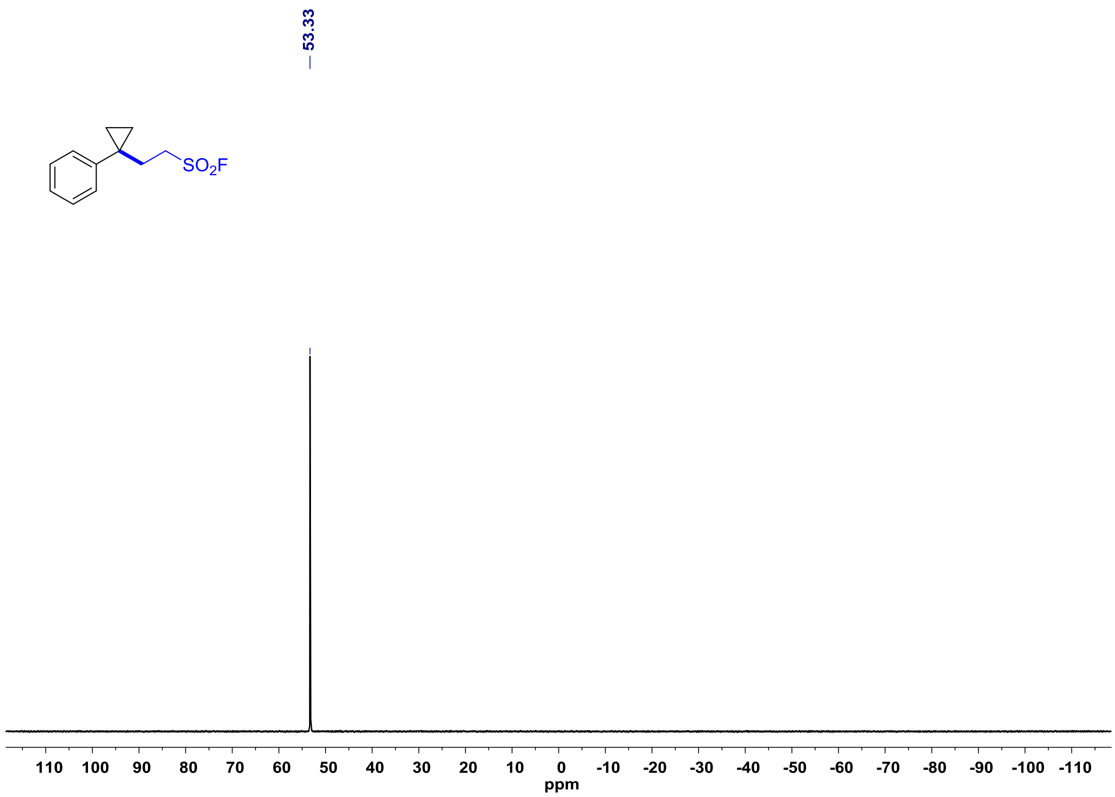

Supplementary Figure 67. ^{19}F NMR spectrum for compound **29**

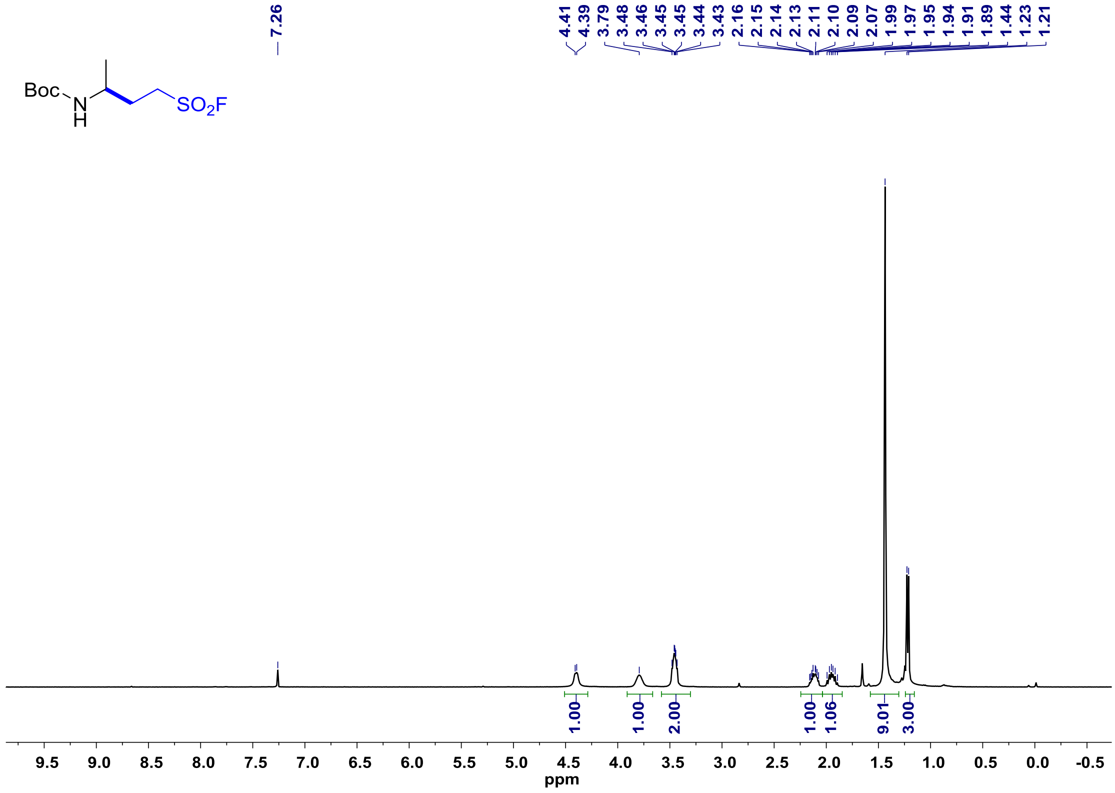

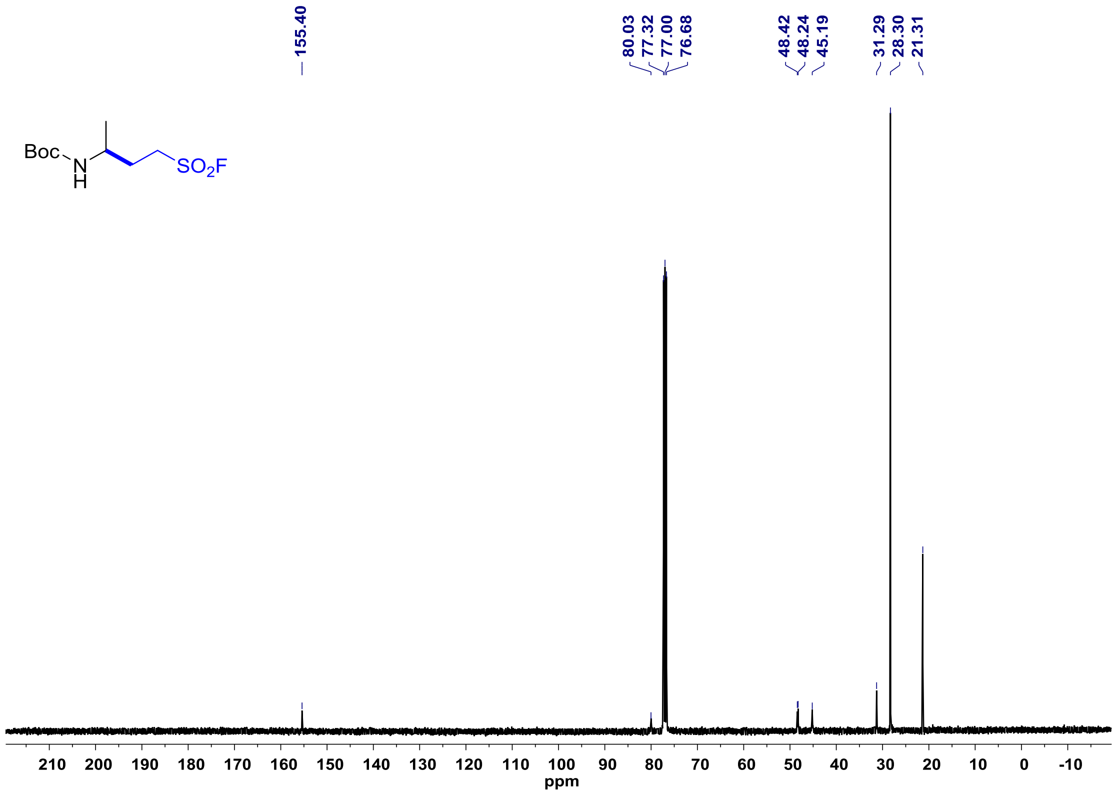

Supplementary Figure 68. ^{1}H and ^{13}C NMR spectra for compound **30**

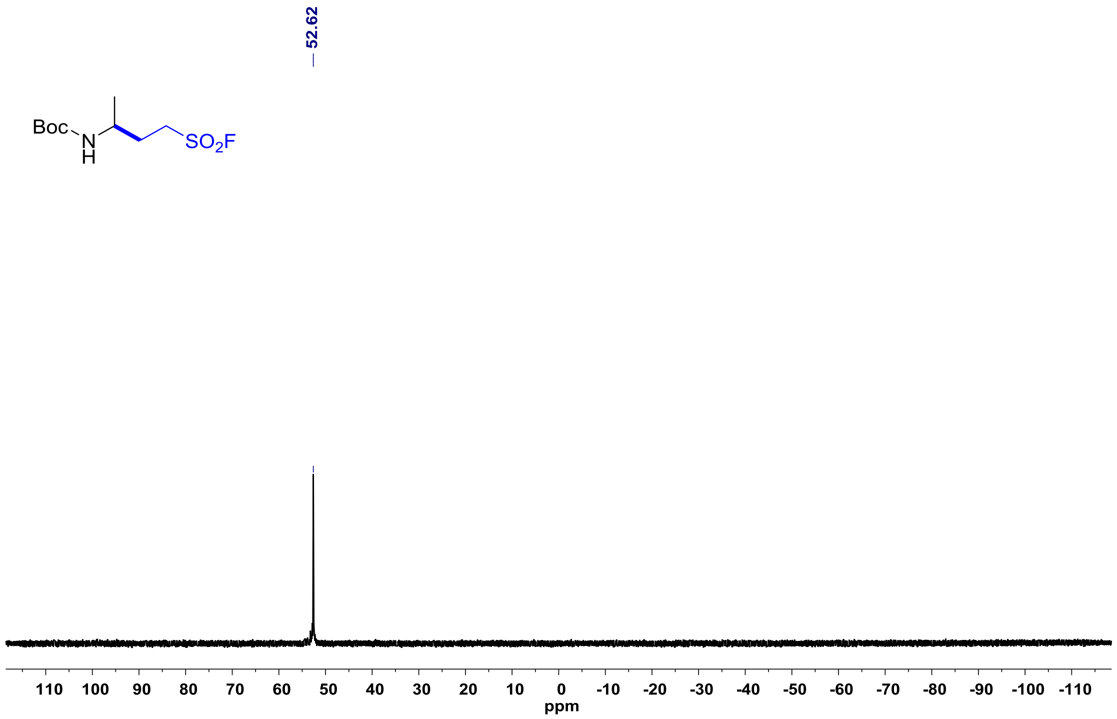

Supplementary Figure 69. ^{19}F NMR spectrum for compound **30**

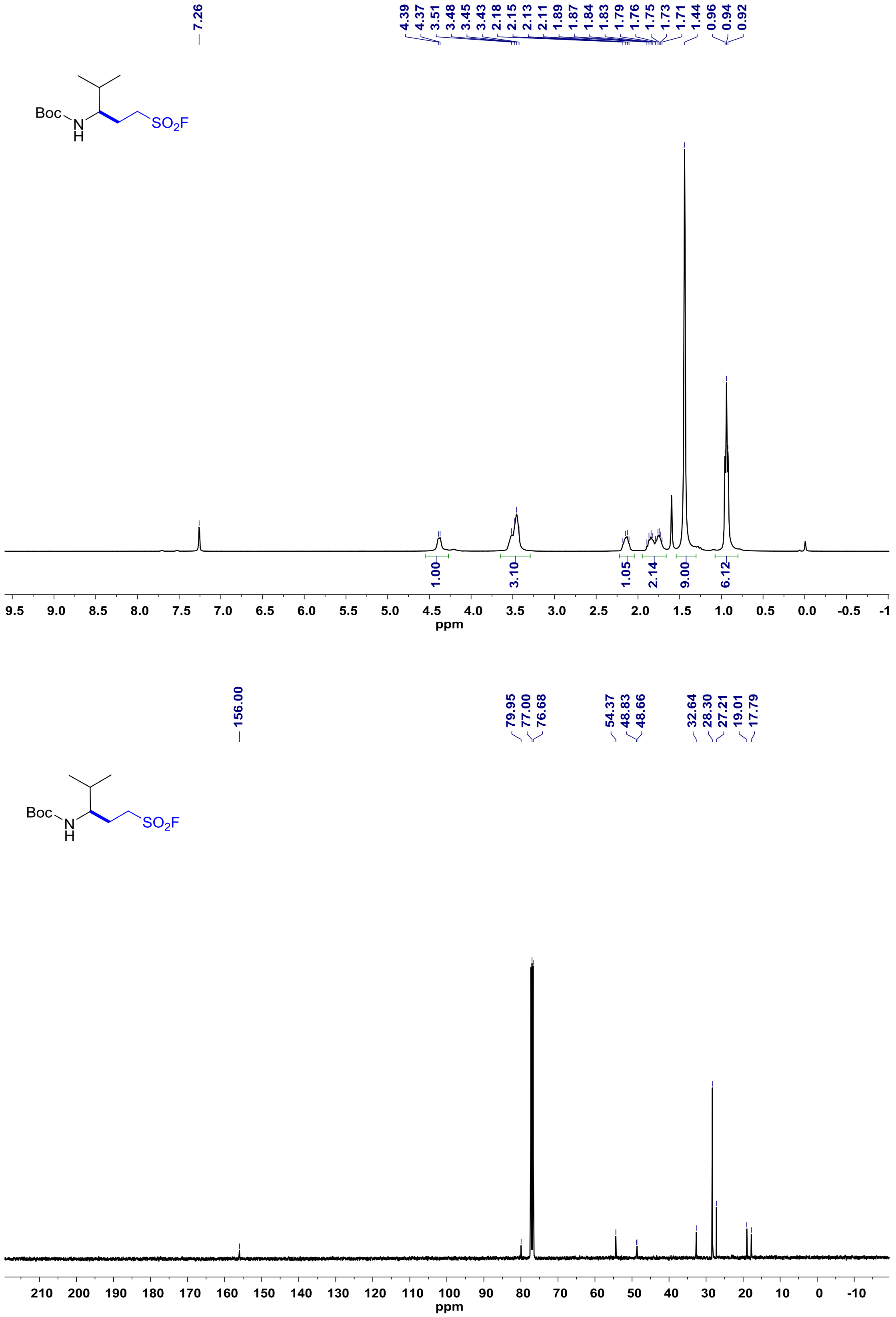

Supplementary Figure 70. ^{1}H and ^{13}C NMR spectra for compound **31**

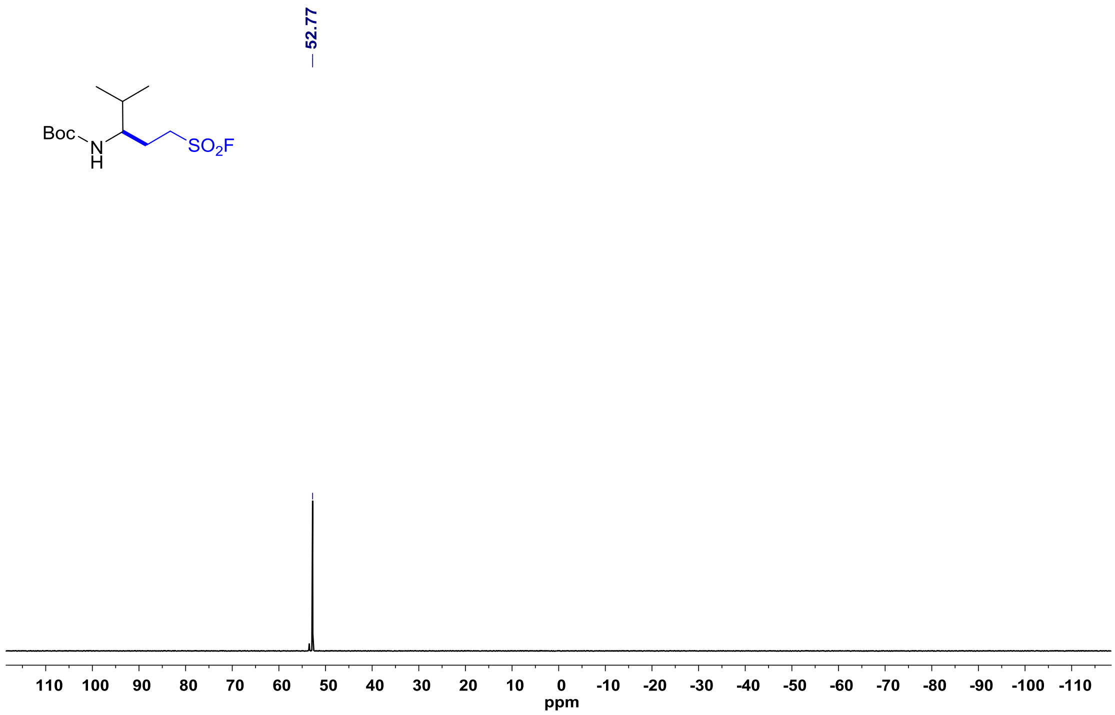

Supplementary Figure 71. ^{19}F NMR spectrum for compound **31**

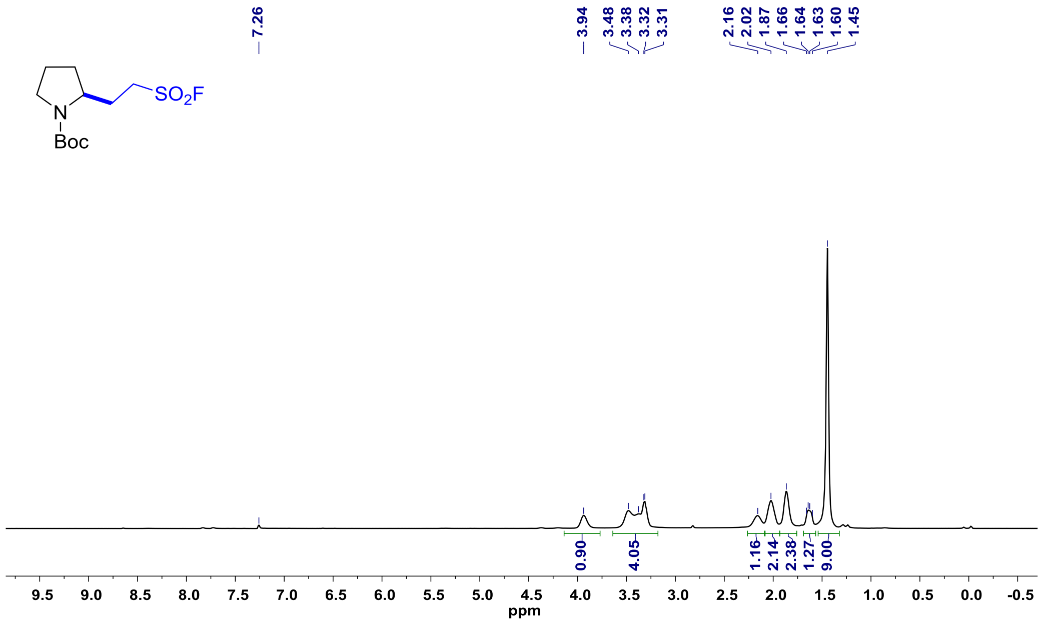

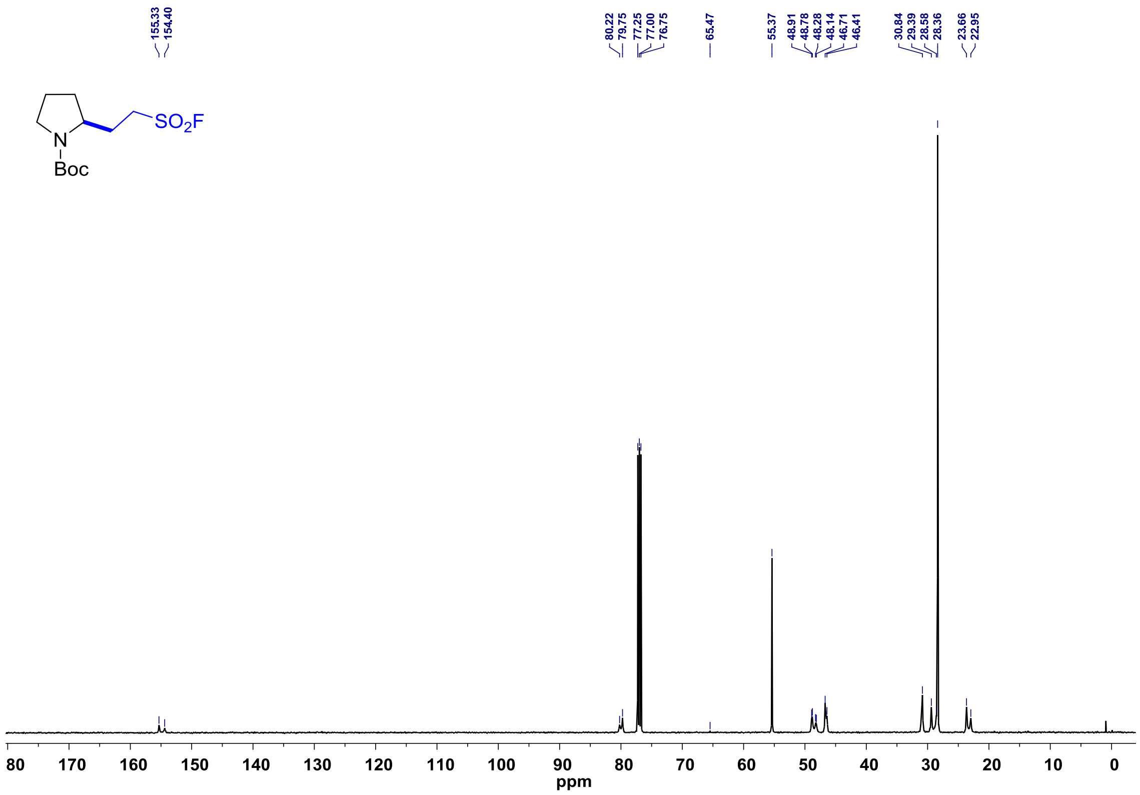

Supplementary Figure 72. ^{1}H and ^{13}C NMR spectra for compound **32**

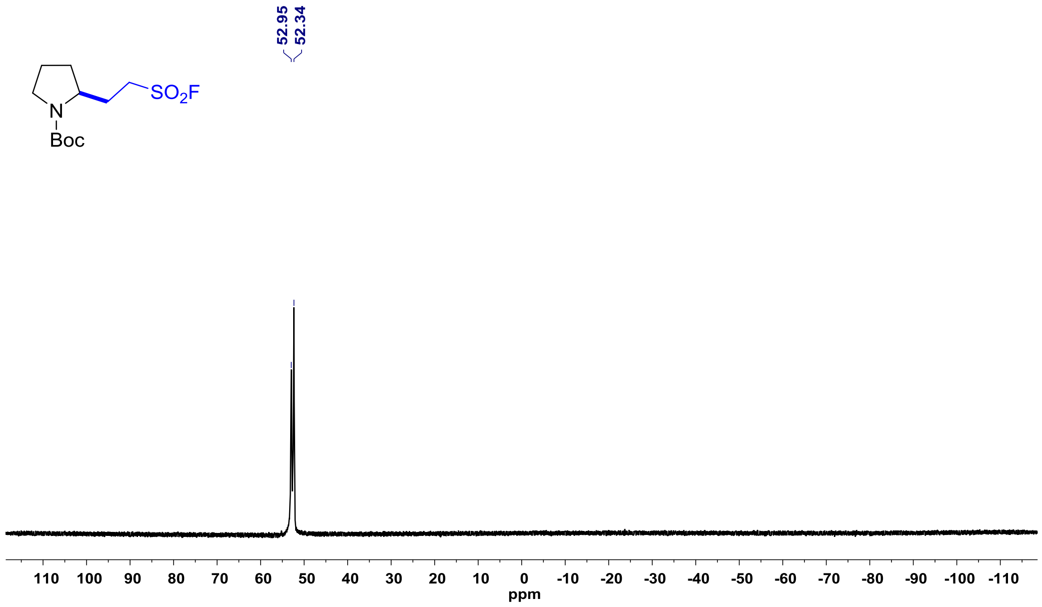

Supplementary Figure 73. ^{19}F NMR spectrum for compound **32**

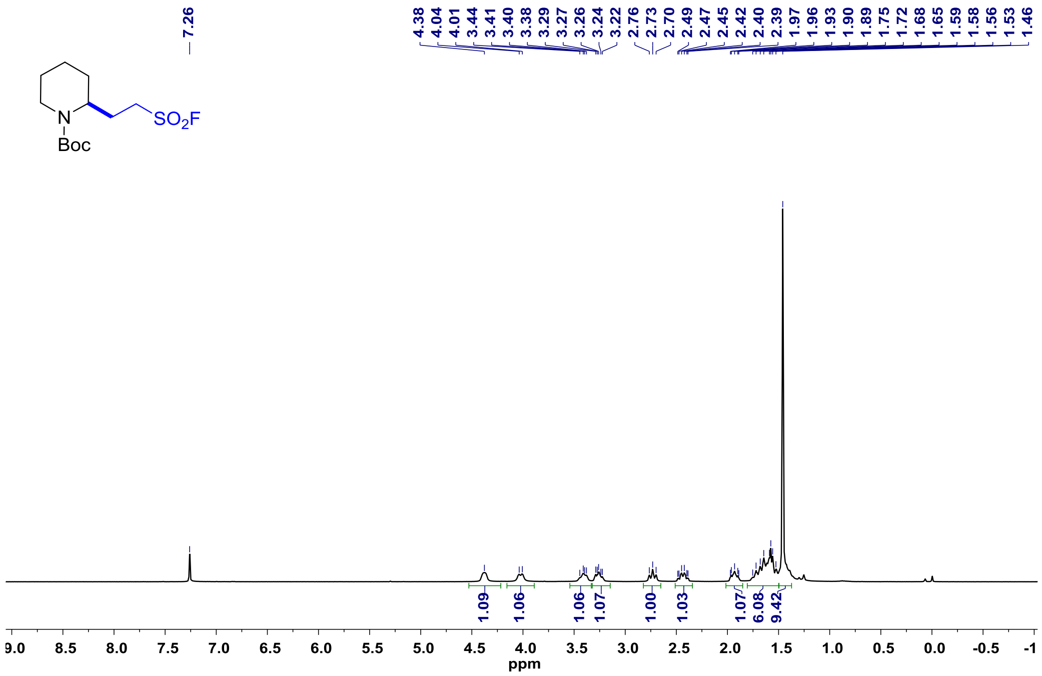

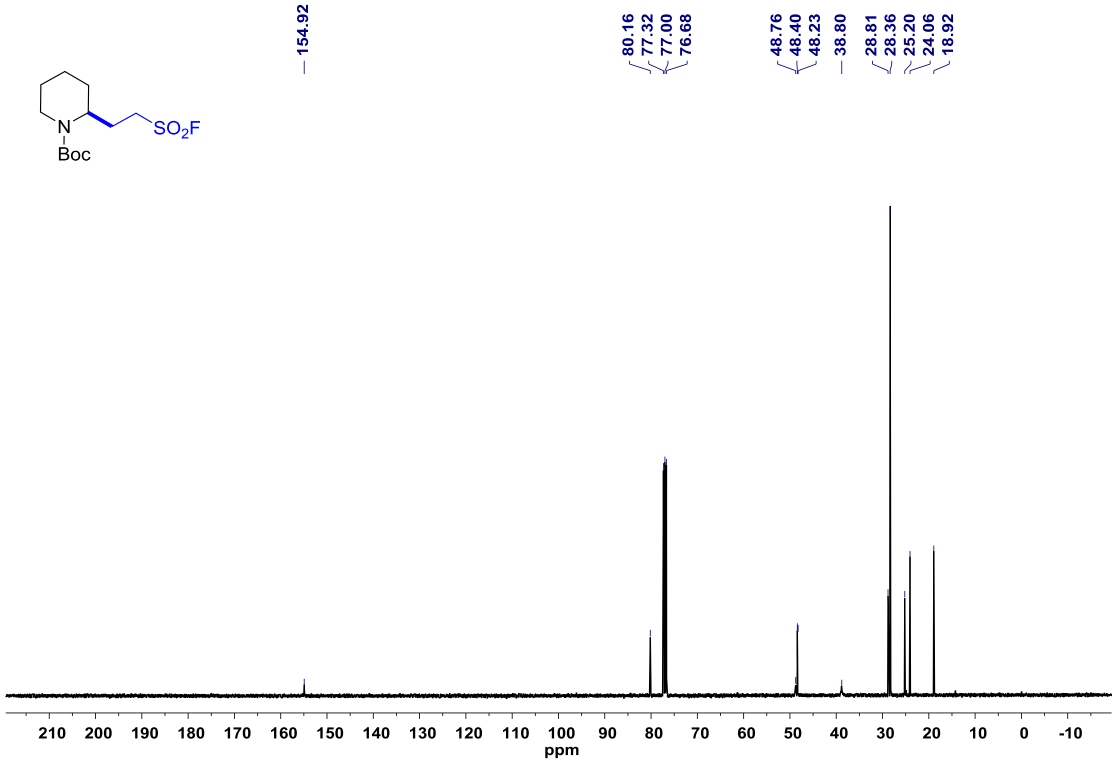

Supplementary Figure 74. ^{1}H and ^{13}C NMR spectra for compound **33**

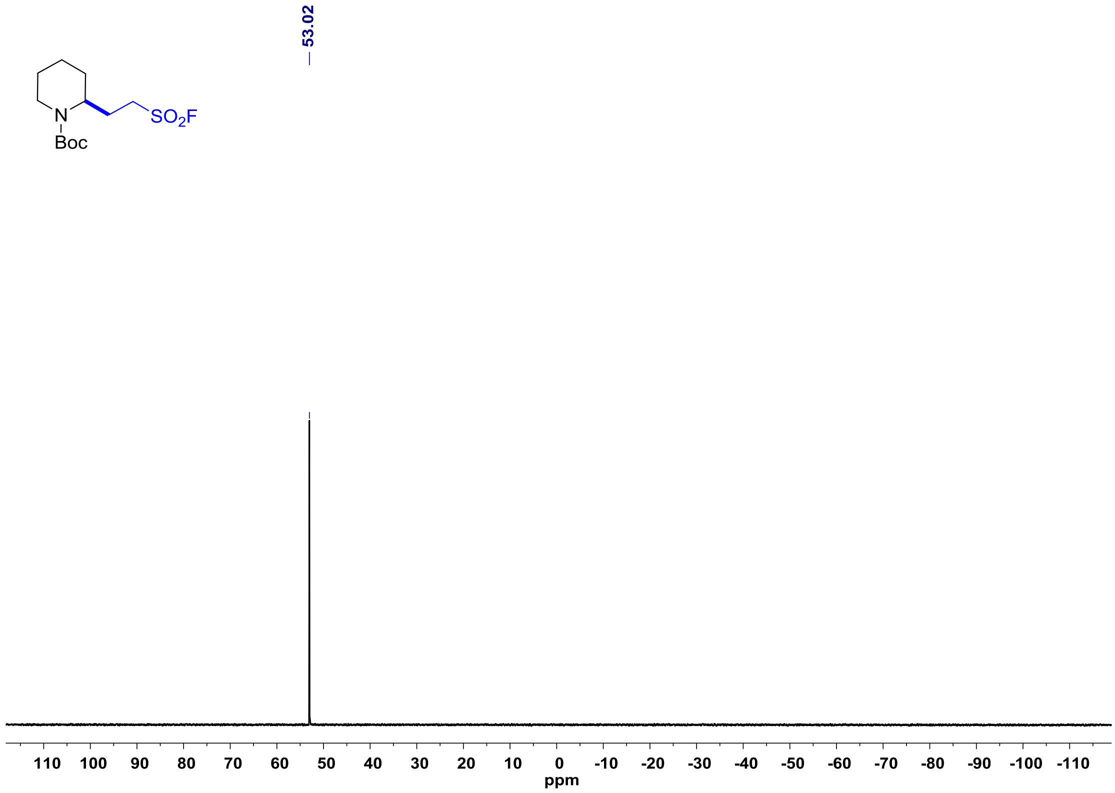

Supplementary Figure 75. ^{19}F NMR spectrum for compound **33**

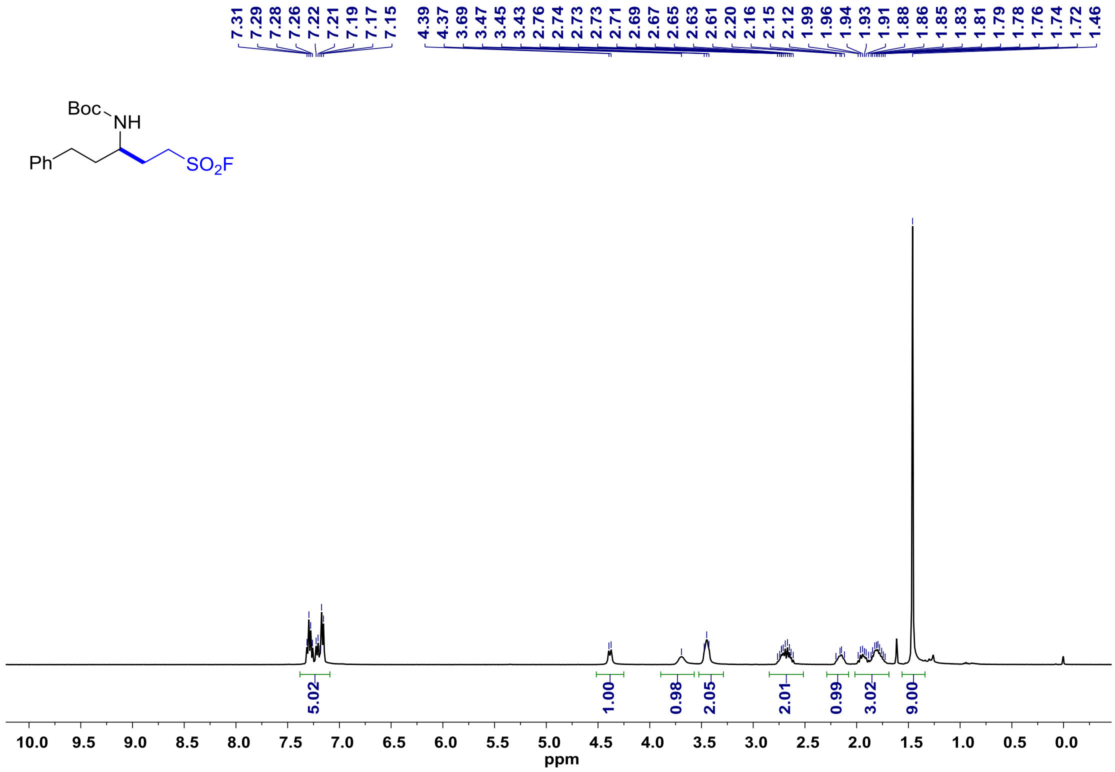

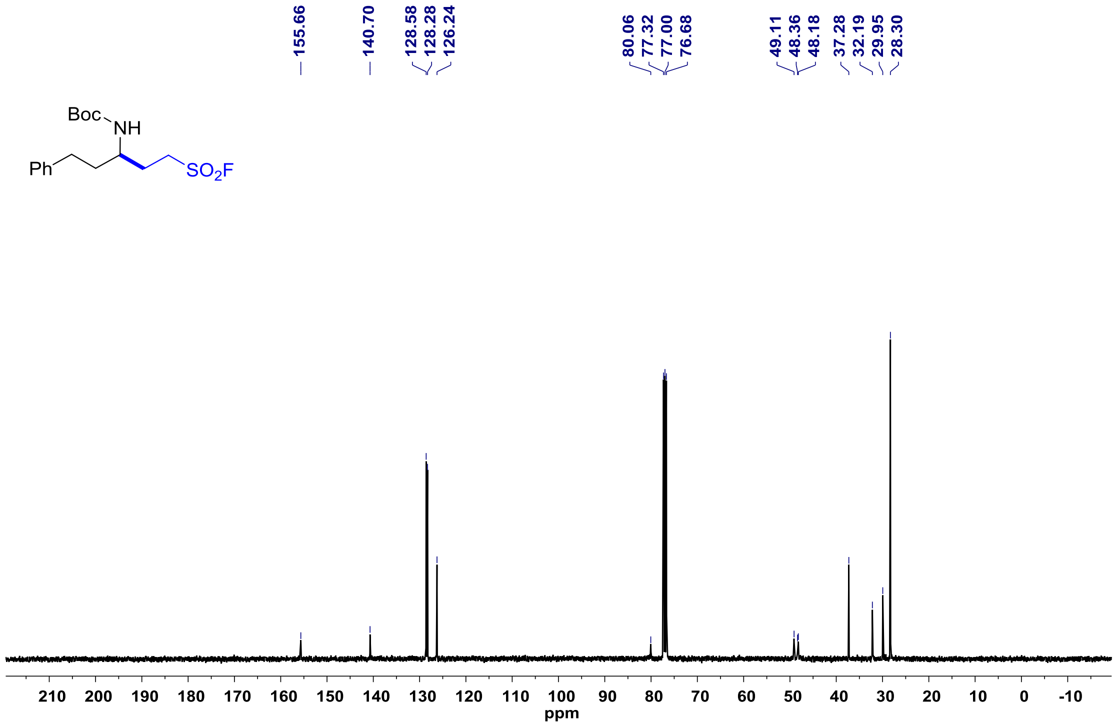

Supplementary Figure 76. ^{1}H and ^{13}C NMR spectra for compound **34**

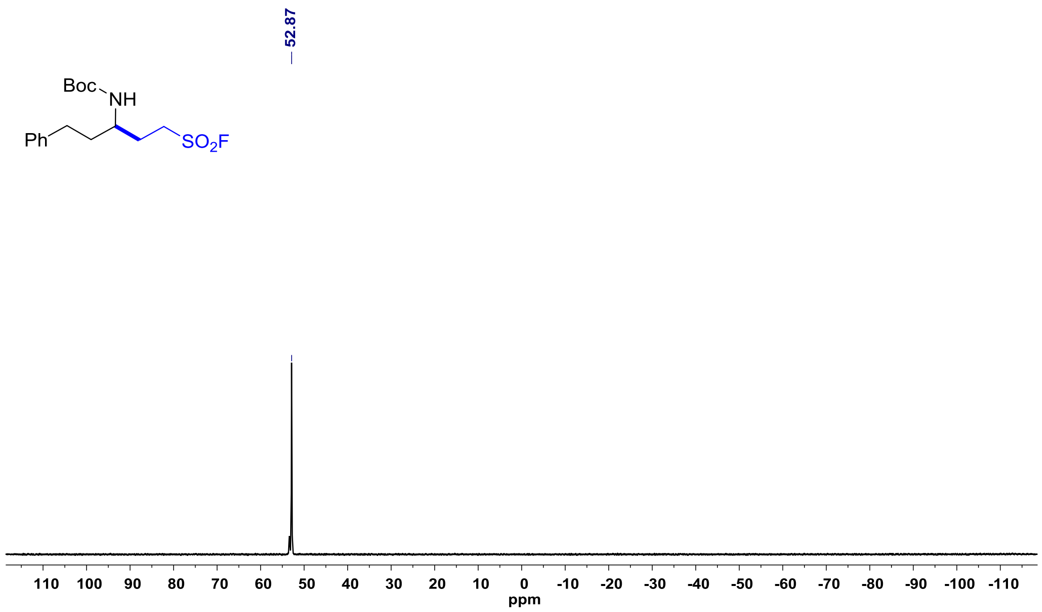

Supplementary Figure 77. ^{19}F NMR spectrum for compound **34**

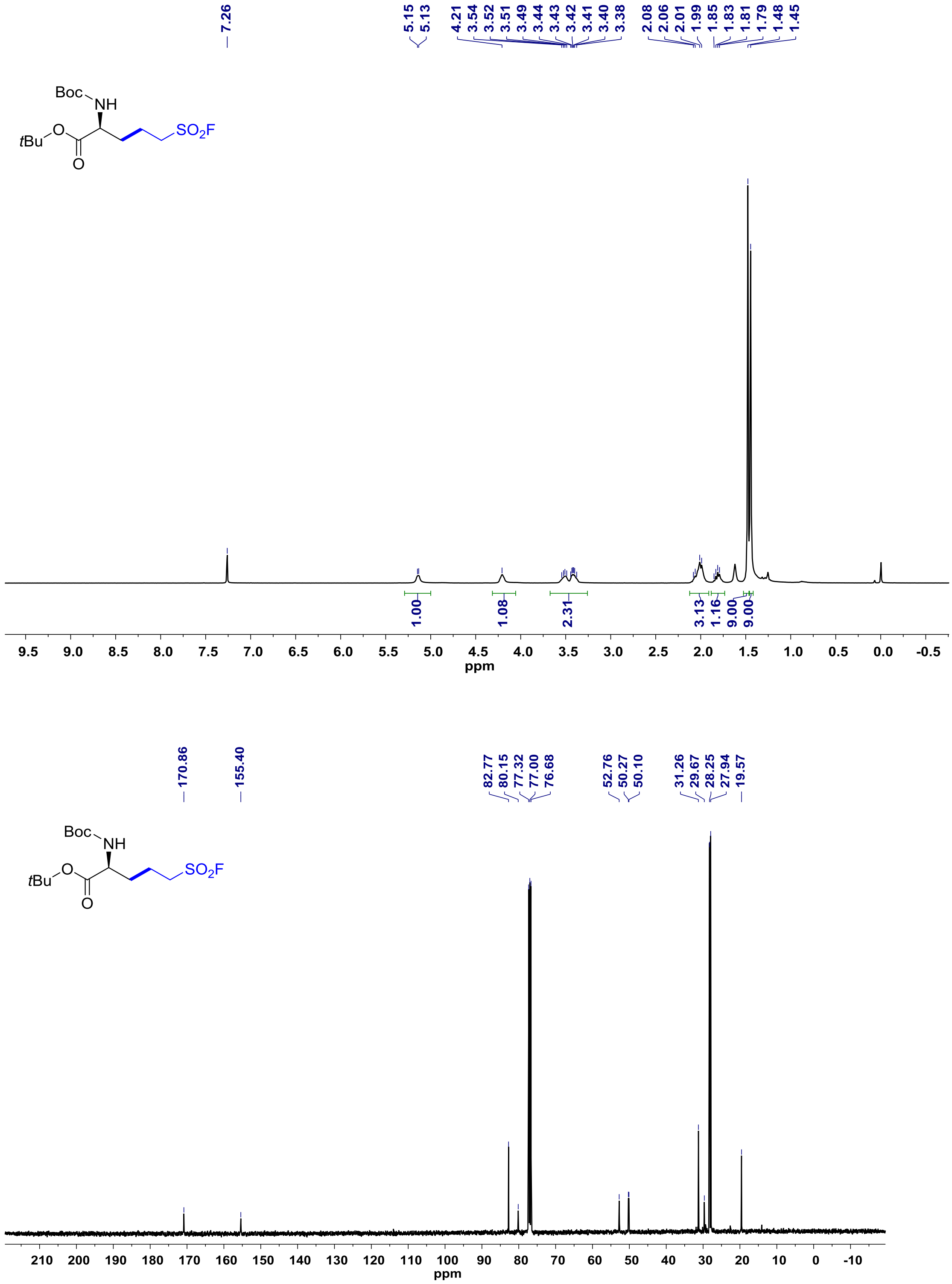

Supplementary Figure 78. ^{1}H and ^{13}C NMR spectra for compound **35**

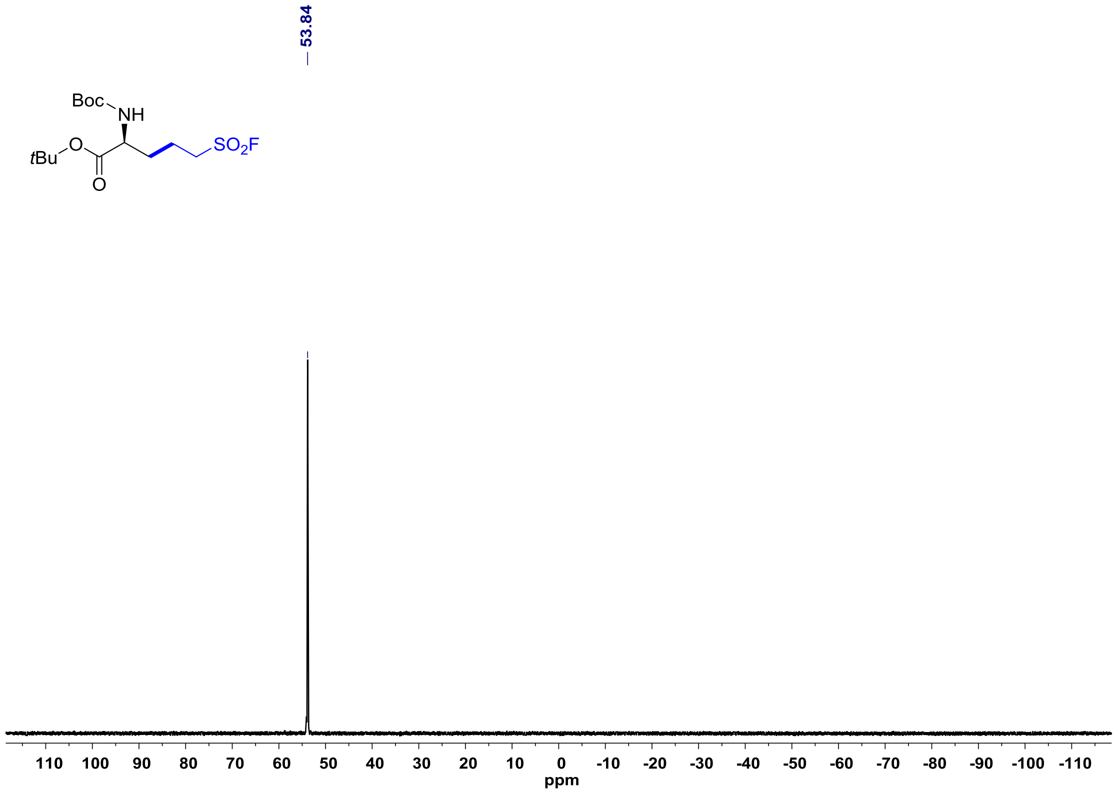

Supplementary Figure 79. ^{19}F NMR spectrum for compound **35**

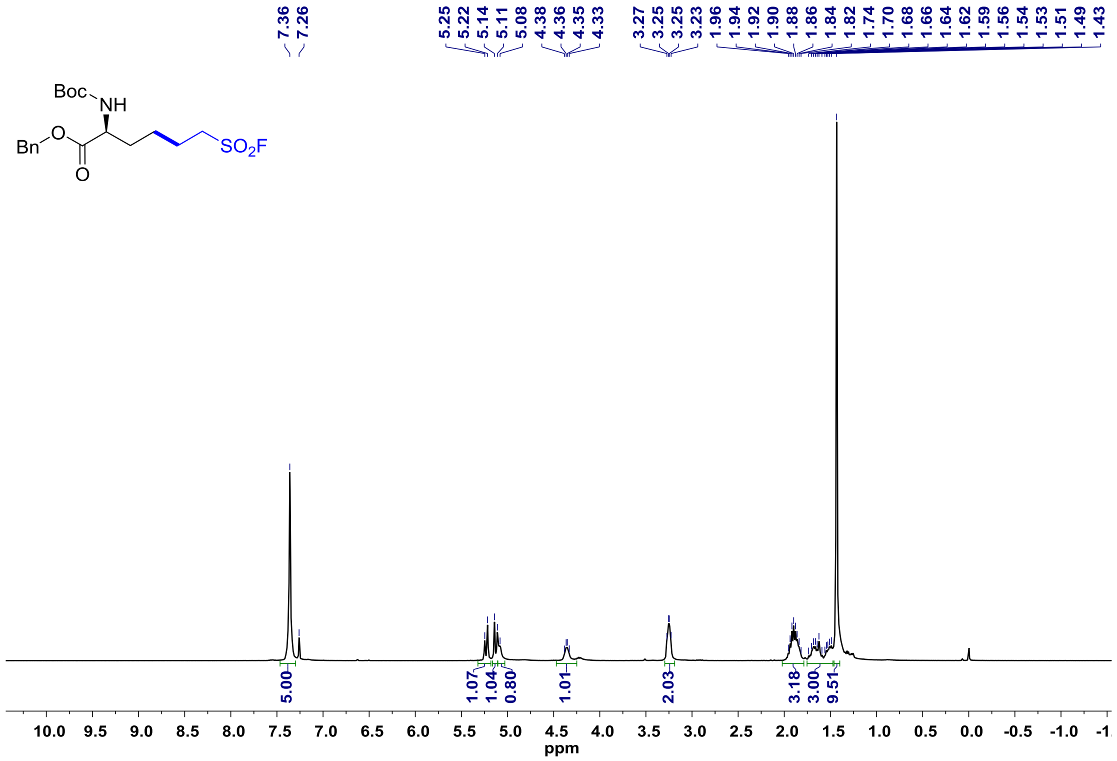

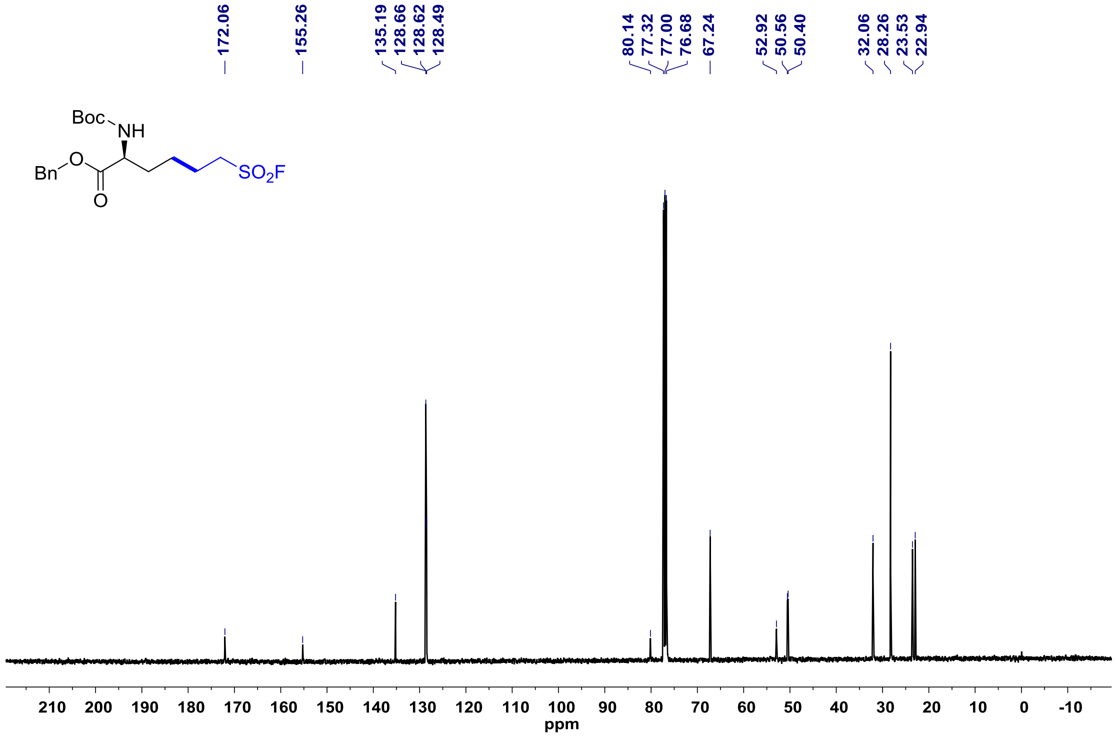

Supplementary Figure 80. ^{1}H and ^{13}C NMR spectra for compound **36**

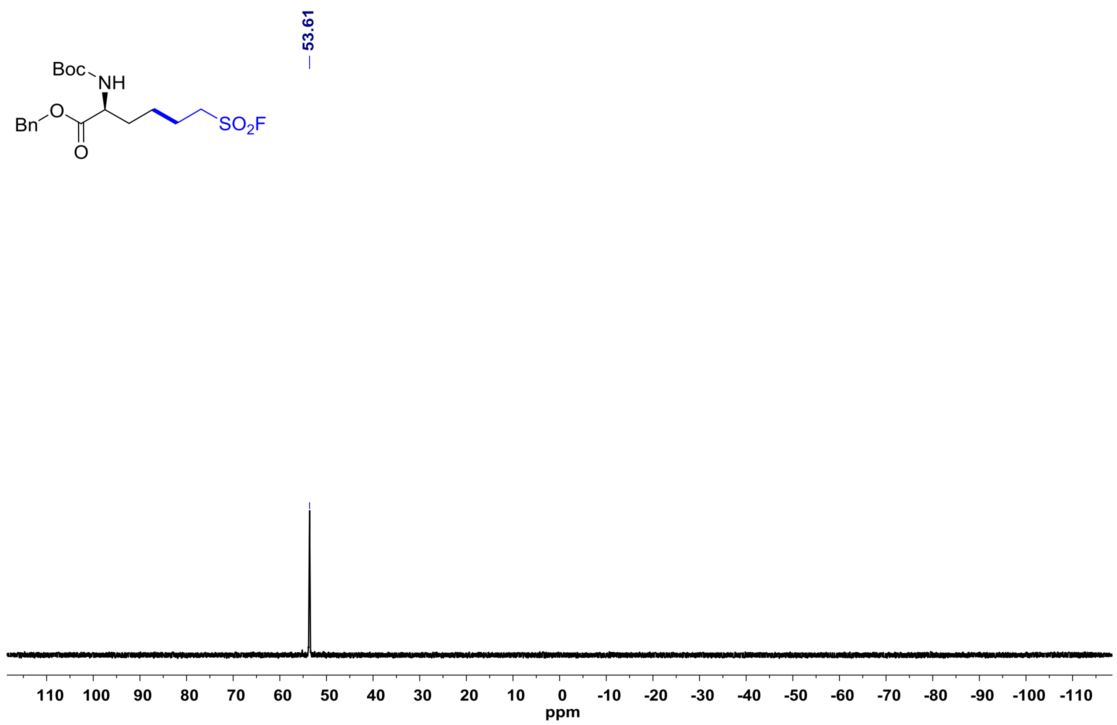

Supplementary Figure 81. ^{19}F NMR spectrum for compound **36**

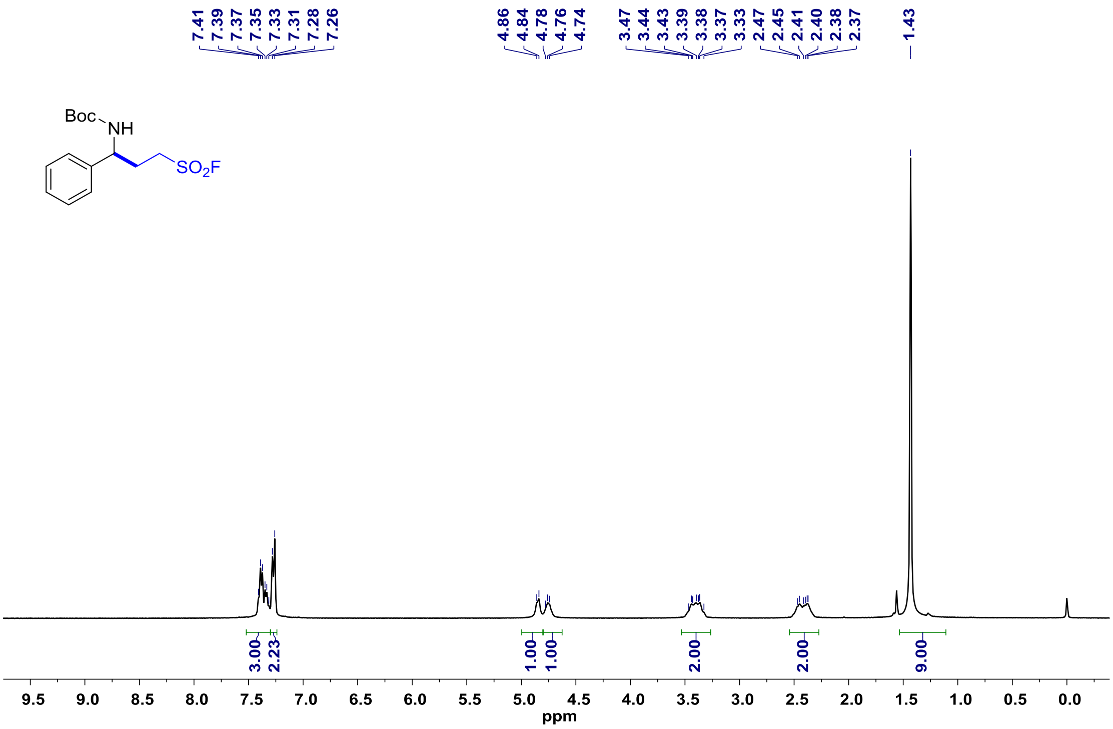

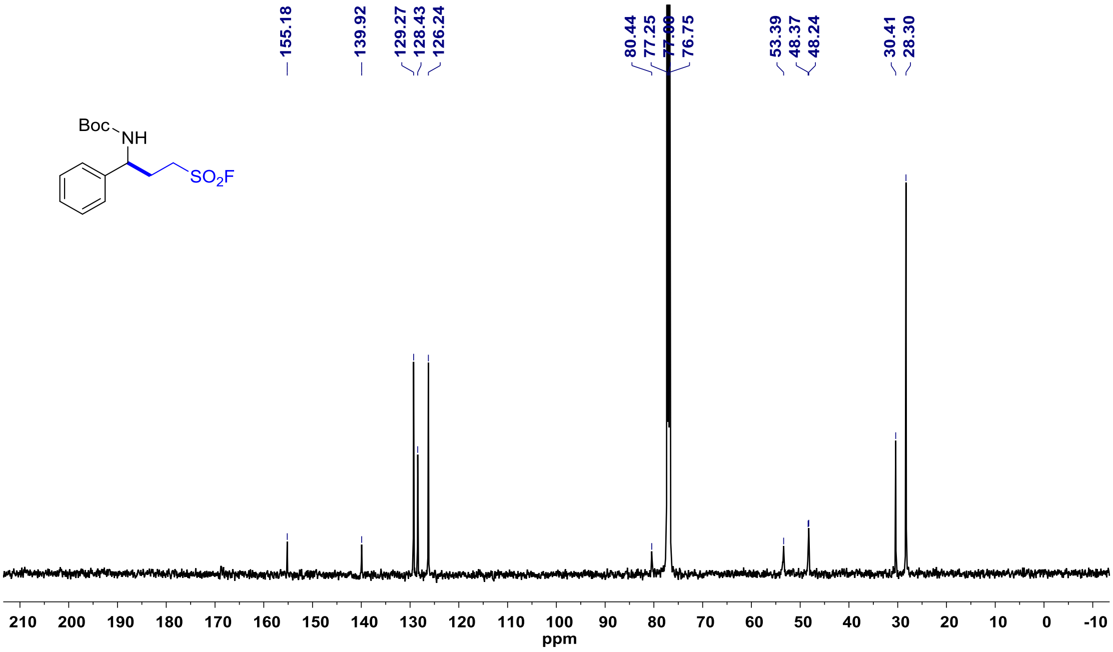

Supplementary Figure 82. ^{1}H and ^{13}C NMR spectra for compound **37**

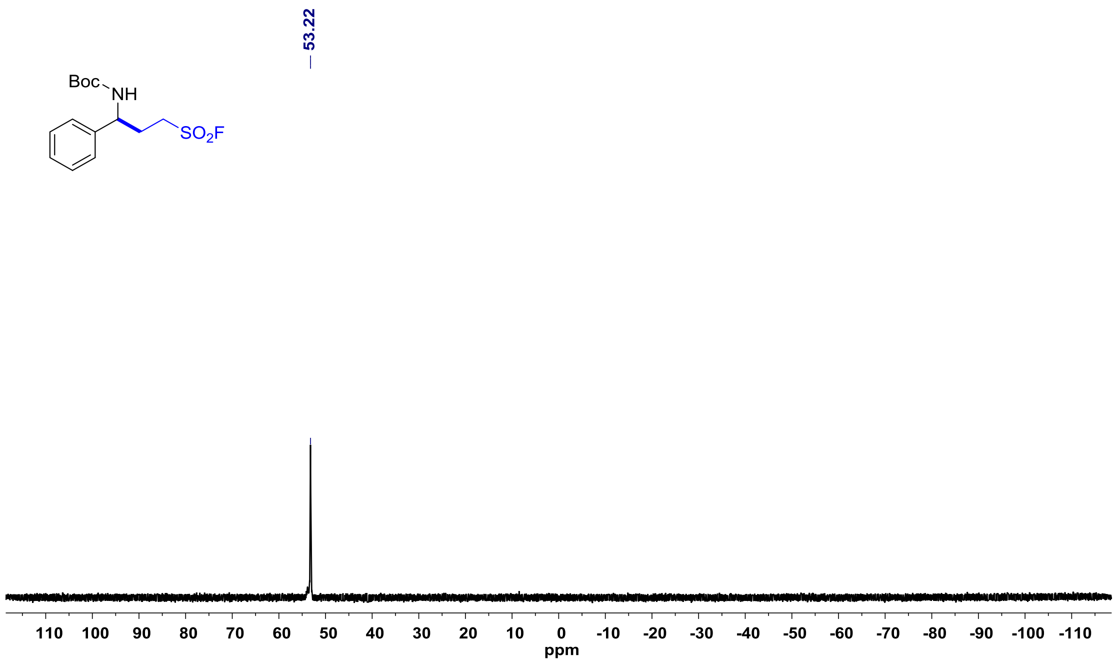

Supplementary Figure 83. ^{19}F NMR spectrum for compound **37**

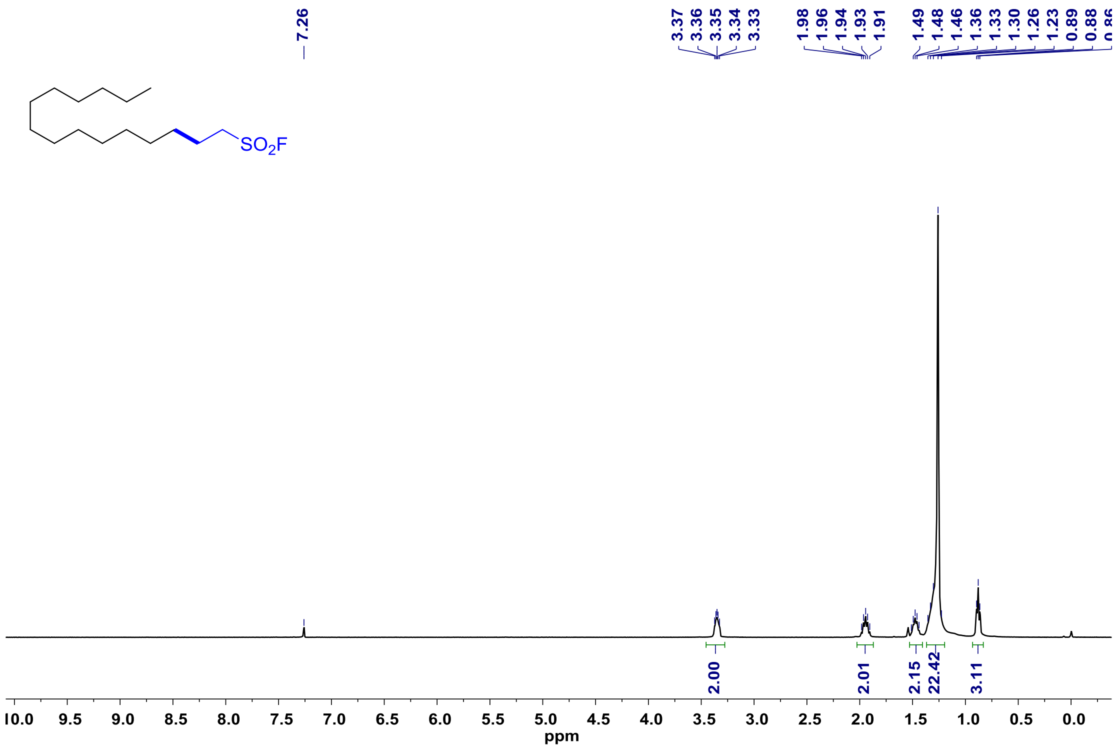

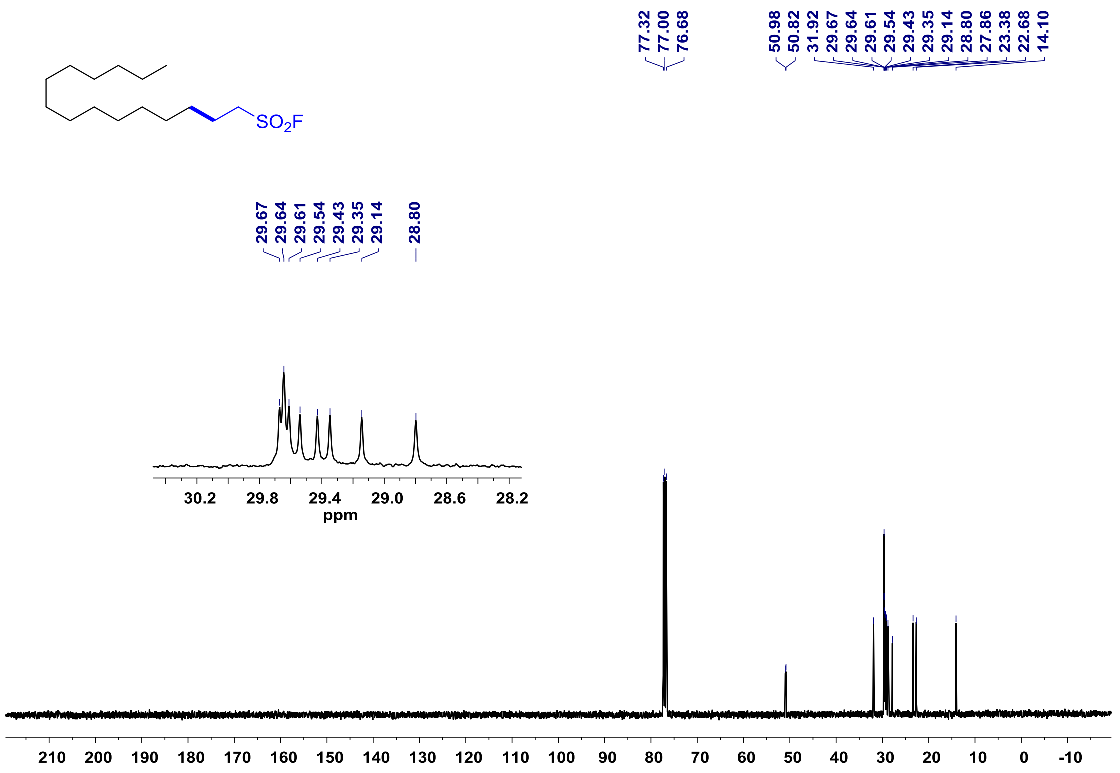

Supplementary Figure 84. ^{1}H and ^{13}C NMR spectra for compound **38**

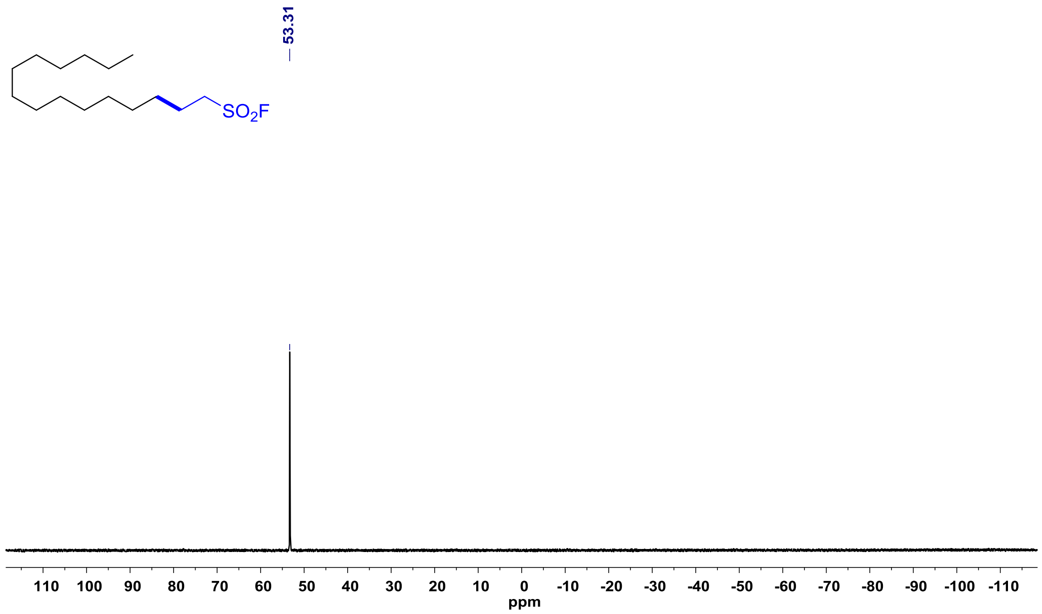

Supplementary Figure 85. ^{19}F NMR spectrum for compound **38**

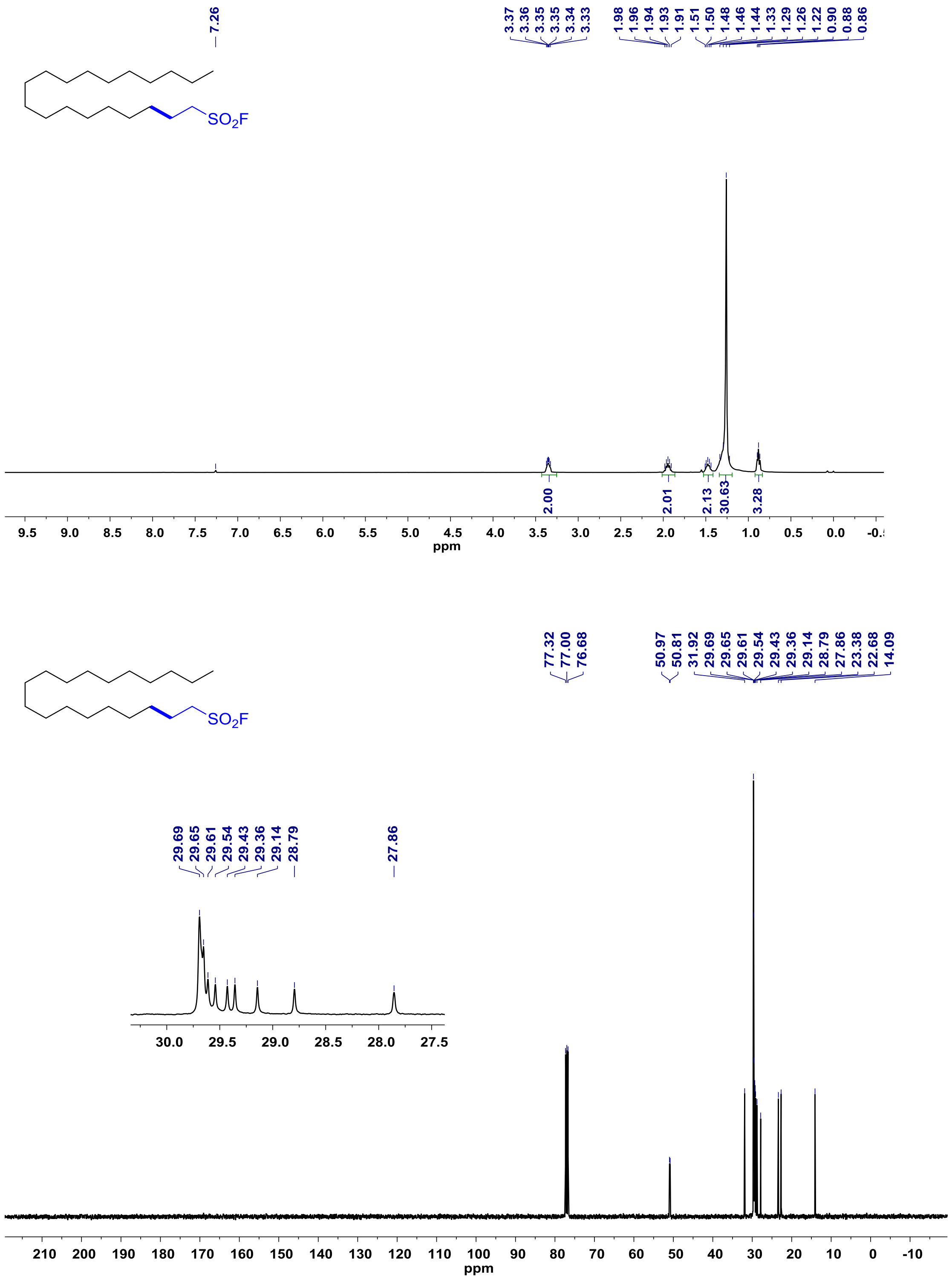

Supplementary Figure 86. ^{1}H and ^{13}C NMR spectra for compound **39**

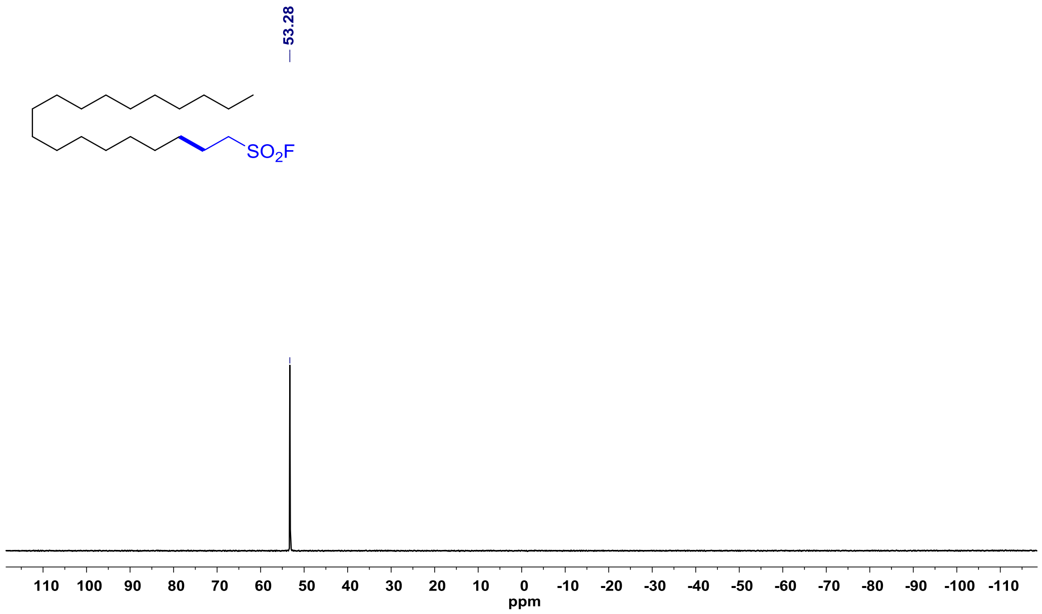

Supplementary Figure 87. ^{19}F NMR spectrum for compound **39**

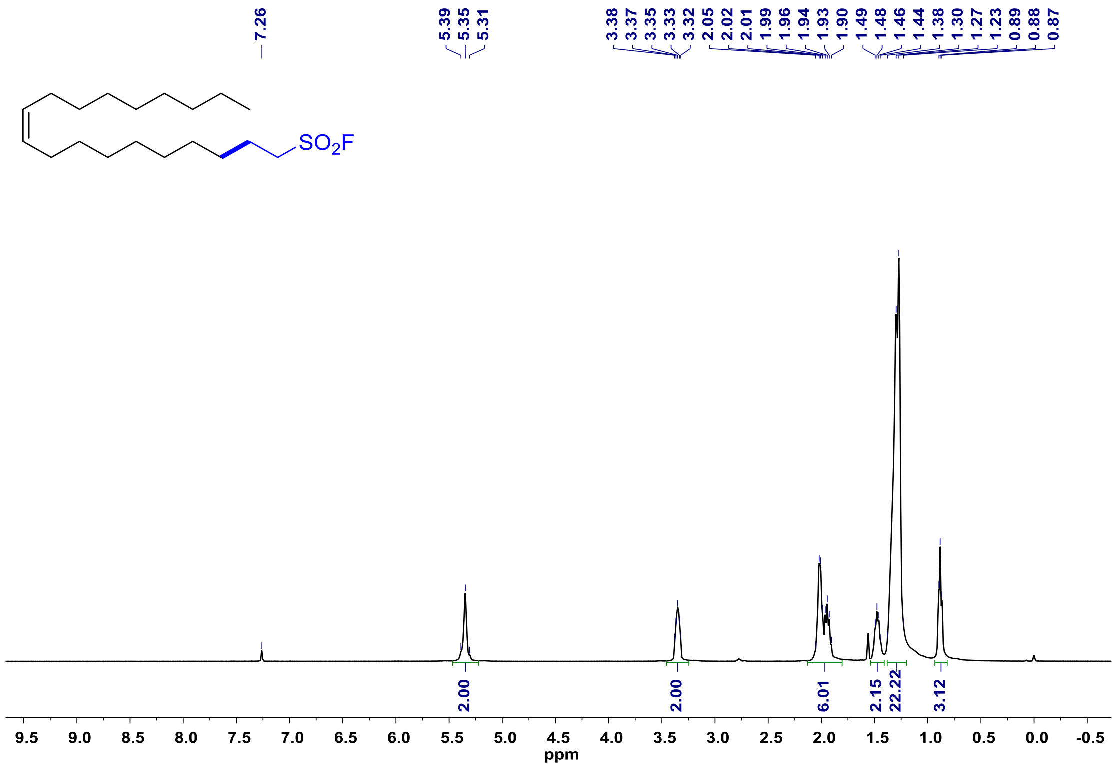

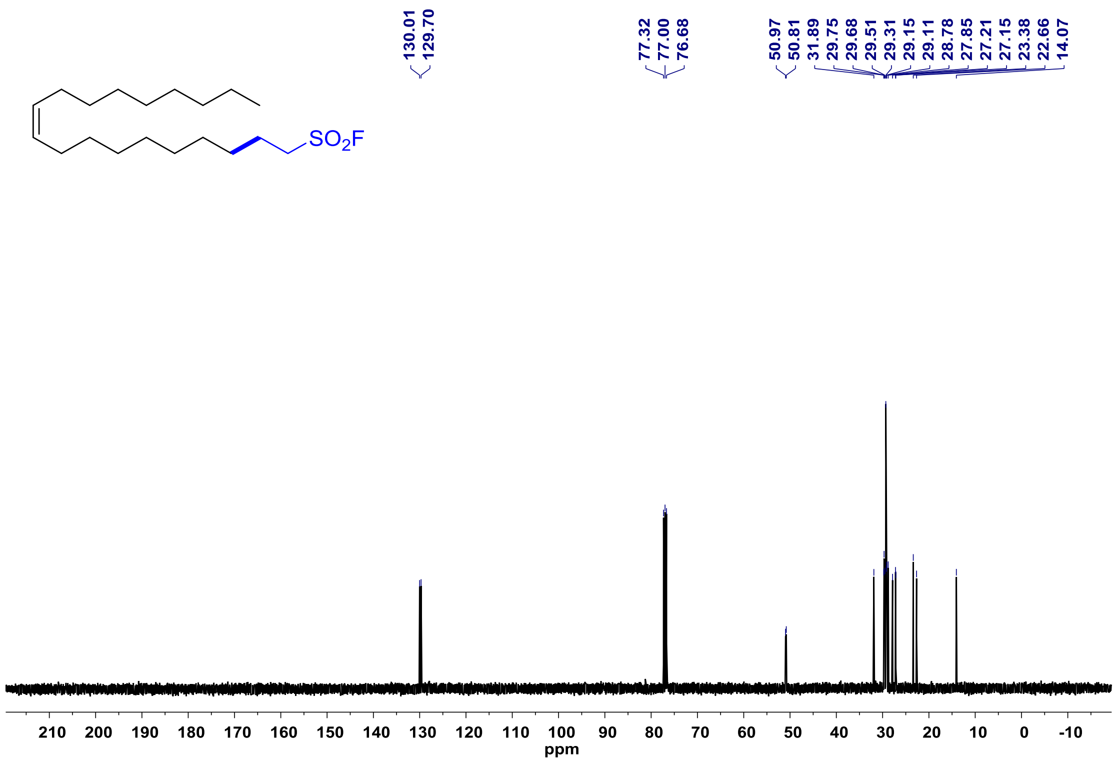

Supplementary Figure 88. ^{1}H and ^{13}C NMR spectra for compound **40**

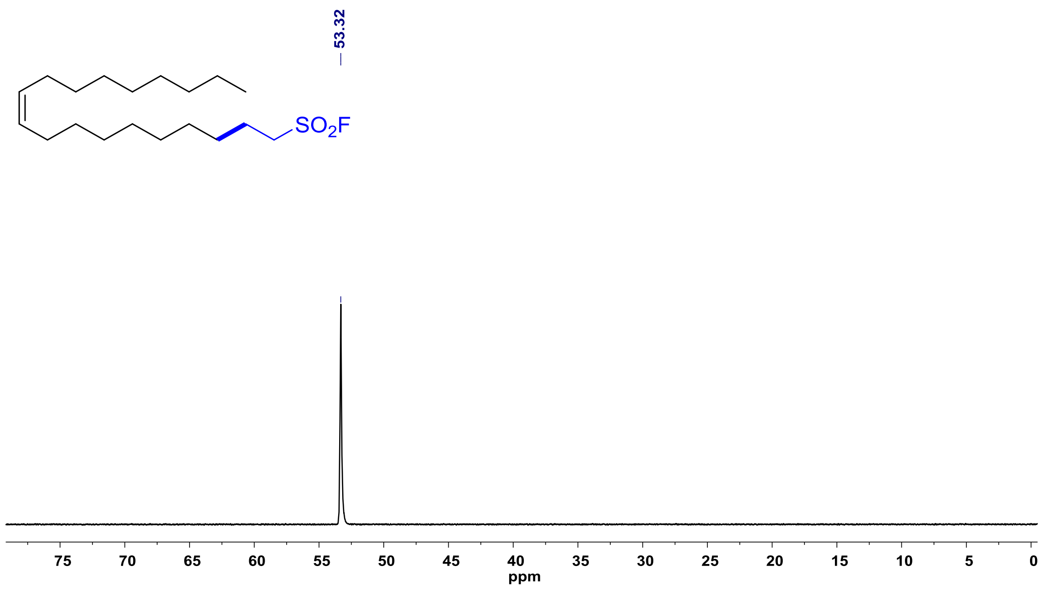

Supplementary Figure 89. ^{19}F NMR spectrum for compound **40**

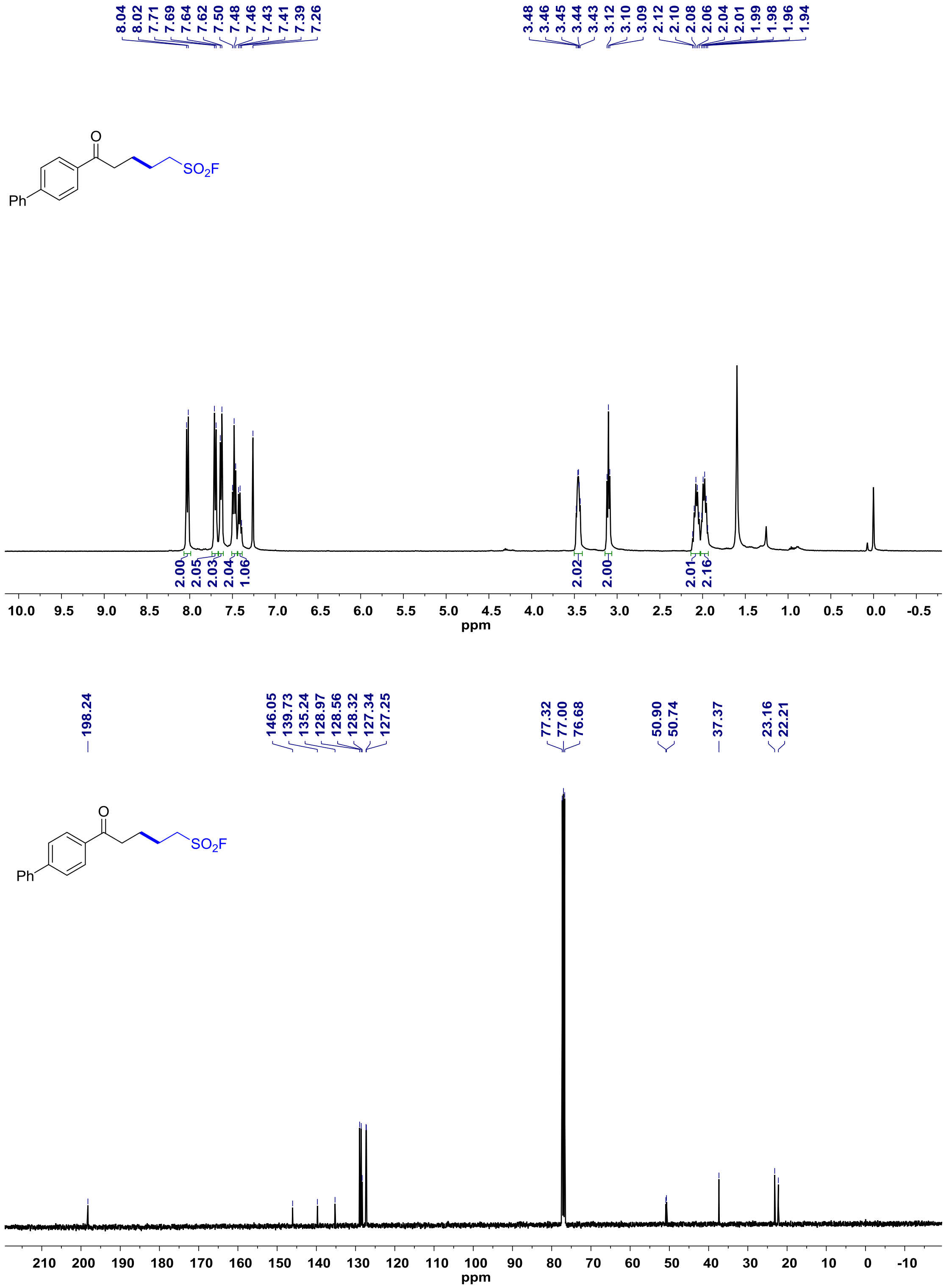

Supplementary Figure 90. ^{1}H and ^{13}C NMR spectra for compound **41**

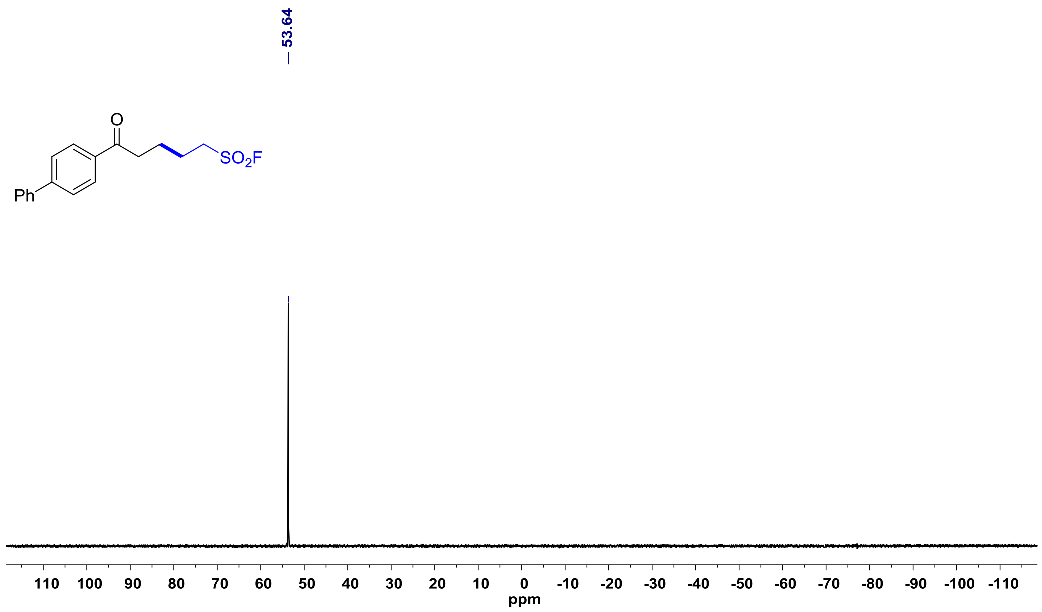

Supplementary Figure 91. ^{19}F NMR spectrum for compound **41**

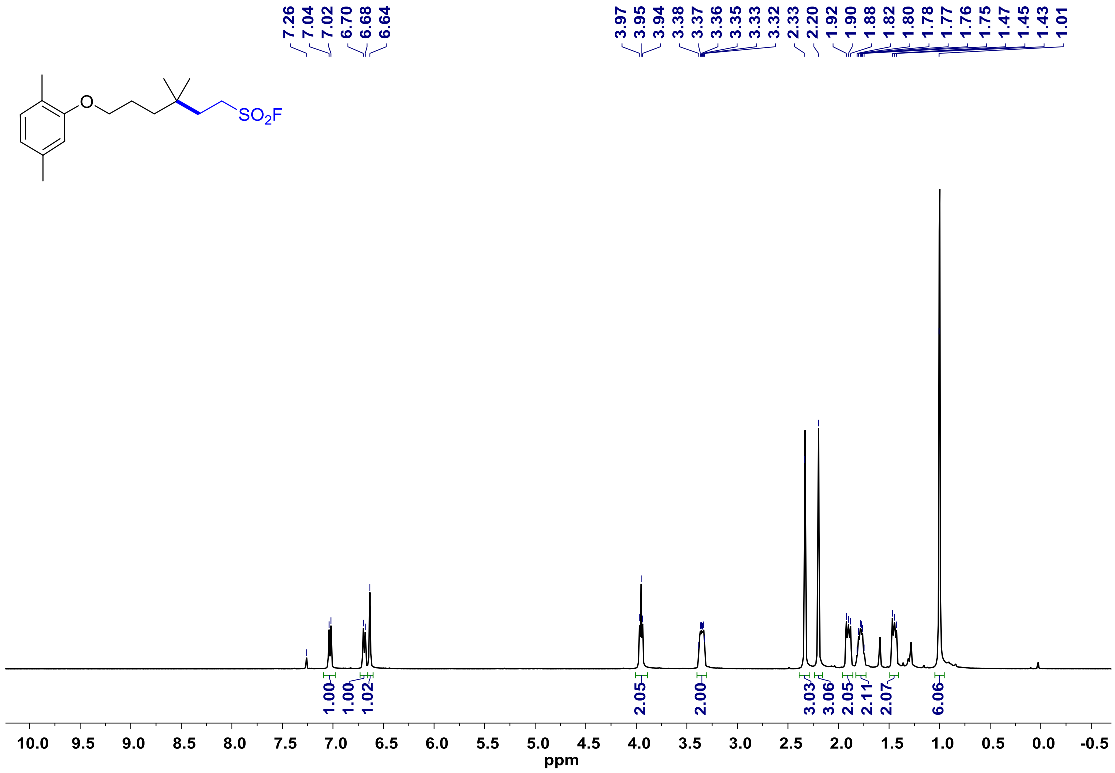

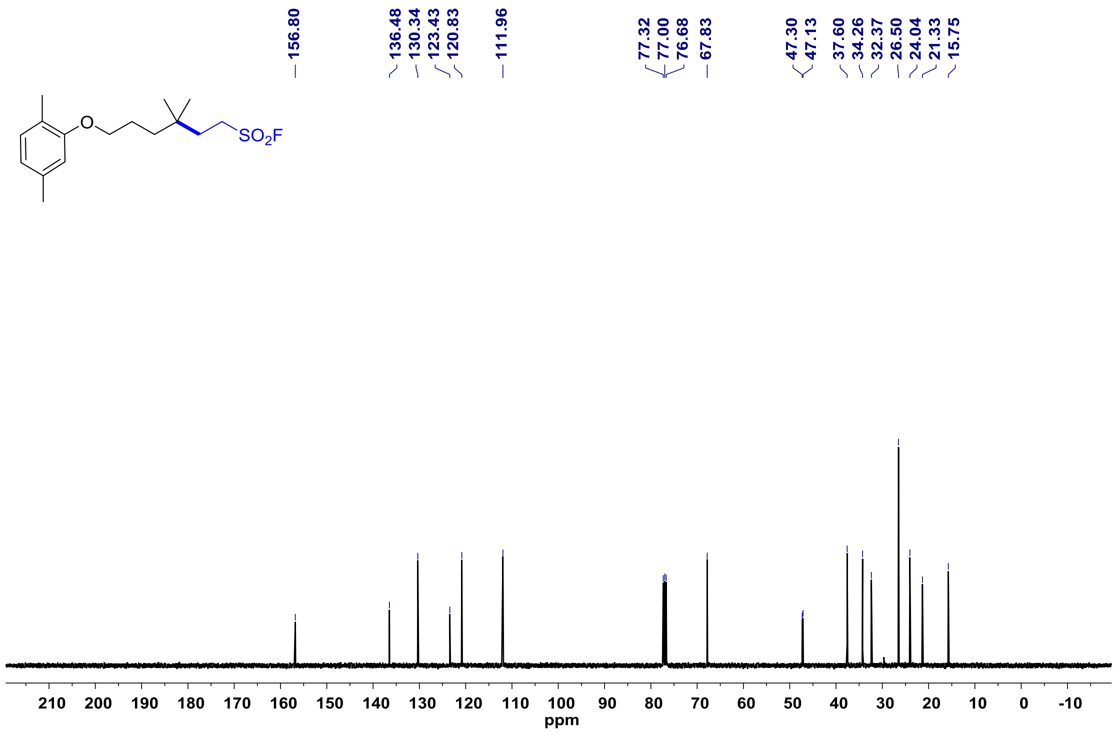

Supplementary Figure 92. ^{1}H and ^{13}C NMR spectra for compound **42**

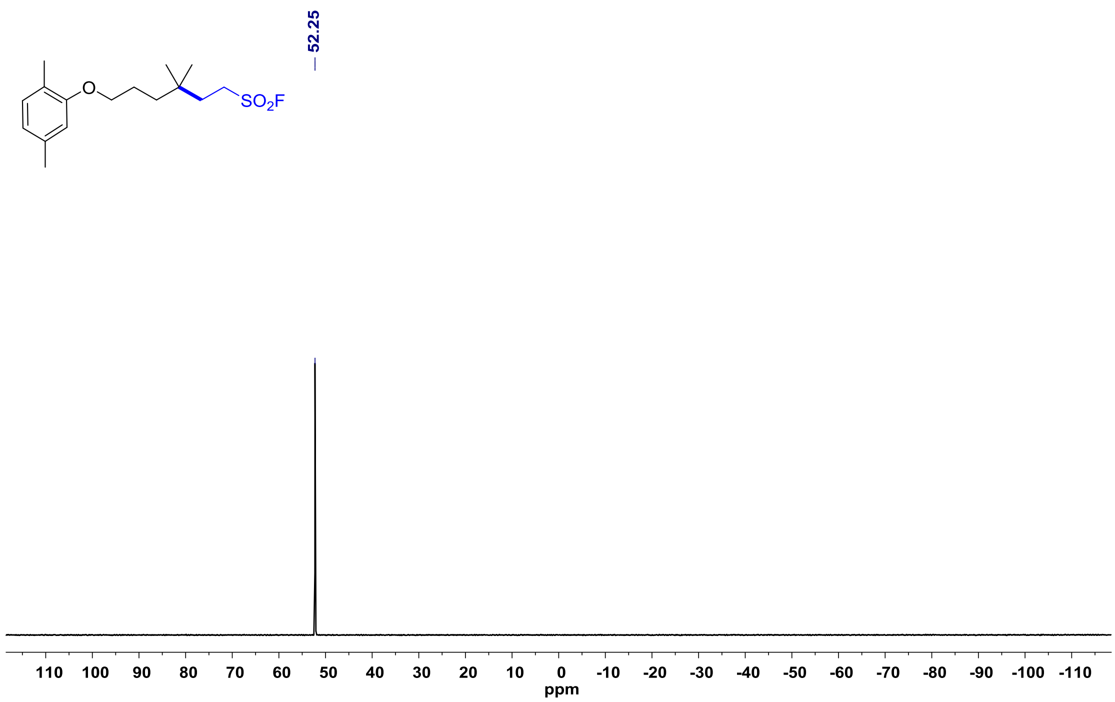

Supplementary Figure 93. ^{19}F NMR spectrum for compound **42**

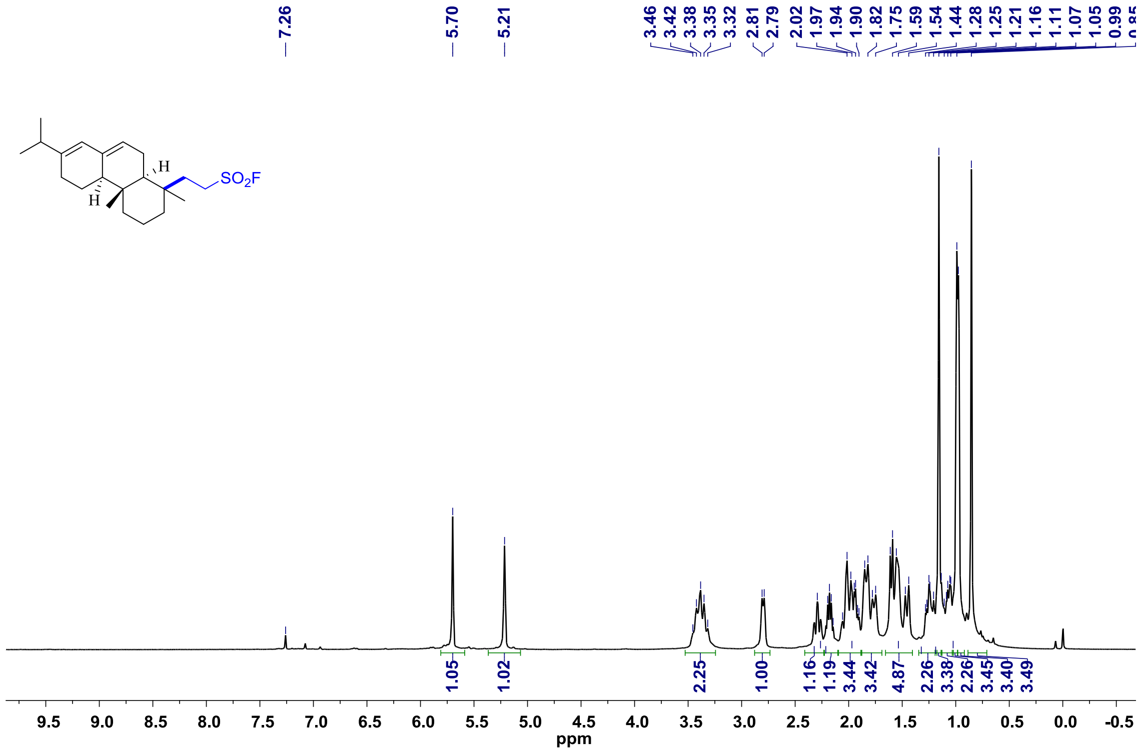

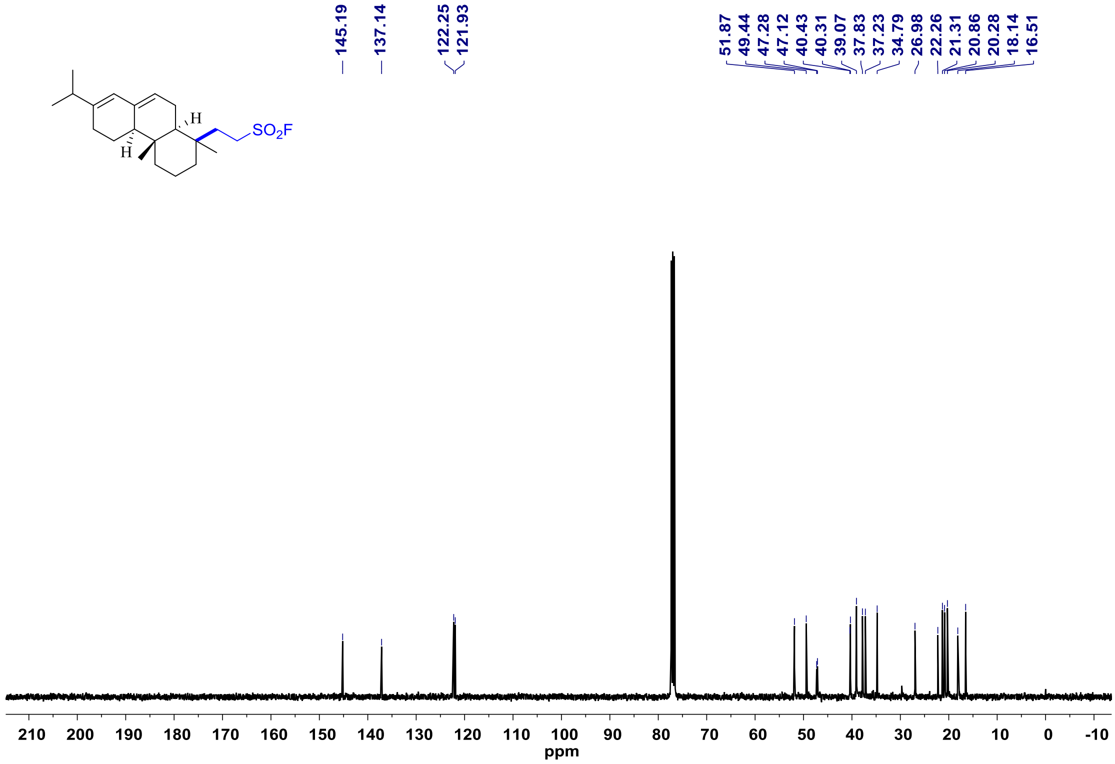

Supplementary Figure 94. ^{1}H and ^{13}C NMR spectra for compound **43**

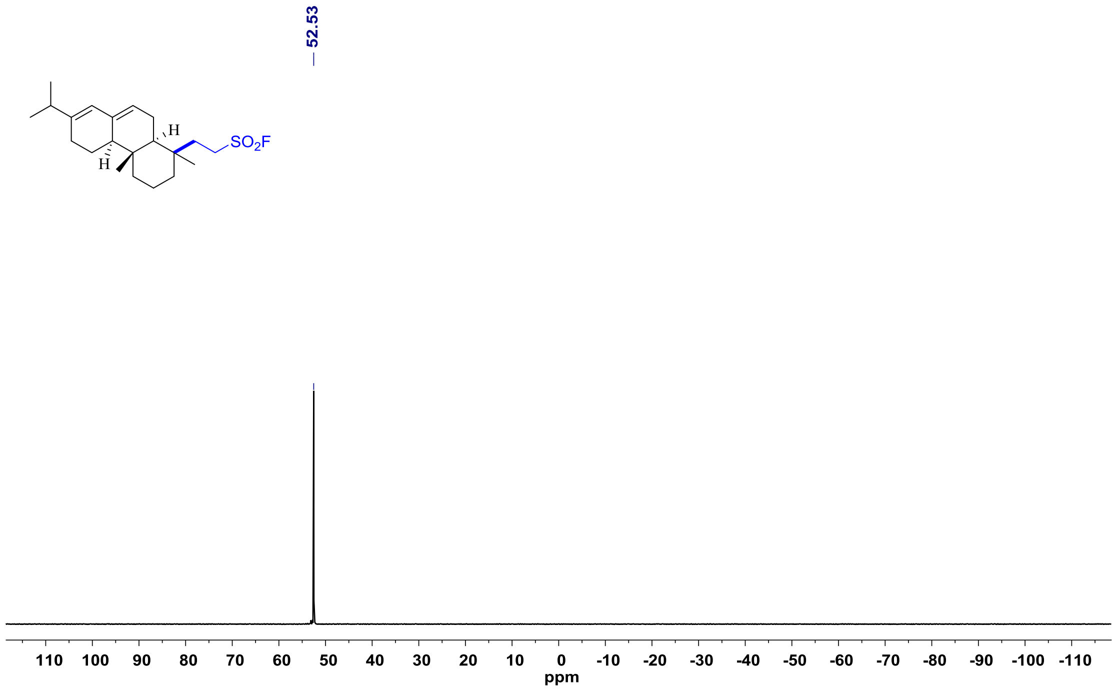

Supplementary Figure 95. ^{19}F NMR spectrum for compound **43**

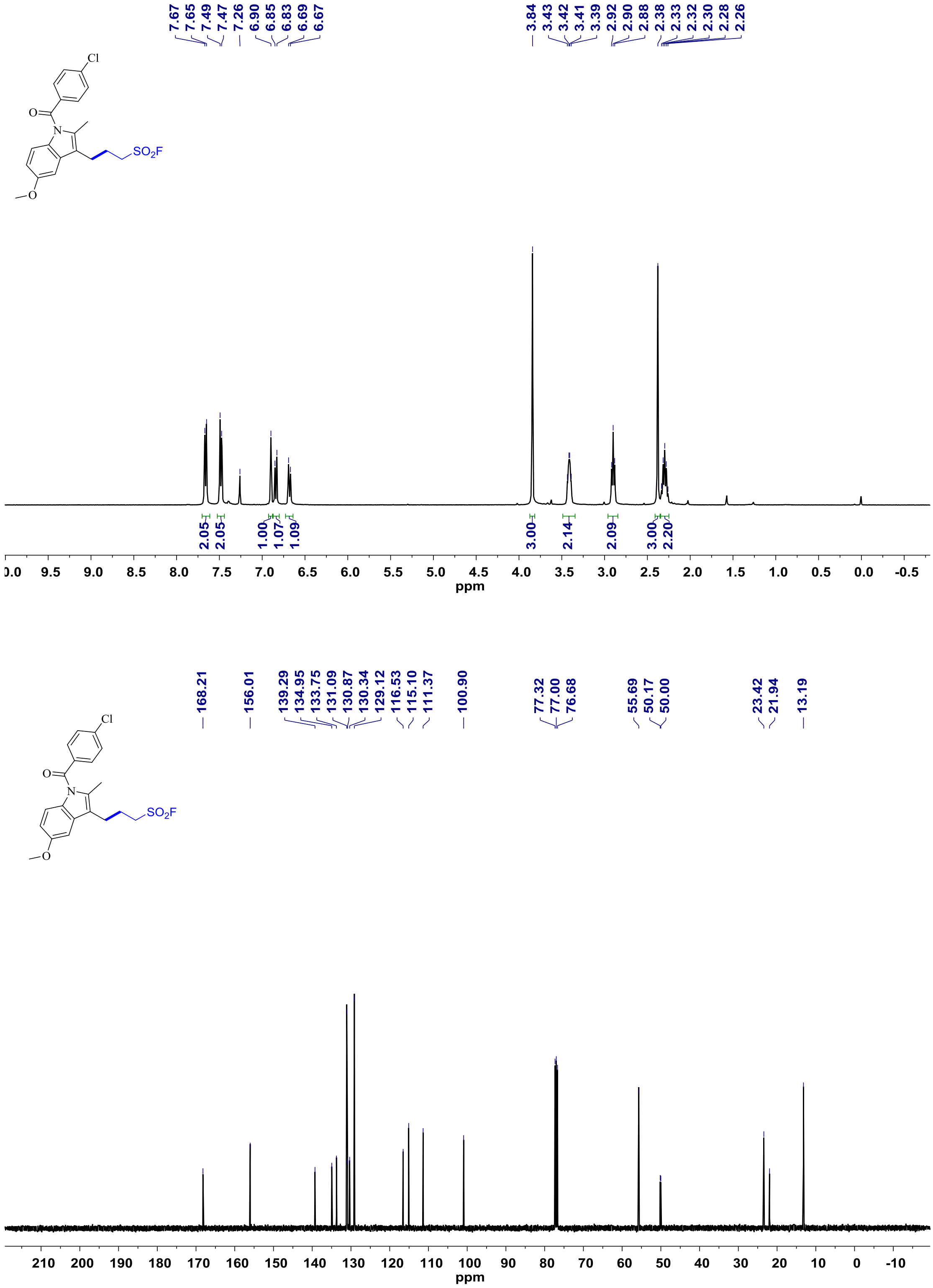

Supplementary Figure 96. ^{1}H and ^{13}C NMR spectra for compound **44**

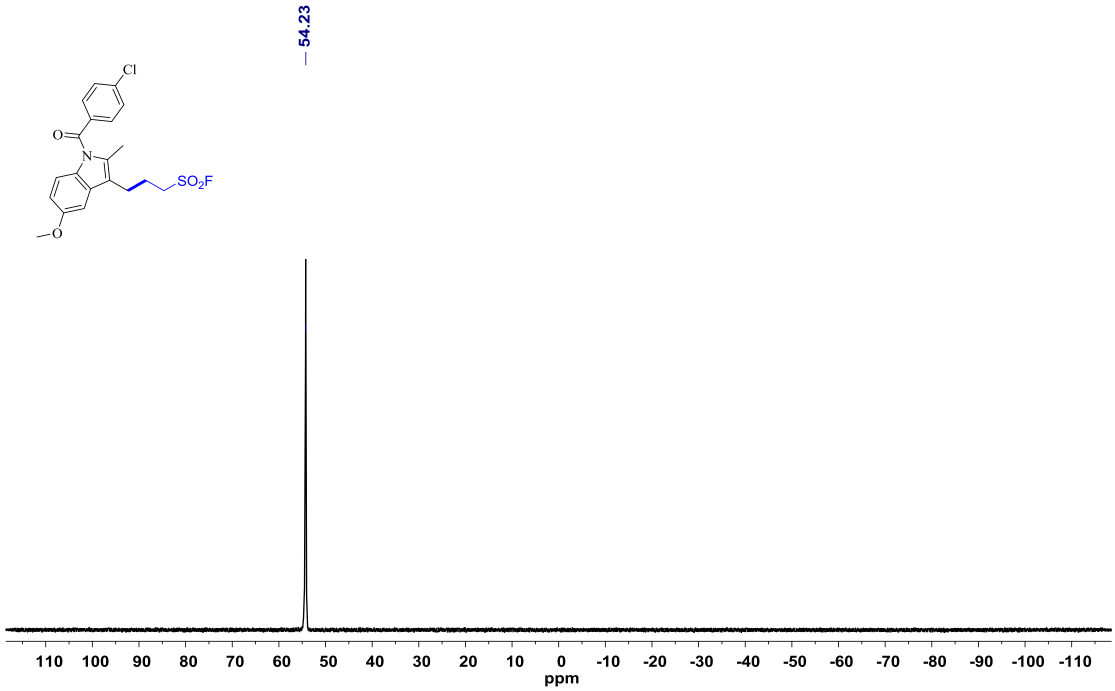

Supplementary Figure 97. ^{19}F NMR spectrum for compound **44**

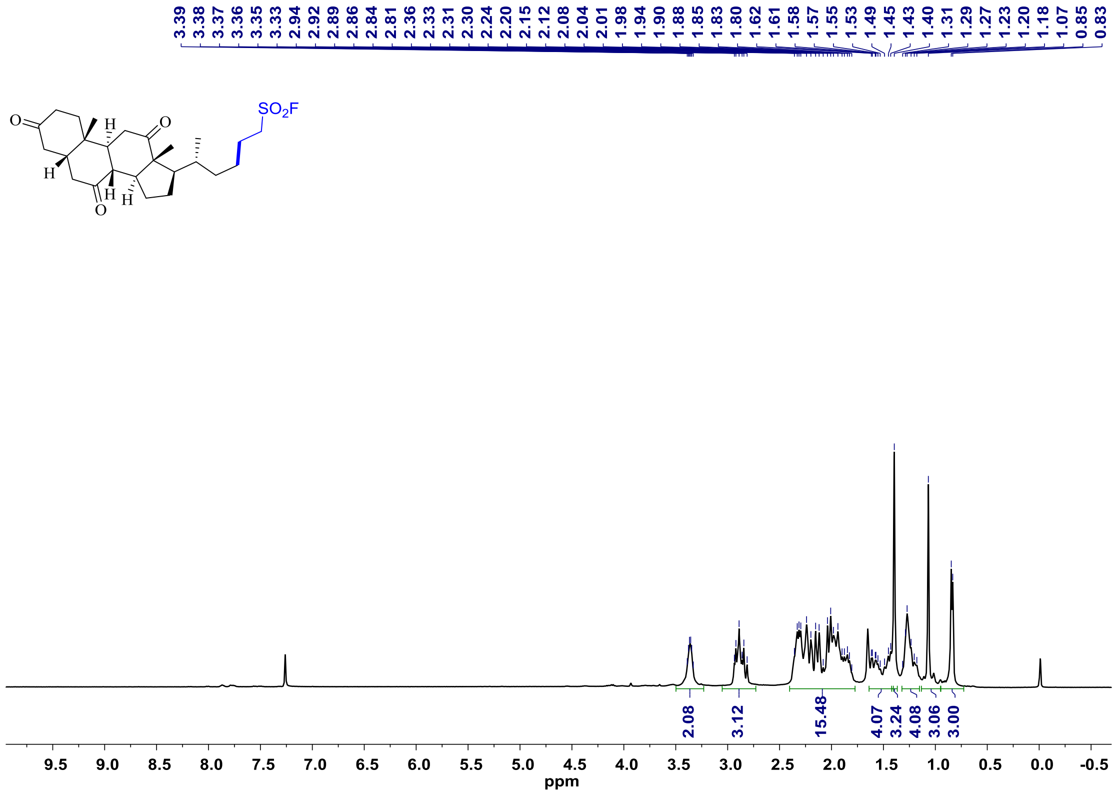

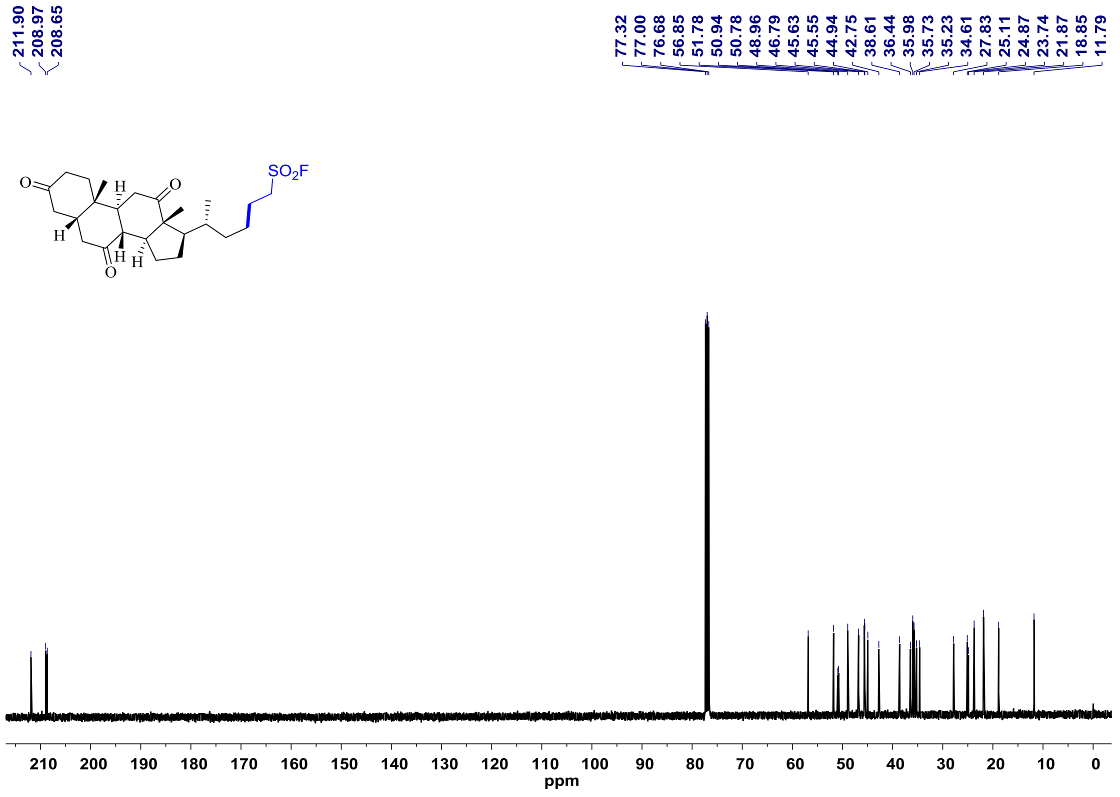

Supplementary Figure 98. ^{1}H and ^{13}C NMR spectra for compound **45**

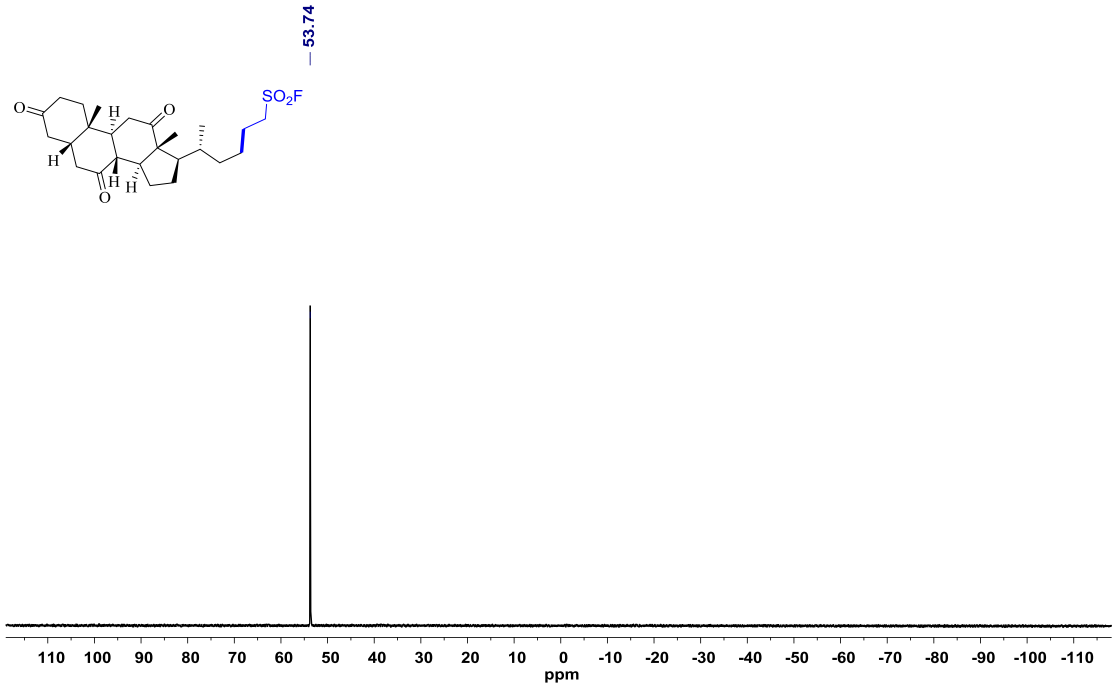

Supplementary Figure 99. ^{19}F NMR spectrum for compound **45**

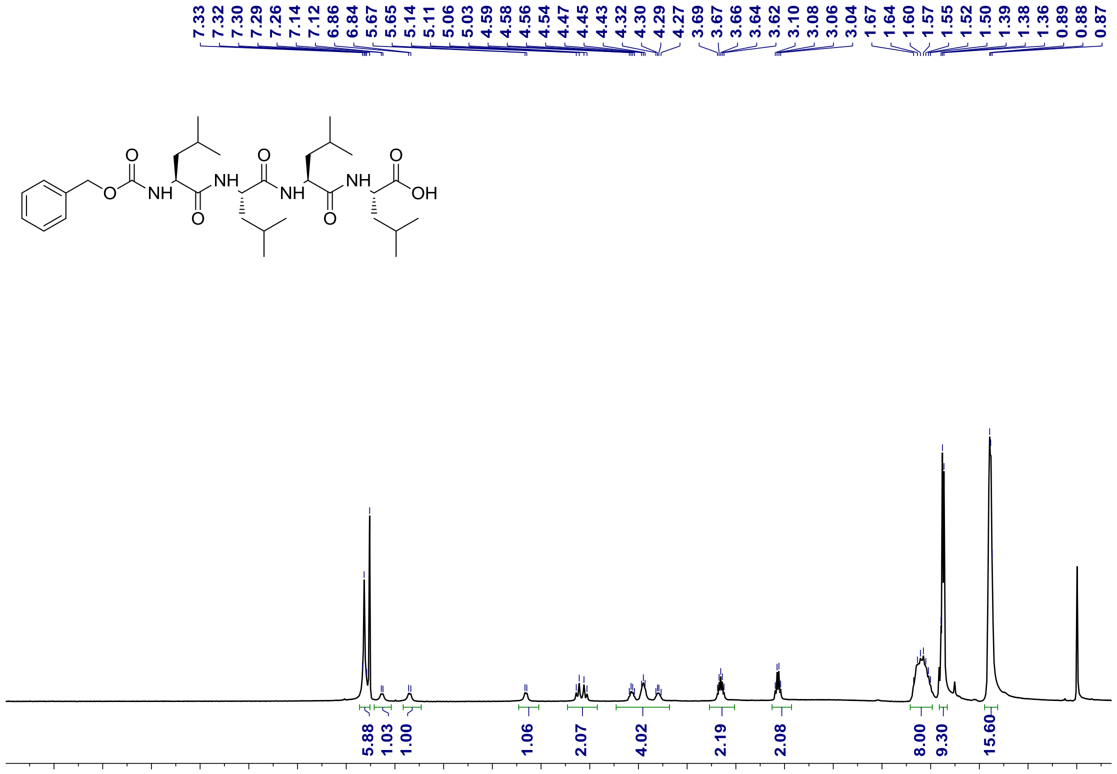

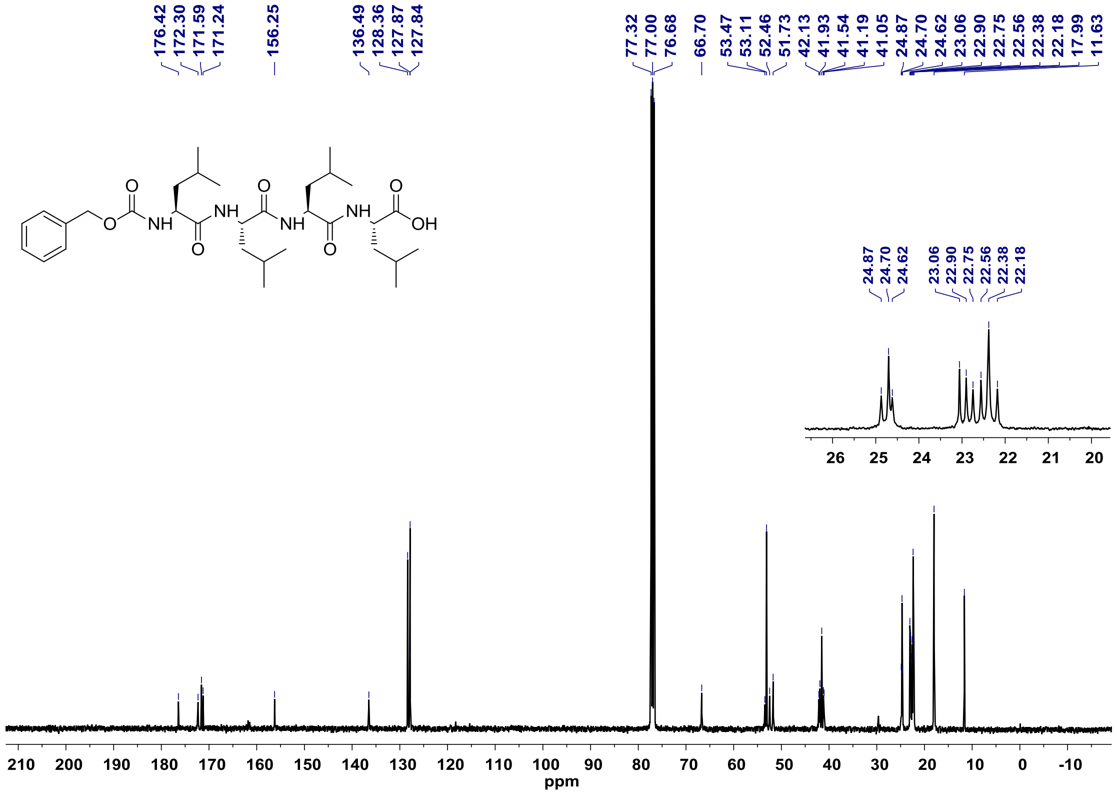

Supplementary Figure 100. ^{1}H and ^{13}C NMR spectra for compound **46**

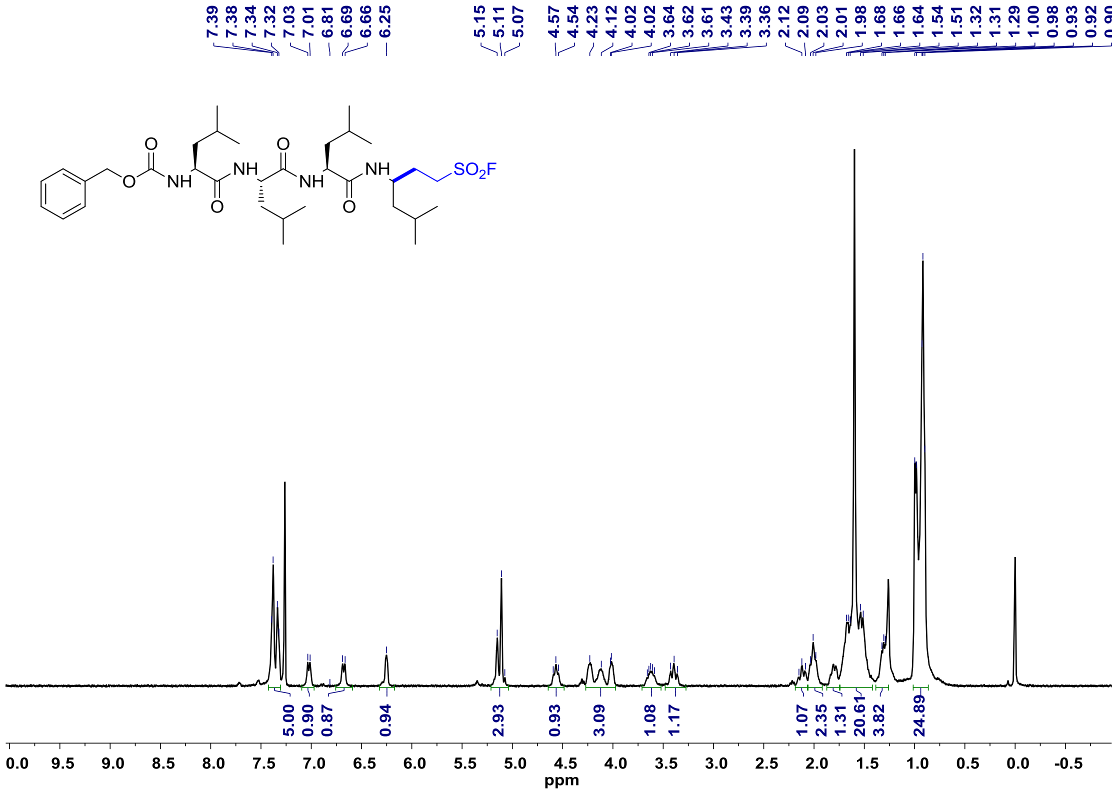

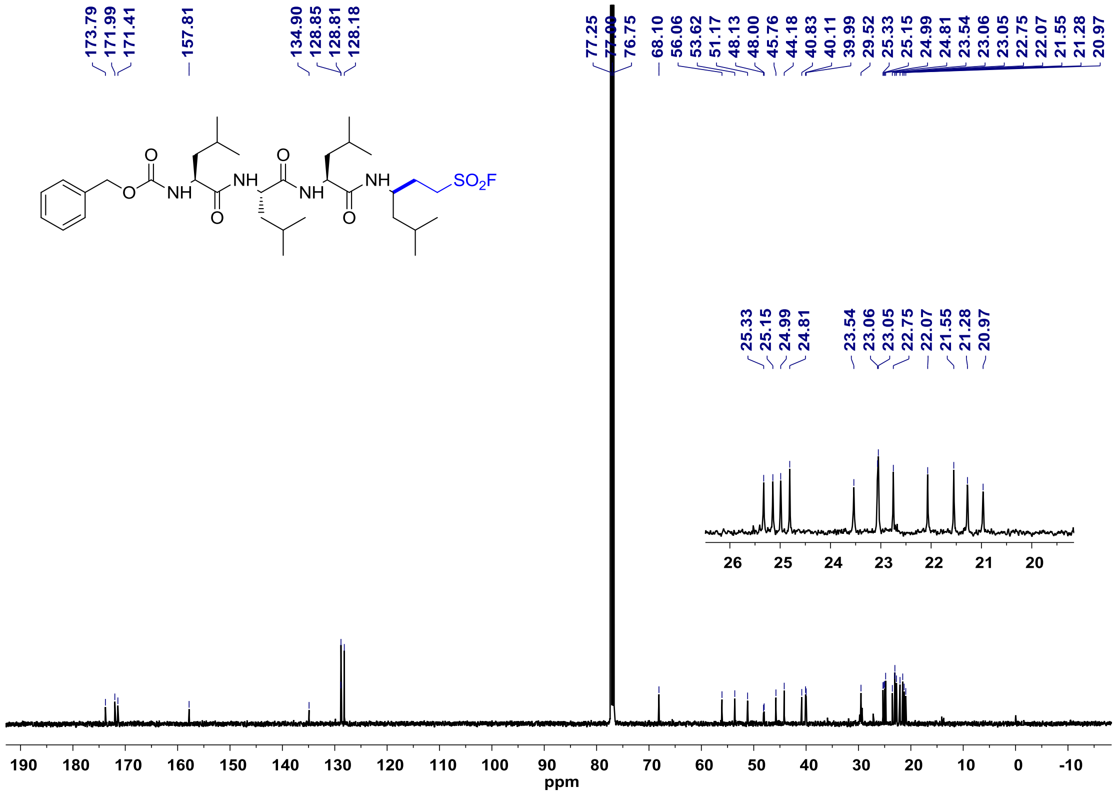

Supplementary Figure 101. ^{1}H and ^{13}C NMR spectra for compound **47**

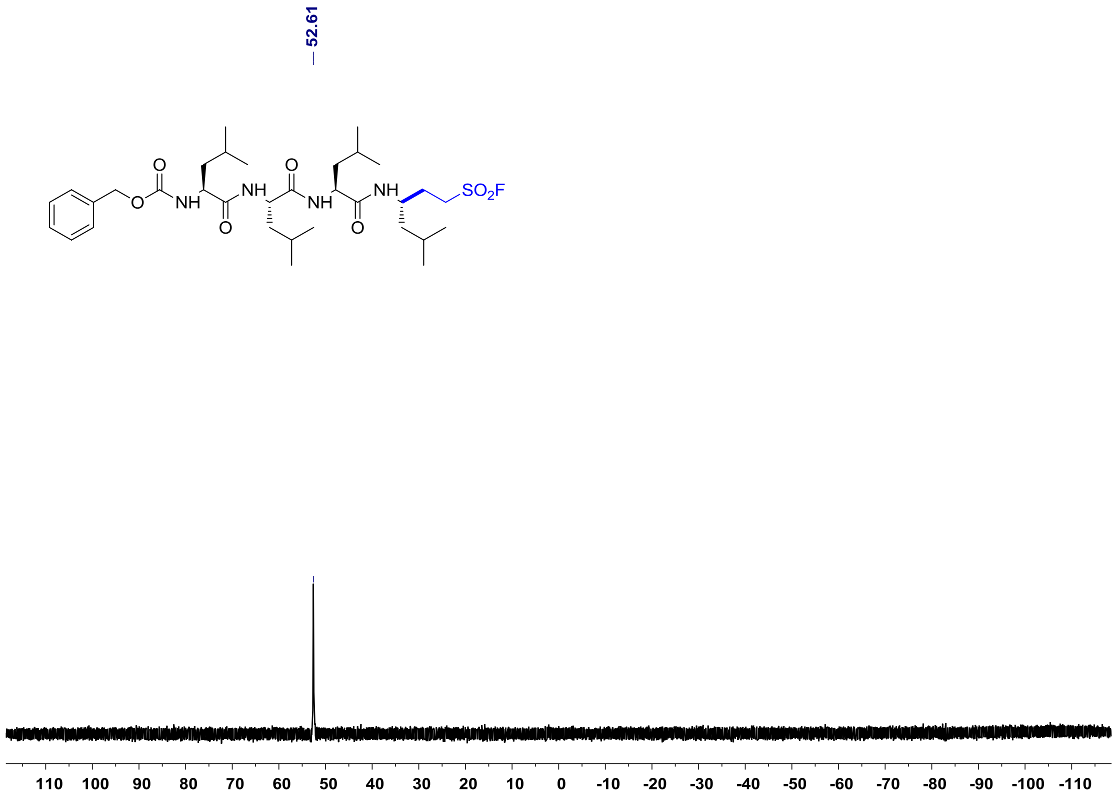

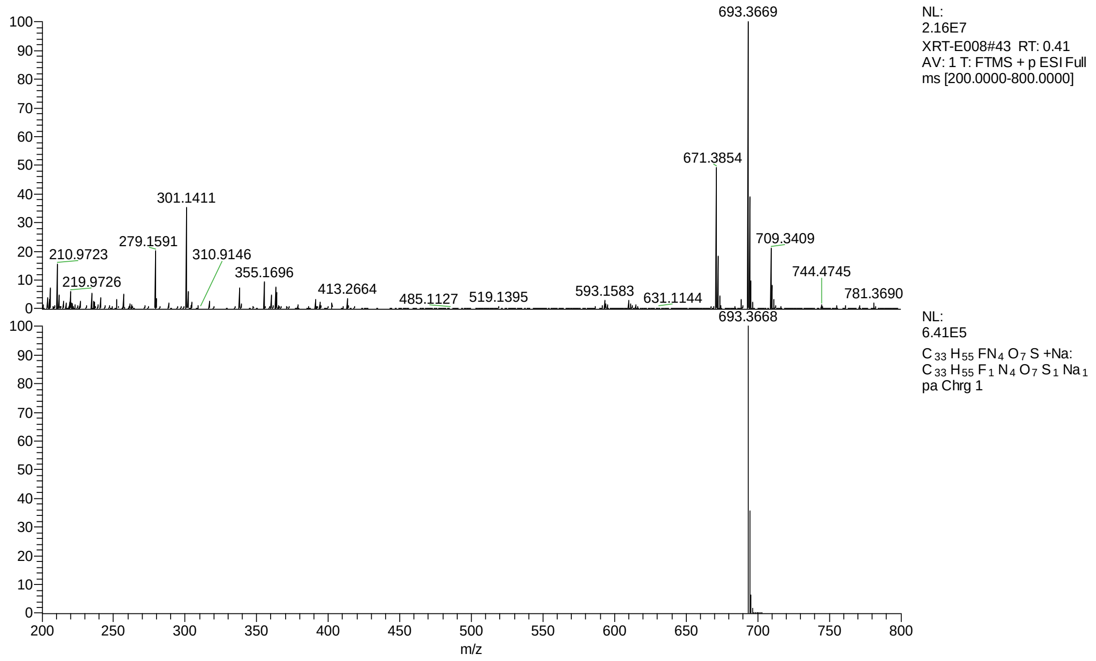

Supplementary Figure 102. ^{19}F NMR and HRMS spectra for compound **47**

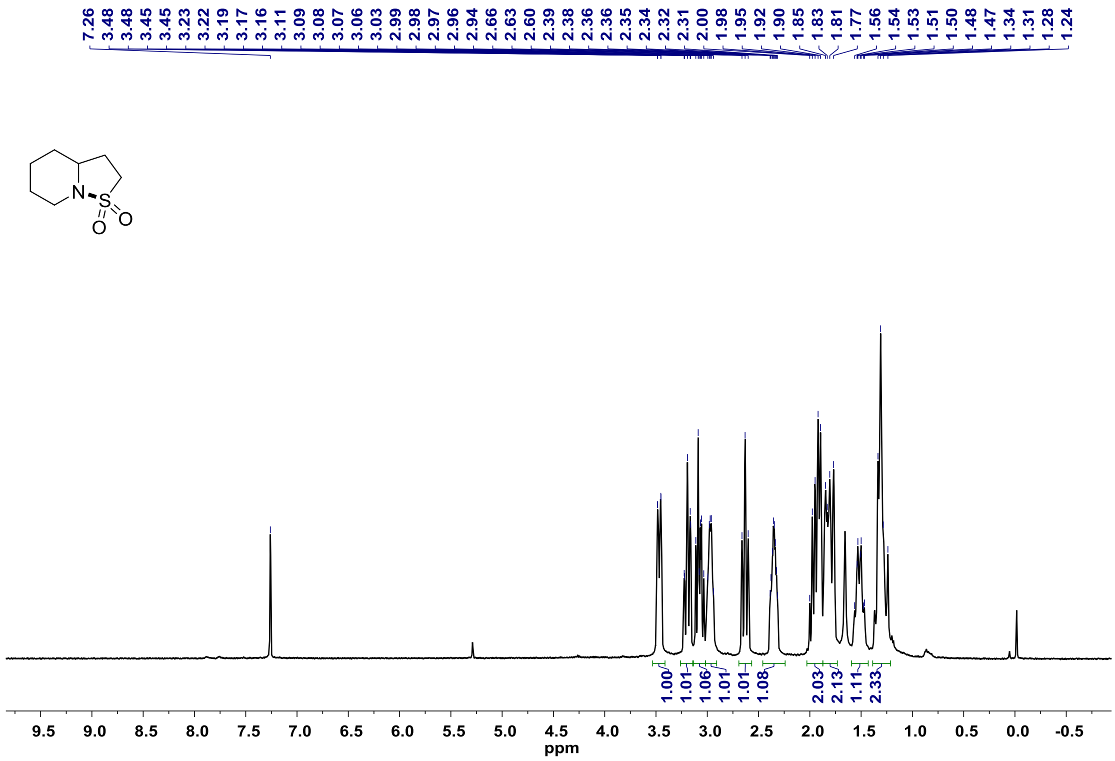

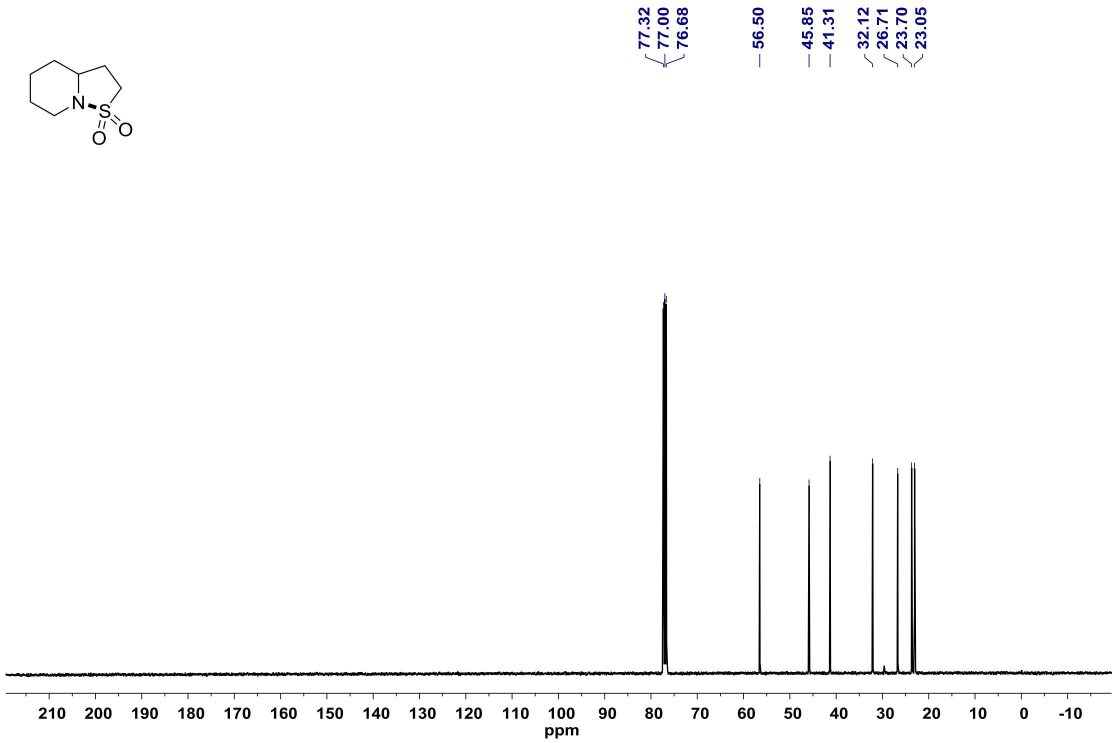

Supplementary Figure 103. ^{1}H and ^{13}C NMR spectra for compound **48**

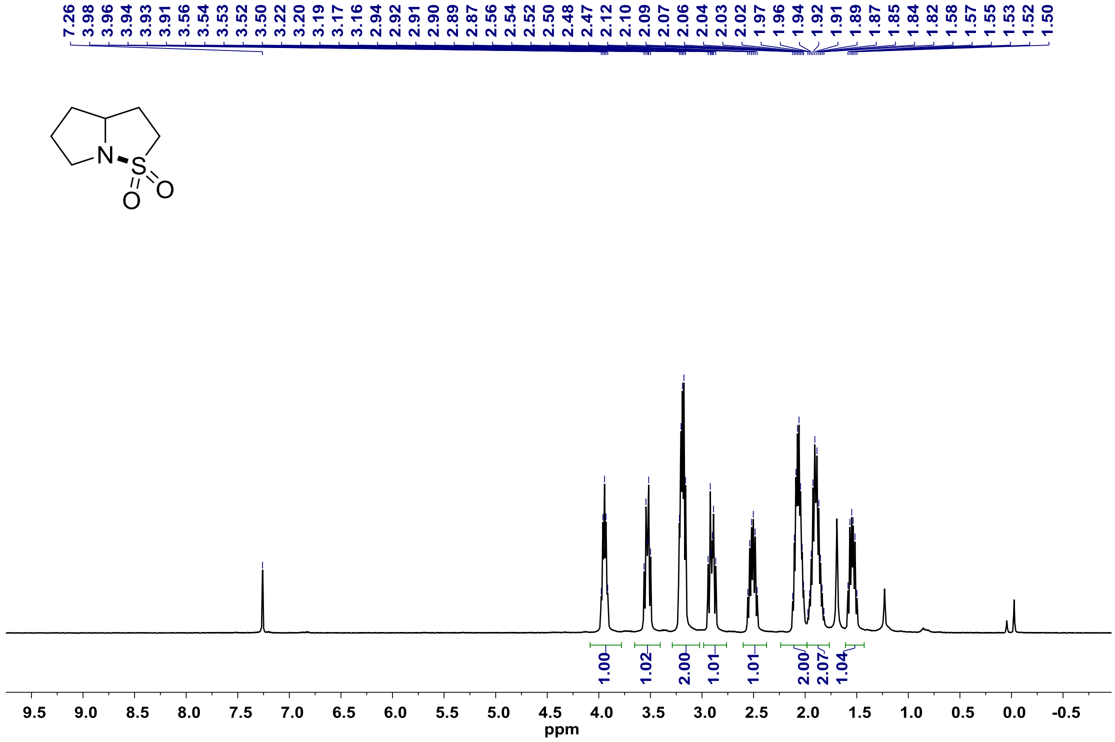

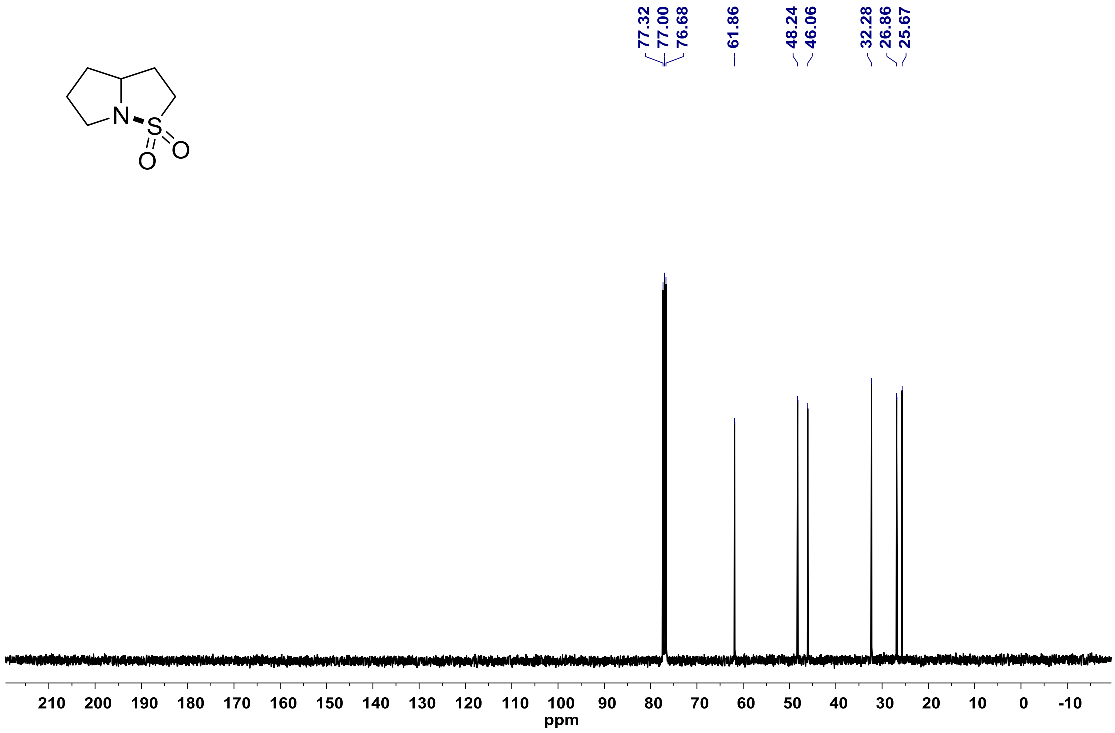

Supplementary Figure 104. ^{1}H and ^{13}C NMR spectra for compound **49**

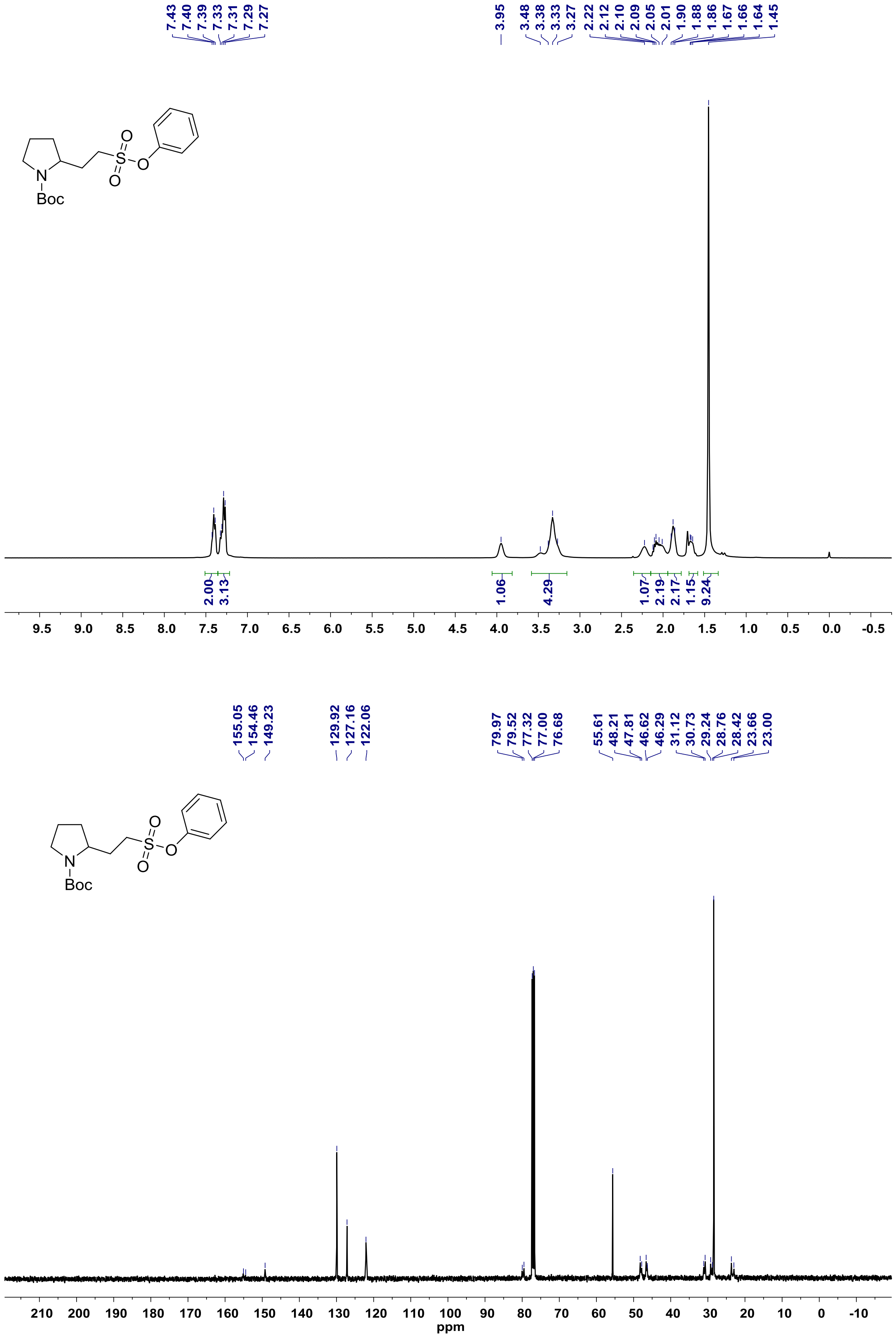

Supplementary Figure 105. ^{1}H and ^{13}C NMR spectra for compound **50**

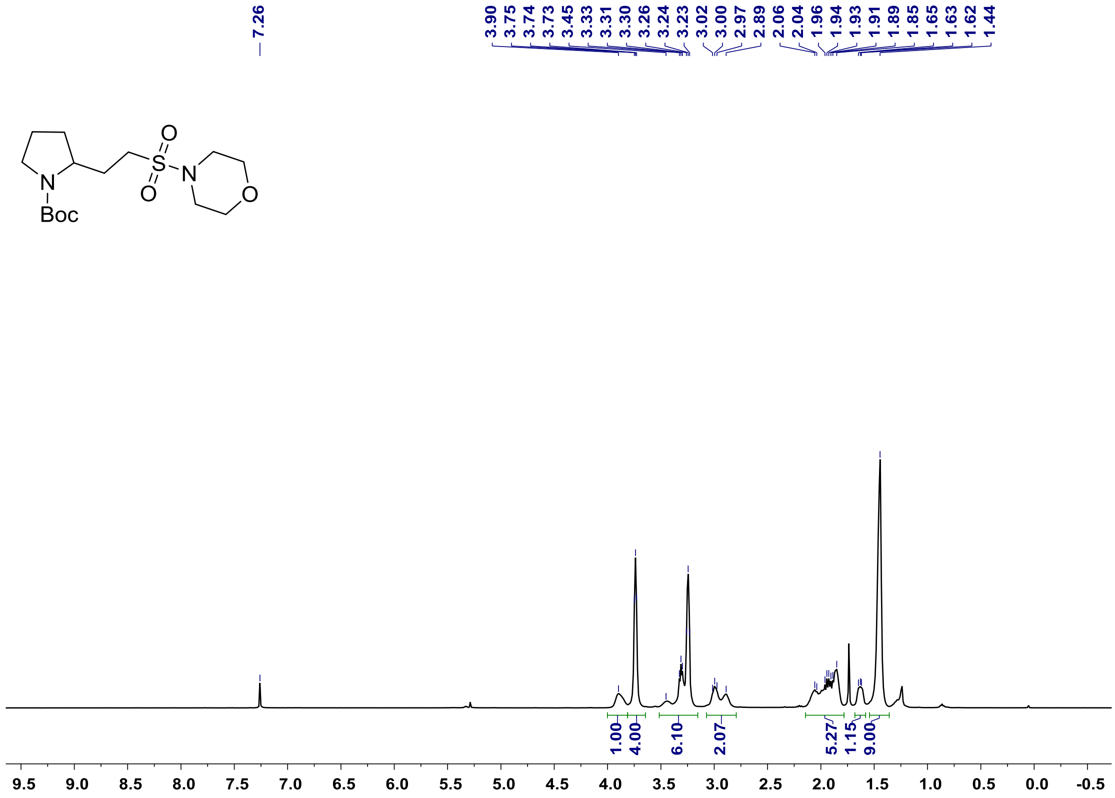

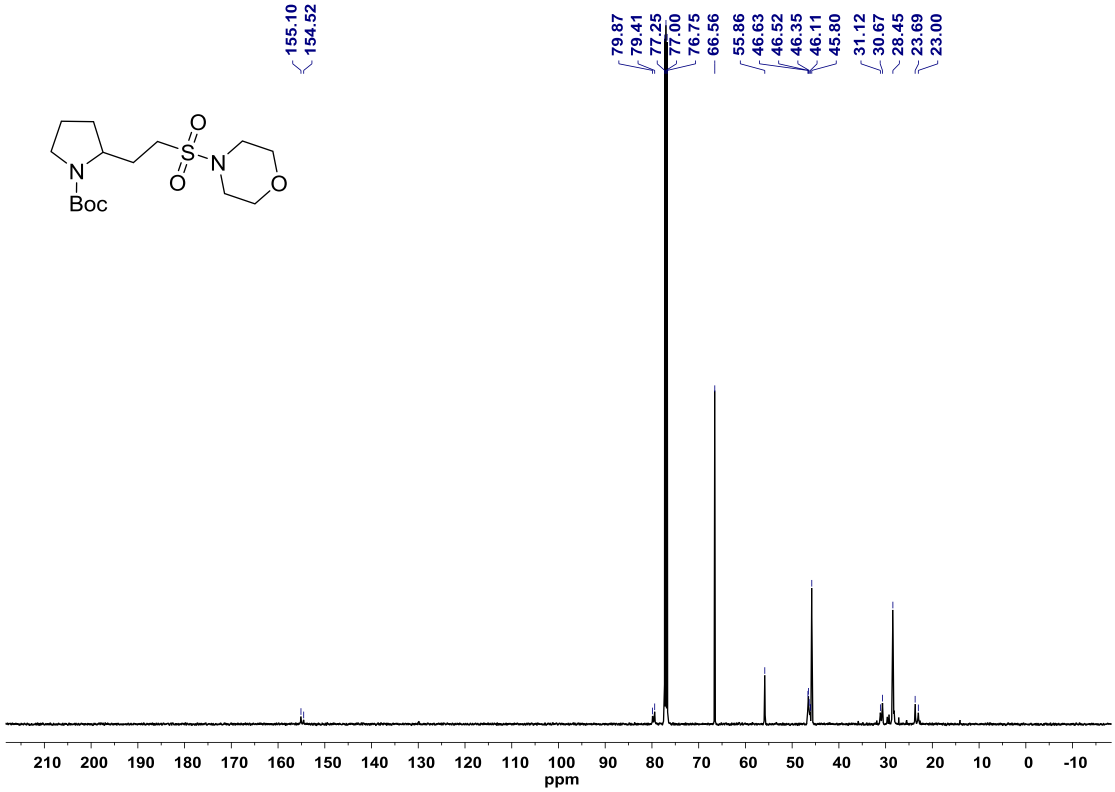

Supplementary Figure 106. ^{1}H and ^{13}C NMR spectra for compound **51**

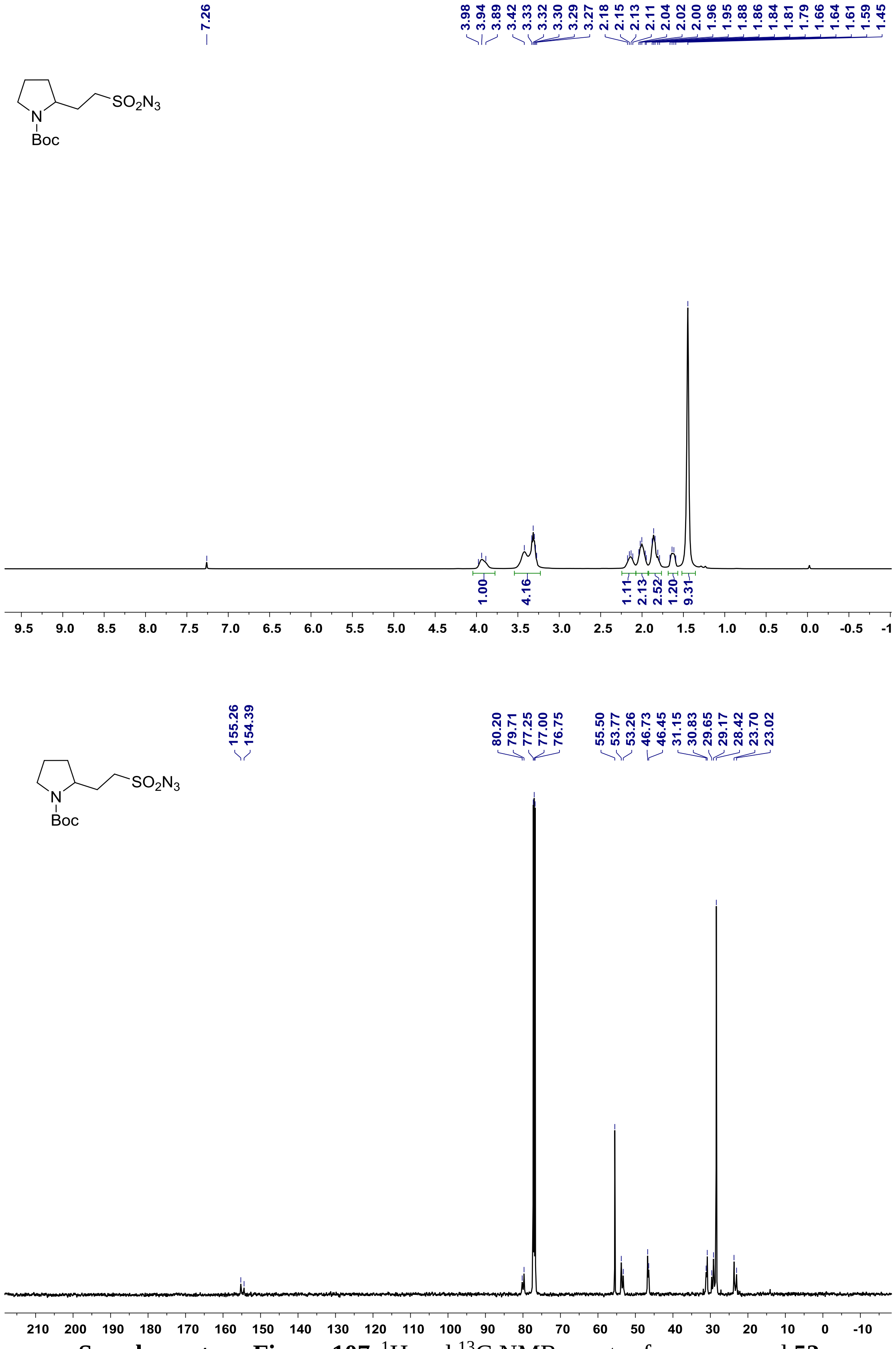

Supplementary Figure 107. ^{1}H and ^{13}C NMR spectra for compound **52**

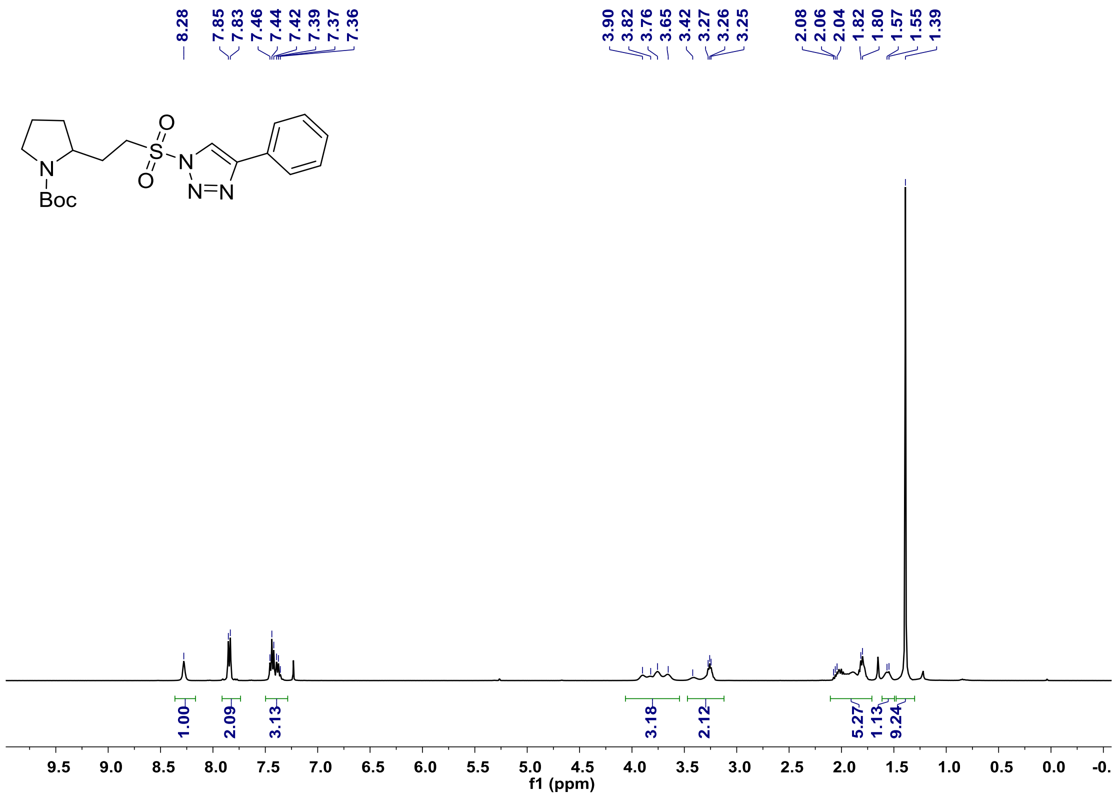

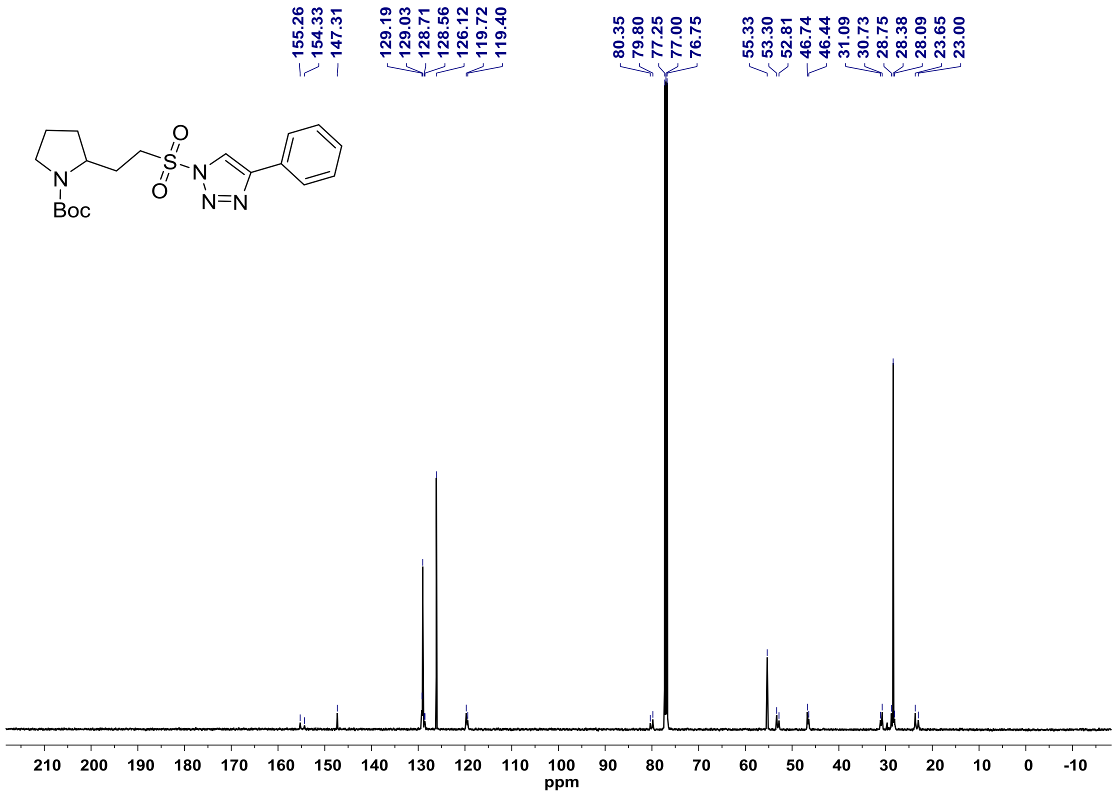

Supplementary Figure 108. ^{1}H and ^{13}C NMR spectra for compound **53**

Supplementary References

1. Zheng Q., Dong J., & Sharpless K. B., Ethenesulfonyl fluoride (ESF): an on-water procedure for the kilogram-scale preparation. *J. Org. Chem.* **81**, 11360–11362 (2016).
2. Cornella J., Edwards J. T., Qin T., Kawamura S., Wang J., Pan C. M., Gianatassio R., Schmidt M., Eastgate M. D., & Baran P. S., Practical Ni-catalyzed aryl–alkyl cross-coupling of secondary redox-active esters. *J. Am. Chem. Soc.* **138**, 2174–2177 (2016).
3. Sheikh M. C., Takagi S., Sakai M., Mori T., Hayashi N., Fujie T., Ono S., Yoshimura T., & Morita, H. Syntheses and reactivities of non-symmetrical “active ester” bi-dentate cross-linking reagents having a phthalimidoyl and acid chloride, 2-benzothiazole, or 1-benzotriazole group. *Org. Biomol. Chem.* **9**, 1244–1254 (2011).
4. Fawcett A., Pradeilles J., Wang Y., Mutsuga T., Myers E. L., & Aggarwal V. K. Photoinduced decarboxylative borylation of carboxylic acids. *Science* **357**, 283–286 (2017).
5. Yu L., Tang M. L., Si C. M., Meng Z., Liang Y., Han J., & Sun X. Zinc-mediated decarboxylative alkylation of gem-difluoroalkenes. *Org. Lett.* **20**, 4579–4583 (2018).
6. Liu X. G., Zhou C. J., Lin E., Han X. L., Zhang S. S., Li Q., & Wang H. Decarboxylative Negishi coupling of redox-active aliphatic esters by cobalt catalysis. *Angew. Chem. Int. Ed.* **57**, 13096–13100 (2018).
7. Zhao W., Wurz R. P., Peters J. C., & Fu G. C. Photoinduced, copper-catalyzed decarboxylative C–N coupling to generate protected amines: an alternative to the Curtius rearrangement. *J. Am. Chem. Soc.* **139**, 12153–12156 (2017).
8. Hu D., Wang L., & Li P. Decarboxylative borylation of aliphatic esters under visible-light photoredox conditions. *Org. Lett.* **19**, 2770–2773 (2017).
9. Qin T., Cornella J., Li C., Malins L. R., Edwards J. T., Kawamura S., Maxwell B. D. Eastgate M. D., & Baran P. S. A general alkyl-alkyl cross-coupling enabled by redox-active esters and alkylzinc reagents. *Science* 352, 801–805 (2016).
10. Schwarz J., & Koenig B. Metal-free, visible-light-mediated, decarboxylative alkylation of biomass-derived compounds. *Green Chem.* **18**, 4743–4749 (2016).

11. Pratsch G., Lackner G. L., & Overman, L. E. Constructing quaternary carbons from N-(acyloxy) phthalimide precursors of tertiary radicals using visible-light photocatalysis. *J. Org. Chem.* **80**, 6025–6036 (2015).

12. Li C., Wang J., Barton L. M., Yu S., Tian M., Peters D. S., Kumar M., Yu A. W., Johnson K. A., Chatterjee A. K., Yan M., & Baran P. S. Decarboxylative borylation. *Science* **356**, eaam7355 (2017).

13. Das M., Vu M. D., Zhang Q., & Liu X. W., Metal-free visible light photoredox enables generation of carbyne equivalents via phosphonium ylides C-H activation. *Chem. Sci.* 10, 1687–1691 (2019).
